# Microtubule-Stabilizing 1,2,4-Triazolo[1,5-*a*]pyrimidines as Candidate Therapeutics for Neurodegenerative Disease: Matched Molecular Pair Analyses and Computational Studies Reveal New Structure–Activity Insights

**DOI:** 10.1021/acs.jmedchem.2c01411

**Published:** 2022-12-19

**Authors:** Thibault Alle, Carmine Varricchio, Yuemang Yao, Bobby Lucero, Goodwell Nzou, Stefania Demuro, Megan Muench, Khoa D. Vuong, Killian Oukoloff, Anne-Sophie Cornec, Karol R. Francisco, Conor R. Caffrey, Virginia M.-Y. Lee, Amos B. Smith, Andrea Brancale, Kurt R. Brunden, Carlo Ballatore

**Affiliations:** † Skaggs School of Pharmacy and Pharmaceutical Sciences, University of California, San Diego, 9500 Gilman Drive, La Jolla, California 92093, United States; ‡ Cardiff School of Pharmacy and Pharmaceutical Sciences, Cardiff University, King Edward VII Avenue, Cardiff CF103NB, U.K.; § Center for Neurodegenerative Disease Research, Perelman School of Medicine, University of Pennsylvania, 3600 Spruce St., Philadelphia, Pennsylvania 19104, United States; ∥ Department of Chemistry & Biochemistry, University of California, San Diego, 9500 Gilman Drive, La Jolla, California 92093, United States; ⊥ Department of Chemistry, School of Arts and Sciences, University of Pennsylvania, 231 South 34th St., Philadelphia, Pennsylvania 19104-6323, United States

## Abstract

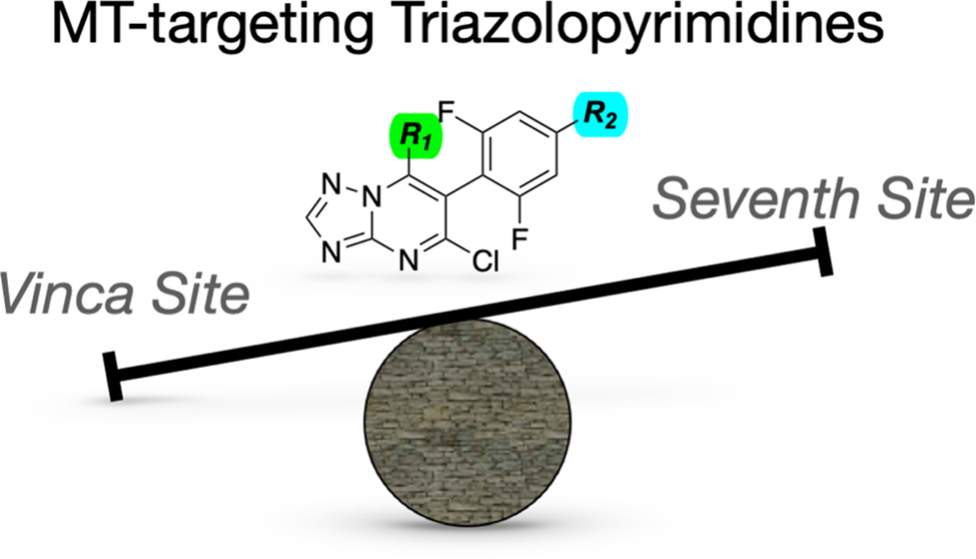

Microtubule (MT)-stabilizing 1,2,4-triazolo[1,5-*a*]pyrimidines (TPDs) hold promise as candidate therapeutics for Alzheimer’s disease (AD) and other neurodegenerative conditions. However, depending on the choice of substituents around the TPD core, these compounds can elicit markedly different cellular phenotypes that likely arise from the interaction of TPD congeners with either one or two spatially distinct binding sites within tubulin heterodimers (*i.e.*, the seventh site and the vinca site). In the present study, we report the design, synthesis, and evaluation of a series of new TPD congeners, as well as matched molecular pair analyses and computational studies, that further elucidate the structure–activity relationships of MT-active TPDs. These studies led to the identification of novel MT-normalizing TPD candidates that exhibit favorable ADME-PK, including brain penetration and oral bioavailability, as well as brain pharmacodynamic activity.

## Introduction

**Figure 1:**
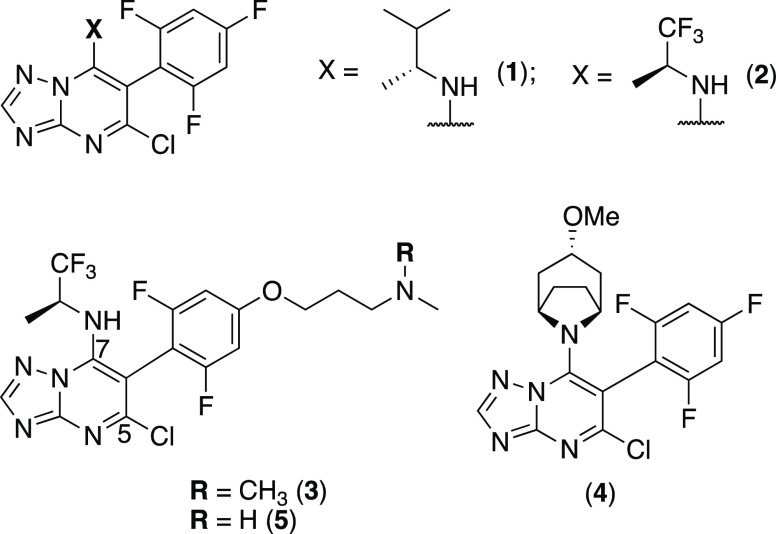
Structure of selected examples of MT-binding triazolopyrimidines.

Neurodegenerative tauopathies are characterized by the hyperphosphorylation and aggregation of the microtubule (MT)-associated protein tau, with evidence of dysregulation of MTs in the axons of neurons that could result in axonal transport deficits and axonal dystrophy that may ultimately contribute to neuronal loss.^1^ Relatively low doses of brain-penetrant MT-stabilizing compounds have been shown to provide therapeutic benefits in mouse models of tauopathy by normalizing axonal MTs and, consequently, by restoring axonal transport.^2−6^ Similarly, MT-stabilizing molecules have been shown to provide benefits in other CNS disease models where MT deficits are thought to occur, including models of amyloid plaque formation,^7^ traumatic brain injury,^8^ and spinal cord injury.^9^ Among the different MT-stabilizing agents tested to date in preclinical animal models,^3−7^ selected members of the 1,2,4-triazolo[1,5-*a*]pyrimidine (TPD) class (*e.g.*, **1**, Figure 1) have been identified as potentially promising candidates due to a generally favorable combination of MT-stabilizing activity and brain penetration. MT-stabilizing TPDs are the first and thus far the only known examples of MT-directed molecules that stabilize MTs through interactions with the vinca site on β-tubulin,^10^ a binding site that is normally targeted by MT-depolymerizing compounds, such as vinblastine. However, cell-based studies from our laboratories demonstrated that different TPD congeners produce significantly different effects on cellular tubulin and MTs.^11^ In particular, short-term (4 h) incubation of QBI293 cells with certain TPD compounds, such as **1** and **2** (Figure 1), produced a dose-dependent increase in markers of stable MTs, such as acetylated α-tubulin (AcTub) and de-tyrosinated α-tubulin (GluTub), without alterations in total tubulin levels. In contrast, other TPD congeners, typified by **3** (Figure 1), caused a bell-shaped dose–response curve with respect to AcTub and GluTub, as well as a proteasome-dependent degradation of tubulin. Considering that MT-normalizing therapies to treat neurodegenerative disease are expected to provide benefits to the extent that they can effectively preserve/restore the normal function of MTs, the different phenotypic responses elicited by TPD congeners are likely to have important ramifications. In this context, preferred candidate compounds would clearly be those that do not cause tubulin degradation. To distinguish the different compounds based on the phenotypic response, MT-active TPDs have been classified as “Class I” if they cause a dose-dependent increase in markers of stable MTs without causing loss of total tubulin, or “Class II” if they cause bell-shaped dose–response curve with respect to AcTub and GluTub and/or a reduction of total tubulin levels. The fragments linked at C6 and C7 of the TPD core were found to play an important role in determining both the phenotypic response and the ADME-PK properties of TPDs.^3,11^ Structure–activity relationship (SAR) studies^12^ revealed that Class I activity generally requires (a) the presence of fluoro substituents at either one or both of the ortho positions of the phenyl ring at C6; (b) an electron-withdrawing group in the para position; and (c) a relatively lipophilic, aliphatic amine at C7. Meanwhile, X-ray co-crystal structures^10,13^ of selected TPD congeners from both classes bound to a tubulin assembly revealed that although the MT-stabilizing Class I compound, **4** (Figure 1), interacts exclusively within the vinca binding site on β-tubulin, the Class II TPD, cevipabulin^14^ (**5**, Figure 1) binds with similar affinity to both the vinca site as well as a related, but spatially distinct, intradimer binding site known as the gatorbulin^15^ or seventh site. Moreover, these studies showed that the proteasome-dependent degradation of tubulin caused by cevipabulin may be a direct consequence of this compound binding to the seventh site, which possibly leads to the release of the nonexchangeable GTP from α-tubulin with resulting unfolding and degradation of tubulin.^13^ Although these SAR and structural biology data provide important insights into the mechanism(s) underlying the different phenotypic responses elicited by TPD congeners in cells, further studies are necessary to better elucidate the role played by the fragments at C6 and C7 in determining the relative affinity for the two binding sites and the resulting effects on MT stability. For example, although our prior SAR studies highlighted the importance of the stereo-electronic properties of the substituent in the para position of the phenyl ring at C6, which is exemplified by electron-donating (*e.g.*, alkoxy) and electron-withdrawing (*e.g.*, nitrile) substituents producing opposite overall effects on the cellular phenotype (Class II and Class I phenotype, respectively), the X-ray structure of **5** bound within the seventh site indicates that the fluorinated amine fragment at C7 may also play a prominent role in triggering tubulin degradation as this fragment establishes direct interactions with the deoxyribose of the nonexchangeable GTP.^13^

To further assess the SAR of TPDs, we have designed and synthesized a series of congeners and conducted matched molecular pair (MMP) analyses. Test compounds were evaluated for MT-stabilizing activity in QBI293 cells, and for their calculated binding energies at the vinca and the seventh site. These analyses further elucidate the interplay between the C6 and C7 fragments in determining the differential affinity of TPDs for the two binding sites and, consequently, the cellular MT phenotype. Moreover, these studies illustrate how docking data can be utilized to predict Class I activity of TPDs. As a result, new Class I TPD congeners were identified that are characterized by the presence of a relatively rigid alkyne-containing side chain at the para position of the aromatic ring at C6 and that are endowed with favorable pharmacokinetic (PK) properties and brain pharmacodynamic (PD) effects. Moreover, this study also identified selected TPD congeners that elicit an unusual cellular phenotype that appears to be hybrid between the Class I and II phenotypes, characterized by a dose-response where higher concentrations show a reduction in activity with respect to MT AcTub modification (*i.e.*, Class II-like) without evidence of a significant decrease in total tubulin levels (*i.e.*, Class I-like). An evaluation of representative Class I, II and hybrid TPDs in a neuronal assay with tau hyperphosphorylation and MT deficits indicates that selected examples of these hybrid compounds act like Class I molecules in the context of neurons by effectively normalizing MTs, suggesting that these compounds may also be considered, in addition to Class I molecules, for further development as candidates for neurodegenerative disease.

## Chemistry

**Scheme 1:**
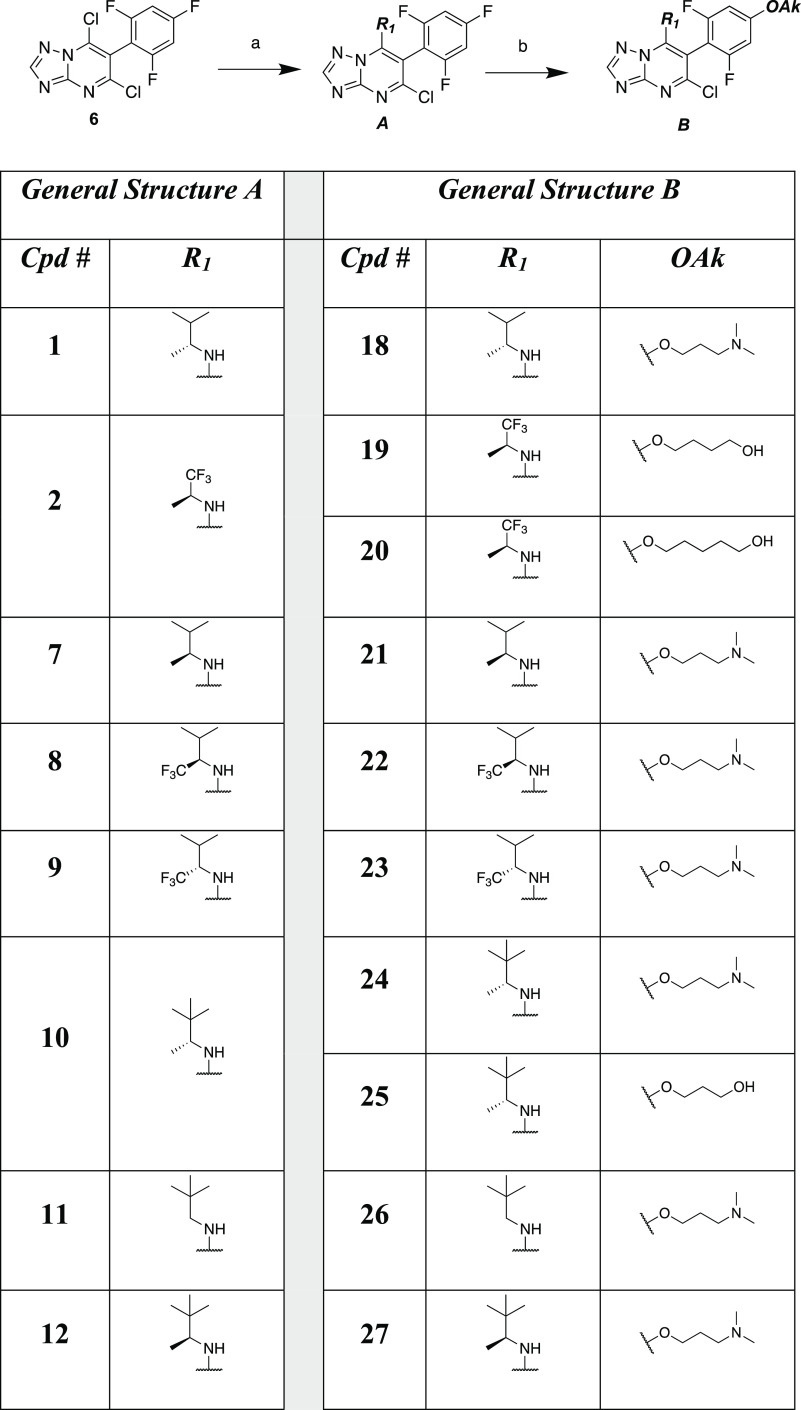

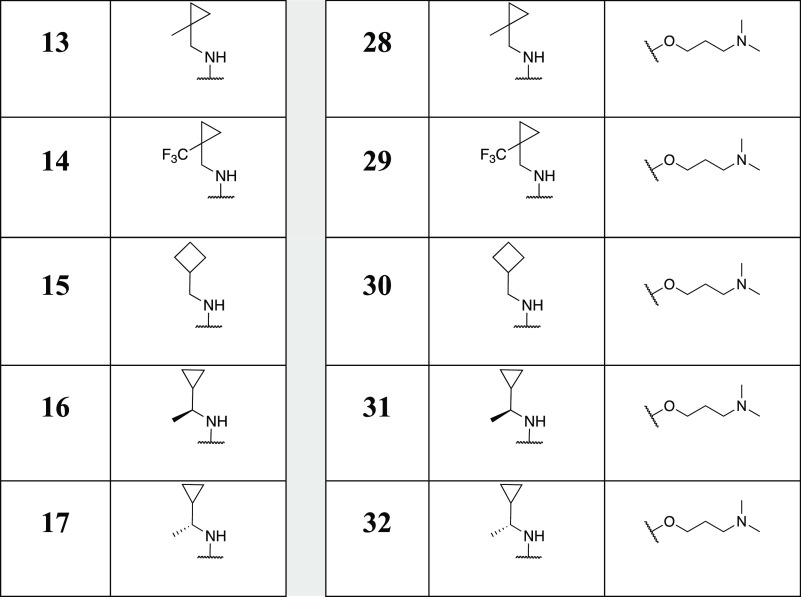


The synthesis of TPD congeners of general structure ***A*** and ***B*** (Scheme 1) started with the TPD dichloride, **6**.^16^ Chemoselective displacement of the chloro-substituent at C7 upon treatment with the appropriate amine fragment furnished TPDs of general structure ***A***, which include previously described **1**,^11^**2**,^14^ and **7**–**12**.^12^ Further treatment of **1**–**17** with the appropriate alcohol in the presence of NaH led to the formation of TPD derivatives of general structure ***B*** (**18**–**32**, Scheme 1).

**Scheme 2:**
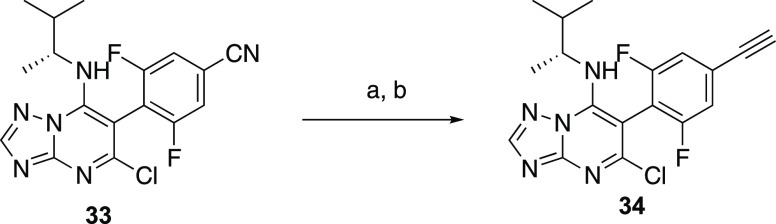


Reduction of the nitrile of TPD **33**(12) to the corresponding aldehyde and conversion of the latter to the alkyne upon treatment with the Bestmann–Ohira reagent^17^ provided acetylene derivative **34** (Scheme 2).

**Scheme 3:**
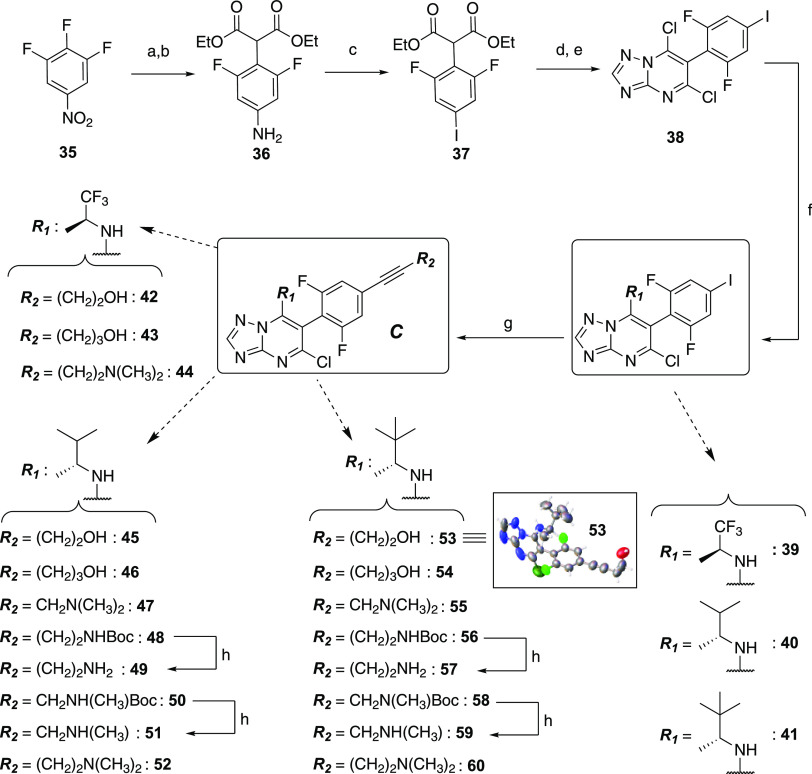


For the synthesis of the disubstituted alkyne derivatives of general structure ***C*** (Scheme 3), the 1,2,3-trifluoro-5-nitrobenzene (**35**)^12^ was treated in an SN_Ar_ reaction with diethylmalonate, followed by a palladium-catalyzed reduction of the nitro group to obtain the corresponding aniline, **36**. A Sandmayer reaction was then used to convert **36** into the corresponding aryl iodide, **37**. Next, condensation of **37** with the aminotriazole, followed by treatment with POCl_3_, led to the expected TPD dichloride, **38**, which was then reacted with the appropriate aliphatic amines to furnish aryl iodides **39**–**41**. Finally, Sonogashira coupling reactions involving the aryl iodides **39**–**41** and the appropriately substituted alkynes led to derivatives **42**–**48**, **50**, **52**–**56**, **58**, and **60**. The structure of **53** was confirmed by X-ray crystallography (Scheme 3 and Supporting Information). Deprotection of the Boc-protecting group of **48**, **50**, **56**, and **58** led, respectively, to final products **49**, **51**, **57**, and **59** (Scheme 3).

## Results

All test compounds were initially screened in the same cell-based (QBI293) assay of MT stabilization used in our previous SAR studies^11,12^ so as to allow direct comparison with literature data. Changes in levels of acetylated α-tubulin (AcTub) and total α-tubulin (α-Tub), relative to vehicle-treated negative control cells, were determined after 4 h of incubation with test compounds at 1 and 10 μM. To account for possible day-to-day differences in the responsiveness of cells to MT-stabilizing treatment, compound-dependent changes in AcTub levels relative to DMSO-treated cells were also normalized to the corresponding changes caused by 100 nM of **5** that was used as a positive control. In addition, docking studies, binding free energy calculations, and in selected cases, molecular dynamic simulations were conducted using an X-ray structure of the vinca and seventh sites (PDB: 5NJH,^10^7CLD^13^). The results of this screening, alongside the observed cellular phenotype and the calculated binding energy of each of the compounds for the vinca site and the seventh site, are summarized in Table 1. For selected compounds, additional studies included testing in the QBI293 cell AcTub assay at additional compound concentrations, and/or evaluation of test compounds for their ability to prevent MT collapse in primary neuron cultures treated with the phosphatase inhibitor, okadaic assay (OA). Finally, cytotoxicity studies in QBI293 and HeLa cells were also conducted for representative examples (Supporting Information).

**Table 1:**
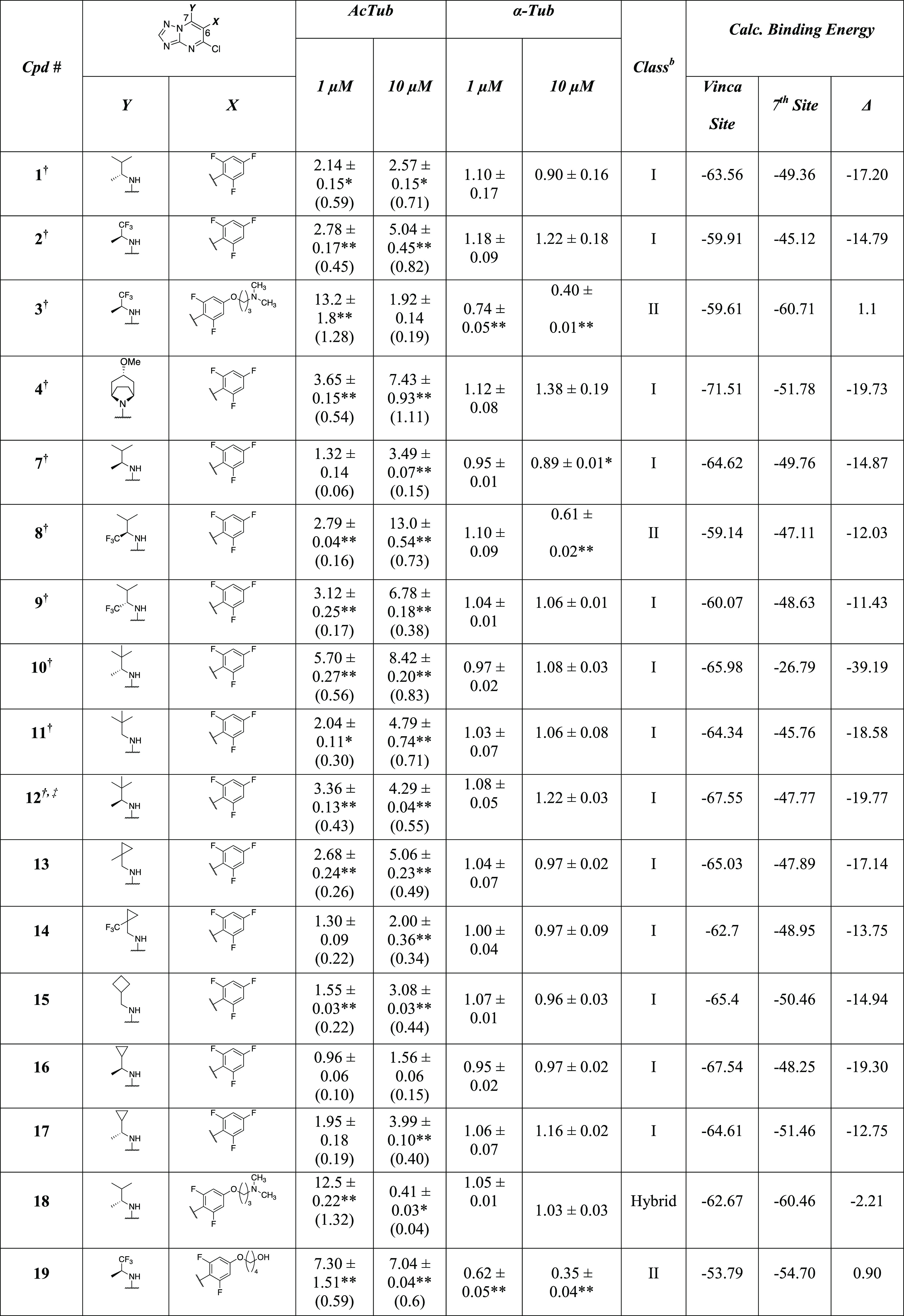

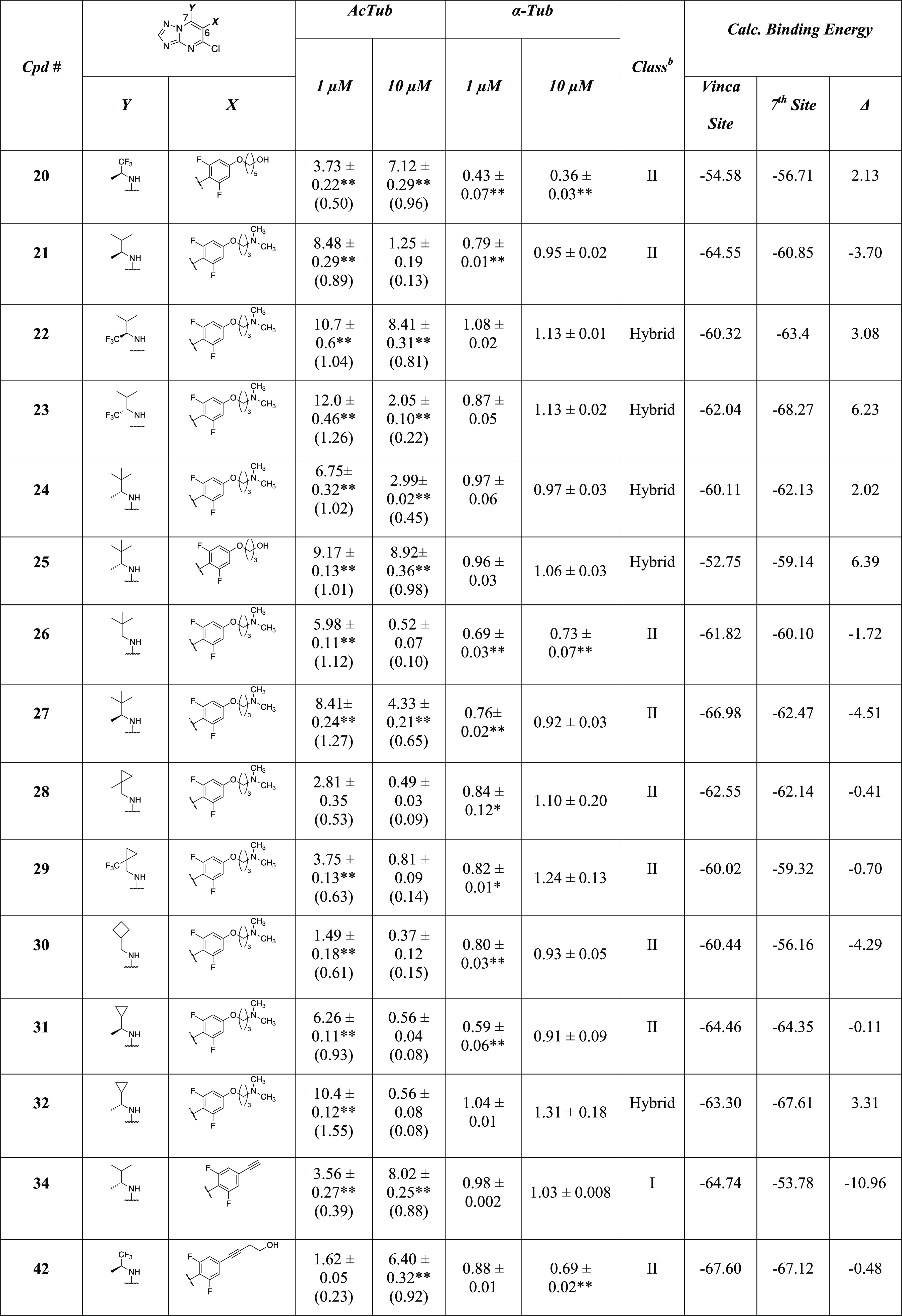

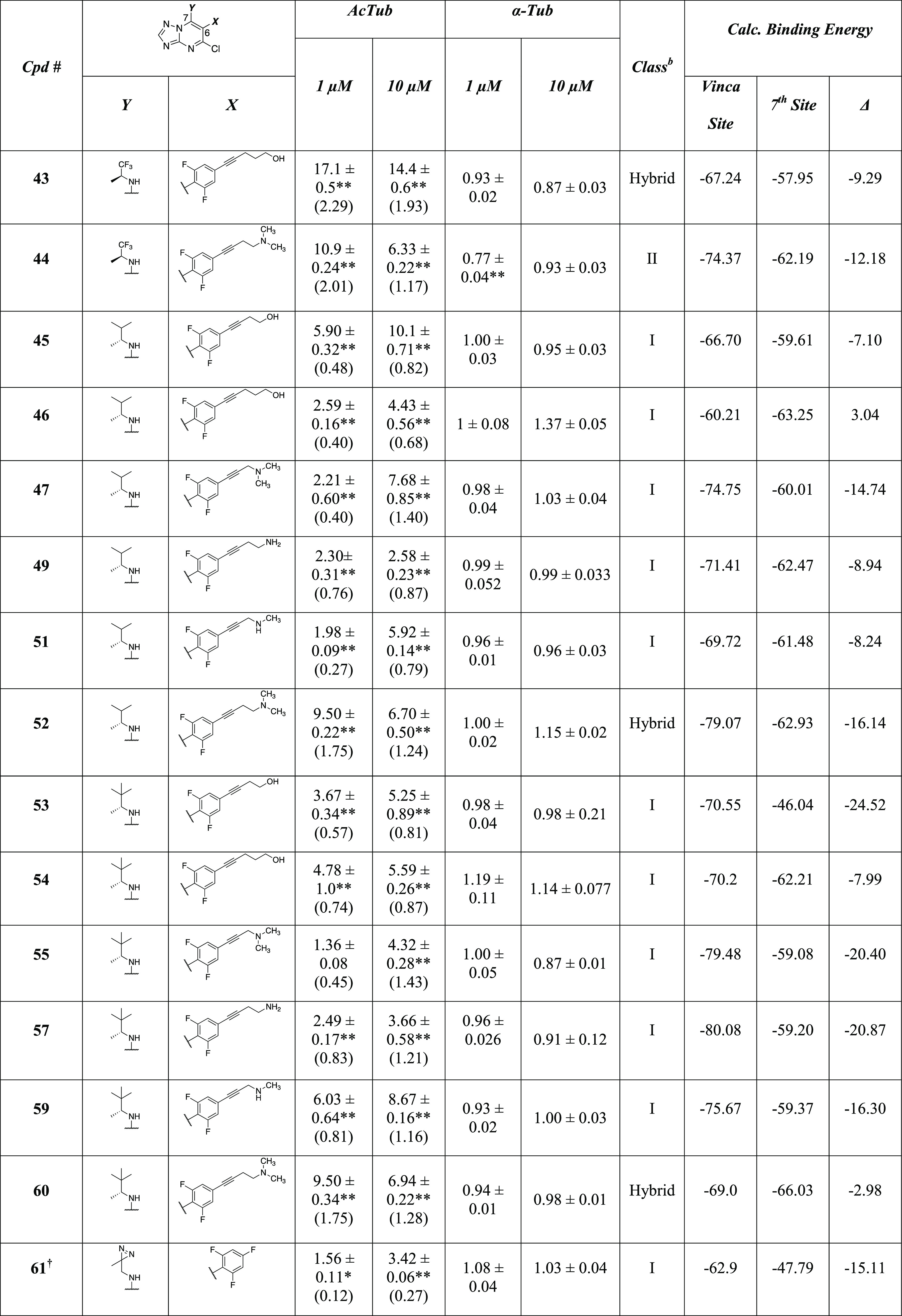

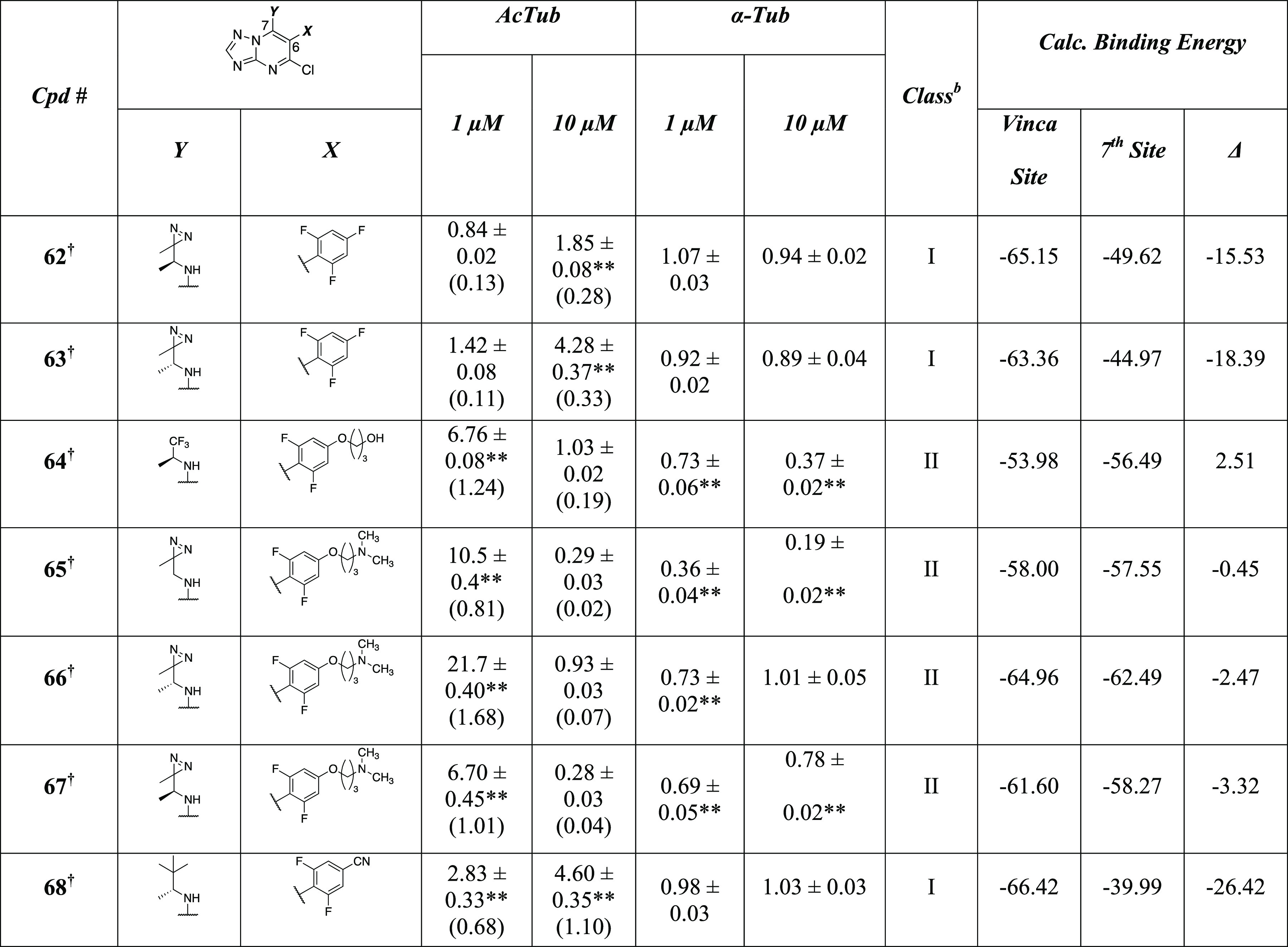
MT-Stabilizing Activity and Calculated Binding Energies for the Vinca Site and the Seventh Site of Test Compounds^a^

a Unless otherwise noted, fold-changes in acetylated α-tubulin (AcTub) and total α-tubulin (α-Tub) levels in QBI293 cells were determined after 4 h incubation with test compounds at either 1 or 10 μM. Reported values for AcTub and α-Tub represent the fold-change compared to vehicle (DMSO)–treated cells (*p < 0.05 and **p < 0.01 by one-way analysis of variance (ANOVA)); numbers in parentheses represent the fold-change of AcTub compared to cells treated with the positive control compound **5** (100 nM).

b Class I compounds are those producing a concentration-dependent increase in AcTub levels and that do not cause >15% reduction in α-tubulin at either concentration; Class II compounds are those that cause >15% decrease in α-tubulin at either 1 or 10 μM compound concentration. Hybrid compounds are those producing elevations in AcTub levels at 10 μM that are lower than the corresponding levels obtained at 1 μM but that do not cause >15% reduction in α-tubulin at either concentration. ^†^The activity in the AcTub and α-tubulin assays have been reported in previous studies^11,12^ and are shown here for comparison. ^‡^Compound was tested at 10 and 30 μM.

**Figure 2:**
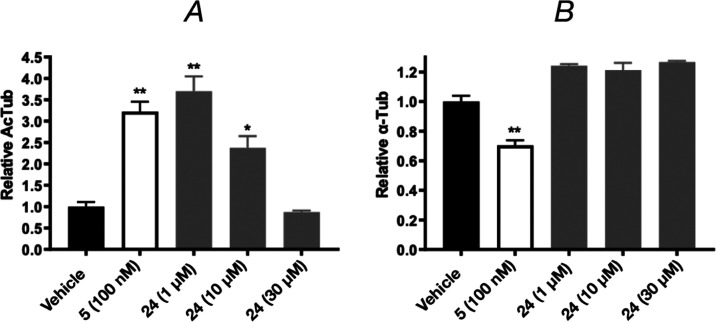
ELISA measuring acetylated AcTub levels (A) and total α-Tub levels (B) in response to 4-h treatment with TPD **5** (100 nM, used as positive control) or **24** at different concentrations. Error bars represent SEM, with *, *p* < 0.05 and **, *p* < 0.01 relative to vehicle as assessed by one-way ANOVA.

The data summarized in Table 1 allow for several comparisons between series of MMPs that differ only by a single chemical transformation. First, a comparison of **3** with 15 match paired congeners (**18**, **21**–**24**, **26**–**32**, **65**–**67**) bearing different amine fragments at C7, including examples of fluorinated (**22**, **23**, and **29**) and nonfluorinated amines, reveals that none of the 15 analogues exhibit Class I activity. However, while 10 of these analogues (**21**, **26**–**31**, **65**–**67**), like **3**, produce a significant reduction (*i.e.*, >15%) of total α-Tub levels when tested at 1 and/or 10 μM (*i.e.*, Class II phenotype), in other cases (**18**, **22**–**24**, and **32**), the replacement of the fluorinated amine of **3** resulted in derivatives that do not produce a meaningful reduction in total tubulin levels at either concentration. However, similar to the parent TPD **3**, these latter matched pair compounds produce an unusual dose–response in the AcTub assay as evidenced by relative increases in this marker of stable MTs that are more pronounced at the low compound concentration (1 μM) than at the high concentration (10 μM). To exemplify and confirm this phenomenon, Figure 2 illustrates a representative example (**24**) that was also tested at a higher (30 μM) concentration, showing a further concentration-dependent decrease in cellular AcTub without a reduction of total α-Tub.

We refer to these compounds as “hybrids” since in QBI293 cells they elicit an unusual dose-dependence in the AcTub assay, which is often seen in Class II compounds (*e.g.*, **3**, **21, 26**–**31, 65**–**67**), without reduction in total tubulin levels, which is a defining feature of Class I compounds (*e.g.*, **1** and **68**).

The observation that the replacement of the amine fragment of **3** can result in analogues that no longer trigger degradation of tubulin appears to be in general agreement with Yang et al.^13^ and suggests that the fluorinated amine fragment of **3** plays an important role in contributing to the Class II activity of this compound and likely other related congeners (*e.g.*, **64**). However, our data also show that the presence of a fluorinated amine at C7 *per se* may not be necessary nor sufficient for Class II activity. This is exemplified by the fact that TPDs bearing fluorinated amines at C7, such as **22** and **23**, exhibit similar hybrid behavior as **24**, and that different TPDs bearing nonfluorinated amines at C7 (*e.g.*, **21**, **26**, **27**, **28**, **30**, **31**, **65**–**67**) produce the Class II defining >15% reduction in total tubulin at either 1 or 10 μM as observed with **3**.

Further comparison of **3** and the above-mentioned analogues (**18**, **21**–**24**, **26**–**32**, **65**–**67**) with the corresponding matched pair compounds in which the alkoxide side chain is replaced by a fluorine atom showed that in all cases but one (MMP **22**–**8**), substitution of the alkoxide side chain with a fluorine results in compounds with Class I activity (see MMPs: **3**–**2; 21**–**7**; **23**–**9**; **24**–**10**; **26**–**11**; **27–12; 28**–**13**; **29**–**14**; **30**–**15**; **31**–**16**; **32**–**17**; **18**–**1**; **65**–**61**; **66**–**63**; **67**–**62**). These results agree with our prior SAR studies^12^ and confirm that in the majority of cases the presence of the alkoxide side chain of **3** and related congeners (*e.g.*, **19**, **20**, and **64**) is conducive to eliciting the Class II phenotype. Moreover, although **2** was found to produce a reduction in total tubulin levels in Yang et al.,^13^ evaluation of this compound in the QBI293 AcTub assay at higher concentrations did not produce evidence of significant reduction of total tubulin levels (see the Supporting Information, Figure S1A). The reason for this discrepancy is not clear, although it may be due to the different cell-type used for the studies (HeLa cells were used in Yang et al.) and/or the different incubation time used in the experiments (16 h incubation time was used in Yang et al.). However, the observation that selected derivatives of **3** (**18, 22**–**24**, and **32**) and **64** (**25**) show hybrid behavior and do not trigger reductions in total tubulin levels clearly highlights the importance that specific aliphatic amines at C7 can have and suggests a more complex interplay between the nature of the amine fragment at C7 and the substituent in the para position of the aromatic ring at C6 in determining the phenotype of TPDs. This situation is also highlighted by comparing MMPs that differ only by the chiral configurations of the amine fragment. In the case of TPDs equipped with an alkoxide side chain, the chiral configuration of the amine was found to have an impact on the phenotype in the pairs **18**–**21**, **24**–**27**, and **31**–**32**, but not in the corresponding compounds bearing a fluorine atom in the para position (*cf.*, **1**–**7**, **10**–**12**, and **16**–**17**).

**Figure 3:**
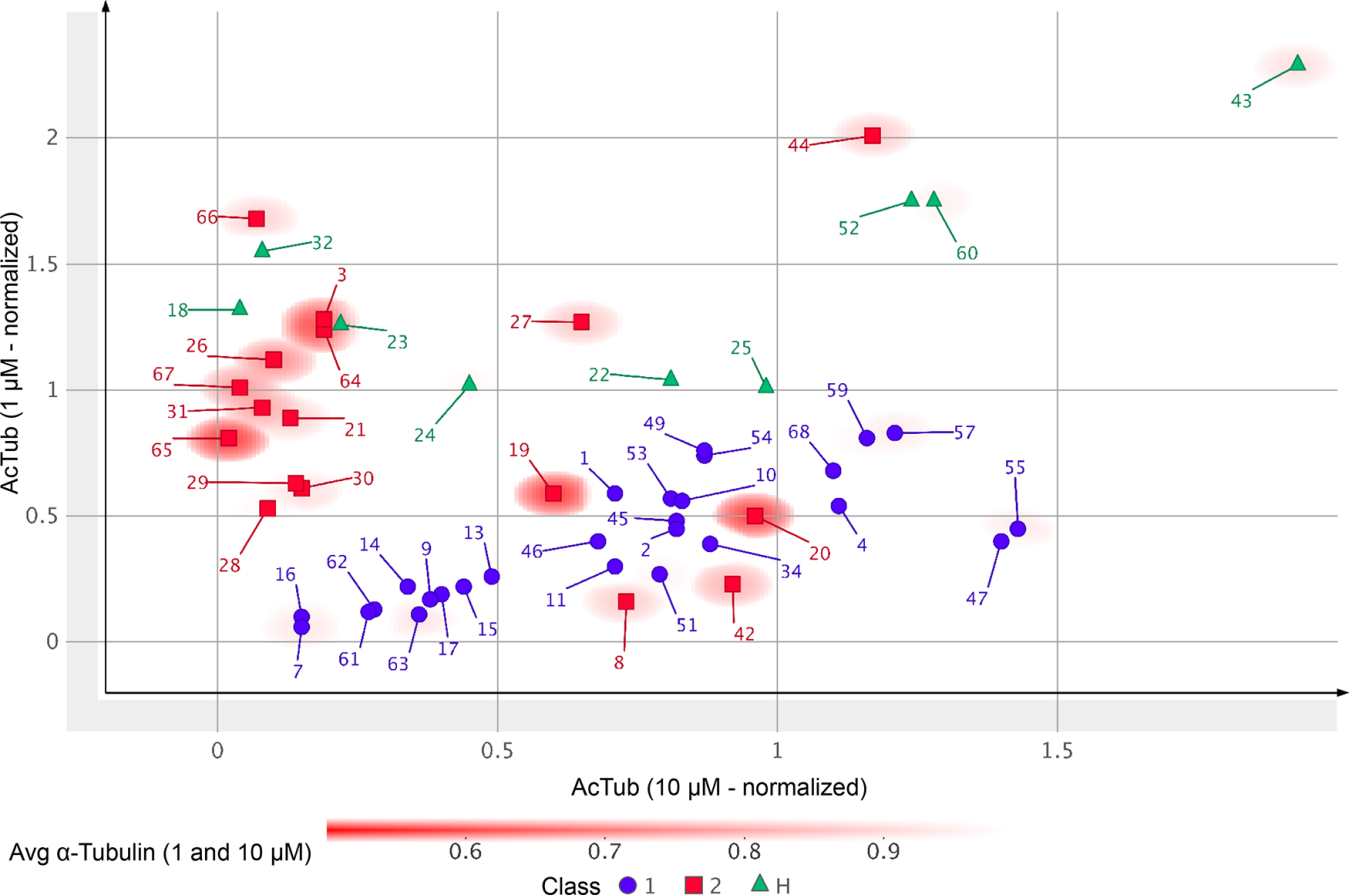
Plot showing the activity of test compounds normalized to positive control (*i.e.*, 100 nM of **5**) in the AcTub assay at 1 μM (*Y*-axis) and 10 μM (*X*-axis). Class I compounds are shown as blue circles; Class II compounds as red squares; and hybrid compounds as green triangles. The average loss of total α-Tub after compound treatment at 1 and 10 μM relative to vehicle-treated controls is plotted as background red color based on the heatmap shown. Note that **12** is not plotted as the activity of this compound was determined at 10 and 30 μM.

To further investigate the role of the side chain in the para position of the fluorinated ring, we evaluated a series of 16 alkyne derivatives. In particular, since prior SAR studies^12^ suggested that electron-withdrawing groups in *para* are generally preferred for Class I activity, we evaluated the effect of incorporating an alkyne linkage within the aliphatic side chain off of the para position of TPDs. Consistent with prior SAR data, terminal alkyne TPD derivative, **34**, was found to exhibit Class I activity. Furthermore, among the 15 disubstituted alkyne derivatives examined in this study (**42**–**47**, **49**, **51**–**55**, **57**, **59**, **60**), ten examples (**45**–**47**, **49**, **51**, **53**–**55**, **57**, **59**) were found to exhibit Class I activity, while two compounds (**42** and **44**) were deemed to be Class II, and three (**43**, **52**, and **60**) were hybrids. Thus, in the majority of cases, the replacement of the alkoxy linkage with a rigid and electron-withdrawing alkyne was found to result in relatively potent compounds that do not cause loss of total tubulin (Table 1 and Figure 3). The only derivatives found to be Class II within our set of alkyne derivatives (**42** and **44**) were those equipped with the same (*S*)-1,1,1-trifluoropropan-2-amine at C7 as in **3**, which again suggests that this particular amine fragment is likely to play a role in the tubulin degradation properties of Class II TPDs. However, even in these cases, the incorporation of the alkyne moiety resulted in derivatives that produced a more moderate reduction in tubulin levels compared to the corresponding alkoxy congeners (*cf.*, **3**–**44**, **64**–**42**, Table 1 and Figure 3). In the particular case of TPD alkyne derivative, **43**, which also features an (*S*)-1,1,1-trifluoropropan-2-amine at C7 like its alkoxy MMP compound (**19**), a reduction in total tubulin levels could be detected only at higher concentrations (*i.e.*, 30 μM, see Supporting Information, Figure S1B).

**Figure 4:**
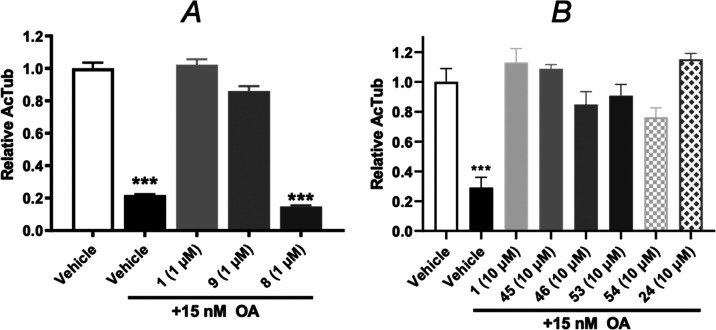
ELISA determination of AcTub levels in homogenates from primary rat cortical neurons treated with either vehicle, or OA (15 nM) in the presence of vehicle or TPD **1**, **8**, **9** (A); and primary mouse cortical neurons treated with vehicle, or OA (15 nM) in the presence of vehicle or TPD **1**, **24**, **45**, **46**, **53, 54** (B). Error bars represent SEM, with ***, *p* < 0.001 relative to vehicle treatment in the absence of OA as determined by one-way ANOVA.

To further evaluate the activity of Class I alkyne derivatives and hybrid compounds, several compounds were also assessed for their ability to compensate for MT deficits in primary neuron cultures that were treated with the phosphatase inhibitor, okadaic acid (OA). As previously described,^11^ treatment of rodent neurons with OA results in tau hyperphosphorylation and MT collapse that presumably results, at least in part, from tau disengagement from MTs. Co-treatment of neurons with Class I TPDs, such as **1**, along with OA prevents this MT collapse, with a resulting normalization of MT structure and neuronal processes which can be observed upon visual inspection of the neurons or through an assessment of neuronal AcTub levels, while Class II TPDs are generally unable to provide meaningful compensation of MT deficits, especially when tested at higher (*i.e.*, 10 μM) compound concentration.^11,12^ This phenomenon is illustrated in Figure 4A, in which Class I TPD, **1**, is evaluated for its ability to normalize AcTub levels in cortical neurons in the OA assay in comparison with TPD enantiomers **8** (*S*) and **9** (*R*) that exhibit, respectively, Class II and Class I phenotype in QBI293 cells (Table 1). Interestingly, evaluation of Class I alkyne derivatives **45**, **46**, **53**, and **54** in the OA assay confirmed that these compounds can prevent the OA-induced MT collapse in cortical neurons in a manner similar to **1** (Figure 4B). Notably, in the case of the hybrid compounds, evaluation in the OA assay revealed that some compounds (*e.g.*, **24** and **60**) provided a similar dose-dependent correction of MT deficits in OA-treated neurons as seen for Class I TPDs, while other compounds (*e.g*., **25**) caused a lesser recovery of OA-induced MT collapse (see Supporting Information, Figure S2).

**Figure 5:**
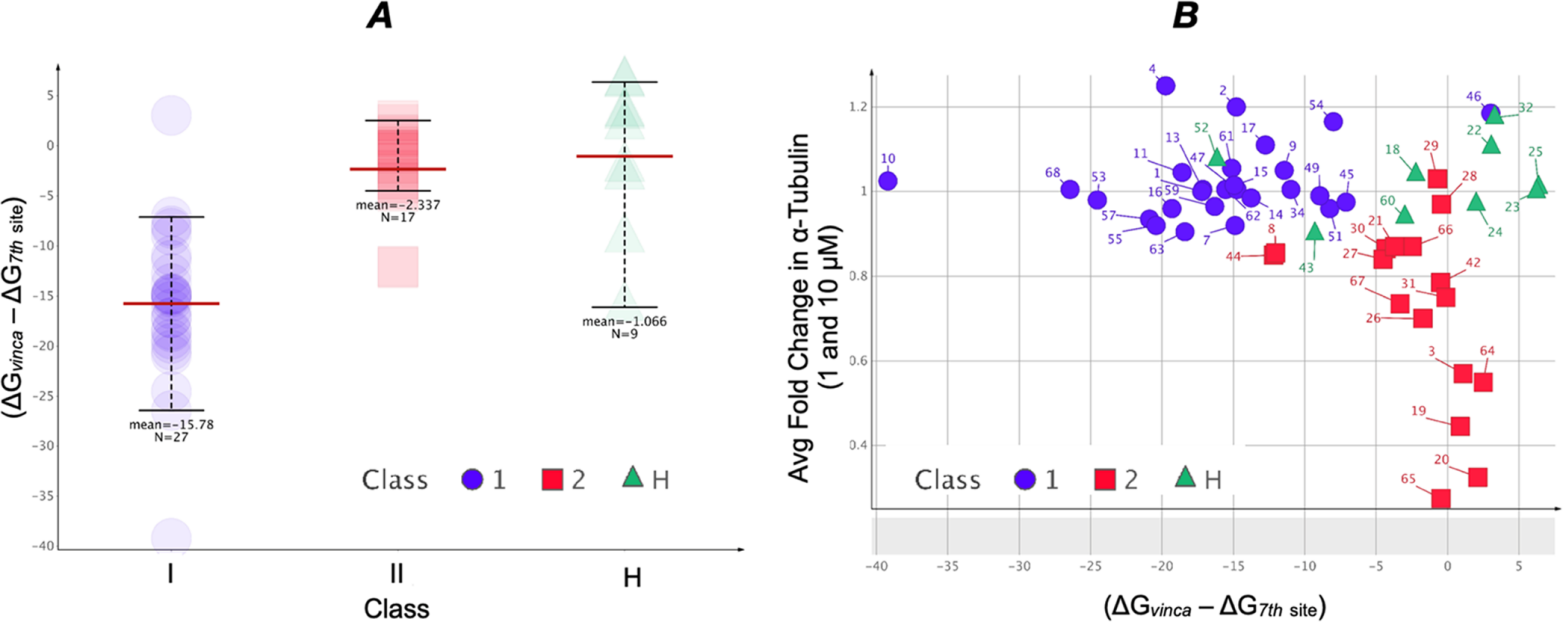
(A) Whisker plot showing the distribution of differential calculated binding energies (*i.e.*, Δ*G*_vinca_ – Δ*G*_7th site_) within each class of compounds. The more negative the differential binding energy, the greater the relative binding to the vinca site compared to the seventh site. Mean values are indicated as thick horizontal red lines. (B) Scatter plot showing the differential calculated binding energies for the two sites (*X*-axis) and the average fold-change in total α-tubulin levels caused by compound treatment at 10 and 1 μM in QBI293 cells [*i.e.*, (α-tub_10μM_ + α-tub_1μM_)/2] relative to vehicle-treated control cells (*Y*-axis). Although Class I compounds are largely characterized by a higher calculated affinity for the vinca than seventh site with little decrease in α-tubulin levels, most of the Class II and hybrid compounds group together with a smaller differential of calculated binding energies with Class II compounds also exhibiting an average fold-change in α-tubulin value that generally is <0.90.

Although the SAR data emerging from these studies highlight an apparently complex interplay between the fragments at C6 and C7, comparison of biological activity data for each of the listed 53 TPD congeners with the corresponding calculated binding energies for the vinca and the seventh sites revealed general trends. In particular, as shown in Figure 5, Class I compounds appear to be generally associated with a greater calculated affinity for the vinca site, whereas Class II and hybrid compounds exhibit a smaller differential of calculated binding energies for the two sites and are thus expected to show little or no selectivity for either one of the two sites. Moreover, a plot of the differential binding affinity for the vinca and seventh site compared to the level of tubulin degradation induced by the compounds reveals a distinct clustering of the Class I, II and hybrid TPDs (Figure 5B).

**Figure 6:**
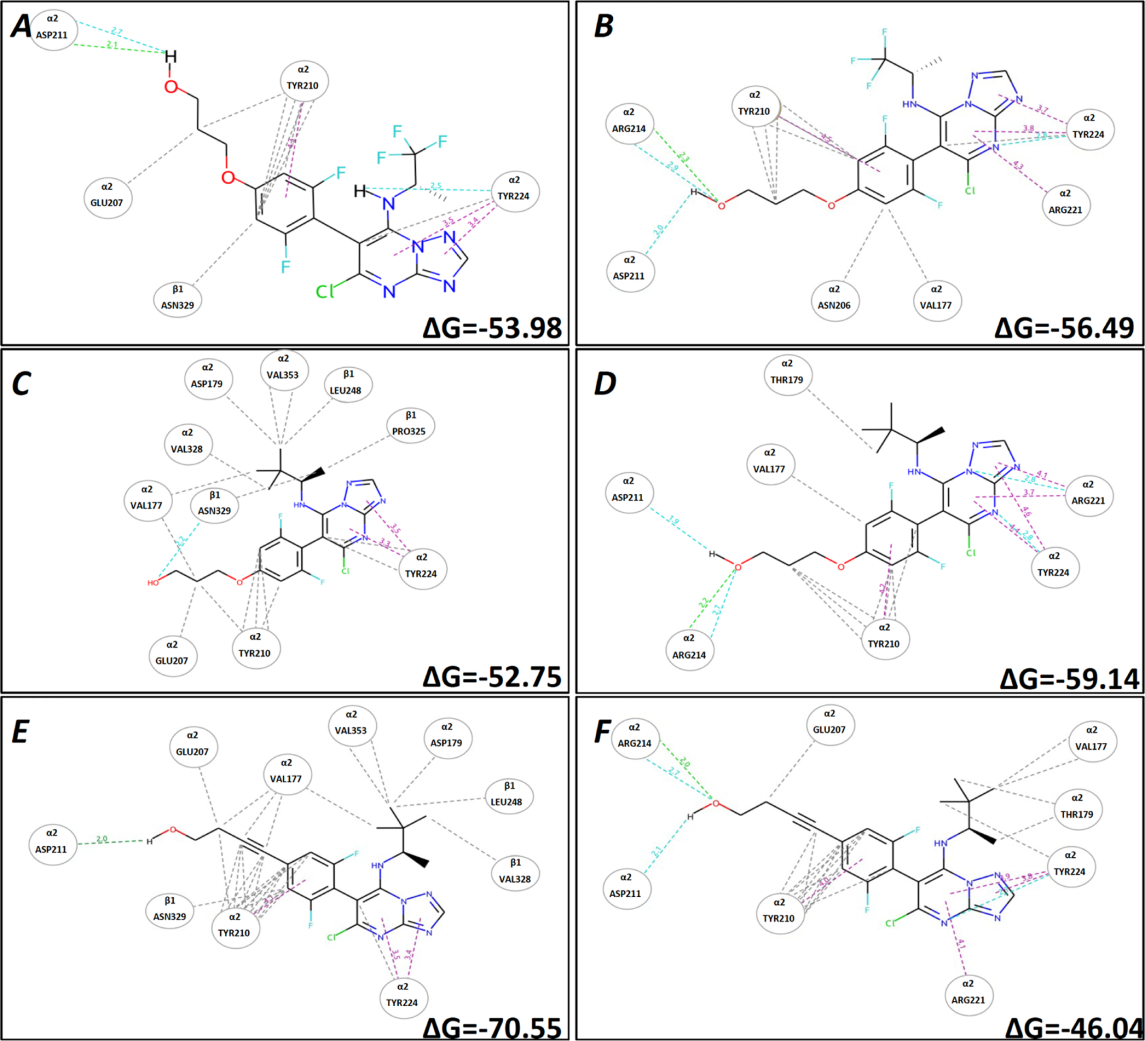
2D interaction plot of Class II compound **64** (A, B), hybrid compound **25** (C, D), and Class I compound **53** (E, F) in the vinca site (left, from PDB: 5NJH) and seventh site (right, from PDB: 7CLD), respectively. Turquoise dashed lines indicate weak H-bonds, green dashed lines indicate strong H-bonds, gray dashed lines indicate hydrophobic interactions, and purple dashed lines indicate electrostatic and π–π interactions.

Evaluation of the relative contribution of van der Waals, electrostatic, hydrogen bond, and π–π interactions showed that hydrophobic and electrostatic effects account for the majority of the Δ*G* difference observed between the two pockets (Supporting Information, Table S1), with Class I compounds forming more hydrophobic interactions in the vinca site compared to the Class II and hybrid compounds. For example, a two-dimensional (2D) interaction plot of a Class II compound (**64**), a hybrid compound (**25**), and a Class I compound (**53**) in both the vinca and seventh site (Figure 6) shows that the Class I congener is expected to establish stronger hydrophobic interactions with β-tubulin residues in the vinca binding site (*i.e.*, Val353, Val328, and Leu248). Moreover, docking studies clearly indicate that the side chain in the para position of the fluorinated phenyl ring at C6 plays a critical role in determining the relative binding affinity for the two pockets (*e.g.*, Figure 6C,E).

**Figure 7:**
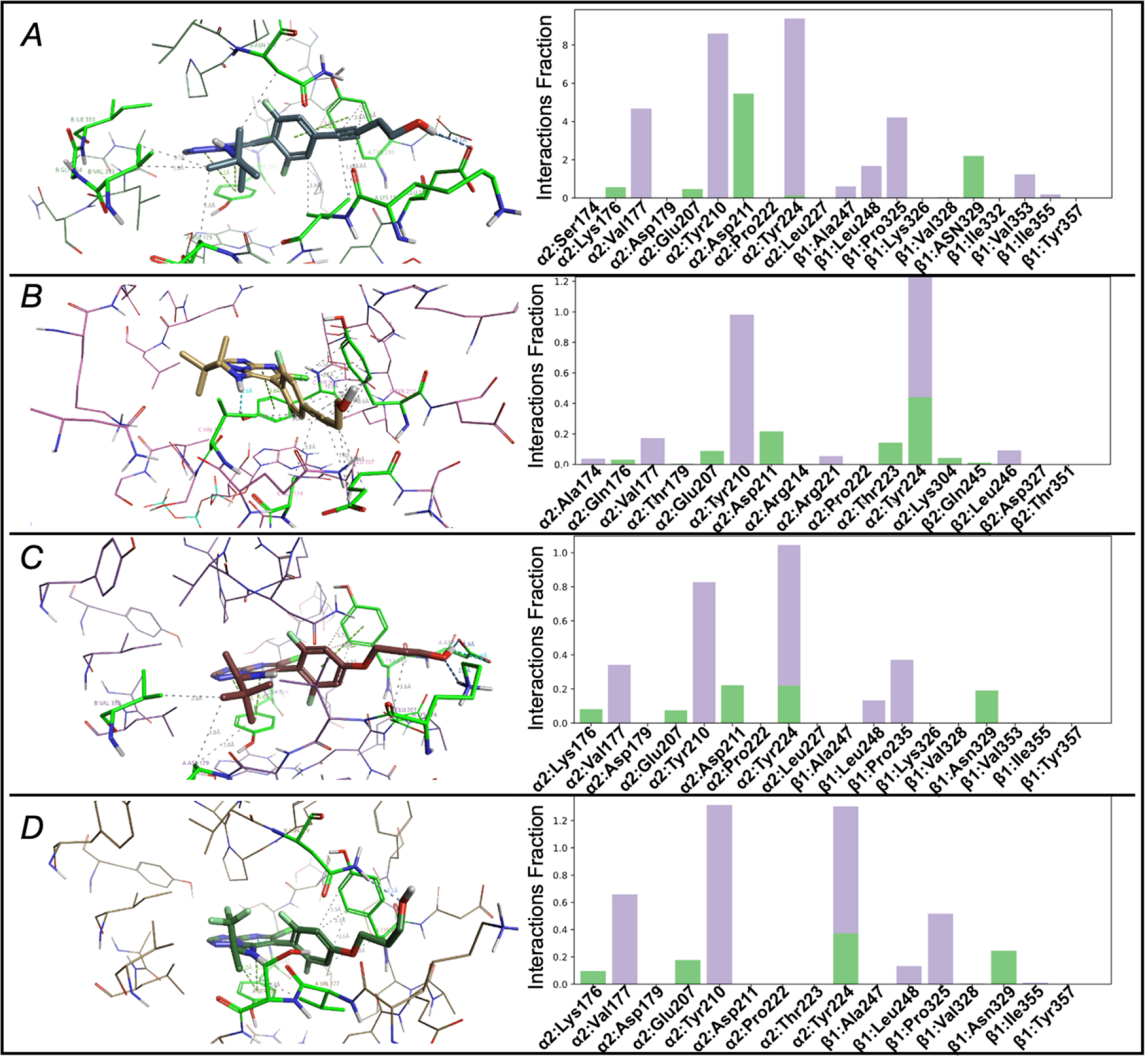
Binding mode for **53** in both pockets (A, vinca site, from PDB: 5NJH; B, seventh site, from PDB: 7CLD) and **25** and **64** in the vinca site (C and D, respectively) after MD simulation, and the corresponding protein interactions throughout the simulation after reaching stability. Protein–ligand interactions are categorized into two groups: hydrogen bond (green), and hydrophobic (purple-gray) interactions. The interaction fraction indicates the normalized percentage of the simulation time for which the specific interaction is maintained.

To further investigate the role of the side chains in the binding selectivity, a series of 100 ns molecular dynamic simulations (MD) were conducted using two MMPs (**25**–**53** and **64**–**42**) that produce differing effects on the cellular phenotype. All of the simulations showed an initial rearrangement of the ligand complex and a consecutive stabilization after 50 ns (Supporting Information, Figure S3). The dynamic results showed that all of the compounds could maintain a stable binding pose during the entire MD simulation in both pockets; however, the alkyne derivatives were found to adjust their orientation to establish interactions with the β-tubulin residues in the vinca site (Figure 7A vs B). Comparison of TPD derivatives **53** and **25** that feature the same amine fragment at C7 revealed that the alkyne **53** can establish multiple hydrophobic contacts in the vinca site throughout the MD simulation (*i.e.*, α-tubulin residue Val177, as well as β-tubulin residues Pro325 and Val353) and that the presence of the rigid alkyne linkage can also facilitate the H-bonding interaction between the terminal hydroxyl and the α-tubulin residue, Asp211, in the vinca site, improving the overall affinity for this binding region (Figure 7A vs C). In contrast, the replacement of the *t*-butyl group with the smaller CF_3_ in the amine fragment at C7, combined with the presence of a more flexible alkoxide side chain (**64**), resulted in a similar affinity for both pockets (Figures 6A,B and 7D).

**Figure 8:**
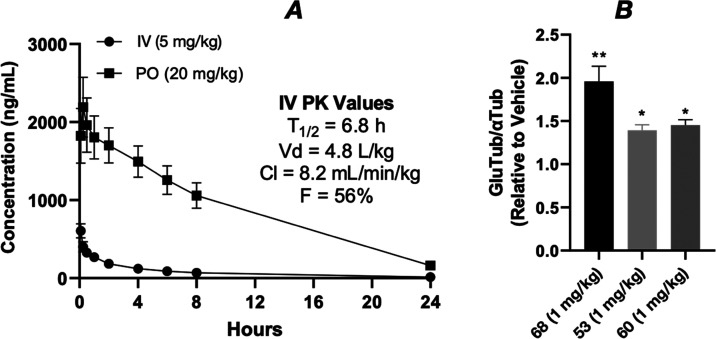
(A) Plasma PK of **60** after administration of 5 mg/kg (i.v. injection; black circles) or 20 mg/kg (p.o.; black squares). Error bars represent standard deviation (SD). (B) Increases in the GluTub-to-total α-Tub ratio in the brains of WT mice after i.p. dosing of **68**, **53**, and **60** at 1 mg/kg once daily for two consecutive days, relative to vehicle-treated mice. The data represent a summary of independent experiments with each compound in which *n* = 4 CD-1 mice received a test compound or vehicle. Error bars represent SEM, with *, *p* < 0.05 and **, *p* < 0.01 as determined by a two-tailed *t*-test.

To evaluate the potential of the newly identified Class I and hybrid alkyne TPD derivatives as potential candidates for tauopathy treatment, selected compounds (**53** and **60**) underwent PK analysis in mice. Determination of brain-to-plasma ratios (*B*/*P*) for these two compounds 1 h post-administration (i.p. injections of 1.5 mg/kg) indicated that both derivatives are brain penetrant with a *B*/*P* of approximately 0.5 (data not shown). Furthermore, a more comprehensive plasma PK study with compound **60** after i.v. and oral (p.o.) dosing revealed favorable properties (Figure 8A), including a relatively long half-life of >6 h and an oral bioavailability of >50% (see also Supporting Information, Figure S4, for plasma PK of **53**). Finally, to evaluate target engagement in the CNS, PD studies were conducted to determine compound-dependent elevations in the stable MT marker, de-tyrosinated α-tubulin (GluTub), in brain homogenates from compound-treated WT mice. As summarized in Figure 8B, **53** and **60**, as well as the previously identified Class I TPD **68**, produced significant elevations in brain GluTub when administered at the low dose of 1 mg/kg (i.p.) once daily for two consecutive days.

## Discussion

MT-normalizing TPDs hold promise as candidate therapeutics for the treatment of tauopathies and potentially other neurodegenerative diseases. However, this class of MT-active compounds presents an apparently complex SAR that arises from the fact that TPDs can interact with varying affinities with at least two spatially distinct binding sites, the vinca site and the seventh site, producing substantially different cellular phenotypes. The availability of the co-crystal structures of **4**(10) and **5**(13) with assembled tubulin, combined with our findings from prior SAR studies,^12^ provided the basis for further exploration of SAR of the fragments at C6 and C7 of the TPD core and the possible link between the calculated relative affinity for the two binding sites and the cellular phenotype associated with different TPD congeners.

**Figure 9:**
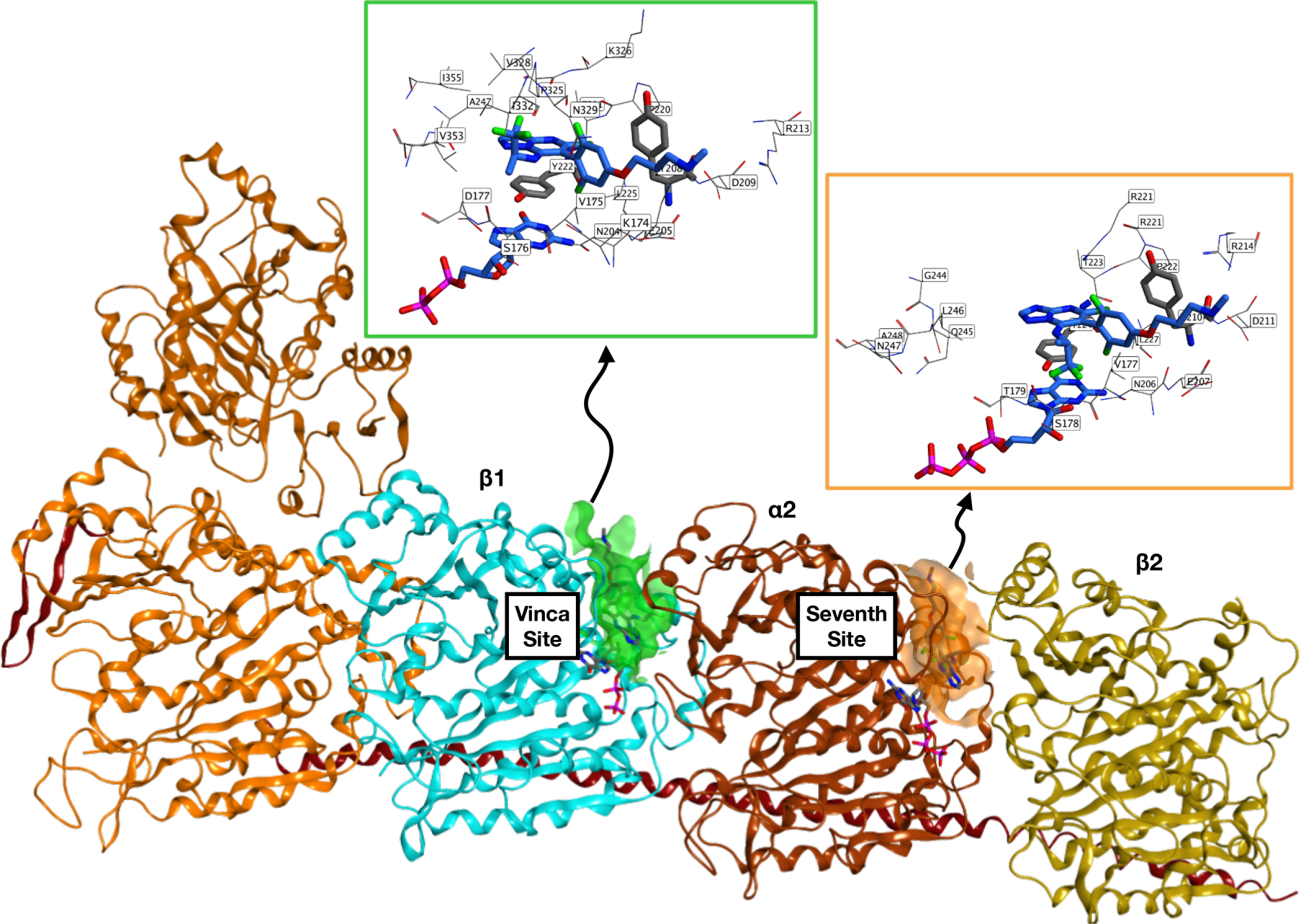
Overview of the vinca binding site between β1 and α2-tubulin, and the sevenths site between α2 and β2-tubulin (from co-crystal structure with compound **5**; PDB: 7CLD(13)).

Structural analysis of the two binding sites revealed a high similarity between the two pockets (Figure 9). However, the vinca site, located at the interface between β1 and α2 tubulin, exhibits different spatial arrangements of several amino acid residues relative to the seventh site, which is located at the α2-β2 interface. These amino acid residues include the β1 residues Val324, Pro325, Lys326, Val328, Asn329, Ala330, Ile332, Val353, and Ile355 that may potentially play a role in the binding of TPDs.

Prior SAR studies already highlighted that with few exceptions, compounds of general structure ***A*** (Scheme 1) in which the aliphatic amine fragment is a relatively bulky and lipophilic exhibit Class I activity. However, an assessment of the same amine fragments in the context of TPDs of general structure ***B*** (Scheme 1) was not conducted. Thus, one of the objectives of the present study was to evaluate/compare more systematically the effect of different amine fragments at C7 in the context of compounds of general structure ***A*** and ***B***. Moreover, considering that our prior SAR studies indicated that the presence of an electron-withdrawing group in the para position of the fluorinated ring at C6 (*i.e.*, substituents characterized by a positive Hammett σ_p_ value) is generally conducive to Class I activity, we expanded the exploration of TPDs to a series of novel congeners that are characterized by the presence of a side chain connected to the para position via an intervening alkyne moiety (general structure ***C***, Scheme 3).

Comparison of biological activity in the QBI293 assay of a series of MMPs of general structure ***A***, ***B***, and ***C*** illustrates an apparently complex interplay between the nature of the amine fragment at C7 and the substituent in the para position of the aromatic ring at C6. Furthermore, in addition to the previously described Class I and II phenotypes, these studies led to the identification of hybrid TPD congeners that do not cause reduction in total tubulin levels yet show an unusual dose–response relationship in the QBI293 AcTub assay. Interestingly, the biological results within our set of compounds correlate generally well with calculated binding energies for both the vinca site and the seventh site (Table 1 and Figures 3 and 5). Out of 27 Class I TPDs examined in this study, 25 examples exhibit a Δ*G* of binding for the vinca site that is at least −60 kcal/mol or lower, as well as a differential of binding energies for the two sites (*i.e.*, Δ*G*_vinca_ – Δ*G*_seventh_) that is at least −5 kcal/mol or lower. However, in the case of Class II and hybrid compounds, the calculated binding affinities for the two sites indicate a smaller differential of binding affinity, with 15/18 Class II compounds and 7/8 hybrid compounds exhibiting a differential of binding energies for the two sites that is above −5 kcal/mol. These observations are consistent with the notion that the MT-stabilizing activity of TPDs is mediated by interactions at the vinca binding site on β-tubulin and suggest that relatively simple estimations of the binding energies of TPDs in the two sites may be used to predict whether TPDs will exhibit Class I activity. Moreover, the fact that no major differences in the calculated relative binding affinities for the two binding sites were observed when comparing Class II and hybrid compounds may suggest that the hybrid TPDs interact, like Class II compounds, both at the vinca and the seventh site but possibly via different binding modes. For example, prior studies^13^ found that the degradation of cellular tubulin observed after addition of Class II compound, **5**, may be linked to the ability of this compound to promote the dissociation of the nonexchangeable GTP in the seventh site. Therefore, it is possible that relatively small differences in binding modes of Class II and hybrid TPDs in the seventh site may result in differing abilities of these compounds to trigger, directly or indirectly, GTP release and subsequent tubulin degradation. In this context, although the AcTub and total α-Tub assays in QBI293 cells provide a generally effective method to sort compounds between different groups based on the magnitude and characteristics of the cellular response, our studies show that further assessment/confirmation of compound activity in the neuronal OA assay with MT deficits is desirable when prioritizing/selecting candidate compounds for further development.

**Figure 10:**
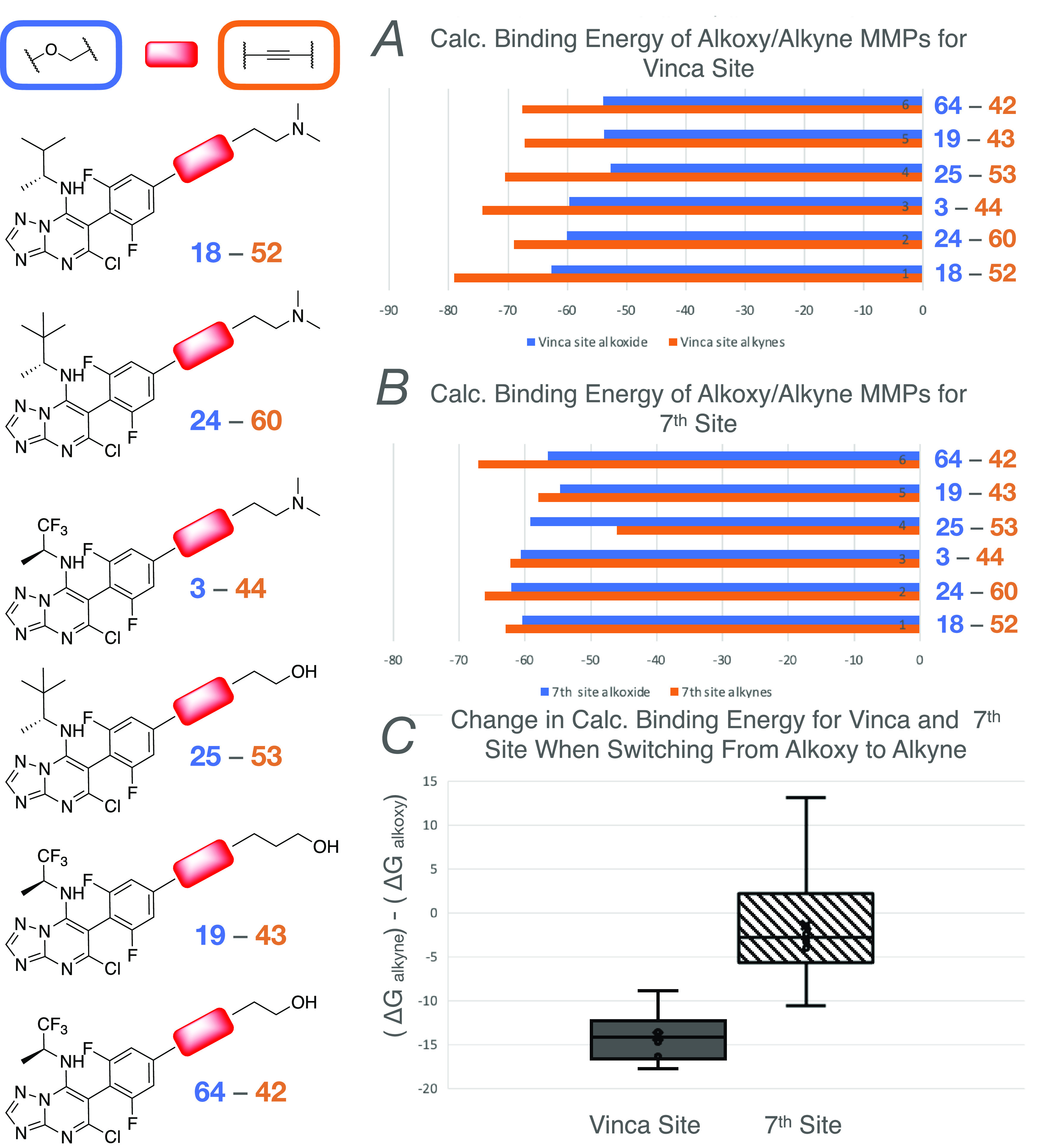
Structure of six pairs of compounds differing only by the nature of the linkage in the para position (left). (A) Comparison of calculated binding energy for the vinca site of the alkoxy (blue) and alkyne (orange) derivatives within each MMP. (B) Comparison of calculated binding energy for the seventh site of the alkoxy (blue) and alkyne (orange) derivatives within each MMP. (C) Average change in calculated binding energy for the vinca and the seventh site when switching from alkoxy to alkyne linkage in the six MMPs (average values are indicated with a cross and median values with a solid line).

With respect to the alkyne derivatives of general structure ***C***, a comparison with the corresponding derivatives of general structure ***B*** that bear an alkoxide side chain (Figure 10) indicates that, in general, the presence of the alkyne linkage results in a relative increase in binding affinity for the vinca site (Figure 10A) and enlargement of the differential of binding affinity between the vinca and the seventh site. This effect is likely due at least in part to the rigidification of the side chain provided by the presence of the alkyne moiety, which ultimately promotes interactions within the vinca site (Figures 6 and 7). Eleven of the 16 alkyne derivatives synthesized and tested were found to exhibit Class I activity. Moreover, even in those cases in which incorporation of the alkyne linkage was not sufficient to impart Class I activity (*e.g.*, see Class II derivatives **42** and **44**, and hybrid derivatives **43**, **52**, and **60**), the presence of the alkyne moiety in these molecules generally led to a notable attenuation of the Class II (*cf*., **3**–**44**; **19**–**43**; and **64**–**42**) or hybrid (*cf.*, **24**–**60**; **18**–**52**) character, as evidenced by a reduced loss of total tubulin and/or a reduced tendency to show a bell-shaped dose–response curve in the AcTub assay compared to the corresponding alkoxy side-chain derivatives (Table 1 and Figure 3).

**Figure 11:**
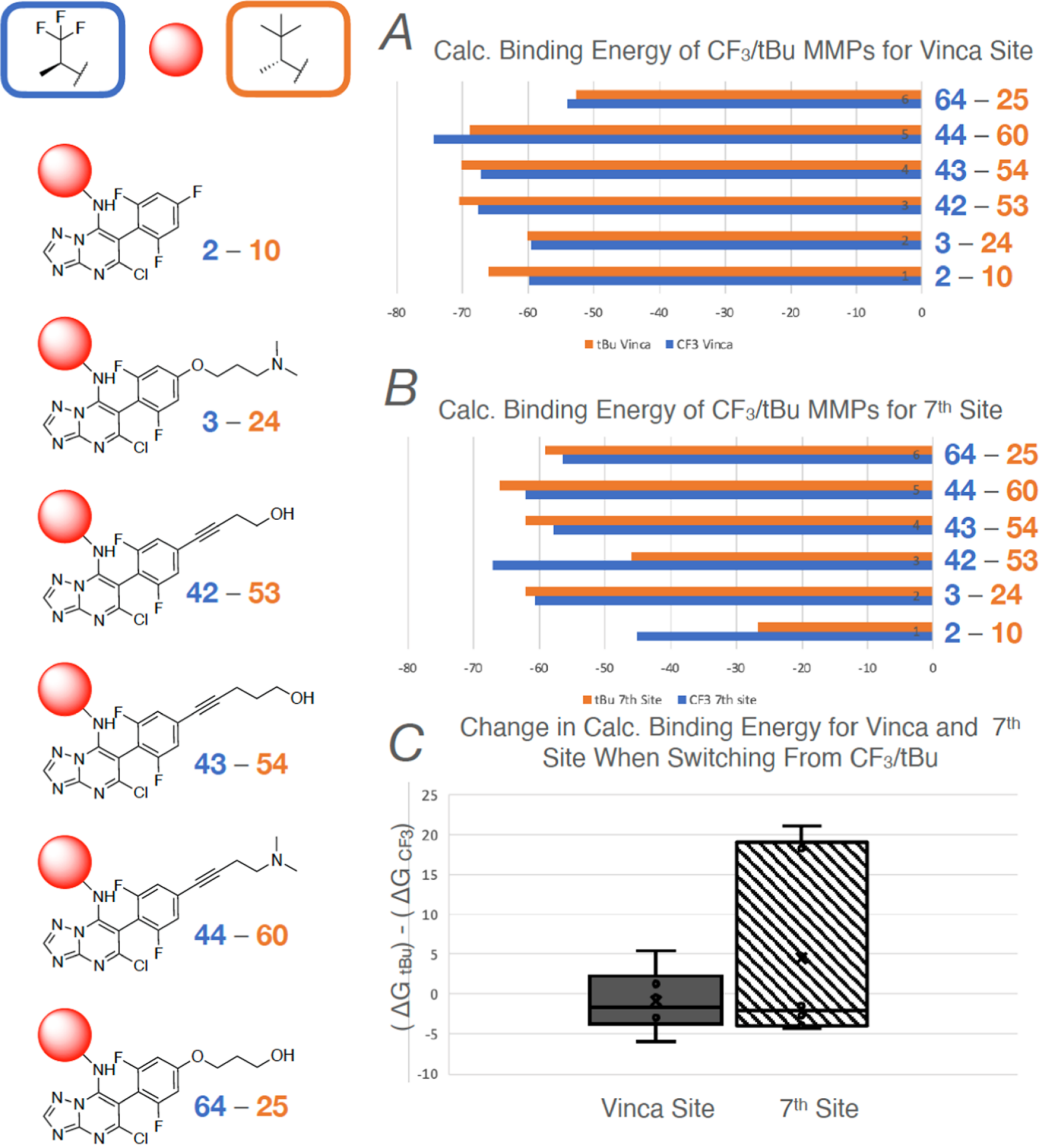
The structure of six pairs of compounds differing only by the nature of the amine fragment at C7 (left). (A) Comparison of calculated binding energy for the vinca site of MMPs bearing either the (*S*)-1,1,1-trifluoropropan-2-amine (blue) or the (*R*)-3,3-dimethylbutan-2-amine (orange) at C7. (B) Comparison of calculated binding energy for the seventh site of MMPs bearing either the (*S*)-1,1,1-trifluoropropan-2-amine (blue) or the (*R*)-3,3-dimethylbutan-2-amine (orange) at C7. (C) Average change in calculated binding energy for the vinca and the seventh site when switching from (*S*)-1,1,1-trifluoropropan-2-amine to (*R*)-3,3-dimethylbutan-2-amine at C7 (average values are indicated with a cross and mean values with a solid line).

With respect to the amine fragment at C7, comparison of MMPs indicates that these fragments generally do not significantly alter the calculated binding energy for the vinca site. However, depending on the nature of the fragment at C6, the relatively bulkier and more lipophilic amines can reduce the binding affinity for the seventh site. For example, direct comparison of six MMPs bearing either the (*S*)-1,1,1-trifluoropropan-2-amine or the (*R*)-3,3-dimethylbutan-2-amine (Figure 11) reveals that in all cases, the change in calculated binding energy for the vinca site between the two series of compounds is relatively small (Figure 11A,C). However, when examining the calculated binding energies for the seventh site, in selected cases (*cf*., **2**–**10** and **42**–**53**) the presence of the bulkier and more lipophilic (*R*)-3,3-dimethylbutan-2-amine results in a drastic lowering of the binding affinity for this site and a consequent increase in the differential of binding energy between the vinca and seventh sites.

Importantly, further evaluation of representative Class I and hybrid alkyne derivatives (respectively, **53** and **60**) in mice revealed that both compounds are brain penetrant (*i.e.*, *B*/*P* ∼ 0.5). Moreover, plasma PK studies of **53** and **60** demonstrated a very favorable PK profile, including relatively prolonged half-life and, in the case of **60**, good oral bioavailability (Figure 8A). Equally important, PD studies with **53**, **60**, and the previously described Class I TPD, **68**,^12^ showed that these alkyne derivatives can produce a significant elevation in markers of stable MTs (*i.e.*, GluTub) in the brain of WT mice at a low dose (Figure 8B). Thus, these results indicate that these novel acetylene derivatives may be considered promising alternatives to **68** as candidate therapeutics for neurodegenerative tauopathies and possibly other neurodegenerative indications.

## Conclusions

Previous studies have shown that, depending on the substitution pattern, MT-targeting TPDs can interact with either one or two spatially distinct binding regions within tubulin heterodimers (*i.e.*, the seventh site and the vinca site) producing different effects on tubulin and MT function. Our structure–activity relationship studies, based on matched molecular pair analyses, provide additional insight into the complex interplay between the fragments at C6 and C7 in determining the biological activity and the relative affinity of TPDs for the two binding sites. Calculated binding energies of TPDs for the vinca and the seventh site were found to largely correlate with biological data and indicate that compounds with preferential affinity for the vinca site exhibit dose-dependent MT-stabilizing activity without causing reduction in total tubulin levels (*i.e.*, Class I activity). The Class II TPDs that have an atypical dose–response profile in assays of MT stabilization and trigger tubulin degradation interact with both binding sites with similar affinity, and our studies have now identified an additional hybrid series of congeners that bind to both sites with comparable affinity but do not decrease total tubulin levels. However, like the Class II compounds, these hybrid molecules are characterized by a nonlinear dose–response relationship in the QBI293 AcTub assay. Finally, our findings that the incorporation of an acetylene linkage in the aliphatic side chain at the para position of the fluorinated ring at C6 can provide potent Class I MT-stabilizing derivatives that exhibit excellent PK/PD profiles allows for a significant expansion of the possible structures that may be considered as potential candidates for CNS-directed MT-stabilizing therapies. In this context, the acetylene derivatives, **53** and **60**, identified in these studies represent promising alternatives to **68** as candidate therapeutics for neurodegenerative tauopathies.

## Experimental Section

### Materials and Methods

All solvents were of reagent grade. All reagents were purchased from Aldrich or Acros Organics and used as received. Thin-layer chromatography (TLC) was performed with 0.25 mm E. Merck precoated silica gel plates. Silica gel column chromatography was performed with silica gel 60 (particle size 0.040–0.062 mm) supplied by Silicycle and Sorbent Technologies. TLC spots were detected by viewing under a UV light. Proton (^1^H) and carbon (^13^C) NMR spectra were recorded on a 500 MHz Bruker AMX-500 spectrometer and on a 600 MHz Bruker AVANCE III. Fluorine (^19^F) NMR spectra were recorded on a 400 MHz Varian Mercury Plus spectrometer using trifluorotoluene as an internal standard. Chemical shifts were reported relative to solvents. Data for ^1^H, ^13^C, and ^19^F NMR spectra are reported as follows: chemical shift [ppm, referenced to protium; s = singlet, d = doublet, t = triplet, q = quartet, quint = quintet, dd = doublet of doublets, td = triplet of doublets, ddd = doublet of doublet of doublets, bs = broad singlet, m = multiplet, coupling constant (Hz), and integration]. Not all ^13^C NMR signals were detected for compounds **42**–**49**, **51**, **52**, **54**, **57**, and **58** despite multiple attempts with exhaustive signal averaging. Some signals are expected to be difficult to detect due to ^19^F coupling reducing their intensity and the longer relaxation times found in a system where an aromatic ring is conjugated with an alkyne. ^13^C NMR spectra of compounds **44**, **50**, **55**, **56**, and **59** were not featured for the same reasons of low signal/noise ratio. High-resolution mass spectra (HRMS) were measured using an Agilent 6230 time-of-flight mass spectrometer with a Jet stream electrospray ionization source. Single-crystal X-ray structure determinations were performed Bruker MicroStar with an APEX II detector, double-bounce micro-focus optics, and a Cu rotating anode source. Analytical reversed-phase (Sunfire C_18_; 4.6 mm × 50 mm, 5 mL) high-performance liquid chromatography (HPLC) was performed with a Gilson HPLC equipped with UV and a mass detector. All samples were analyzed employing a linear gradient from 10 to 90% of MeCN in H_2_O over 6 min and a flow rate of 2 mL/min. Preparative reversed-phase HPLC purifications were performed on a Gilson instrument employing Waters SunFire preparative C_18_ OBD columns (5 μm 19 mm × 50 mm or 19 mm × 100 mm). Purifications were carried out employing a linear gradient from 10 to 90% of MeCN in water using formic acid or trifluoroacetic acid as a 0.1% modifier. All final compounds were found to be >95% pure by HPLC analysis.

#### General Procedure A (Addition of the Amine)

According to a reported procedure,^14^ to 5,7-dichloro-6-(2,4,6-trifluorophenyl)-[1,2,4]triazolo[1,5-*a*]pyrimidine (1.0 equiv) in *N*,*N*-dimethylformamide (DMF) (0.1 M) at rt was added the appropriate amine or amine hydrochloride (1.5 to 3.0 equiv) followed by Et_3_N (3.0 equiv) if necessary. The reaction mixture was stirred for 0.5–2 h at rt and diluted with H_2_O. The aqueous phase was extracted with EtOAc (×3), and the combined organic layers were washed with brine (×2), dried (Na_2_SO_4_), filtered, and concentrated. The products were purified by flash chromatography.

#### General Procedure B (Addition of the Alkoxy Side Chain)

According to a reported procedure,^14^ to a suspension of NaH (4.0 equiv) in DMSO (0.35 M) was added the appropriate amino alcohol (4.0 equiv), and the mixture was heated to 60 °C for 1 h. The resulting solution was treated with a solution of trifluoroarene (1.0 equiv) in DMSO (0.5 M). The reaction mixture was stirred at 60 °C for 3 h and monitored by liquid chromatography–mass spectrometry (LCMS). Following complete consumption of the starting material, the reaction mixture was cooled to rt and diluted with H_2_O and EtOAc. The aqueous layer was extracted with EtOAc (×2). The combined organic layers were washed with brine (×2), dried (Na_2_SO_4_), filtered, and concentrated. Purification by reversed-phase HPLC yielded the desired product.

#### General Procedure C (Sonogashira Coupling)

To a solution of the appropriate Iodo-TPD (1 equiv) in degassed DMF (0.2 M) were added successively CuI (0.15 equiv), Et_3_N (3 equiv), and the desired alkyne (3 equiv). The mixture was degassed and backfilled with nitrogen before Tetrakis palladium (0.1 equiv) was added. The mixture was degassed and backfilled with nitrogen three times and then stirred at rt for 8 h before it was diluted with water. The aqueous layer was extracted with EtOAc (×2). The combined organic fractions were washed with brine, dried, and concentrated under reduced pressure. Finally, purification via silica gel flash chromatography (Hexanes/EtOAc) or by reversed-phase HPLC (water/MeCN + 0.1% FA or trifluoroacetic acid (TFA), (10 to 90%), 20 mL/min, 20 min ramp time) yielded the desired compound.

#### General Procedure D (Boc Deprotection)

To a solution of the appropriate Boc-protected alkynylated TPD (1 equiv) in MeOH (0.1 M) was added a 4 M solution of HCl in 1,4-dioxane (18 equiv). After 3 h at rt, the mixture was evaporated under reduced pressure to furnish the title compound as a HCl salt as a yellow or brown solid.

##### 5-Chloro-*N*-((1-methylcyclopropyl)methyl)-6-(2,4,6-trifluorophenyl)-[1,2,4]triazolo[1,5-*a*]pyrimidin-7-amine (**13**)

General procedure A was followed using dichloride **6** (50 mg, 0.16 mmol), (1-methylcyclopropyl)methanamine hydrochloride (29 mg, 0.24 mmol), and Et_3_N (66 μL, 0.47 mmol), and the mixture was stirred for 1 h at rt. Purification via silica gel flash chromatography (Hexanes/EtOAc: 100/0 to 80/20) provided compound **13** as a white powder (46 mg, 0.13 mmol, 80%). ^1^H NMR (600 MHz, CDCl_3_) δ 8.35 (s, 1H), 6.84 (dt, *J* = 6.0, 6.0 Hz, 2H), 6.43 (bs, 1H), 2.81 (d, *J* = 4.5 Hz, 2H), 1.09 (s, 3H), 0.44–0.42 (m, 2H), 0.40–0.38 (m, 2H) ppm. ^13^C NMR (151 MHz, CDCl_3_) δ 164.09 (dt, *J* = 253.7, 15.1 Hz), 161.75 (dd, *J* = 250.7, 7.5 Hz), 161.67 (dd, *J* = 250.7, 7.5 Hz), 158.12, 155.12, 153.65, 146.47, 107.33 (dt, *J* = 19.6, 4.5 Hz), 101.09–100.76 (m), 88.93, 52.68, 20.99, 16.20, 11.93 ppm. IR (film) ν 1620, 1606, 1596, 1566, 1555, 1459, 1435, 1371, 1260, 1250, 1234, 1139, 1120, 1035, 1022, 997, 836, 763 cm^–1^. HRMS (ES^+^) calcd for C_16_H_14_ClF_6_N_5_ [M + H]^+^, 368.0884; found 368.0881.

##### 5-Chloro-*N*-((1-(trifluoromethyl)cyclopropyl)methyl)-6-(2,4,6-trifluorophenyl)-[1,2,4]triazolo[1,5-*a*]pyrimidin-7-amine (**14**)

General A procedure was followed using dichloride **6** (50 mg, 0.16 mmol), (1-(trifluoromethyl)cyclopropyl)methanamine hydrochloride (41 mg, 0.24 mmol), and Et_3_N (66 μL, 0.47 mmol), and the mixture was stirred for 1 h at rt. Purification via silica gel flash chromatography (Hexanes/EtOAc: 100/0 to 80/20) provided compound **14** as a white powder (64 mg, 0.15 mmol, 97%). ^1^H NMR (600 MHz, CDCl_3_) δ 8.35 (s, 1H), 6.89–6.86 (m, 2H), 6.32 (bs, 1H), 3.51 (s, 2H), 1.11 (d, *J* = 6.0 Hz, 2H), 0.74 (d, *J* = 6.0 Hz, 2H) ppm. ^13^C NMR (151 MHz, CDCl_3_) δ 164.33 (dt, *J* = 255.2, 15.1 Hz), 161.71 (dd, *J* = 252.4, 9.0 Hz), 161.56 (dd, *J* = 251.4, 9.0 Hz), 157.89, 155.28, 154.07, 146.32, 126.48 (q, *J* = 274.8 Hz), 101.42–101.05 (m), 89.84, 45.60, 23.56 (q, *J* = 33.2 Hz), 8.54 ppm. IR (film) ν 1624, 1613, 1571, 1560, 1469, 1440, 1383, 1359, 1246, 1146, 1121, 1056, 1034, 997, 909, 844, 766 cm^–1^. HRMS (ES^+^) calculated for C_16_H_10_ClF_6_N_5_Na [M + Na]^+^, 444.0421; found 444.0419.

##### 5-Chloro-*N*-(cyclobutylmethyl)-6-(2,4,6-trifluorophenyl)-[1,2,4]triazolo[1,5-*a*]pyrimidin-7-amine (**15**)

General procedure A was followed using dichloride **6** (50 mg, 0.16 mmol), cyclobutylmethanamine hydrochloride (29 mg, 0.24 mmol), and Et_3_N (66 μL, 0.47 mmol), and the mixture was stirred for 1 h at rt. Purification via silica gel flash chromatography (Hexanes/EtOAc: 100/0 to 80/20) provided compound **15** as a white powder (39 mg, 0.11 mmol, 68%). ^1^H NMR (600 MHz, CDCl_3_) δ 8.33 (s, 1H), 6.85 (dd, *J* = 6.0, 6.0 Hz, 2H), 6.31 (bs, 1H), 3.00 (t, *J* = 6.0 Hz, 2H), 2.47 (hept, *J* = 6.0 Hz, 1H), 2.90–2.03 (m, 2H), 1.96–1.89 (m, 1H), 1.86–1.80 (m, 1H), 1.62–1.55 (m, 2H) ppm. ^13^C NMR (151 MHz, CDCl_3_) δ 164.06 (dt, *J* = 253.7, 15.1 Hz), 161.82 (dd, *J* = 250.7, 9.1 Hz), 161.72 (dd, *J* = 250.7, 9.1 Hz), 158.06, 155.04, 153.68, 146.62, 107.28 (dt, *J* = 21.1, 6.0 Hz), 101.03–100.66 (m), 89.00, 48.75, 34.91, 25.44, 18.21 ppm. IR (film) ν 1579, 1571, 1562, 1545, 1492, 1478, 1335, 1252, 1204, 1168, 1121, 1034, 999, 844, 764, 513 cm^–1^. HRMS (ES^+^) calcd for C_16_H_14_ClF_3_N_5_ [M + H]^+^, 368.0884; found 368.0886.

##### (*S*)-5-Chloro-*N*-(1-cyclopropylethyl)-6-(2,4,6-trifluorophenyl)-[1,2,4]triazolo[1,5-*a*]pyrimidin-7-amine (**16**)

General procedure A was followed using dichloride **6** (150 mg, 0.47 mmol) and (*S*)-1-cyclopropylethan-1-amine (105 μL, 0.99 mmol, 2.1 equiv), and the mixture was stirred for 1 h at rt. Purification via silica gel flash chromatography (Hexanes/EtOAc: 100/0 to 60/40) provided compound **16** as a white powder (136 mg, 0.37 mmol, 79%). ^1^H NMR (600 MHz, CDCl_3_) δ 8.29 (s, 1H), 7.11–6.65 (m, 2H), 6.40 (d, *J* = 9.2 Hz, 1H), 2.98–2.89 (m, 1H), 1.11 (d, *J* = 6.6 Hz, 3H), 0.89 (dq, *J* = 11.2, 4.0 Hz, 1H), 0.50 (ddt, *J* = 22.1, 10.1, 4.4 Hz, 2H), 0.16 (dq, *J* = 10.3, 5.1 Hz, 1H), 0.03 (dq, *J* = 9.5, 4.9 Hz, 1H) ppm. ^13^C NMR (151 MHz, CDCl_3_) δ 164.08 (dt, *J* = 30.9, 16.2 Hz), 162.84–160.35 (m), 157.91, 155.06, 154.81, 153.88, 145.89, 107.23 (t, *J* = 21.4 Hz), 101.81–100.32 (m), 88.90, 54.32, 20.92, 17.66, 3.27 ppm. HRMS (ES^+^) calcd for C_16_H_14_ClF_3_N_5_ [M + H]^+^, 368.0884; found 368.0882.

##### (*R*)-5-Chloro-*N*-(1-cyclopropylethyl)-6-(2,4,6-trifluorophenyl)-[1,2,4]triazolo[1,5-*a*]pyrimidin-7-amine (**17**)

General procedure A was followed using dichloride **6** (100 mg, 0.31 mmol) and (*R*)-1-cyclopropylethan-1-amine (67 μL, 0.63 mmol, 2.1 equiv), and the mixture was stirred for 1 h at rt. Purification via silica gel flash chromatography (Hexanes/EtOAc: 100/0 to 60/40) provided compound **17** as a white powder (82 mg, 0.22 mmol, 71%). ^1^H NMR (600 MHz, CDCl_3_) δ 8.30 (s, 1H), 6.86–6.77 (m, 2H), 6.39 (d, *J* = 9.1 Hz, 1H), 2.94 (q, *J* = 7.1 Hz, 1H), 1.11 (d, *J* = 6.5 Hz, 3H), 0.94–0.82 (m, 1H), 0.56–0.44 (m, 2H), 0.17 (dq, *J* = 10.3, 5.1 Hz, 1H), 0.03 (dq, *J* = 9.5, 4.9 Hz, 1H). ^13^C NMR (151 MHz, CDCl_3_) δ 164.13 (dt, *J* = 30.3, 15.0 Hz), 162.85–160.25 (m), 157.95, 154.98, 153.93, 145.93, 107.36 (dd, *J* = 20.7, 4.5 Hz), 101.78–100.28 (m), 88.94, 54.44, 20.97, 17.73, 3.39 ppm. HRMS (ES^+^) calcd for C_16_H_14_ClF_3_N_5_ [M + H]^+^, 368.0884; found 368.0883.

##### (*R*)-3-(4-(5-Chloro-7-((3-methylbutan-2-yl)amino)-[1,2,4]triazolo[1,5-*a*]pyrimidin-6-yl)-3,5-difluorophenoxy)-*N*,*N*-dimethylpropan-1-aminium Formate (**18**)

General procedure B was followed using 3-(dimethylamino)propan-1-ol (89 μL, 0.76 mmol), NaH (60% in oil, 30 mg, 0.76 mmol), and **1** (70 mg, 0.19 mmol). Purification via preparative reversed-phase chromatography provided the title compound as a white solid (15 mg, 16% yield). ^1^H NMR (600 MHz, CDCl_3_) δ 8.43 (s, 1H), 8.31 (s, 1H), 6.59 (d, *J* = 9.1 Hz, 2H), 6.31 (s, 1H), 4.11 (t, *J* = 5.2 Hz, 2H), 3.29 (s, 1H), 3.06–3.01 (m, 2H), 2.66 (s, 6H), 2.24 (dt, *J* = 12.3, 6.0 Hz, 2H), 1.64 (dq, *J* = 13.1, 6.6 Hz, 1H), 1.06 (d, *J* = 6.6 Hz, 3H), 0.80 (t, *J* = 6.1 Hz, 6H) ppm. ^13^C NMR (151 MHz, CDCl_3_) δ 167.81, 161.78 (dd, *J* = 247.9, 8.6 Hz), 161.73 (dd, *J* = 247.8, 8.7 Hz), 161.52 (t, *J* = 13.8 Hz), 158.62, 154.83, 153.63, 146.28, 103.18 (t, *J* = 20.7 Hz), 98.80 (d, *J* = 25.9 Hz), 89.77, 66.27, 55.07, 54.71, 43.30, 33.67, 25.03, 18.21, 18.12, 18.01 ppm. HRMS (ES^+^) calcd for C_21_H_28_ClF_2_N_6_O [M + H]^+^, 453.1976; found 453.1979.

##### (*S*)-4-(4-(5-Chloro-7-((1,1,1-trifluoropropan-2-yl)amino)-[1,2,4]triazolo[1,5-*a*]pyrimidin-6-yl)-3,5-difluorophenoxy)butan-1-ol (**19**)

General procedure B was followed using butane-1,4-diol (36 mg, 0.40 mmol), NaH (60% in oil, 16 mg, 0.40 mmol), and **2** (40 mg, 0.10 mmol). Purification via preparative reversed-phase chromatography provided compound **19** as a white solid (38 mg, 82% yield). ^1^H NMR (500 MHz; CDCl_3_): δ 8.40 (s, 1H), 6.65–6.63 (m, 2H), 5.92 (d, *J* = 10.2 Hz, 1H), 4.77 (br s, 1H), 4.08 (t, *J* = 6.3 Hz, 2H), 3.78 (t, *J* = 6.3 Hz, 2H), 1.99–1.93 (m, 2H), 1.82–1.76 (m, 2H), 1.41 (d, *J* = 6.8 Hz, 3H) ppm. ^13^C NMR (126 MHz; CDCl_3_): δ 162.87 (t, *J* = 13.8 Hz), 161.77 (dd, *J* = 249.8, 9.0 Hz), 161.51 (dd, *J* = 248.2, 9.0 Hz), 158.87, 155.06, 153.93, 146.11, 127.99, 125.75, 123.51, 121.27, 100.44 (t, *J* = 21.1 Hz), 99.31 (ddd, *J* = 30.4, 25.6, 3.4 Hz), 92.82, 69.08, 62.48, 50.87 (q, *J* = 32.1 Hz), 29.17, 25.66, 15.31 ppm. IR: ν 3344, 2945, 2882, 1616, 1575, 1558, 1184, 1156, cm^–1^. HRMS (ES^+^) calcd for C_18_H_18_ClF_5_N_5_O_2_ [M + H]^+^, 466.1069; found 466.1069.

##### (*S*)-5-(4-(5-Chloro-7-((1,1,1-trifluoropropan-2-yl)amino)-[1,2,4]triazolo[1,5-*a*]pyrimidin-6-yl)-3,5-difluorophenoxy)pentan-1-ol (**20**)

General procedure B was followed using pentane-1,5-diol (31 mg, 0.32 mmol), NaH (60% in oil, 13 mg, 0.32 mmol), and **2** (30 mg, 0.08 mmol). Purification via preparative reversed-phase chromatography provided compound **20** as a white solid (25 mg, 68% yield). ^1^H NMR (500 MHz; CDCl_3_): δ 8.38 (s, 1H), 6.63 (dd, *J* = 9.1, 2.3 Hz, 2H), 5.93 (d, *J* = 9.8 Hz, 1H), 4.77–4.76 (br s, 1H), 4.03 (t, *J* = 6.3 Hz, 2H), 3.71 (t, *J* = 6.3 Hz, 2H), 1.88 (dt, *J* = 14.2, 6.9 Hz, 2H), 1.67 (dt, *J* = 14.2, 6.9 Hz, 2H), 1.62–1.56 (m, 2H), 1.40 (d, *J* = 6.8 Hz, 3H) ppm. ^13^C NMR (126 MHz; CDCl_3_): δ 162.90 (t, *J* = 14.0 Hz), 161.76 (dd, *J* = 249.9, 9.1 Hz), 161.50 (dd, *J* = 248.1, 9.0 Hz), 158.63, 155.33, 154.12, 146.08, 124.65 (q, *J* = 282.0 Hz), 100.45 (t, *J* = 21.0 Hz), 99.25 (ddd, *J* = 29.3, 25.7, 3.4 Hz), 92.60, 69.15, 62.74, 50.82 (q, *J* = 32.1 Hz), 32.42, 28.84, 22.48, 15.30 ppm. IR (film): ν 3336, 2949, 2873, 1617, 1575, 1553, 1184, 1146, 1036 cm^–1^. HRMS (ES^+^) calcd for C_19_H_20_ClF_5_N_5_O_2_ [M + H]^+^, 480.1226; found 480.1232.

##### (*S*)-3-(4-(5-Chloro-7-((3-methylbutan-2-yl)amino)-[1,2,4]triazolo[1,5-*a*]pyrimidin-6-yl)-3,5-difluorophenoxy)-*N*,*N*-dimethylpropan-1-aminium Formate (**21**)

General procedure B was followed using 3-(dimethylamino)propan-1-ol (63 μL, 0.54 mmol), NaH (60% in oil, 22 mg, 0.54 mmol), and **7** (50 mg, 0.14 mmol). Purification via preparative reversed-phase chromatography provided compound **21** as a white solid (26 mg, 39% yield). ^1^H NMR (600 MHz, CDCl_3_) δ 9.21 (s, 1H), 8.41 (s, 1H), 8.30 (s, 1H), 6.59 (d, *J* = 9.1 Hz, 2H), 6.31 (s, 1H), 4.11 (t, *J* = 5.2 Hz, 2H), 3.29 (s, 1H), 3.20–2.96 (m, 2H), 2.70 (s, 6H), 2.26 (dq, *J* = 11.9, 5.9 Hz, 1H), 1.64 (dq, *J* = 13.0, 6.6 Hz, 1H), 1.05 (d, *J* = 6.6 Hz, 3H), 0.79 (t, *J* = 6.2 Hz, 6H) ppm. ^13^C NMR (151 MHz, CDCl_3_) δ 167.60, 161.76 (dd, *J* = 247.9, 8.7 Hz), 161.71 (dd, *J* = 247.7, 8.8 Hz), 161.47 (t, *J* = 13.9 Hz), 158.60, 154.80, 153.61, 146.27, 103.18 (t, *J* = 20.8 Hz), 98.80 (dd, *J* = 25.9, 2.4 Hz), 89.75, 66.17, 54.99, 54.72, 43.13, 33.65, 24.84, 18.20, 18.10, 18.00 ppm. HRMS (ES^+^) calcd for C_21_H_28_ClF_2_N_6_O [M + H]^+^, 453.1976; found 453.1979.

##### (*R*)-3-(4-(5-Chloro-7-((1,1,1-trifluoro-3-methylbutan-2-yl)amino)-[1,2,4]triazolo[1,5-*a*]pyrimidin-6-yl)-3,5-difluorophenoxy)-*N*,*N*-dimethylpropan-1-aminium Formate (**22**)

General procedure B was followed using 3-(dimethylamino)propan-1-ol (66 μL, 0.57 mmol), NaH (60% in oil, 23 mg, 0.57 mmol), and **8** (60 mg, 0.14 mmol). Purification via preparative reversed-phase chromatography provided compound **21** as a white solid (43 mg, 55% yield). ^1^H NMR (500 MHz, CDCl_3_) δ 8.38 (s, 1H), 6.65 (t, *J* = 10.0 Hz, 2H), 6.42 (s, 1H), 4.09 (t, *J* = 6.1 Hz, 2H), 2.48 (t, *J* = 6.9 Hz, 2H), 2.28 (s, 6H), 2.20–2.10 (m, 1H), 2.01 (p, *J* = 6.5 Hz, 2H), 1.01 (d, *J* = 6.8 Hz, 3H), 0.96 (d, *J* = 6.7 Hz, 3H). ^13^C NMR (126 MHz, CDCl_3_) δ 168.49, 162.83 (t, *J* = 13.8 Hz), 161.62 (dd, *J* = 250.0, 8.9 Hz), 161.44 (dd, *J* = 248.0, 8.9 Hz), 158.87, 155.31, 153.88, 146.62, 124.59 (q, *J* = 283.6 Hz), 100.10, 99.34 (dd, *J* = 25.6, 3.5 Hz), 99.22 (t, *J* = 25.2 Hz)*, 67.38, 58.62 (q, *J* = 29.4 Hz), 56.05, 45.57, 28.25, 27.20, 19.71, 16.67 ppm. * two C–H of aryl ring in the same signal. IR (KBr) ν 3423, 2970, 1617, 1574, 1574, 1494, 1445, 1357, 1263, 1157, 1090, 1037, 936, 842, 768, 731, 652 cm^–1^ HRMS (ES^+^) calcd for C_21_H_25_ClF_5_N_6_O [M + H]^+^, 507.1693; found 507.1706.

##### (*S*)-3-(4-(5-Chloro-7-((1,1,1-trifluoro-3-methylbutan-2-yl)amino)-[1,2,4]triazolo[1,5-*a*]pyrimidin-6-yl)-3,5-difluorophenoxy)-*N*,*N*-dimethylpropan-1-aminium Formate (**23**)

General procedure B was followed using 3-(dimethylamino)propan-1-ol (63 μL, 0.54 mmol), NaH (60% in oil, 22 mg, 0.54 mmol), and **9** (57 mg, 0.14 mmol). Purification via preparative reversed-phase chromatography provided compound **21** as a white solid (49 mg, 66% yield). ^1^H NMR (500 MHz, CDCl_3_) δ 8.38 (s, 1H), 6.66 (t, *J* = 9.8 Hz, 2H), 4.09 (t, *J* = 6.4 Hz, 2H), 2.48 (t, *J* = 7.0 Hz, 2H), 2.28 (s, 6H), 2.15 (dq, *J* = 13.2, 6.6 Hz, 1H), 2.01 (p, *J* = 6.7 Hz, 2H), 1.01 (d, *J* = 6.7 Hz, 3H), 0.96 (d, *J* = 6.7 Hz, 3H) ppm. ^13^C NMR (126 MHz, CDCl_3_) δ 162.91 (t, *J* = 13.8 Hz), 161.63 (dd, *J* = 250.0, 9.0 Hz), 161.45 (dd, *J* = 247.9, 8.9 Hz), 155.32, 146.64, 124.59 (q, *J* = 283.6 Hz), 99.53–98.89 (m), 67.38, 58.62 (q, *J* = 29.0 Hz), 56.04, 45.57, 28.25, 27.19, 19.71, 16.66 ppm. HRMS (ES^+^) calcd for C_21_H_25_ClF_5_N_6_O [M + H]^+^, 507.1693; found 507.1706.

##### (*R*)-3-(4-(5-Chloro-7-((3,3-dimethylbutan-2-yl)amino)-[1,2,4]triazolo[1,5-*a*]pyrimidin-6-yl)-3,5-difluorophenoxy)-*N*,*N*-dimethylpropan-1-aminium Formate (**24**)

General procedure B was followed using 3-(dimethylamino)propan-1-ol (61 μL, 0.52 mmol), NaH (60% in oil, 21 mg, 0.52 mmol), and **10** (50 mg, 0.13 mmol). Purification via preparative reversed-phase chromatography provided compound **24** as a formic salt adduct as a white powder (62 mg, 0.12 mmol, 93%). ^1^H NMR (600 MHz, CDCl_3_) δ 8.43 (s, 1H), 8.28 (s, 1H), 8.02 (s, 1H), 6.58 (d, *J* = 6.0 Hz, 2H), 6.37 (bs, 1H), 4.09 (t, *J* = 6.0 Hz, 2 H), 3.03 (t, *J* = 6.0 Hz, 2 H), 2.66 (s, 6H), 2.22 (quint, *J* = 6.0 Hz, 2H), 0.98 (t, *J* = 6.0 Hz, 3H) 0.80 (s, 9H) ppm. ^13^C NMR (151 MHz, CDCl_3_) δ 167.94, 161.27 (dd, *J* = 247.6, 9.1 Hz), 161.56 (t, *J* = 13.6 Hz), 161.45 (dd, *J* = 247.6, 9.1 Hz), 158.56, 154.72, 153.54, 146.42, 103.09 (t, *J* = 21.1 Hz), 98.83 (dd, *J* = 26.4, 1.5 Hz), 98.74 (dd, *J* = 25.7, 1.5 Hz), 89.57, 66.26, 57.85, 54.94, 43.20, 34.68, 25.79, 24.93, 16.61 ppm. ^19^F NMR (376 MHz, DMSO-*D*_6_) δ −112.70, −114.09 (d, *J* = 11.3 Hz) ppm. IR (film) ν 1607, 1571, 1496, 1443, 1349, 1259, 1152, 1029, 765 cm^–1^. HRMS (ES^+^) calcd for C_22_H_30_ClF_2_N_6_O [M + H]^+^, 467.2132; found 467.2133.

##### (*R*)-3-(4-(5-Chloro-7-((3,3-dimethylbutan-2-yl)amino)-[1,2,4]triazolo[1,5-*a*]pyrimidin-6-yl)-3,5-difluorophenoxy)propan-1-ol (**25**)

General procedure B was followed using propan-1,3-diol (29 μL, 0.40 mmol), NaH (60% in oil, 16 mg, 0.40 mmol) and **10** (39 mg, 0.10 mmol). Purification via preparative reversed-phase chromatography provided compound **25** as a white powder (9 mg, 0.02 mmol, 20%). ^1^H NMR (600 MHz, CDCl_3_) δ 8.32 (s, 1H), 6.63 (d, *J* = 9.7 Hz, 2H), 6.37 (bs, 1H), 4.19 (t, *J* = 6.2 Hz, 2H), 3.90 (t, *J* = 5.9 Hz, 2H), 3.28 (bs, 1H), 2.22–1.94 (m, 2H), 1.02 (d, *J* = 6.7 Hz, 3H), 0.84 (s, 9H) ppm. ^13^C NMR (151 MHz, CDCl_3_) δ 162.15 (t, *J* = 13.6 Hz),161.80 (dd, *J* = 248.6, 8.6 Hz), 161.57 (dd, *J* = 247.6, 8.6 Hz), 158.78, 154.78, 146.59, 100.86–96.50 (m), 89.88, 66.28, 59.43, 57.91, 34.75, 31.88, 25.87, 16.70 ppm. HRMS (ES^+^) calcd for C_20_H_24_ClF_2_N_5_O_2_ [M + H]^+^, 439.1587; found 439.1588.

##### 5-Chloro-6-(4-(3-(dimethylamino)propoxy)-2,6-difluorophenyl)-*N*-neopentyl-[1,2,4]triazolo[1,5-*a*]pyrimidin-7-amine (**26**)

General procedure B was followed using 3-(dimethylamino)propan-1-ol (51 μL, 0.43 mmol), NaH (60% in oil, 17 mg, 0.43 mmol), and **11** (40 mg, 0.11 mmol). Purification by reversed-phase HPLC provided compound **26** as a white powder (24 mg, 0.053 mmol, 49%). ^1^H NMR (600 MHz, CDCl_3_) δ 8.33 (s, 1H), 6.61 (d, *J* = 6.0 Hz, 2H), 6.36 (bs, 1H), 4.07 (t, *J* = 6.0 Hz, 2 H), 2.89 (d, *J* = 6.0 Hz, 2 H), 2.48 (t, *J* = 6.0 Hz, 2H), 2.28 (s, 6H), 2.01 (quint, *J* = 6.0 Hz, 2H), 0.89 (s, 9H) ppm. ^13^C NMR (151 MHz, CDCl_3_) δ 162.19 (t, *J* = 15.1 Hz), 161.86 (dd, *J* = 247.6, 9.1 Hz), 158.68, 154.92, 153.67, 146.94, 102.51 (t, *J* = 22.7 Hz), 98.82–98.63 (m), 90.13, 67.27, 56.08, 54.47, 45.58, 31.87, 27.22, 26.91 ppm. IR (film) ν 1610, 1571, 1561, 1462, 1441, 1429, 1352, 1247, 1152, 1138, 1027, 765 cm^–1^. HRMS (ES^+^) calcd for C_21_H_28_ClF_2_N_6_O [M + H]^+^, 453.1976; found 453.1974.

##### (*S*)-5-Chloro-6-(4-(3-(dimethylamino)propoxy)-2,6-difluorophenyl)-*N*-(3,3-dimethylbutan-2-yl)-[1,2,4]triazolo[1,5-*a*]pyrimidin-7-aminium Formate (**27**)

General procedure B was followed using 3-(dimethylamino)propan-1-ol (61 μL, 0.52 mmol), NaH (60%, 21 mg, 0.52 mmol), and **12** (50 mg, 0.13 mmol). Purification by reversed-phase HPLC provided compound **27** as a formic salt adduct as a white powder (66 mg, 0.12 mmol, 98%). ^1^H NMR (600 MHz, CDCl_3_) δ 8.43 (s, 1H), 8.27 (s, 1H), 7.95 (s, 1H), 6.57 (d, *J* = 6.0 Hz, 2H), 6.36 (bs, 1H), 4.0 (t, *J* = 6.0 Hz, 2 H), 3.04 (t, *J* = 6.0 Hz, 2 H), 2.66 (s, 6H), 2.22 (quint, *J* = 6.0 Hz, 2H), 0.97 (t, *J* = 6.0 Hz, 3H) 0.79 (s, 9H) ppm. ^13^C NMR (151 MHz, CDCl_3_) δ 167.93, 161.27 (dd, *J* = 247.6, 9.1 Hz), 161.56 (t, *J* = 13.6 Hz), 161.45 (dd, *J* = 247.6, 9.1 Hz), 158.53, 154.68, 153.51, 146.41, 103.09 (t, *J* = 21.1 Hz), 98.83 (dd, *J* = 26.4, 1.5 Hz), 98.74 (dd, *J* = 25.7, 1.5 Hz), 89.56, 67.99, 66.23, 57.83, 54.89, 43.15, 34.65, 25.77, 24.88, 16.58 ppm. IR (film) ν 1607, 1571, 1496, 1443, 1349, 1259, 1152, 1029, 954, 765 cm^–1^. HRMS (ES^+^) calcd for C_22_H_30_ClF_2_N_6_O [M + H]^+^, 467.2132; found 467.2132.

##### 5-Chloro-6-(4-(3-(dimethylamino)propoxy)-2,6-difluorophenyl)-*N*-((1-methylcyclopropyl)methyl)-[1,2,4]triazolo[1,5-*a*]pyrimidin-7-amine (**28**)

General procedure B was followed using 3-(dimethylamino)propan-1-ol (44 μL, 0.37 mmol), NaH (60%, 15 mg, 0.37 mmol), and **13** (34 mg, 0.092 mmol). Purification by reversed-phase HPLC provided compound **28** as a white powder (25 mg, 0.055 mmol, 60%). ^1^H NMR (600 MHz, CDCl_3_) δ 8.33 (s, 1H), 6.59 (d, *J* = 12.0 Hz, 2H), 6.37 (bs, 1H), 4.07 (t, *J* = 6.0 Hz, 2 H), 2.50 (t, *J* = 6.0 Hz, 2 H), 2.29 (s, 6H), 2.01 (quint, *J* = 6.0 Hz, 2H), 1.08 (s, 3H), 0.42–0.38 (m, 4H) ppm. ^13^C NMR (151 MHz, CDCl_3_) δ 162.13 (t, *J* = 13.6 Hz), 161.93 (dd, *J* = 246.1, 9.1 Hz), 158.68, 154.95, 153.62, 146.66, 102.47(t, *J* = 21.1 Hz), 98.77–98.58 (m), 90.05, 67.19, 56.08, 52.57, 45.54, 27.18, 21.01, 16.26, 11.91 ppm. IR (film) ν 1608, 1571, 1561, 1495, 1455, 1441, 1426, 1364, 1342, 1260, 1246, 1205, 1153, 1139, 1119, 1057, 1030, 1017, 927, 912, 820, 765, 559, 540, 515 cm^–1^. HRMS (ES^+^) calcd for C_21_H_25_ClF_2_N_6_O [M + H]^+^, 451.1819; found 451.1816.

##### 5-Chloro-6-(4-(3-(dimethylamino)propoxy)-2,6-difluorophenyl)-*N*-((1-(trifluoromethyl)cyclopropyl)methyl)-[1,2,4]triazolo[1,5-*a*]pyrimidin-7-amine (**29**)

General procedure B was followed using 3-(dimethylamino)propan-1-ol (40 μL, 0.34 mmol), NaH (60%, 14 mg, 0.34 mmol), and **14** (36 mg, 0.085 mmol). Purification by reversed-phase HPLC provided compound **29** as a white powder (25 mg, 0.050 mmol, 58%). ^1^H NMR (600 MHz, CD_3_CN) δ 8.33 (s, 1H), 6.76 (d, *J* = 6.0 Hz, 2H), 6.59 (bs, 1H), 4.11 (t, *J* = 6.0 Hz, 2 H), 3.85 (bs, 2H), 2.48 (t, *J* = 6.0 Hz, 2 H), 2.24 (s, 6H), 1.97–1.94 (m, 2H), 0.97 (t, *J* = 6 Hz, 2H), 0.82 (bs, 2H) ppm. ^13^C NMR (151 MHz, CD_3_CN): selected representative peaks δ 163.56, 161.93, 155.67, 148.32, 100.06–99.87 (m), 68.04, 56.10, 45.21, 44.52, 27.31, 7.88 ppm. HRMS (ES^+^) calculated for C_21_H_23_ClF_5_N_6_O [M + H]^+^, 505.1537; found 505.1533.

##### 5-Chloro-*N*-(cyclobutylmethyl)-6-(4-(3-(dimethylamino)propoxy)-2,6-difluorophenyl)-[1,2,4]triazolo[1,5-*a*]pyrimidin-7-amine (**30**)

General procedure B was followed using 3-(dimethylamino)propan-1-ol (55 μL, 0.47 mmol), NaH (60%, 19 mg, 0.47 mmol), and **15** (43 mg, 0.12 mmol). Purification by reversed-phase HPLC provided compound **30** as a white powder (30 mg, 0.067 mmol, 57%). ^1^H NMR (600 MHz, CDCl_3_) δ 8.32 (s, 1H), 6.60 (d, *J* = 6.0 Hz, 2H), 6.22 (bs, 1H), 4.07 (t, *J* = 6.0 Hz, 2 H), 3.06 (t, *J* = 6.0 Hz, 2 H), 2.49–2.45 (m, 3H), 2.27 (s, 6H), 2.06–1.99 (m, 4H), 1.95–1.79 (m, 2H), 1.59 (quint, *J* = 6.0 Hz, 2H) ppm. ^13^C NMR (151 MHz, CDCl_3_) δ 162.14 (t, *J* = 13.6 Hz), 161.98 (dd, *J* = 246.89, 9.1 Hz), 158.68, 154.91, 153.62, 146.58, 102.40 (t, *J* = 22.7 Hz), 98.75–98.55 (m), 90.17, 67.22, 56.08, 48.66, 45.56, 34.98, 27.20, 25.47, 18.24 ppm. IR (film) ν 1611, 1572, 1562, 1461, 1441, 1353, 1336, 1248, 1206, 1151, 1137, 1027, 838 cm^–1^. HRMS (ES^+^) calcd for C_21_H_25_ClF_2_N_6_O [M + H]^+^, 451.1819; found 451.1816.

##### (*S*)-3-(4-(5-Chloro-7-((1-cyclopropylethyl)amino)-[1,2,4]triazolo[1,5-*a*]pyrimidin-6-yl)-3,5-difluorophenoxy)-*N*,*N*-dimethylpropan-1-aminium Formate (**31**)

General procedure B was followed using 3-(dimethylamino)propan-1-ol (64 μL, 0.54 mmol), NaH (60%, 21 mg, 0.54 mmol), and **16** (50 mg, 0.14 mmol). Purification by reversed-phase HPLC provided compound **31** as a white powder (22 mg, 0.04 mmol, 33%). ^1^H NMR (600 MHz, CDCl_3_) δ 8.41 (s, 1H), 8.30 (s, 1H), 6.56 (t, *J* = 10.9 Hz, 2H), 6.37 (d, *J* = 8.6 Hz, 1H), 4.10 (t, *J* = 4.9 Hz, 2H), 3.32–3.01 (m, 2H), 3.02 (s, 1H), 2.72 (s, 6H), 2.25 (s, 2H), 1.11 (d, *J* = 6.5 Hz, 3H), 0.88 (dt, *J* = 8.5, 4.7 Hz, 1H), 0.49 (ddt, *J* = 18.6, 9.0, 5.0 Hz, 2H), 0.18 (dq, *J* = 9.9, 4.8 Hz, 1H), 0.05 (dt, *J* = 9.3, 4.8 Hz, 1H) ppm. ^13^C NMR (151 MHz, CDCl_3_) δ 167.60, 161.76 (dd, *J* = 247.5, 8.7 Hz), 161.58 (dd, *J* = 248.0, 9.1 Hz), 161.47 (t, *J* = 13.9 Hz), 158.41, 154.77, 153.77, 145.98, 102.89 (t, *J* = 20.9 Hz), 98.78 (ddd, *J* = 50.7, 25.8, 2.9 Hz), 89.84, 66.10, 54.93, 43.07, 24.75, 21.10, 17.75, 3.52, 3.46 ppm. HRMS (ES^+^) calcd for C_21_H_26_ClF_2_N_6_O [M + H]^+^, 451.1819; found 451.1817.

##### (*R*)-3-(4-(5-Chloro-7-((1-cyclopropylethyl)amino)-[1,2,4]triazolo[1,5-*a*]pyrimidin-6-yl)-3,5-difluorophenoxy)-*N*,*N*-dimethylpropan-1-aminium Formate (**32**)

General procedure B was followed using 3-(dimethylamino)propan-1-ol (64 μL, 0.54 mmol), NaH (60%, 21 mg, 0.54 mmol), and **17** (50 mg, 0.14 mmol). Purification by reversed-phase HPLC provided compound **31** as a formic acid salt as a white powder (23 mg, 0.04 mmol, 34%). ^1^H NMR (600 MHz, CDCl_3_) δ 8.41 (s, 1H), 8.30 (s, 1H), 6.56 (t, *J* = 10.9 Hz, 2H), 6.37 (d, *J* = 8.6 Hz, 1H), 4.10 (t, *J* = 4.9 Hz, 2H), 3.32–3.01 (m, 2H), 3.02 (s, 1H), 2.72 (s, 6H), 2.25 (s, 2H), 1.11 (d, *J* = 6.5 Hz, 3H), 0.88 (dt, *J* = 8.5, 4.7 Hz, 1H), 0.49 (ddt, *J* = 18.6, 9.0, 5.0 Hz, 2H), 0.18 (dq, *J* = 9.9, 4.8 Hz, 1H), 0.05 (dt, *J* = 9.3, 4.8 Hz, 1H) ppm. ^13^C NMR (151 MHz, CDCl_3_) δ 167.60, 161.76 (dd, *J* = 247.5, 8.7 Hz), 161.58 (dd, *J* = 248.0, 9.1 Hz), 161.47 (t, *J* = 13.9 Hz), 158.41, 154.77, 153.77, 145.98, 102.89 (t, *J* = 20.9 Hz), 98.78 (ddd, *J* = 50.7, 25.8, 2.9 Hz), 89.84, 66.10, 54.93, 43.07, 24.75, 21.10, 17.75, 3.52, 3.46 ppm. HRMS (ES^+^) calcd for C_21_H_26_ClF_2_N_6_O [M + H]^+^, 451.1819; found 451.1814.

##### (*R*)-5-Chloro-6-(4-ethynyl-2,6-difluorophenyl)-*N*-(3-methylbutan-2-yl)-[1,2,4]triazolo[1,5-*a*]pyrimidin-7-amine (**34**)

To a solution of **33** (0.047 g, 0.125 mmol, 1 equiv) in dry toluene (12 mL) at 0 °C was added D*i*BAl-H (0.170 mL, 1.1 M, 0.027 g, 0.182 mmol, 1.5 equiv) dropwise, and the mixture was stirred for 1 h at this temperature before it was quenched with a 1 M solution of HCl. The aqueous layer was extracted twice with EtOAc, and then the combined organic fractions were washed with brine, dried, and concentrated under reduced pressure to furnish the aldehyde intermediate, which was directly dissolved into MeOH (4 mL). K_2_CO_3_ (0.025 g, 0.182 mmol, 1.5 equiv) and dimethyl (1-diazo-2-oxopropyl)phosphonate (0.035 g, 0.182 mmol, 1.5 equiv) were successively added, and the mixture was stirred at rt for 3 h. The reaction was then filtered over sintered glass, and the filtrate was evaporated under reduced pressure. Purification via silica gel flash chromatography (Hexanes/EtOAc: 90/10 to 75/25) provided compound **34** as an off-white solid (0.005 g, 0.012 mmol, 10% over two steps). ^1^H NMR (600 MHz, CDCl_3_) δ 8.34 (s, 1H), 7.20 (d, *J* = 8.3 Hz, 2H), 6.36 (d, *J* = 9.8 Hz, 1H), 3.31 (s, 1H), 3.16 (bs, 1H), 1.75–1.49 (m, 1H), 1.06 (d, *J* = 6.7 Hz, 3H), 0.81 (d, *J* = 6.7 Hz, 3H), 0.79 (d, *J* = 6.8 Hz, 3H) ppm. ^13^C NMR (151 MHz, CDCl_3_) δ 160.89 (dd, *J* = 249.9, 7.7 Hz), 160.82 (dd, *J* = 250.0, 6.9 Hz), 157.85, 154.94, 145.99, 126.40 (t, *J* = 12.0 Hz), 115.66 (ddd, *J* = 24.0, 7.7, 3.6 Hz), 112.16 (t, *J* = 20.4 Hz), 89.18, 81.37, 80.79, 54.95, 33.69, 29.83, 18.11, 18.08, 17.92 ppm. HRMS (ES^+^) calcd for C_18_H_17_N_5_F_2_Cl [M + H]^+^, 376.1135; found 376.1139.

##### Diethyl 2-(4-Amino-2,6-difluorophenyl)malonate (**36**)

To a cooled solution of NaH (1.887 g, 47.44 mmol, 2.1 equiv) in tetrahydrofuran (THF, 60 mL) was added diethylmalonate (6.891 mL, 7.236 g, 45.18 mmol, 2 equiv) and stirred for 1 h at rt before adding **35** (2.63 mL, 4.000 g, 22.59 mmol, 1 equiv) dropwise and stirred for 2 h at rt. The reaction was quenched with a NH_4_Cl_Sat._ solution and extracted with EtOAc twice. The combined organic fractions were washed with water and brine, dried over Na_2_SO_4_, and concentrated under vacuum. Filtration over a silica pad (Hexanes/EtOAc: 95/5) provided the nitro malonate intermediate + diethylmalonate (ratio 1/1), which will be used in the next step without further purification. To a solution of the intermediate nitro malonate in MeOH (150 mL) backfilled with N_2_ was added Pd/C (2.404 g, 10% weight, 2.259 mmol, 0.1 equiv). The mixture was put under atmospheric hydrogen pressure for 4 h. The flask was backfilled three times with N_2_ before the solution was filtered over Celite and rinsed with MeOH. The filtrate was concentrated and purified over silica gel flash chromatography (Hexanes/EtOAc: 98/02 to 85/15) to provide compound **36** (2.590 g, 9.02 mmol, 39% over 2 steps) as a pale yellow oil. ^1^H NMR (599 MHz, CDCl_3_) δ 6.18 (d, *J* = 9.9 Hz, 2H), 4.80 (s, 1H), 4.23 (q, *J* = 7.2 Hz, 4H), 3.82 (bs, 2H), 1.26 (t, *J* = 7.2 Hz, 6H) ppm. ^13^C NMR (151 MHz, CDCl_3_) δ 167.72, 162.81 (d, *J* = 10.5 Hz), 161.17 (d, *J* = 10.4 Hz), 148.66 (t, *J* = 14.1 Hz), 99.62 (t, *J* = 19.5 Hz), 98.34–97.10 (m), 62.08, 46.92, 14.05 ppm. LCMS: [M + H]^+^, 288.

##### Diethyl 2-(2,6-Difluoro-4-iodophenyl)malonate (**37**)

To a solution of **36** (2.050 g, 7.136 mmol, 1 equiv) in 6 N HCl (12 mL, 71.36 mmol, 10 equiv) cooled to 0 °C, a solution of NaNO_2_ (0.492 g, 7.136 mmol, 1 equiv) in water (2.7 mL) was added dropwise. The resulting solution was added dropwise to a solution of KI (4.916 g, 29.62 mmol, 4.15 equiv) in water (5 mL) keeping the temperature at 0 °C. The reaction mixture was allowed to warm to room temperature and stirred for 3 h, then it was stopped and extracted twice with EtOAc. The combined layers were washed in sequence with 10% Na_2_S_2_O_3_ and brine, then dried over Na_2_SO_4_ and concentrated under vacuum. Purification via silica gel flash chromatography (Hexanes/EtOAc: 100/0 to 75/25) provided compound **37** (2.054 g, 5.159 mmol, 72%) as a yellow oil. ^1^H NMR (600 MHz, CDCl_3_) δ 7.31 (d, *J* = 7.0 Hz, 1H), 4.89 (s, 1H), 4.25 (q, *J* = 7.1 Hz, 4H), 1.27 (t, *J* = 7.2 Hz, 6H) ppm. ^13^C NMR (151 MHz, CDCl_3_) δ 166.36, 160.77 (dd, *J* = 255.2, 7.8 Hz), 121.41 (dd, *J* = 23.6, 4.8 Hz), 101.61 (dt, *J* = 20.3, 14.7 Hz), 62.45, 47.22, 14.05 ppm. LCMS: [M + H]^+^, 339.

##### 5,7-Dichloro-6-(2,6-difluoro-4-iodophenyl)-[1,2,4]triazolo[1,5-*a*]pyrimidine (**38**)

A pressure flask flushed with nitrogen was charged with **37** (1.920 g, 6.459 mmol, 1 equiv), 1*H*-1,2,4-triazol-5-amine (0.570 g, 6.782 mmol, 1.05 equiv), and tributylamine (1.620 mL, 6.782 mmol, 1.05 equiv). The mixture was heated to 170 °C for 2 h. Toluene (10 mL) was then added at 110 °C followed by a 50% NaOH solution (1.023 mL, 19.38 mmol, 3 equiv) at 50 °C. Once at rt, the mixture was filtered over sintered glass and rinsed twice with toluene to furnish the bis-phenolate intermediate (2.068 g, 6.621 mmol, 97%) that is engaged in the next step without further purification. In a pressure flask charged with the intermediate bis-phenolate (2.000 g, 4.608 mmol, 1 equiv) was added phosphoryl chloride (7.670 mL, 82.020 mmol, 17.8 equiv) and the mixture was heated to 130 °C for 6 h. The reaction was poured over ice, the aqueous phase was extracted twice with CH_2_Cl_2_, and the combined organic fractions were washed with brine, dried, and concentrated under reduced pressure. Purification via silica gel flash chromatography (Hexanes/EtOAc: 100/0 to 70/30) provided compound **38** as an off-white solid (1.520 g, 3.56 mmol, 77%). ^1^H NMR (600 MHz, CDCl_3_) δ 8.61 (s, 1H), 7.52 (d, *J* = 6.4 Hz, 2H) ppm. ^13^C NMR (151 MHz, CDCl_3_) δ 160.45 (d, *J* = 6.2 Hz), 158.75 (d, *J* = 6.2 Hz), 157.28, 156.13, 153.85, 141.28, 122.05 (dd, *J* = 23.0, 4.2 Hz), 112.35, 109.18 (t, *J* = 19.8 Hz), 95.68 (t, *J* = 10.2 Hz) ppm. HRMS (ES^+^) calcd for C_11_H_4_N_4_F_2_ICl_2_ [M + H]^+^, 426.8820; found 426.8812.

##### (*S*)-5-Chloro-6-(2,6-difluoro-4-iodophenyl)-*N*-(1,1,1-trifluoropropan-2-yl)-[1,2,4]triazolo[1,5-*a*]pyrimidin-7-amine (**39**)

General procedure A was followed: To a solution of **38** (0.200 g, 0.468 mmol, 1 equiv) in DMF (2 mL) was added (*S*)-1,1,1-trifluoropropan-2-amine (0.097 mL, 0.111 g, 0.984 mmol, 2.1 equiv), and the mixture was stirred for 24 h at 40 °C. Purification via silica gel flash chromatography (Hexanes/EtOAc: 100/0 to 70/30) provided compound **39** as an off-white solid (0.121 g, 0.240 mmol, 51%). ^1^H NMR (600 MHz, CDCl_3_) δ 8.38 (s, 1H), 7.49 (d, *J* = 6.6 Hz, 2H), 5.97 (d, *J* = 9.6 Hz, 1H), 4.84 (bs, 1H), 1.43 (d, *J* = 6.8 Hz, 3H). ^13^C NMR (151 MHz, CDCl_3_) δ 160.61 (dd, *J* = 257.4, 5.9 Hz), 160.29 (dd, *J* = 255.8, 6.0 Hz), 157.41, 155.41, 154.26, 145.87, 124.59 (q, *J* = 282.1 Hz), 122.32 (dd, *J* = 63.0, 3.8 Hz), 122.16 (dd, *J* = 62.9, 3.7 Hz), 109.10 (t, *J* = 20.2 Hz), 95.55 (t, *J* = 10.1 Hz), 91.74, 51.08 (q, *J* = 32.1 Hz), 15.16 ppm. HRMS (ES^+^) calcd for C_14_H_9_N_5_F_5_ICl [M + H]^+^, 503.9506; found 503.9502.

##### (*R*)-5-Chloro-6-(2,6-difluoro-4-iodophenyl)-*N*-(3-methylbutan-2-yl)-[1,2,4]triazolo[1,5-*a*]pyrimidin-7-amine (**40**)

General procedure A was followed: To a solution of **38** (0.156 g, 0.365 mmol, 1 equiv) in DMF (1 mL) were added (*R*)-3-methylbutan-2-amine (0.064 mL, 0.048 g, 0.548 mmol, 1.5 equiv) and triethylamine (0.153 mL, 0.111 g, 1.100 mmol, 3 equiv), and the mixture was stirred for 1 h at rt. Purification via silica gel flash chromatography (Hexanes/EtOAc: 100/0 to 70/30) provided compound **40** as an off-white solid (0.161 g, 0.337 mmol, 92%). ^1^H NMR (600 MHz, CDCl_3_) δ 8.32 (s, 1H), 7.46 (d, *J* = 6.5 Hz, 2H), 6.35 (d, *J* = 7.8 Hz, 1H), 3.18 (s, 1H), 1.64 (dq, *J* = 13.2, 6.7 Hz, 1H), 1.06 (d, *J* = 6.6 Hz, 3H), 0.79 (dd, *J* = 11.6, 6.8 Hz, 6H) ppm. ^13^C NMR (151 MHz, CDCl_3_) δ 159.60 (dd, *J* = 254.9, 8.4 Hz), 159.56 (dd, *J* = 254.8, 8.4 Hz), 156.51, 153.82, 152.51, 144.76, 120.65 (dt, *J* = 24.4, 3.7 Hz), 110.04 (t, *J* = 21.1 Hz), 93.34 (t, *J* = 10.2 Hz), 87.81, 53.81, 32.50, 16.95, 16.94, 16.77 ppm. HRMS (ES^+^) calculated for C_16_H_16_N_5_F_2_ICl [M + H]^+^, 478.0101; found 478.0098.

##### (*R*)-5-Chloro-6-(2,6-difluoro-4-iodophenyl)-*N*-(3,3-dimethylbutan-2-yl)-[1,2,4]triazolo[1,5-*a*]pyrimidin-7-amine (**41**)

General procedure A was followed: To a solution of **38** (0.156 g, 0.365 mmol, 1 equiv) in DMF (1 mL) were added (*R*)-3,3-dimethylbutan-2-amine hydrochloride (0.075 g, 0.548 mmol, 1.5 equiv) and triethylamine (0.153 mL, 0.111 g, 1.100 mmol, 3 equiv), and the mixture was stirred for 1 h at rt. Purification via silica gel flash chromatography (Hexanes/EtOAc: 100/0 to 70/30) provided compound **41** as an off-white solid (0.158 g, 0.321 mmol, 88%). ^1^H NMR (600 MHz, CDCl_3_) δ 8.31 (s, 1H), 7.46 (d, *J* = 6.4 Hz, 2H), 6.42 (s, 1H), 3.10 (s, 1H), 1.00 (d, *J* = 6.7 Hz, 4H), 0.82 (s, 9H) ppm. ^13^C NMR (151 MHz, CDCl_3_) δ 160.67 (dd, *J* = 254.9, 6.2 Hz), 160.40 (dd, *J* = 254.8, 6.0 Hz), 157.59, 154.89, 153.58, 146.03, 121.82 (dd, *J* = 21.7, 3.9 Hz), 121.67 (dd, *J* = 21.7, 3.9 Hz), 111.23 (t, *J* = 18.2 Hz), 94.41 (t, *J* = 10.1 Hz), 88.71, 58.10, 34.74, 25.77, 16.56, 16.55 ppm. HRMS (ES^+^) calcd for C_17_H_18_N_5_F_2_ICl [M + H]^+^, 492.0258; found 492.0256.

##### (*S*)-4-(4-(5-Chloro-7-((1,1,1-trifluoropropan-2-yl)amino)-[1,2,4]triazolo[1,5-*a*]pyrimidin-6-yl)-3,5-difluorophenyl)but-3-yn-1-ol (**42**)

General procedure C was followed using **39** (0.032 g, 0.064 mmol) and but-3-yn-1-ol (0.014 mL, 0.013 g, 0.190 mmol). Purification by reversed-phase HPLC provided compound **42** as an off-white solid (0.011 g, 0.025 mmol, 39%). ^1^H NMR (600 MHz, CD_3_OD) δ 8.50 (s, 1H), 7.24 (d, *J* = 9.0 Hz, 2H), 5.77 (bs, 1H), 3.76 (t, *J* = 5.9 Hz, 2H), 3.32 (bs, 1H), 2.68 (t, *J* = 5.9 Hz, 2H), 1.46 (d, *J* = 6.5 Hz, 3H) ppm. ^13^C NMR (151 MHz, CDCl_3_) (selected representative peaks) δ 157.85, 155.52, 145.75, 143.53, 115.82, 115.65, 115.31, 115.16, 94.99, 61.61, 31.16, 16.12, 15.26 ppm. HRMS (ES^+^) calcd for C_18_H_14_N_5_F_5_ClO [M + H]^+^, 446.0802; found 446.0804.

##### (*S*)-5-(4-(5-Chloro-7-((1,1,1-trifluoropropan-2-yl)amino)-[1,2,4]triazolo[1,5-*a*]pyrimidin-6-yl)-3,5-difluorophenyl)pent-4-yn-1-ol (**43**)

General procedure C was followed using **39** (0.033 g, 0.066 mmol) and pent-4-yn-1-ol (0.020 mL, 0.017 g, 0.200 mmol). Purification by reversed-phase HPLC provided compound **43** as an off-white solid (0.010 g, 0.022 mmol, 33%). ^1^H NMR (600 MHz, CD_3_OD) δ 8.50 (s, 1H), 7.21 (d, *J* = 8.8 Hz, 2H), 5.80 (bs, 1H), 3.71 (t, *J* = 6.0 Hz, 2H), 3.32 (bs, 1H), 2.57 (t, *J* = 7.0 Hz, 2H), 1.94–1.71 (m, 2H), 1.46 (d, *J* = 6.7 Hz, 3H) ppm. ^13^C NMR (151 MHz, CD_3_OD) δ 163.05 (dd, *J* = 115.0, 7.0 Hz), 161.40 (dd, *J* = 114.6, 7.0 Hz), 158.58, 155.77, 149.07, 130.24 (t, *J* = 12.4 Hz), 126.50 (q, *J* = 281.6 Hz), 116.38 (dd, *J* = 23.5, 3.3 Hz), 116.08 (dd, *J* = 23.6, 3.3 Hz), 109.98 (t, *J* = 20.8 Hz), 95.25, 94.42, 79.42, 61.46, 52.43 (q, *J* = 31.7 Hz) ppm. HRMS (ES^+^) calcd for C_19_H_16_N_5_F_5_ClO [M + H]^+^, 460.0958; found 460.0961.

##### (*S*)-5-Chloro-6-(4-(4-(dimethylamino)but-1-yn-1-yl)-2,6-difluorophenyl)-*N*-(1,1,1-trifluoropropan-2-yl)-[1,2,4]triazolo[1,5-*a*]pyrimidin-7-amine (**44**)

General procedure A was followed using **39** (0.033 g, 0.066 mmol) and *N,N-*dimethylprop-2-yn-1-amine (0.023 mL, 0.019 g, 0.197 mmol). Purification by reversed-phase HPLC provided compound **44** as an off-white solid (0.012 g, 0.025 mmol, 39%). ^1^H NMR (600 MHz, CDCl_3_) δ 8.40 (s, 1H), 7.14 (dd, *J* = 8.4, 5.6 Hz, 2H), 5.94 (s, 1H), 4.67 (s, 1H), 2.84 (d, *J* = 7.1 Hz, 2H), 2.76 (d, *J* = 7.2 Hz, 2H), 2.48 (s, 6H), 1.40 (d, *J* = 6.8 Hz, 3H) ppm. HRMS (ES^+^) calcd for C_20_H_19_N_5_F_6_Cl [M + H]^+^, 473.1274; found 473.1277.

##### (*R*)-4-(4-(5-Chloro-7-((3-methylbutan-2-yl)amino)-[1,2,4]triazolo[1,5-*a*]pyrimidin-6-yl)-3,5-difluorophenyl)but-3-yn-1-ol (**45**)

General procedure C was followed using **40** (0.040 g, 0.084 mmol) and but-3-yn-1-ol (0.019 mL, 0.018 g, 0.250 mmol). Purification by reversed-phase HPLC provided compound **45** as an off-white solid (0.012 g, 0.029 mmol, 34%). ^1^H NMR (600 MHz, CD_3_OD) δ 8.44 (s, 1H), 7.26 (d, *J* = 8.4 Hz, 2H), 3.77 (t, *J* = 6.5 Hz, 2H), 3.31 (bs, 1H), 2.68 (t, *J* = 6.5 Hz, 2H), 1.80–1.66 (m, 1H), 1.11 (d, *J* = 6.6 Hz, 3H), 0.80 (d, *J* = 6.7 Hz, 6H) ppm. ^13^C NMR (151 MHz, CDCl_3_) (selected representative peaks) δ 161.58, 160.00, 157.65, 127.89, 115.19 (dd, *J* = 23.2, 8.8 Hz), 91.50, 89.36, 79.89, 60.95, 55.04, 33.69, 23.91, 18.11, 18.10, 17.97 ppm. ^19^F NMR (376 MHz, MeCN-*D*_3_) δ −110.61, −111.02 to −111.11 (m) ppm. HRMS (ES^+^) calcd for C_20_H_21_N_5_F_2_ClO [M + H]^+^, 420.1397; found 420.1398.

##### (*R*)-5-(4-(5-Chloro-7-((3-methylbutan-2-yl)amino)-[1,2,4]triazolo[1,5-*a*]pyrimidin-6-yl)-3,5-difluorophenyl)pent-4-yn-1-ol (**46**)

General procedure C was followed using **40** (0.027 g, 0.057 mmol) and pent-4-yn-1-ol (0.016 mL, 0.014 g, 0.170 mmol). Purification by reversed-phase HPLC provided compound **46** as an off-white solid (0.012 g, 0.028 mmol, 49%). ^1^H NMR (600 MHz, CD_3_OD) δ 8.43 (s, 1H), 7.22 (d, *J* = 8.4 Hz, 2H), 3.71 (t, *J* = 6.2 Hz, 2H), 3.31 (bs, 1H), 2.58 (t, *J* = 7.1 Hz, 2H), 1.84 (p, *J* = 6.7 Hz, 2H), 1.73 (dq, *J* = 13.6, 6.8 Hz, 2H), 1.11 (d, *J* = 6.6 Hz, 3H), 0.80 (d, *J* = 6.7 Hz, 6H) ppm. ^13^C NMR (151 MHz, CDCl_3_) (selected representative peaks) δ 161.67, 158.02, 154.94, 146.02, 128.27, 115.06 (d, *J* = 20.3 Hz), 94.29, 89.46, 61.63, 54.86, 33.70, 31.20, 18.11, 17.95, 16.12 ppm. HRMS (ES^+^) calcd for C_21_H_23_N_5_F_2_ClO [M + H]^+^, 434.1554; found 434.1553.

##### (*R*)-5-Chloro-6-(4-(3-(dimethylamino)prop-1-yn-1-yl)-2,6-difluorophenyl)-*N*-(3-methylbutan-2-yl)-[1,2,4]triazolo[1,5-*a*]pyrimidin-7-amine (**47**)

General procedure C was followed using **40** (0.070 g, 0.147 mmol) and *N,N-*dimethylprop-2-yn-1-amine (0.047 mL, 0.037 g, 0.440 mmol). Purification by reversed-phase HPLC provided compound **47** as an off-white solid (0.043 g, 0.099 mmol, 68%). ^1^H NMR (599 MHz, CD_3_OD) δ 8.53 (bs, 1H), 8.44 (s, 2H), 7.32 (d, *J* = 8.3 Hz, 2H), 3.61 (s, 2H), 2.44 (s, 6H), 1.73 (h, *J* = 6.8 Hz, 1H), 1.11 (d, *J* = 6.6 Hz, 3H), 0.80 (d, *J* = 6.8 Hz, 6H) ppm. ^13^C NMR (151 MHz, CD_3_OD) δ 162.96 (dd, *J* = 18.0, 6.8 Hz), 161.31 (dd, *J* = 18.1, 6.8 Hz), 158.80, 155.44, 155.02, 147.99, 128.64 (t, *J* = 12.3 Hz), 116.28 (dt, *J* = 24.2, 3.4 Hz), 112.93–112.48 (m), 90.42, 89.00, 84.17, 57.10, 44.24, 34.73, 19.27, 18.69, 18.18 ppm. HRMS (ES^+^) calcd for C_21_H_24_N_6_F_2_Cl [M + H]^+^, 433.1714; found 433.1715.

##### *tert*-Butyl (*R*)-(4-(4-(5-Chloro-7-((3-methylbutan-2-yl)amino)-[1,2,4]triazolo[1,5-*a*]pyrimidin-6-yl)-3,5-difluorophenyl)but-3-yn-1-yl)carbamate (**48**)

General procedure C was followed using **40** (0.040 g, 0.084 mmol) and *tert*-butyl but-3-yn-1-ylcarbamate (0.043 g, 0.250 mmol). Purification via silica gel flash chromatography (Hexanes/EtOAc: 90/10 to 60/40) provided compound **48** as a yellow solid (0.043 g, 0.083 mmol, 99%). ^1^H NMR (599 MHz, CDCl_3_) δ 8.34 (s, 1H), 7.10 (d, *J* = 7.9 Hz, 2H), 6.34 (s, 1H), 4.85 (s, 1H), 3.40 (t, *J* = 5.9 Hz, 2H), 3.19 (s, 1H), 2.66 (t, *J* = 6.5 Hz, 2H), 1.66–1.62 (m, 1H), 1.47 (s, 9H), 1.06 (d, *J* = 6.6 Hz, 3H), 0.80 (t, *J* = 7.4 Hz, 6H) ppm. ^13^C NMR (151 MHz, CDCl_3_) (selected representative peaks) δ 154.98, 146.00, 54.87, 33.71, 28.54, 21.29, 18.12 ppm. HRMS (ES^+^) calcd for C_25_H_29_N_6_F_2_ClO_2_ [M + Na]^+^, 519.2081; found 519.2083.

##### (*R*)-6-(4-(4-Aminobut-1-yn-1-yl)-2,6-difluorophenyl)-5-chloro-*N*-(3-methylbutan-2-yl)-[1,2,4]triazolo[1,5-*a*]pyrimidin-7-amine Hydrochloride **49**

General procedure D was followed using **48** (0.040 g, 0.077 mmol) to provide compound **49** (0.034 g, 0.075 mmol, 97%) as a yellow solid. ^1^H NMR (600 MHz, CD_3_OD) δ 8.87 (s, 1H), 7.37 (d, *J* = 8.3 Hz, 2H), 3.33 (bs, 1H), 3.23 (t, *J* = 6.7 Hz, 2H), 2.92 (t, *J* = 6.8 Hz, 2H), 1.77 (dq, *J* = 13.6, 6.8 Hz, 1H), 1.15 (d, *J* = 6.6 Hz, 3H), 0.81 (t, *J* = 7.3 Hz, 6H) ppm. ^13^C NMR (151 MHz, CD_3_OD) (selected representative peaks) δ 158.87, 155.47, 148.02, 128.96, 116.35 (d, *J* = 24.0 Hz), 90.13, 81.36, 57.10, 34.79, 19.30, 18.72, 18.17 ppm. HRMS (ES^+^) calcd for C_20_H_22_N_6_F_2_Cl [M + H]^+^, 419.1557; found 419.1560.

##### *tert*-Butyl (*R*)-(3-(4-(5-Chloro-7-((3-methylbutan-2-yl)amino)-[1,2,4]triazolo[1,5-*a*]pyrimidin-6-yl)-3,5-difluorophenyl)prop-2-yn-1-yl)(methyl)carbamate (**50**)

General procedure D was followed using **40** (0.050 g, 0.105 mmol) and *tert*-butyl methyl(prop-2-yn-1-yl)carbamate (0.053 g, 0.314 mmol). Purification via silica gel flash chromatography (Hexanes/EtOAc: 90/10 to 50/50) provided compound **50** as a yellow solid (0.020 g, 0.039 mmol, 37%). ^1^H NMR (600 MHz, CDCl_3_) δ 8.33 (s, 1H), 7.12 (d, *J* = 8.0 Hz, 2H), 6.35 (d, *J* = 8.1 Hz, 1H), 4.32 (bs, 2H), 3.16 (bs, 1H), 2.99 (s, 3H), 1.65–1.60 (m, 1H), 1.49 (s, 9H), 1.05 (d, *J* = 6.6 Hz, 3H), 0.80 (d, *J* = 6.9 Hz, 3H), 0.78 (d, *J* = 6.9 Hz, 3H) ppm. HRMS (ES^+^) calcd for C_25_H_30_N_6_F_2_ClO_2_ [M + H]^+^, 519.2081; found 519.2078.

##### (*R*)-5-Chloro-6-(2,6-difluoro-4-(3-(methylamino)prop-1-yn-1-yl)phenyl)-*N*-(3-methylbutan-2-yl)-[1,2,4]triazolo[1,5-*a*]pyrimidin-7-amine Hydrochloride (**51**)

General procedure D was followed using **50** (0.015 g, 0.029 mmol) to provide compound **51** (0.011 g, 0.024 mmol, 84%) as a brown solid. ^1^H NMR (600 MHz, CD_3_OD) δ 8.59 (s, 1H), 7.42 (d, *J* = 8.2 Hz, 2H), 4.23 (s, 2H), 3.31 (bs, 1H), 2.85 (s, 3H), 1.74 (dq, *J* = 13.4, 6.7 Hz, 1H), 1.11 (d, *J* = 6.5 Hz, 3H), 0.79 (d, *J* = 4.4 Hz, 6H) ppm. HRMS (ES^+^) calcd for C_20_H_22_N_6_F_2_Cl [M + H]^+^, 419.1557; found 419.1554.

##### (*R*)-5-Chloro-6-(4-(4-(dimethylamino)but-1-yn-1-yl)-2,6-difluorophenyl)-*N*-(3-methylbutan-2-yl)-[1,2,4]triazolo[1,5-*a*]pyrimidin-7-amine 2,2,2-Trifluoroacetate (**52**)

General procedure C was followed using **40** (0.029 g, 0.0612 mmol) and *N,N-*dimethylprop-2-yn-1-amine (0.022 mL, 0.018 g, 0.182 mmol). Purification by reversed-phase HPLC provided compound furnish compound **52** as a TFA salt as an off-white solid (0.010 g, 0.022 mmol, 37%). ^1^H NMR (600 MHz, CDCl_3_) δ 8.31 (s, 1H), 7.08 (d, *J* = 7.7 Hz, 2H), 6.35 (d, *J* = 8.3 Hz, 1H), 4.95 (bs, 2H), 3.16 (bs, 1H), 2.89 (t, *J* = 7.3 Hz, 2H), 2.78 (t, *J* = 7.3 Hz, 2H), 1.75–1.48 (m, 1H), 1.04 (d, *J* = 6.6 Hz, 3H), 0.89–0.68 (m, 6H) ppm. ^13^C NMR (151 MHz, CD_3_OD) (selected representative peaks) δ 153.54, 129.18, 67.28, 51.18, 32.83, 26.48, 17.43, 16.74, 16.18 ppm. HRMS (ES^+^) calcd for C_22_H_26_ClF_2_N_6_ [M + H]^+^, 447.1870; found 447.1873.

##### (*R*)-4-(4-(5-Chloro-7-((3,3-dimethylbutan-2-yl)amino)-[1,2,4]triazolo[1,5-*a*]pyrimidin-6-yl)-3,5-difluorophenyl)but-3-yn-1-ol (**53**)

General procedure C was followed using **41** (0.040 g, 0.081 mmol) and but-3-yn-1-ol (0.018 mL, 0.017 g, 0.240 mmol). Purification by reversed-phase HPLC provided compound **53** as an off-white solid (0.014 g, 0.032 mmol, 40%). ^1^H NMR (600 MHz, CD_3_OD) δ 8.46 (s, 1H), 7.28 (dd, *J* = 8.1, 1.5 Hz, 2H), 3.77 (t, *J* = 6.5 Hz, 2H), 3.31 (bs, 1H), 2.68 (t, *J* = 6.5 Hz, 2H), 1.07 (d, *J* = 6.7 Hz, 3H), 0.84 (s, 9H) ppm. ^13^C NMR (151 MHz, CDCl_3_) δ 159.85 (dd, *J* = 249.8, 7.0 Hz), 159.60 (dd, *J* = 249.8, 6.9 Hz), 157.03, 153.87, 152.67, 145.22, 126.93, 114.20 (td, *J* = 23.5, 3.2 Hz), 110.06, 90.55, 88.27, 78.86, 59.92, 57.09, 33.81, 24.85, 22.91, 15.60 ppm. ^19^F NMR (376 MHz, MeCN-*D*_3_) δ −108.51 (dd, *J* = 6.9 Hz, 6.9 Hz), −109.14 (dd, *J* = 6.8 Hz, 6.8 Hz) ppm. HRMS (ES^+^) calcd for C_21_H_23_N_5_F_2_ClO [M + H]^+^, 434.1554; found 434.1556.

##### (*R*)-5-(4-(5-Chloro-7-((3,3-dimethylbutan-2-yl)amino)-[1,2,4]triazolo[1,5-*a*]pyrimidin-6-yl)-3,5-difluorophenyl)pent-4-yn-1-ol (**54**)

General procedure C was followed using **41** (0.040 g, 0.081 mmol) and pent-4-yn-1-ol (0.023 mL, 0.021 g, 0.240 mmol). Purification by reversed-phase HPLC provided compound **54** as an off-white solid (0.036 g, 0.031 mmol, 38%). ^1^H NMR (600 MHz, CD_3_OD) δ 8.46 (s, 1H), 7.25 (dd, *J* = 8.0, 1.5 Hz, 2H), 3.72 (t, *J* = 6.2 Hz, 2H), 3.31 (bs, 1H), 2.58 (t, *J* = 7.1 Hz, 2H), 1.85 (p, *J* = 6.7 Hz, 2H), 1.08 (d, *J* = 6.7 Hz, 3H), 0.84 (s, 9H) ppm. ^13^C NMR (151 MHz, CDCl_3_) (selected representative peaks) δ 160.86 (dd, *J* = 250.1, 7.1 Hz), 160.61 (dd, *J* = 249.0, 7.1 Hz), 158.04, 153.67, 146.23, 134.55–130.43 (m), 128.51 (dd, *J* = 46.6, 12.1 Hz), 115.07, 94.36, 89.31, 78.80, 61.58, 58.07, 34.80, 31.20, 25.85, 16.61, 16.12 ppm. ^19^F NMR (376 MHz, MeCN-*D*_3_) δ −112.20 (dd, *J* = 8.1 Hz, 8.1 Hz), −112.83 (dd, *J* = 8.7 Hz, 8.1 Hz) ppm. HRMS (ES^+^) calcd for C_22_H_25_N_5_F_2_ClO [M + H]^+^, 448.1710; found 448.1711.

##### (*R*)-5-Chloro-6-(4-(3-(dimethylamino)prop-1-yn-1-yl)-2,6-difluorophenyl)-*N*-(3,3-dimethylbutan-2-yl)-[1,2,4]triazolo[1,5-*a*]pyrimidin-7-amine (**55**)

General procedure C was followed using **41** (0.070 g, 0.142 mmol) and *N,N*-dimethylprop-2-yn-1-amine (0.046 mL, 0.036 g, 0.427 mmol). Purification by reversed-phase HPLC provided compound **55** as an off-white solid (0.043 g, 0.096 mmol, 68%). ^1^H NMR (600 MHz, CD_3_OD) δ 8.50 (s, 1H), 7.33 (d, *J* = 7.3 Hz, 2H), 3.58 (bs, 2H), 3.31 (bs, 1H), 2.42 (s, 6H), 1.08 (d, *J* = 6.6 Hz, 3H), 0.83 (s, 9H) ppm. HRMS (ES^+^) calcd for C_22_H_26_N_6_F_2_Cl [M + H]^+^, 447.1870; found 447.1872.

##### *tert*-Butyl (*R*)-(4-(4-(5-Chloro-7-((3,3-dimethylbutan-2-yl)amino)-[1,2,4]triazolo[1,5-*a*]pyrimidin-6-yl)-3,5-difluorophenyl)but-3-yn-1-yl)carbamate (**56**)

General procedure C was followed, using **41** (0.040 g, 0.081 mmol) and tert-butyl but-3-yn-1-ylcarbamate (0.041 g, 0.240 mmol). Purification via silica gel flash chromatography (Hexanes/EtOAc: 90/10 to 60/40) provided compound **56** as a yellow solid (0.042 g, 0.079 mmol, 97%). ^1^H NMR (600 MHz, CDCl_3_) δ 8.40 (s, 1H), 7.11 (d, *J* = 7.7 Hz, 2H), 6.43 (bs, 1H), 4.85 (bs, 1H), 3.41 (d, *J* = 5.1 Hz, 2H), 3.14 (bs, 1H), 2.66 (t, *J* = 6.4 Hz, 2H), 1.47 (s, 9H), 1.02 (d, *J* = 6.6 Hz, 3H), 0.84 (s, 9H) ppm. HRMS (ES^+^) calculated for C_26_H_32_N_6_F_2_ClO_2_ [M + H]^+^, 533.2238; found 533.2238.

##### (*R*)-6-(4-(4-Aminobut-1-yn-1-yl)-2,6-difluorophenyl)-5-chloro-*N*-(3,3-dimethylbutan-2-yl)-[1,2,4]triazolo[1,5-*a*]pyrimidin-7-amine Hydrochloride (**57**)

General procedure D was followed using **41** (0.040 g, 0.075 mmol) to provide compound **57** (0.034 g, 0.072 mmol, 97%) as a yellow solid. ^1^H NMR (600 MHz, CD_3_OD) δ ^1^H NMR (599 MHz, CD_3_OD) δ 8.55 (s, 1H), 8.46 (s, 1H), 7.34 (d, *J* = 8.9 Hz, 2H), 3.21–3.17 (m, 2H), 2.89 (s, 2H), 1.06 (d, *J* = 6.8 Hz, 3H), 0.82 (s, 9H) ppm. ^13^C NMR (151 MHz, CD_3_OD) (selected representative peaks) δ 170.30, 162.82 (d, *J* = 51.9 Hz), 161.88–160.69 (m), 158.96, 155.57, 154.88, 148.10, 129.12, 116.40 (d, *J* = 23.9 Hz), 90.42, 81.26, 59.54, 39.47, 36.06, 26.07, 19.64, 16.17 ppm. ^19^F NMR (376 MHz, CD_3_OD) δ −110.82 (bs), −111.85 to −112.24 (m) ppm. HRMS (ES^+^) calcd for C_21_H_24_N_6_F_2_Cl [M + H]^+^, 433.1714; found 433.1716.

##### *tert*-Butyl (*R*)-(3-(4-(5-Chloro-7-((3,3-dimethylbutan-2-yl)amino)-[1,2,4]triazolo[1,5-*a*]pyrimidin-6-yl)-3,5-difluorophenyl)prop-2-yn-1-yl)(methyl)carbamate (**58**)

General procedure C was followed using **41** (0.050 g, 0.102 mmol) and *tert*-butyl methyl(prop-2-yn-1-yl)carbamate (0.052 g, 0.305 mmol). Purification via silica gel flash chromatography (Hexanes/EtOAc: 90/10 to 50/50) provided compound **58** as a yellow solid (0.043 g, 0.081 mmol, 79%). ^1^H NMR (600 MHz, CDCl_3_) δ 8.32 (s, 1H), 7.12 (d, *J* = 7.5 Hz, 2H), 6.43 (bs, 1H), 4.31 (bs, 2H), 2.98 (s, 3H), 1.49 (s, 9H), 1.47–1.45 (m, 1H), 1.01 (d, *J* = 6.7 Hz, 3H), 0.83 (s, 9H) ppm. ^13^C NMR (151 MHz, CDCl_3_) (selected representative peaks) δ 160.83 (dd, *J* = 250.5, 6.9 Hz), 160.58 (dd, *J* = 250.0, 6.1 Hz), 157.88, 154.90, 153.60, 146.16, 132.20 (d, *J* = 9.9 Hz), 128.63 (d, *J* = 12.2 Hz), 115.19 (t, *J* = 23.2 Hz), 89.29, 89.07, 80.92, 80.55, 58.09, 34.78, 33.94, 28.48, 25.80, 16.56 (d, *J* = 1.6 Hz) ppm. HRMS (ES^+^) calcd for C_26_H_32_N_6_F_2_ClO_2_ [M + H]^+^, 533.2238; found 533.2234.

##### (*R*)-5-Chloro-6-(2,6-difluoro-4-(3-(methylamino)prop-1-yn-1-yl)phenyl)-*N*-(3,3-dimethylbutan-2-yl)-[1,2,4]triazolo[1,5-*a*]pyrimidin-7-amine Hydrochloride (**59**)

General procedure D was followed using **58** (0.035 g, 0.066 mmol) to provide compound **59** (0.028 g, 0.060 mmol, 91%) as a HCl salt as a light brown solid. ^1^H NMR (600 MHz, CD_3_OD) δ 8.74 (s, 1H), 7.47 (dd, *J* = 8.4, 3.1 Hz, 2H), 4.24 (s, 2H), 3.33 (bs, 1H), 2.86 (s, 3H), 1.10 (d, *J* = 6.7 Hz, 3H), 0.84 (s, 9H) ppm. HRMS (ES^+^) calcd for C_21_H_24_N_6_F_2_Cl [M + H]^+^, 433.1714; found 433.1716.

##### (*R*)-5-Chloro-6-(4-(4-(dimethylamino)but-1-yn-1-yl)-2,6-difluorophenyl)-*N*-(3,3-dimethylbutan-2-yl)-[1,2,4]triazolo[1,5-*a*]pyrimidin-7-amine (**60**)

General procedure C was followed using **41** (0.040 g, 0.081 mmol) and *N*,*N*-dimethylbut-3-yn-1-amine (0.029 mL, 0.024 g, 0.224 mmol). Purification by reversed-phase HPLC provided compound furnish compound **60** as an off-white solid (0.023 g, 0.050 mmol, 61%). ^1^H NMR (599 MHz, CD_3_OD) δ 8.48 (s, 1H), 7.48–7.19 (m, 2H), 3.47 (t, *J* = 6.9 Hz, 2H), 3.05 (t, *J* = 6.9 Hz, 2H), 3.00 (s, 5H), 1.07 (d, *J* = 6.7 Hz, 3H), 0.83 (s, 9H) ppm. ^13^C NMR (151 MHz, CD_3_OD) δ 160.68, 159.03, 156.92, 153.63, 114.52, 114.36, 87.26, 57.57, 54.66, 41.66, 34.06, 24.07, 14.63, 14.16 ppm. ^19^F NMR (376 MHz, MeCN-*D*_3_) δ −75.26, −110.00 to −110.13 (m), −110.69 (dd, *J* = 11.0, 7.5 Hz) ppm. HRMS (ES^+^) calcd for C_23_H_28_ClF_2_N_6_ [M + H]^+^, 461.2027; found 461.2031.

### *In Silico* Studies

All molecular modeling experiments were performed using Maestro software package (Schrodinger Release 2021–1).^18^ Molecular Operating Environment (MOE)^19^ and Flare (Cresset)^20^ were used to visualize the structures and protein–ligand interaction, and acquire the images.

The X-ray crystal structures of αβ-tubulin in complex with **4** and **5** were downloaded from the Protein Data Bank (PDB ID: 5NJH and 7CLD, respectively). Both proteins were prepared with Protein Preparation Wizard. Waters and other co-crystallized molecules were removed, except for the ligand and GDP. Predicting protonation states of protein residues were calculated considering a temperature of 300 K and a pH of 7. A 15 Å docking grid (inner box, 10 Å; outer box, 20 Å) was prepared using as centroid the co-crystallized ligands. The TPD compounds were prepared considering the ionization states at pH 7 ± 2. The docking studies were performed using Glide SP precision keeping the default parameters and setting, and it was combined with molecular mechanics generalized Born surface area (MMGBSA), implemented in the Prime module from Maestro to re-score, the three output docking poses of each compound.

Molecular dynamics simulations were performed using Desmond module, employing OPLS4 force field in the explicit solvent and the TIP3 water model. The initial coordinates for the MD simulation were taken from the best docking poses obtained for each compound. A cubic water box was used for the solvation of the system, ensuring a buffer distance of 12 Å between each box side and the protein–ligand complex. The system was minimized and preequilibrated using the default parameters. A 100 ns MD simulation was performed. The protein–ligand during the MD simulations was analyzed using the Simulation Interaction Diagram tool implemented in the Desmond package.

#### QBI293 Cell and Neuronal Culture Acetyl-Tubulin and α-Tubulin Determinations

Compound-induced changes in acetylated-tubulin and α-tubulin in QBI293 cells or primary mouse or rat neurons were as previously described.^11,12^ For compound testing, QBI293 cells were plated at a density of 2 × 10^5^ cells/well in 12-well plates. The plated cells were incubated overnight, after which the medium was aspirated and fresh medium containing vehicle (0.25–0.3% DMSO) or test compound was added. Test compounds were prepared as 4–20 mM stock solutions in 100% DMSO, and these were diluted in culture medium prior to addition to the QBI293 cells. After 4 h incubation, whole-cell extracts were prepared from the QBI293 cells as described^11^ and the supernatant fraction from each sample was assessed for protein content by bicinchoninic acid (BCA) assay. The amount of acetyl-tubulin (AcTub) and α-tubulin (αTub) within the cell extract samples was quantified using specific enzyme-linked immunosorbent assays (ELISA), as previously described.^11^ Acetyl-tubulin levels were determined in a similar manner in rat or mouse cortical neuron cultures in which embryonic dissociated cortical neurons were plated at 3 × 10^5^ cells/well in poly-d-lysine-coated 12-well plates, essentially as previously described.^11^ The neurons were allowed to grow for 10 days before they were treated for 8 h with 15 nM okadaic acid in the presence or absence of test compound prepared as described above, or vehicle (0.25% DMSO). Culture homogenates were prepared for the determination of AcTub levels by ELISA as previously described.^11^

#### Determination of Plasma Pharmacokinetics of Compound **60**

Studies to determine the pharmacokinetic properties of compounds **53** and **60** were conducted at Touchstone Biosciences (Plymouth Meeting, PA). For the determination of plasma pharmacokinetics, three male CD-1 mice (2–3 months of age) were injected i.v. (tail vein) with test compound at a dose of 5 mg/kg in a solution of 10% DMSO, 40% polyethylene glycol, and 50% water, and another three male CD-1 mice received oral gavage dosing of **60** formulated as above at a dose of 20 mg/kg. Blood samples were collected from the saphenous or submandibular veins at multiple time points after dosing and collected in Greiner MiniCollect K_2_EDTA tubes. Compound levels in plasma were determined by LC-MS/MS, using protocols similar to those previously described.^16,22^

#### Assessment of Glu-Tubulin in Brain Tissue after Compound Treatment

All animal protocols were approved by the University of Pennsylvania Institutional Animal Care and Use Committee (IACUC). Groups of four CD-1 mice received two i.p. injections of compounds **53**, **60**, or **68** at 1 mg/kg or vehicle only spaced approximately 24 h apart. Compounds were diluted from 20 or 40 mM DMSO stock solutions into 30% Kolliphor EL (Sigma-Aldrich) phosphate-buffered saline, pH 7.4. After 4 h following the second injection, mice were euthanized and cortices and hippocampi were isolated from each brain and placed in 0.2 mL of ice-cold radioimmunoprecipitation assay (RIPA) buffer (50 mM Tris, 150 mM NaCl, 5 mM EDTA, 0.5% sodium deoxycholate, 1% NP-40, 0.1% sodium dodecyl sulfate (SDS), pH 8.0) containing protease inhibitor (PI) cocktail (Sigma-Aldrich), 1 mM phenylmethylsulfonyl fluoride (PMSF) (Sigma-Aldrich), and 3 μM trichostatin A (TSA) (Sigma-Aldrich). The brain samples were homogenized with a handheld battery-operated mixer and subsequently sonicated. After centrifugation at 100,000*g* for 30 min at 4 °C, supernatant samples were transferred to a new tube and the pellets were resuspended in 0.15 mL of RIPA buffer and again homogenized, sonicated, and centrifuged. The supernatant from the second centrifugation was pooled with that from the first. The combined supernatant samples were assessed for protein concentration using a BCA assay (Thermo Fisher Scientific), and equal protein amounts from each brain sample underwent separation by 10% sodium dodecyl sulfate polyacrylamide gel electrophoresis (SDS-PAGE) followed by transfer onto nitrocellulose membranes essentially as previously described.^11^ Membranes were incubated overnight in primary antibodies in blocking buffer at 4 °C, utilizing antibodies to de-tyrosinated (Glu)-tubulin, α-tubulin, and glyceraldehyde-3-phosphate dehydrogenase as previously described.^11^ Incubation with infrared dye-conjugated secondary antibodies and subsequent imaging and quantification of the blots on the Odyssey imaging system were as described.^11^

## Supporting Information Available

NMR spectra of test compounds and X-ray crystal structures of compound **53** (CCDC 2178417) (PDF)
The authors will release the atomic coordinates and experimental data upon article publication; PDB coordinates for Figure 6A (PDB)
PDB coordinates for Figure 6B (PDB)
PDB coordinates for Figure 6C (PDB)
PDB coordinates for Figure 6D (PDB)
PDB coordinates for Figure 6E (PDB)
PDB coordinates for Figure 6F (PDB)
PDB coordinates for Figure 7A (PDB)
PDB coordinates for Figure 7B (PDB)
PDB coordinates for Figure 7C (PDB)
PDB coordinates for Figure 7D (PDB)
SMILES string structures along the full data set in tabular form (CSV)

## Supplementary Material

jm2c01411_si_001.pdf

jm2c01411_si_002.pdb

jm2c01411_si_003.pdb

jm2c01411_si_004.pdb

jm2c01411_si_005.pdb

jm2c01411_si_006.pdb

jm2c01411_si_007.pdb

jm2c01411_si_008.pdb

jm2c01411_si_009.pdb

jm2c01411_si_010.pdb

jm2c01411_si_011.pdb

## Dedication

The authors dedicate this article to the late Dr. John Q. Trojanowski, Professor of Geriatric Medicine and Gerontology at University of Pennsylvania, who championed the idea that microtubule-normalizing drugs may provide benefit in tauopathies and who provided continued encouragement of the studies described here.

## Abbreviations Used

AcTub: acetylated α-tubulin
α-Tub: α-tubulin
MMGBSA: molecular mechanics generalized Born surface area
MT: microtubule
NFTs: neurofibrillary tangles
NTs: neuropil threads
OA: okadaic acid
PD: pharmacodynamic
PK: pharmacokinetic
Tg: transgenic
