# Supplementary material for: Microtubule-Stabilizing 1,2,4-Triazolo[1,5-a]pyrimidines as Candidate Therapeutics for Neurodegenerative Disease: Matched Molecular Pair Analyses and Computational Studies Reveal New Structure–Activity Insights
Source: J Med Chem. 2022 Dec 19;66(1):435–59. doi: 10.1021/acs.jmedchem.2c01411 (PMC9841533; doi:10.1021/acs.jmedchem.2c01411)
Supplement: Supplementary file 1 — jm2c01411_si_001.pdf [file jm2c01411_si_001.pdf]

Supporting Information

Microtubule-Stabilizing 1,2,4-Triazolo[1,5-*a*]pyrimidines as Candidate Therapeutics for Neurodegenerative Diseases: Matched Molecular Pair Analyses and Computational Studies Reveal New Structure-Activity Insights.

Thibault Alle,[a] Carmine Varricchio,[b] Yuemang Yao,[c] Bobby Lucero,[d] Goodwell Nzou,[c] Stefania Demuro,[a] Megan Muench,[c] Khoa D. Vuong,[a] Killian Oukoloff,[a] Anne-Sophie Cornec,[e] Karol R. Francisco,[a] Conor R. Caffrey,[a] Virginia M.-Y. Lee,[c] Amos B. Smith III,[e] Andrea Brancale,[b] Kurt R. Brunden[c,*] and Carlo Ballatore[a,*]

[a]Skaggs School of Pharmacy and Pharmaceutical Sciences, University of California, San Diego, 9500 Gilman Drive, La Jolla, CA 92093; [b]Cardiff School of Pharmacy and Pharmaceutical Sciences, Cardiff, King Edward VII Avenue, Cardiff CF103NB, UK;

[c]Center for Neurodegenerative Disease Research, Perelman School of Medicine, University of Pennsylvania, 3600 Spruce St., Philadelphia, PA 19104; [d]Department of Chemistry & Biochemistry, University of California San Diego, 9500 Gilman Drive, La Jolla, CA, 92093, USA; [e]Department of Chemistry, School of Arts and Sciences, University of Pennsylvania, 231 South 34th St., Philadelphia, PA 19104-6323

S2. Figure S1 – Evaluation of compounds **2**, **25**, **43**, and **53** in the QBI293 assay of MT stabilization.
S3. Figure S2 – Evaluation of compounds **25**, in the okadaic acid assay.
S4-S5. HeLa/QBI293 cells cytotoxicity assay of selected compounds
S6. Figure S3 – RMSD evolution of protein-ligand complexes during the simulations.
S7. Figure S4. Plasma PK of **53**
S8-S100. NMR spectra of test compounds.
S101-S108. LC/MS chromatograms of representative compounds.
S109-S123. X-ray report for compound **43**.

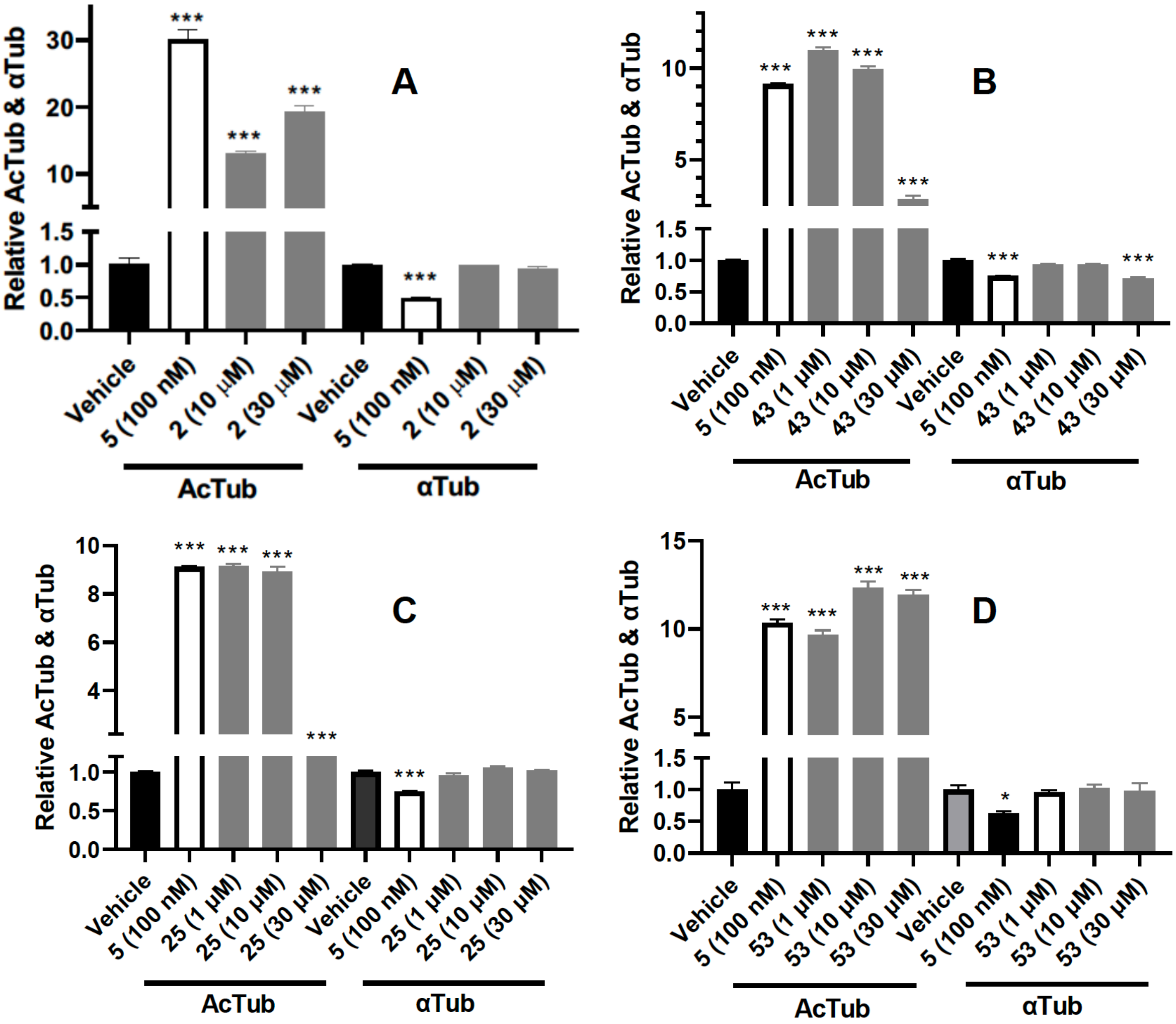

Figure S1. Assessment of AcTub and total αTub levels in QBI293 cells after 4 hour incubation with vehicle, the positive control compound, **5**, or compounds **2** (A), **43** (B), **25** (C) or **53** (D) at multiple concentrations. *, $p<0.05$ and ***, $p<0.001$ relative to vehicle as determined by one-way ANOVA.

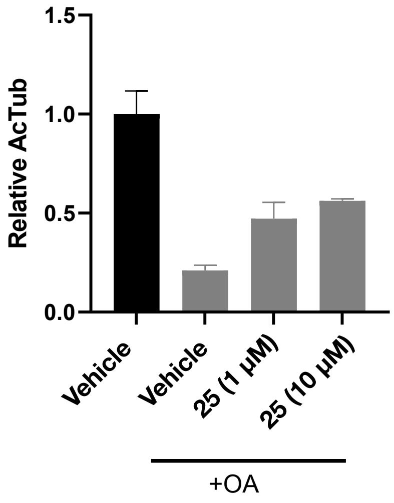

Figure S2. Assessment of AcTub levels in primary mouse cortical neurons treated with vehicle, or vehicle and compound **25** at 1 and 10 µM in presence of OA (15 nM).

Cpd #	Structure	QBI293 $CC_{50} \pm SD$ (nM)	HeLa $CC_{50} \pm SD$ (nM)
**Taxol**	-	1.29 ± 0.16	1.53 ± 0.26
**Vinblastine**	-	3.23 ± 0.67	2.11 ± 0.10
**53**	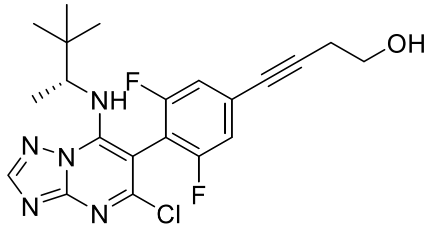	30.1 ± 4.8	30.8 ± 4.7
**64**	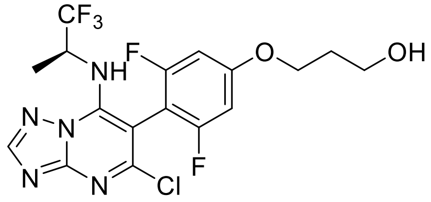	226 ± 17	189 ± 19
**68**	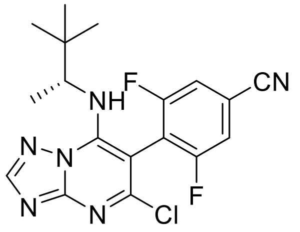	30.7 ± 5.2	41.7 ± 3.0

Cell Culture. QBI293 and HeLa cells were cultured in DMEM supplemented with 10% heat-inactivated FBS and 1% penicillin-streptomycin. Cells were grown in T175 cell culture flasks maintained at 37 °C in 5% CO_2 and sub-cultured when at 60-80% cell confluence.

Resazurin Cell Viability Assay. Cytotoxicity in QBI293 and HeLa cells was measured using the resazurin cell viability assay.[1] Test compounds were serially diluted in DMSO and added to 96-well polystyrene assay plates to give final assay concentrations ranging from 100 nM to 4000 nM (1 μL; 1% total DMSO). Fresh medium was added to the assay plate (49 μL/well). QBI293 or HeLa cells were suspended to 4 x 10^5 cells/mL in DMEM and added to each well (50 μL) for a total density of 2 x 10^4 cells/well. Assay plates were incubated at 37 °C and 5% CO_2 for 48 h, followed by addition of 20 μL 0.5 mM resazurin (Alfa Aesar, Cat. B21187) in PBS to each well. Assay plates were incubated in the dark for

4 h at 37 °C. Fluorescence was measured at 531 nm and 595 nm excitation and emission wavelengths, respectively, using a 2104 EnVision® multilabel plate reader. The viability of each well was normalized to positive and negative controls in each assay plate. Dose-response curves were generated and CC_{50} values calculated with GraphPad Prism, version 9.3 (San Diego, CA) using a sigmoidal four parameter logistic curve. The means ± standard deviations from three biological replicates are shown.

Reference

(1) O'Brien, J.; Wilson, I.; Orton, T.; Pognan, F. Investigation of the Alamar Blue (resazurin) fluorescent dye for the assessment of mammalian cell cytotoxicity. *Eur. J. Biochem.* **2000**, *267* (17), 5421-5426.

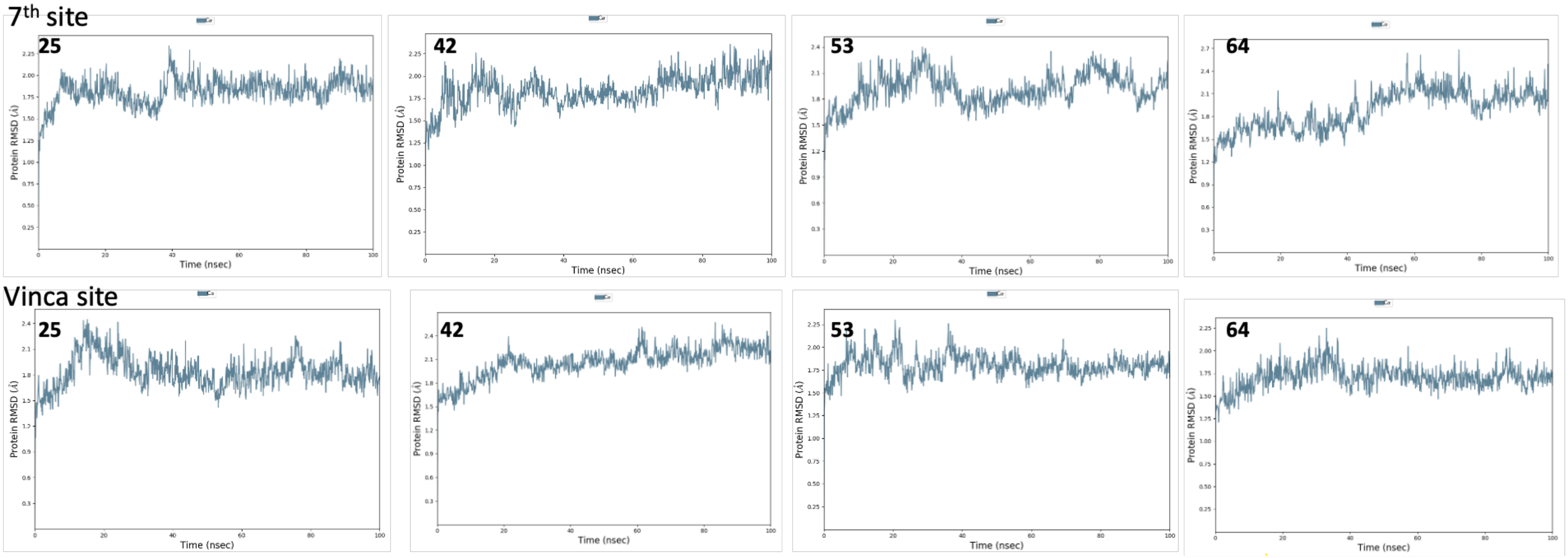

Figure S3. RMSD evolution of protein-ligand complexes during the simulations. All protein frames are first aligned at protein conformation at time 0, and then the RMSD is calculated based on the reference structure.

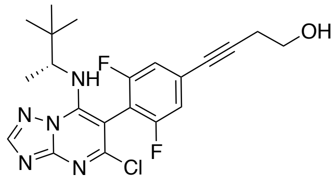

53

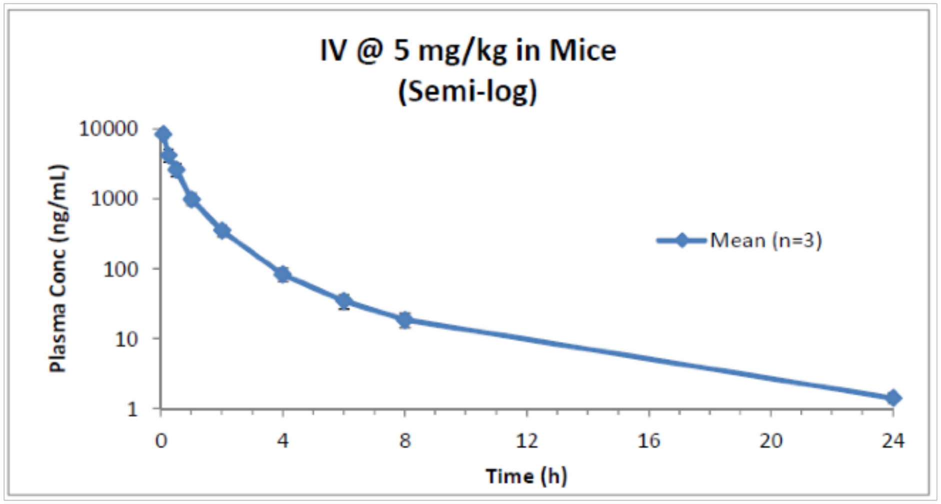

IV Administration: 5.0 mg/kg Dose Vol: 5.0 mL/kg Formulation: 10%DMSO:40%PG:50%H2O

Animal #	$t_{1/2}$ (hr)	C_0 (ng/mL)	T_{last} (hr)	C_{last} (ng/mL)	AUC_{last} (hr*ng/mL)	AUC_{inf} (hr*ng/mL)	AUC_{inf}/D (hr*mg/mL)	V_z (L/kg)	CL_p (mL/min/kg)	MRT_{inf} (hr)	V_{ss} (L/kg)
1	4.09	12798	24.0	1.14	5308	5314	1063	5.55	15.7	0.903	0.850
2	4.13	12465	24.0	1.47	5544	5553	1111	5.37	15.0	1.06	0.954
3	3.95	10387	24.0	1.64	4435	4445	889	6.42	18.7	1.33	1.50
**Mean (n=3)**	**4.06**	**11883**	**24.0**	**1.42**	**5096**	**5104**	**1021**	**5.78**	**16.5**	**1.10**	**1.10**
SD	0.092	1306	0.00	0.254	584	583	117	0.561	1.99	0.218	0.349
%CV	2.28	11.0	0.00	17.9	11.5	11.4	11.4	9.72	12.1	19.8	31.7

Figure S4. Plasma PK of **53** after administration of 5 mg/kg (i.v. injection).

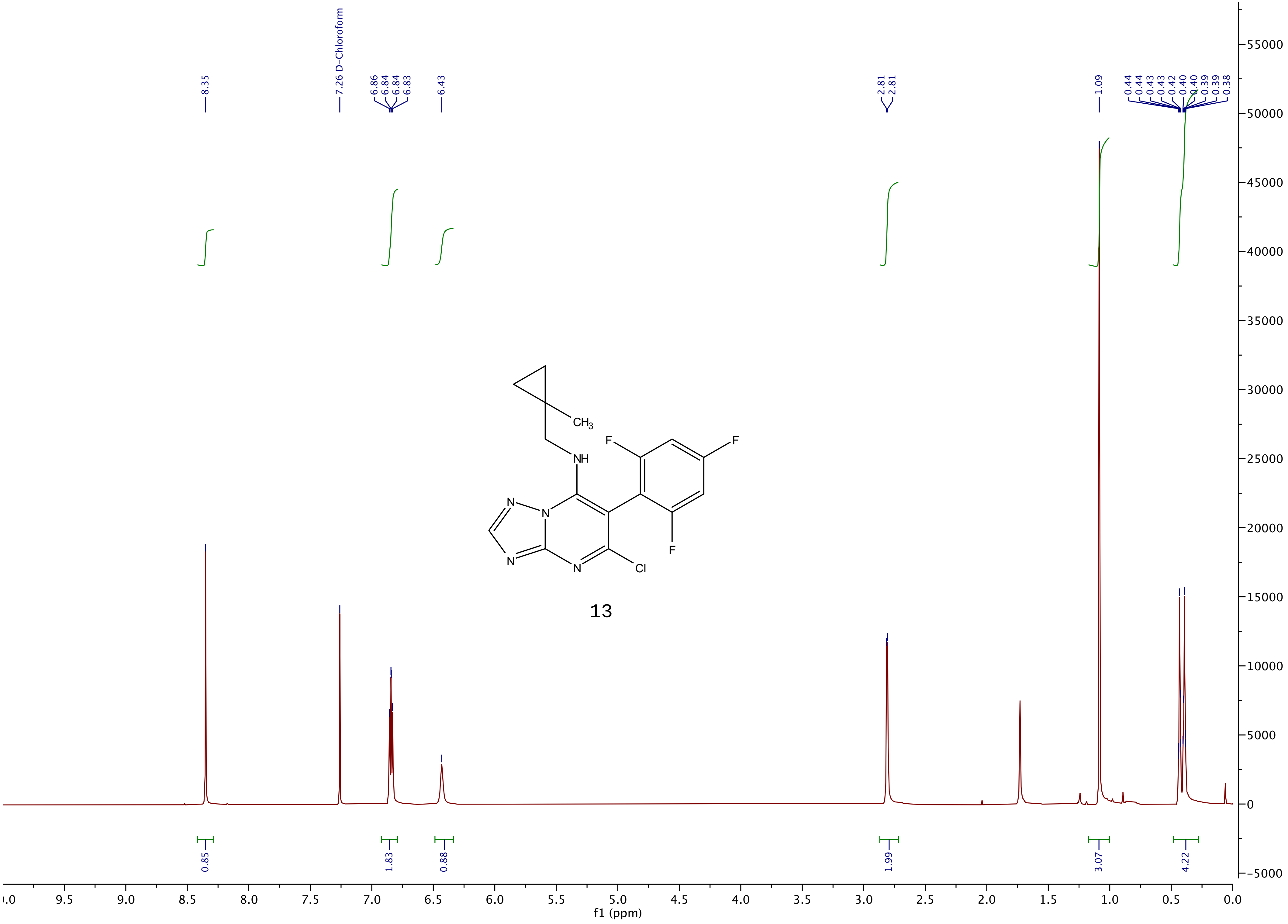
8.35
7.26 D-Chloroform
6.86
6.84
6.84
6.83
6.43
2.81
2.81
1.09
0.44
0.44
0.43
0.43
0.42
0.40
0.40
0.39
0.39
0.38
CH3
NH
F
F
F
N
N
N
N
Cl
0.85
1.83
0.88
1.99
3.07
4.22
f1 (ppm)

13

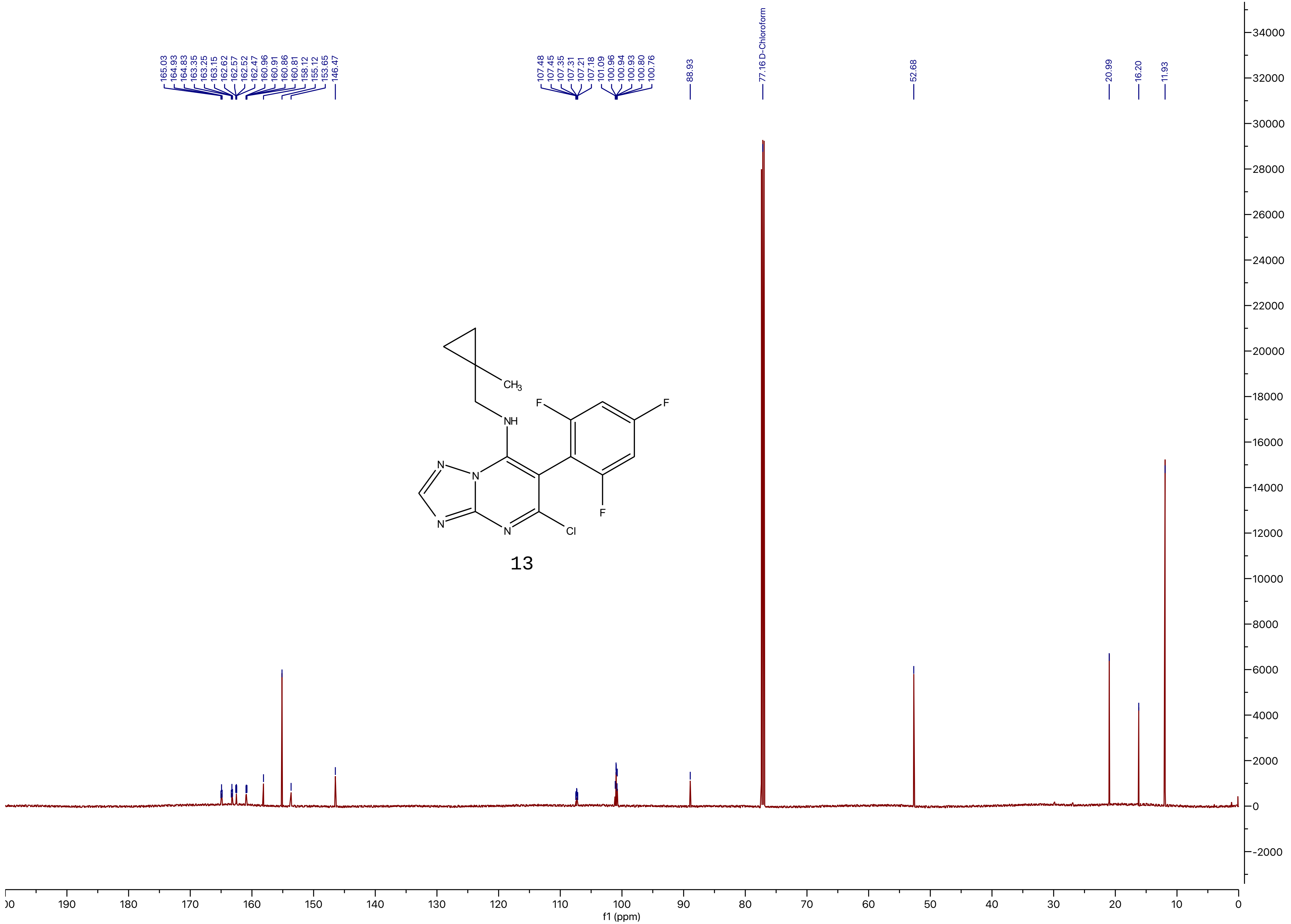
165.03
164.93
164.83
163.35
163.25
163.15
162.62
162.57
162.52
162.47
160.96
160.91
160.86
160.81
158.12
155.12
153.65
146.47
107.48
107.45
107.35
107.31
107.21
107.18
101.09
100.96
100.94
100.93
100.80
100.76
88.93
77.16 D-Chloroform
52.68
20.99
16.20
11.93
CH3
NH
F
F
F
N
N
N
N
Cl
13
f1 (ppm)
34000
32000
30000
28000
26000
24000
22000
20000
18000
16000
14000
12000
10000
8000
6000
4000
2000
0
-2000
190
180
170
160
150
140
130
120
110
100
90
80
70
60
50
40
30
20
10
0

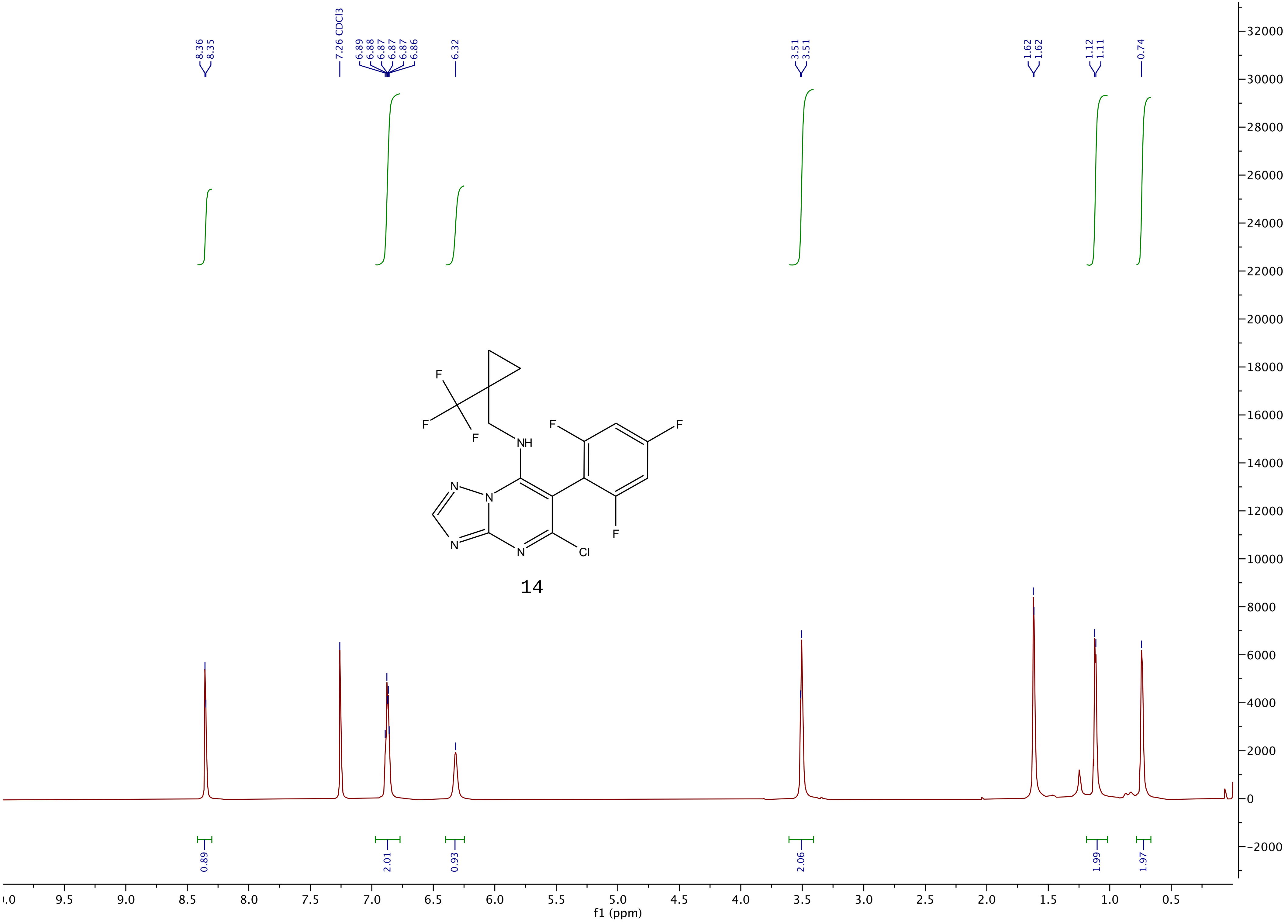
8.36
8.35
7.26 CDCl3
6.89
6.88
6.87
6.87
6.87
6.86
6.32
3.51
3.51
1.62
1.62
1.12
1.11
0.74
NH
N
N
N
N
Cl
F
F
F
F
F
F
14
0.89
2.01
0.93
2.06
1.99
1.97
f1 (ppm)

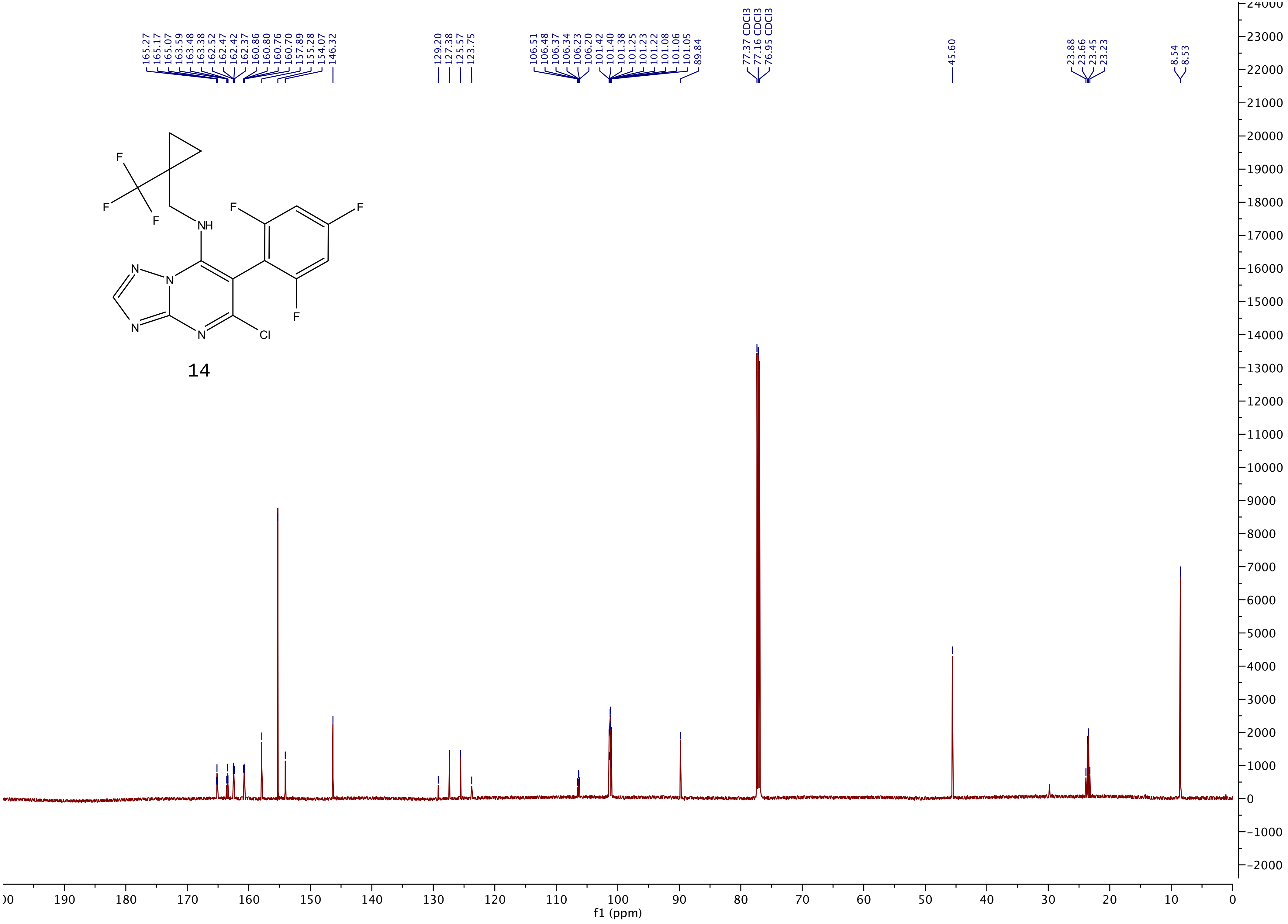
165.27
165.17
165.07
163.59
163.48
163.38
162.52
162.47
162.42
162.37
160.86
160.80
160.76
160.70
157.89
155.28
154.07
146.32
129.20
127.38
125.57
123.75
106.51
106.48
106.37
106.34
106.23
106.20
101.42
101.40
101.38
101.25
101.23
101.22
101.08
101.06
101.05
89.84
77.37 CDCl3
77.16 CDCl3
76.95 CDCl3
45.60
23.88
23.66
23.45
23.23
8.54
8.53
F
F
F
NH
F
F
N
N
N
N
Cl
F
f1 (ppm)

14

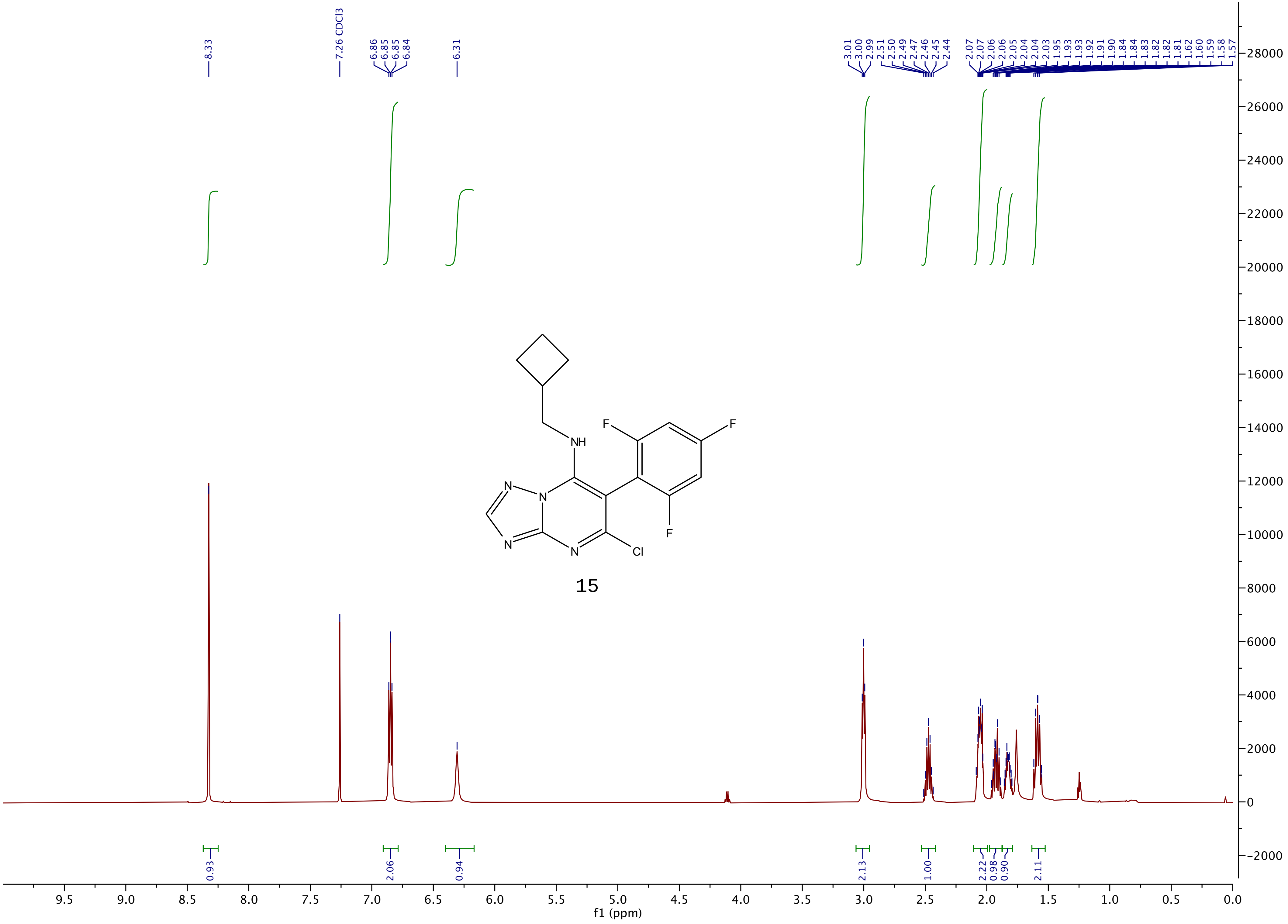
8.33
7.26 CDCl3
6.86 6.85 6.85 6.84
6.31
3.01 3.00 2.99 2.51 2.50 2.49 2.47 2.46 2.45 2.44
2.07 2.07 2.06 2.06 2.05 2.04 2.04 2.03 1.95 1.93 1.93 1.92 1.91 1.90 1.84 1.84 1.83 1.82 1.82 1.81 1.62 1.60 1.59 1.58 1.57
F
F
NH
N
N
N
N
F
Cl
15
0.93
2.06
0.94
2.13
1.00
2.22
0.98
0.90
2.11
28000
26000
24000
22000
20000
18000
16000
14000
12000
10000
8000
6000
4000
2000
0
−2000
9.5 9.0 8.5 8.0 7.5 7.0 6.5 6.0 5.5 5.0 4.5 4.0 3.5 3.0 2.5 2.0 1.5 1.0 0.5 0.0
f1 (ppm)

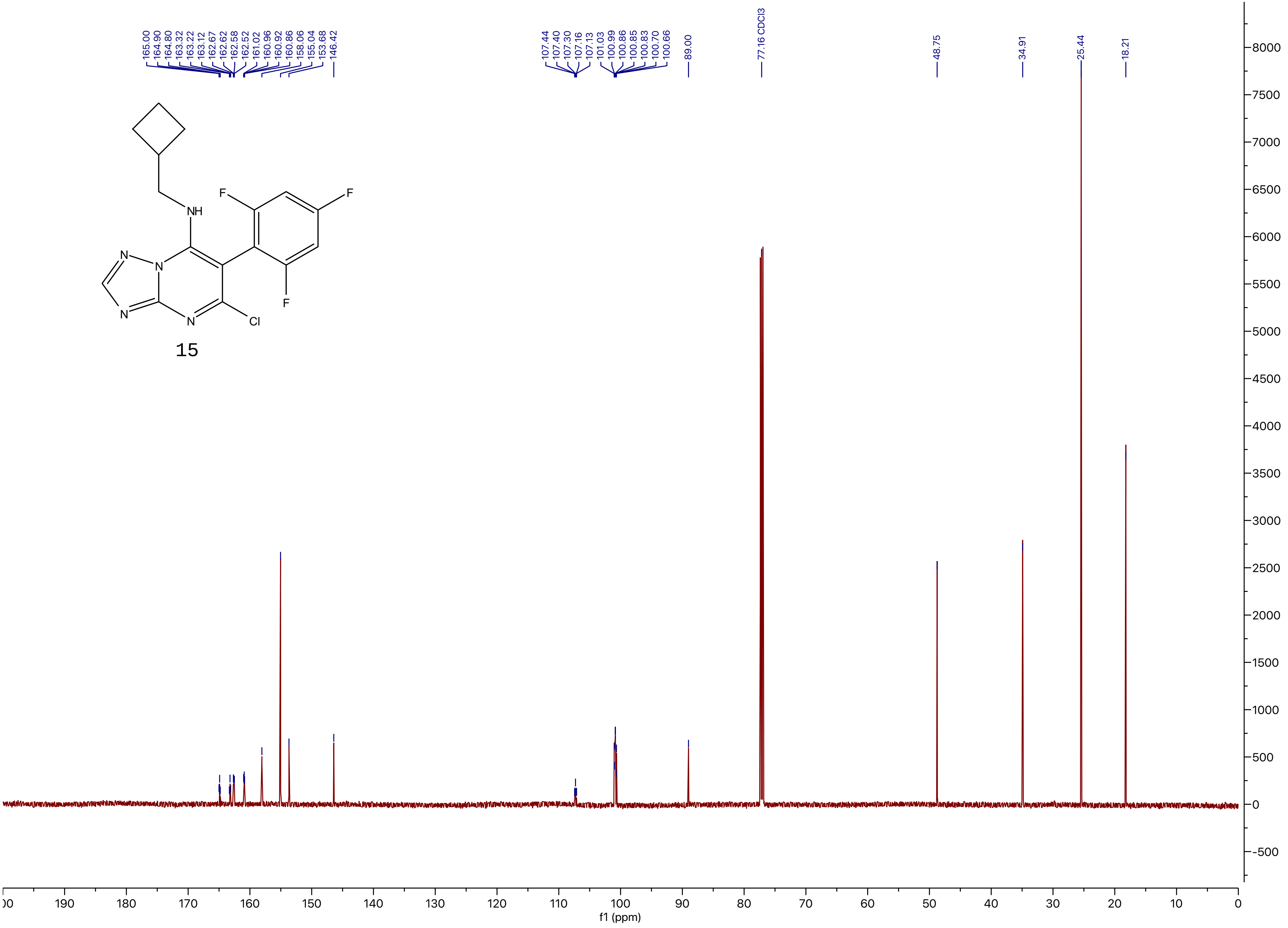
165.00
164.90
164.80
163.32
163.22
163.12
162.67
162.62
162.58
162.52
161.02
160.96
160.92
160.86
158.06
155.04
153.68
146.42
107.44
107.40
107.30
107.16
107.13
101.03
100.99
100.86
100.85
100.83
100.70
100.66
89.00
77.16 CDCl3
48.75
34.91
25.44
18.21
f1 (ppm)
NH
F
F
F
N
N
N
N
Cl

15

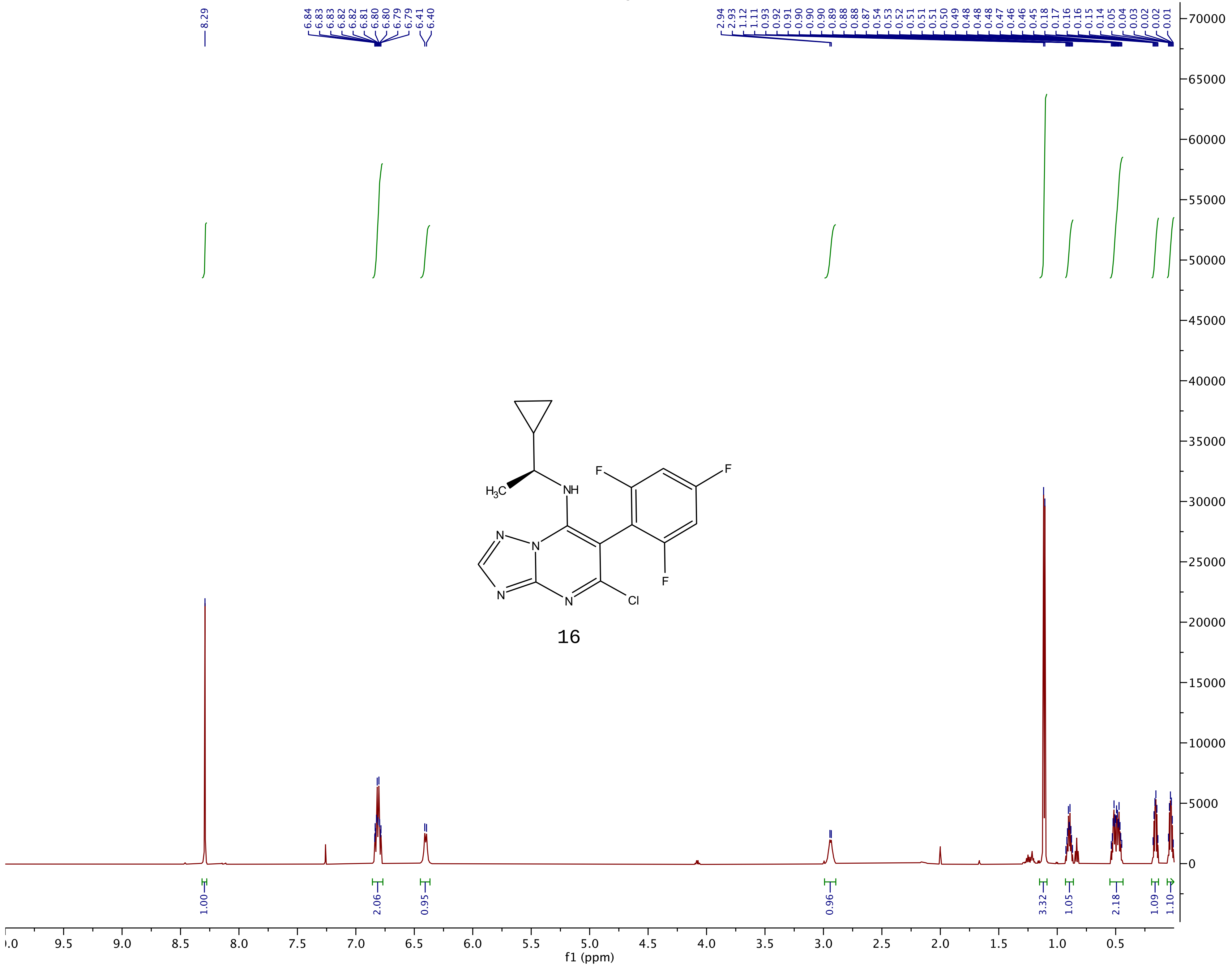

8.29
6.84
6.83
6.83
6.82
6.82
6.81
6.80
6.80
6.79
6.79
6.41
6.40
2.94
2.93
1.12
1.11
0.93
0.92
0.91
0.90
0.90
0.89
0.88
0.88
0.87
0.54
0.53
0.52
0.51
0.51
0.51
0.50
0.49
0.48
0.48
0.47
0.46
0.46
0.45
0.18
0.17
0.16
0.16
0.15
0.14
0.05
0.04
0.03
0.02
0.02
0.01
H3C
NH
F
F
F
N
N
N
N
Cl
16
1.00
2.06
0.95
0.96
3.32
1.05
2.18
1.09
1.10
f1 (ppm)

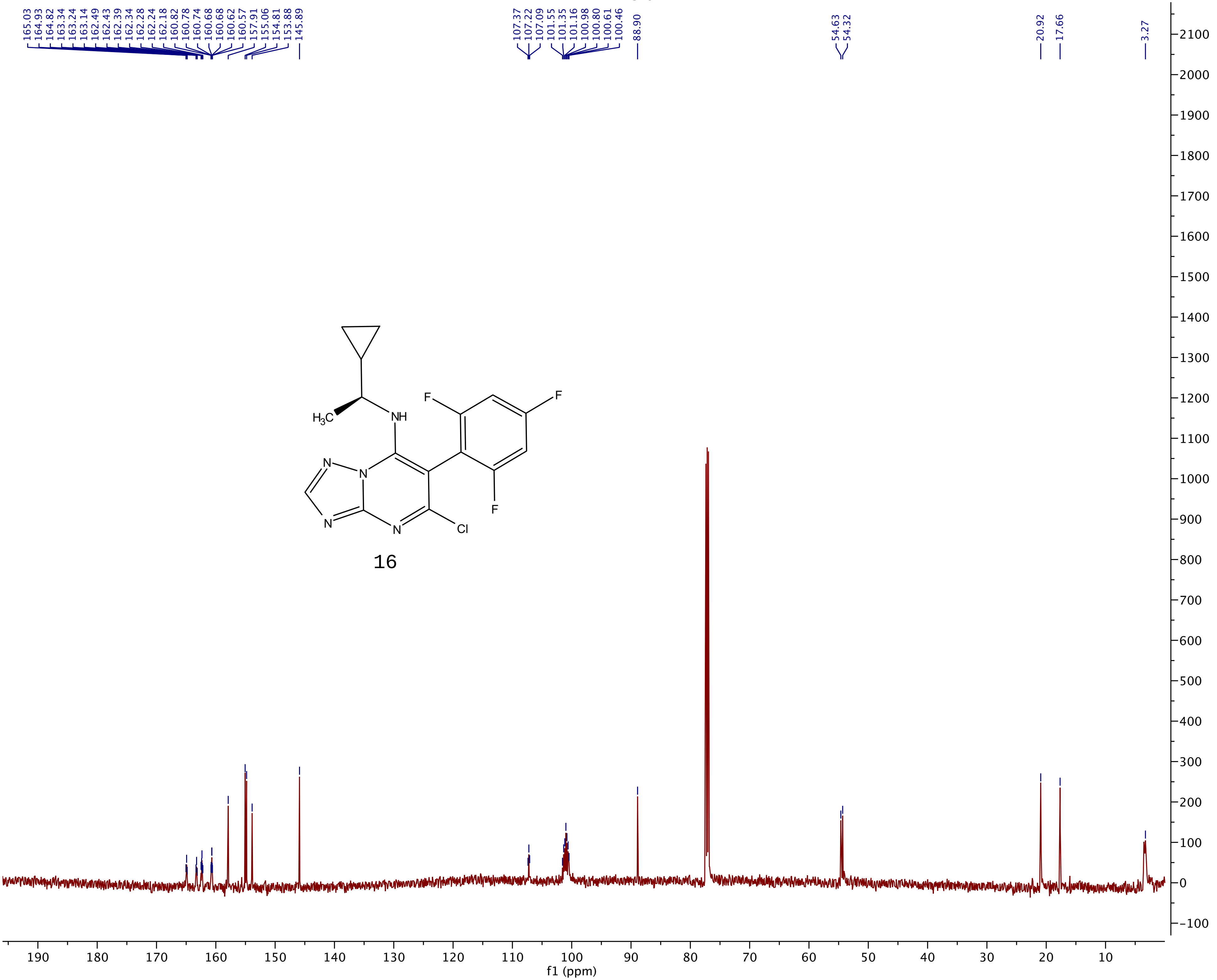
165.03
164.93
164.82
163.34
163.24
163.14
162.49
162.43
162.39
162.34
162.28
162.24
162.18
160.82
160.78
160.74
160.68
160.68
160.62
160.57
157.91
155.06
154.81
153.88
145.89
107.37
107.22
107.09
101.55
101.35
101.16
100.98
100.80
100.61
100.46
88.90
54.63
54.32
20.92
17.66
3.27
F
F
F
NH
H_3C
N
N
N
N
Cl
16
f1 (ppm)

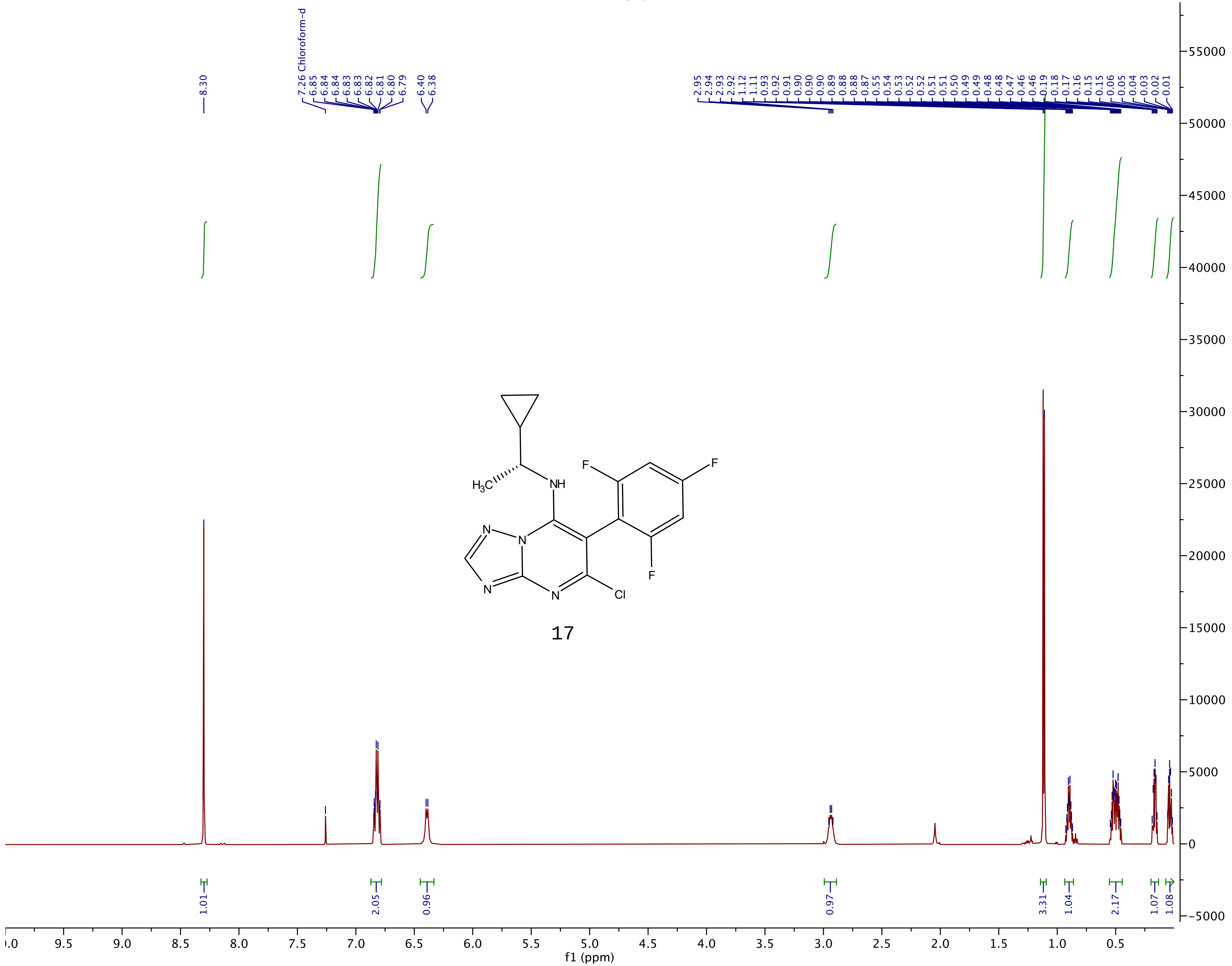

7.26 Chloroform-d
H3C
NH
N
Cl
F
17
f1 (ppm)

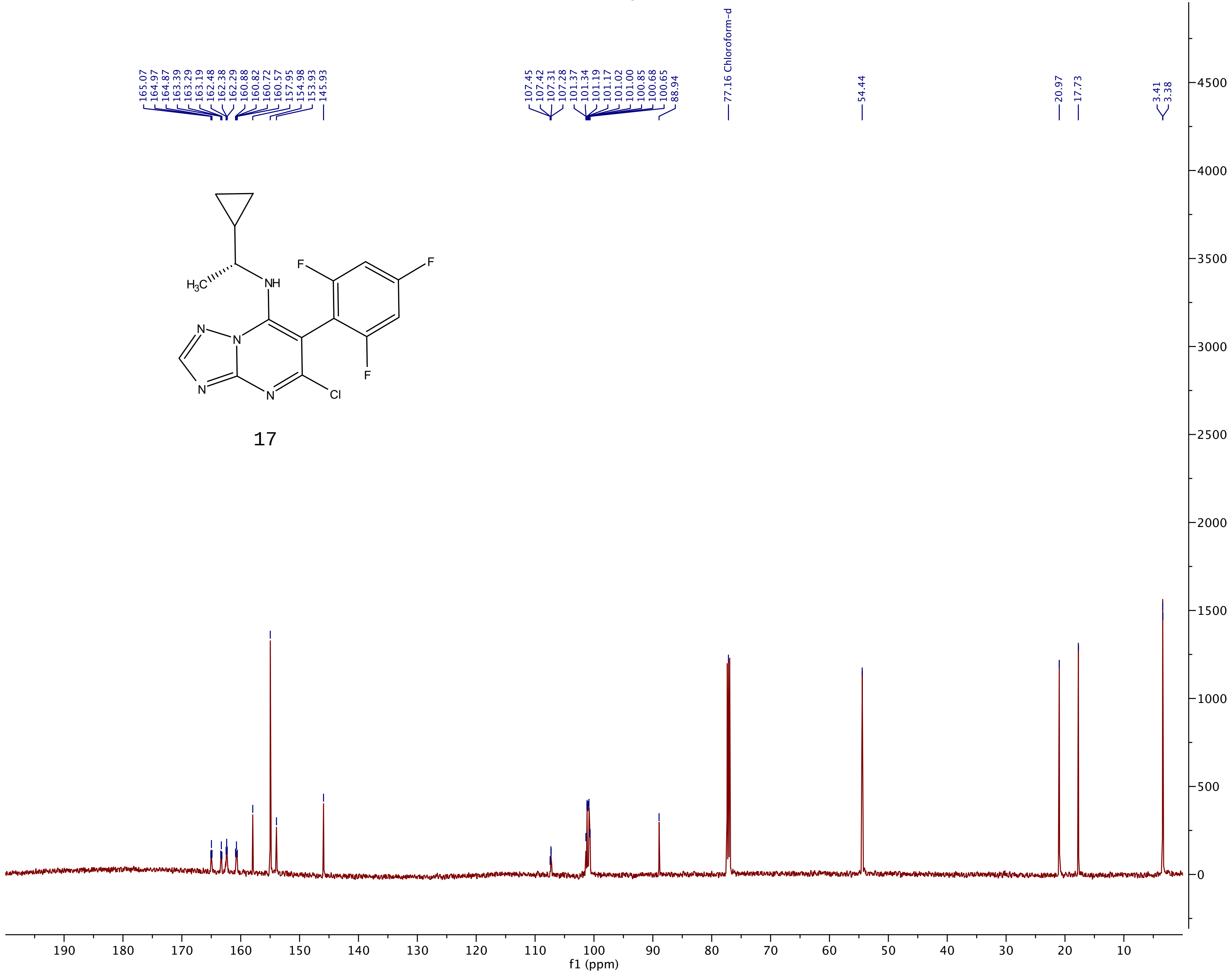
165.07
164.97
164.87
163.39
163.29
163.19
162.48
162.38
162.29
160.88
160.82
160.72
160.57
157.95
154.98
153.93
145.93
107.45
107.42
107.31
107.28
101.37
101.34
101.19
101.17
101.02
101.00
100.85
100.68
100.65
88.94
77.16 Chloroform-d
54.44
20.97
17.73
3.41
3.38
f1 (ppm)

17

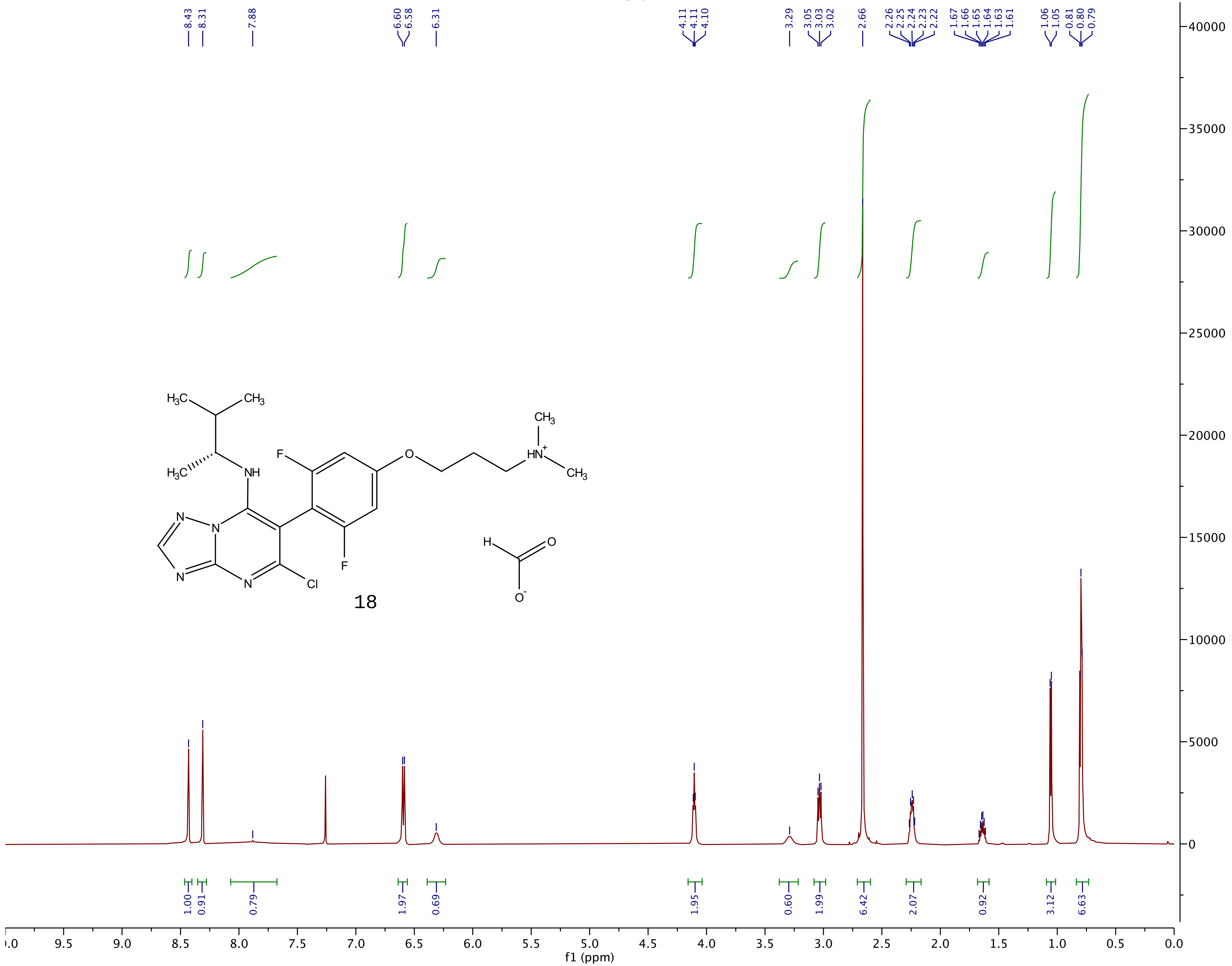
8.43
8.31
7.88
6.60
6.58
6.31
4.11
4.11
4.10
3.29
3.05
3.03
3.02
2.66
2.26
2.25
2.24
2.23
2.22
1.67
1.66
1.65
1.64
1.63
1.61
1.06
1.05
0.81
0.80
0.79
1.00
0.91
0.79
1.97
0.69
1.95
0.60
1.99
6.42
2.07
0.92
3.12
6.63
f1 (ppm)
H3C
CH3
F
O
HN+
CH3
NH
N
N
Cl
F
H
O
O-
18

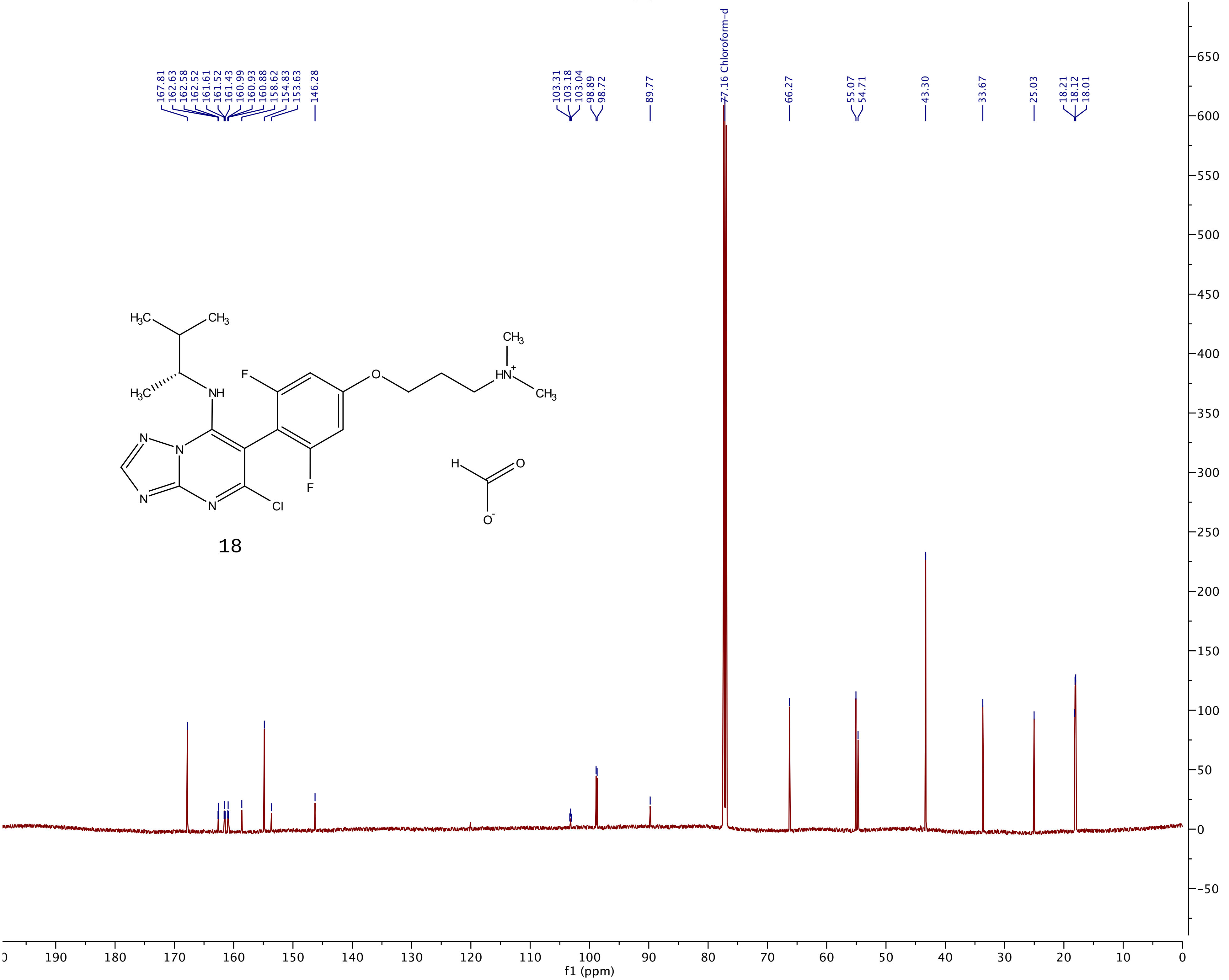

167.81
162.63
162.58
162.52
161.61
161.52
161.43
160.99
160.93
160.88
158.62
154.83
153.63
146.28
103.31
103.18
103.04
98.89
98.72
89.77
77.16 Chloroform-d
66.27
55.07
54.71
43.30
33.67
25.03
18.21
18.12
18.01
18
f1 (ppm)

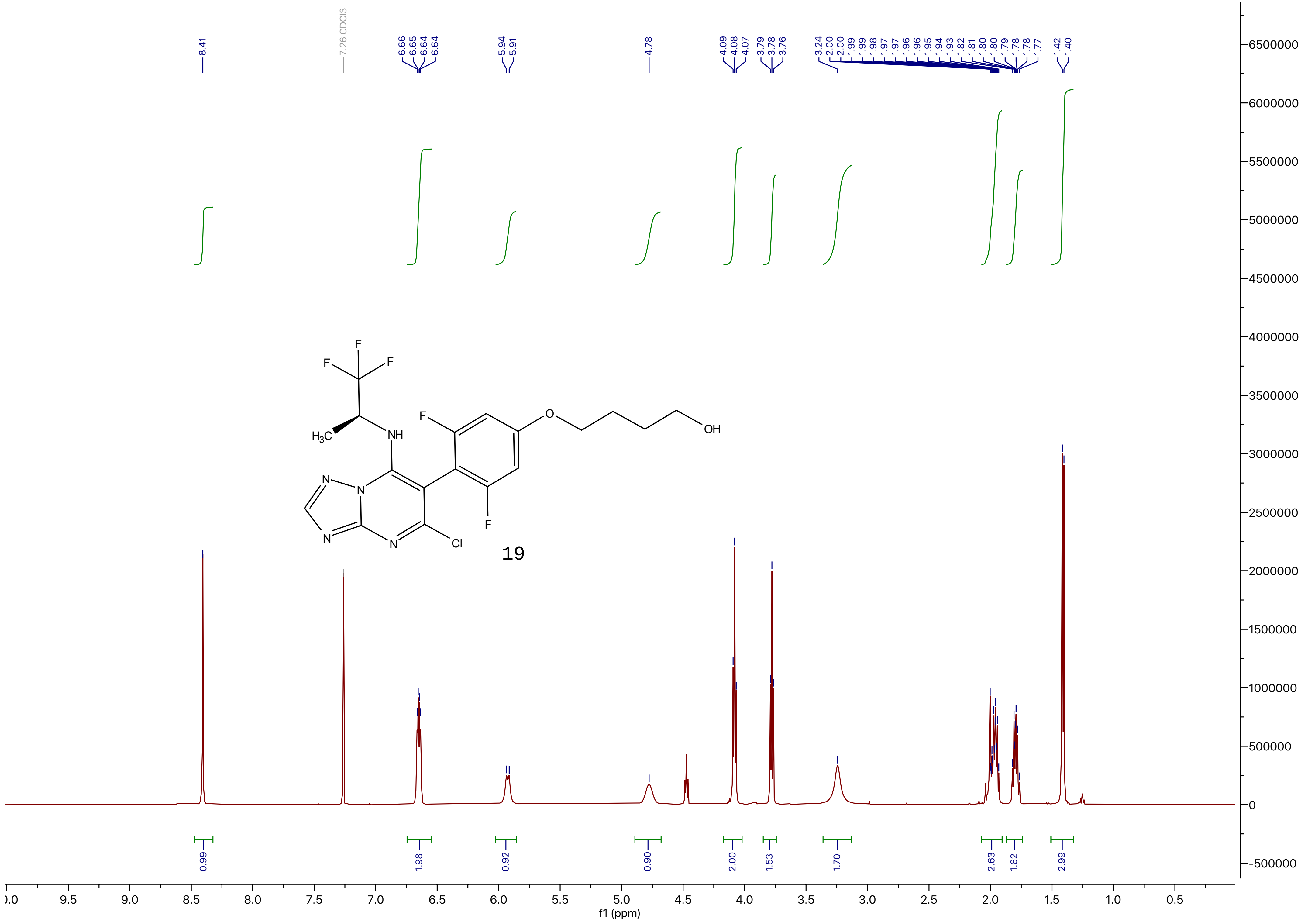
8.41
7.26 CDCl3
6.66 6.65 6.64 6.64
5.94 5.91
4.78
4.09 4.08 4.07
3.79 3.78 3.76
3.24 2.00 2.00 1.99 1.99 1.98 1.97 1.96 1.96 1.95 1.94 1.93 1.82 1.81 1.80 1.80 1.79 1.78 1.78 1.77
1.42 1.40
F
F
F
F
H3C
NH
O
OH
N
N
N
N
F
Cl
19
0.99
1.98
0.92
0.90
2.00
1.53
1.70
2.63
1.62
2.99
f1 (ppm)

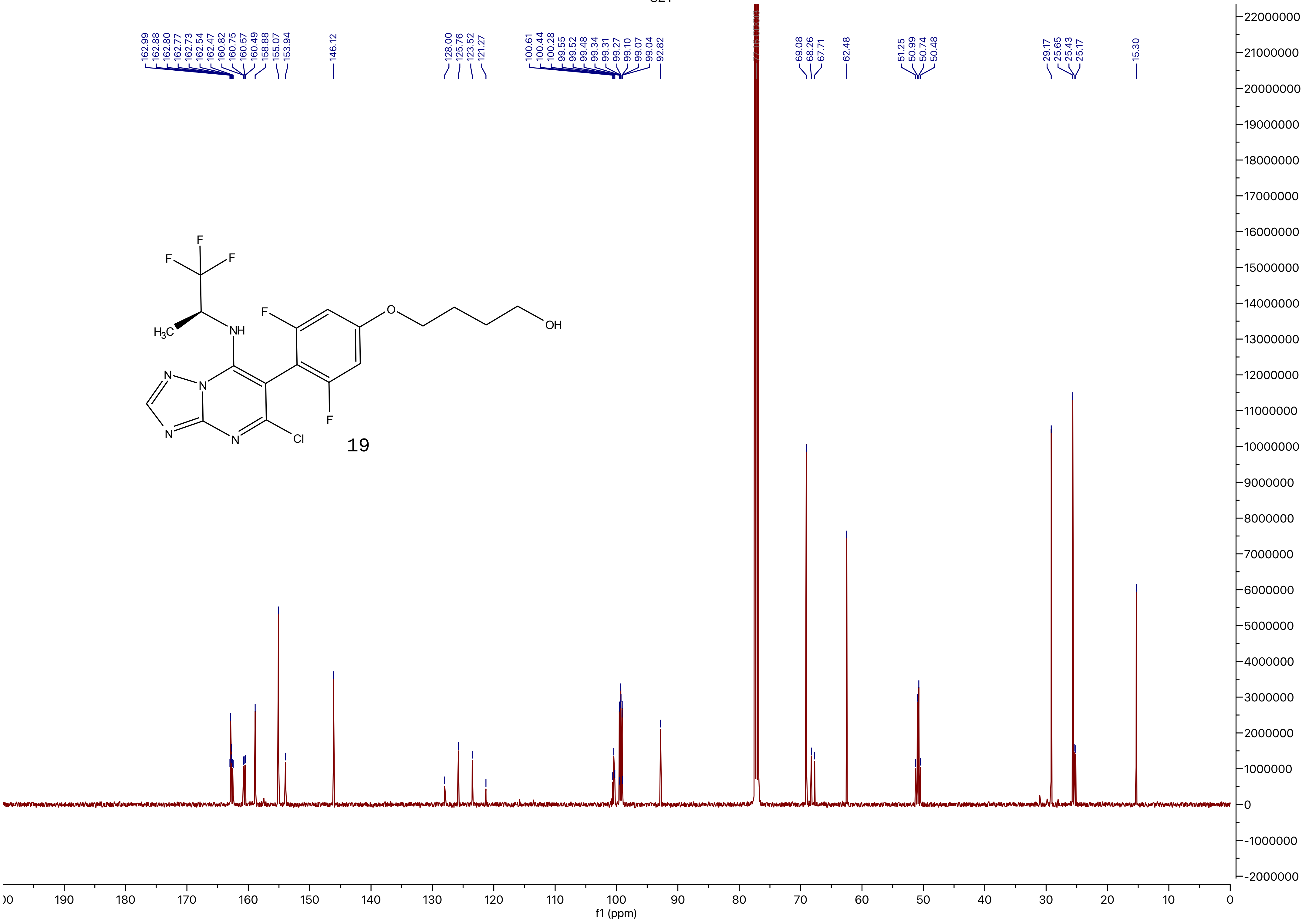
162.99
162.88
162.80
162.77
162.73
162.54
162.47
160.82
160.75
160.57
160.49
158.88
155.07
153.94
146.12
128.00
125.76
123.52
121.27
100.61
100.44
100.28
99.55
99.52
99.48
99.34
99.31
99.27
99.10
99.07
99.04
92.82
69.08
68.26
67.71
62.48
51.25
50.99
50.74
50.48
29.17
25.65
25.43
25.17
15.30
F
F
F
H3C
NH
F
O
OH
N
N
N
N
F
Cl
19
f1 (ppm)
190
180
170
160
150
140
130
120
110
100
90
80
70
60
50
40
30
20
10
0
22000000
21000000
20000000
19000000
18000000
17000000
16000000
15000000
14000000
13000000
12000000
11000000
10000000
9000000
8000000
7000000
6000000
5000000
4000000
3000000
2000000
1000000
0
-1000000
-2000000

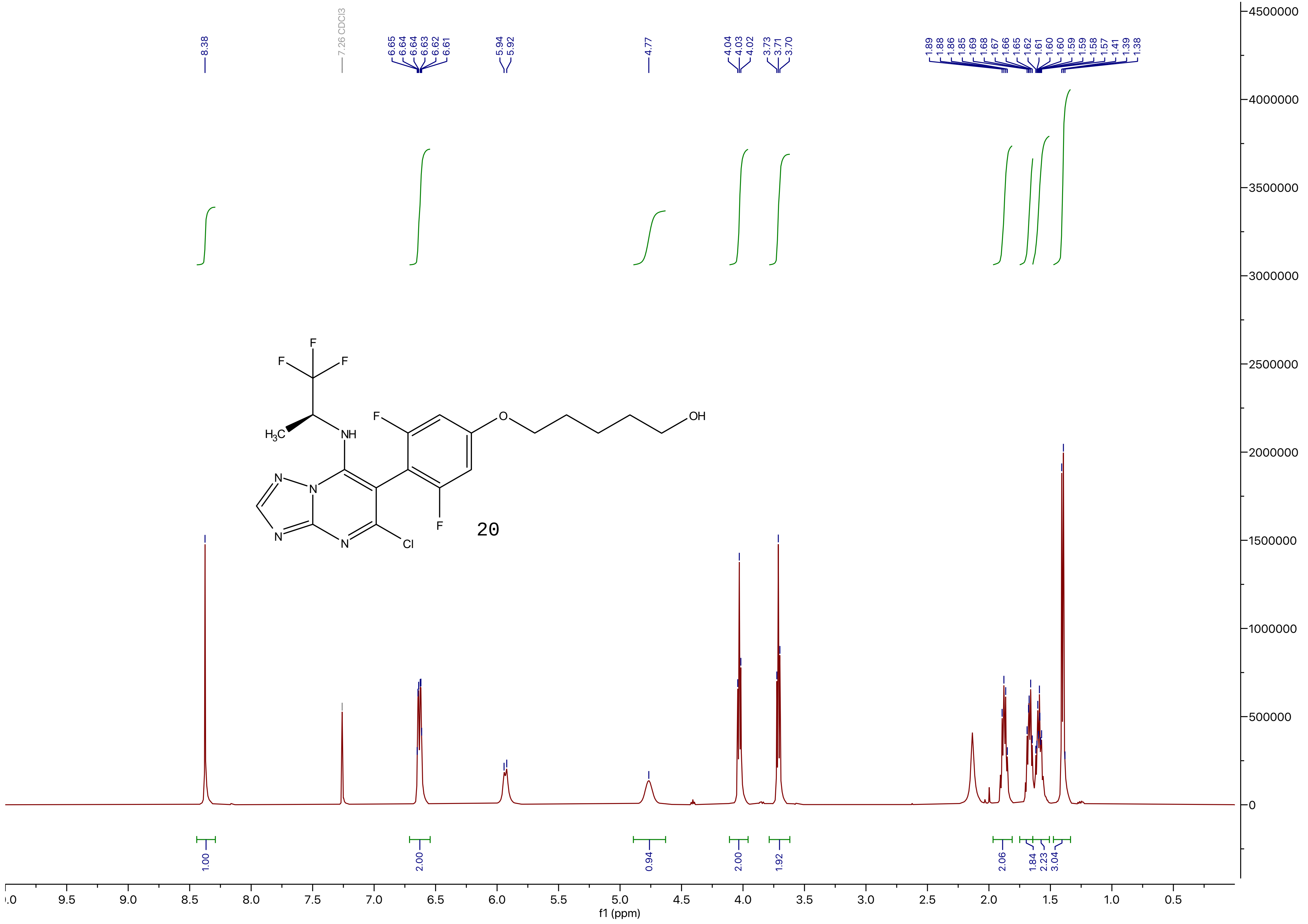
8.38
7.26 CDCl3
6.65 6.64 6.64 6.63 6.62 6.61
5.94 5.92
4.77
4.04 4.03 4.02
3.73 3.71 3.70
1.89 1.88 1.86 1.85 1.69 1.68 1.67 1.66 1.65 1.62 1.61 1.60 1.60 1.59 1.59 1.58 1.57 1.41 1.39 1.38
1.00
2.00
0.94
2.00
1.92
2.06
1.84
2.23
3.04
f1 (ppm)
F
F
F
H3C
NH
F
O
OH
N
N
N
N
Cl
F
20

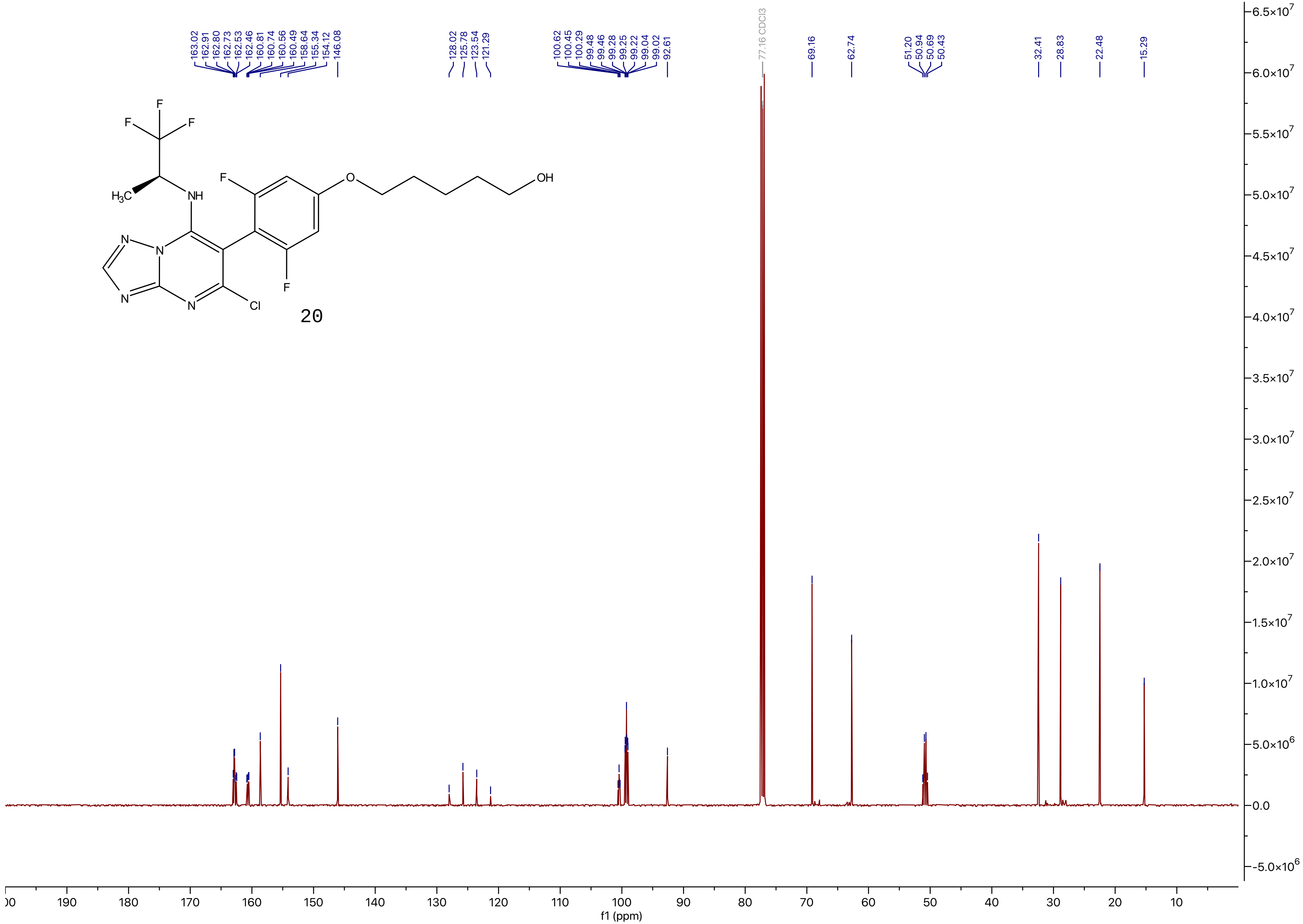
163.02
162.91
162.80
162.73
162.53
162.46
160.81
160.74
160.56
160.49
158.64
155.34
154.12
146.08
128.02
125.78
123.54
121.29
100.62
100.45
100.29
99.48
99.46
99.28
99.25
99.22
99.04
99.02
92.61
77.16 CDCl3
69.16
62.74
51.20
50.94
50.69
50.43
32.41
28.83
22.48
15.29
f1 (ppm)

20

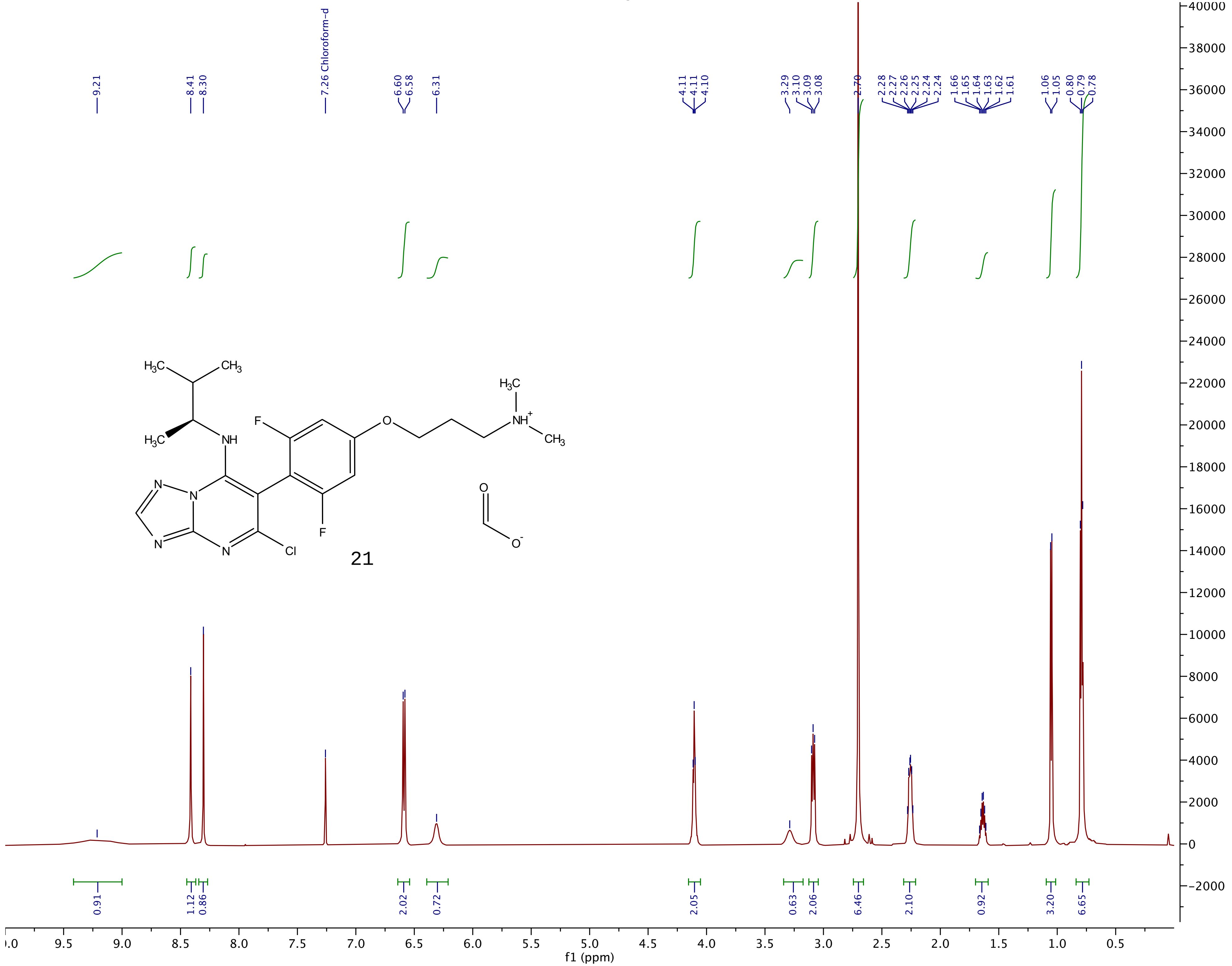
9.21
8.41
8.30
7.26 Chloroform-d
6.60
6.58
6.31
4.11
4.11
4.10
3.29
3.10
3.09
3.08
2.70
2.28
2.27
2.26
2.25
2.24
2.24
1.66
1.65
1.64
1.63
1.62
1.61
1.06
1.05
0.80
0.79
0.78
0.91
1.12
0.86
2.02
0.72
2.05
0.63
2.06
6.46
2.10
0.92
3.20
6.65
21
f1 (ppm)

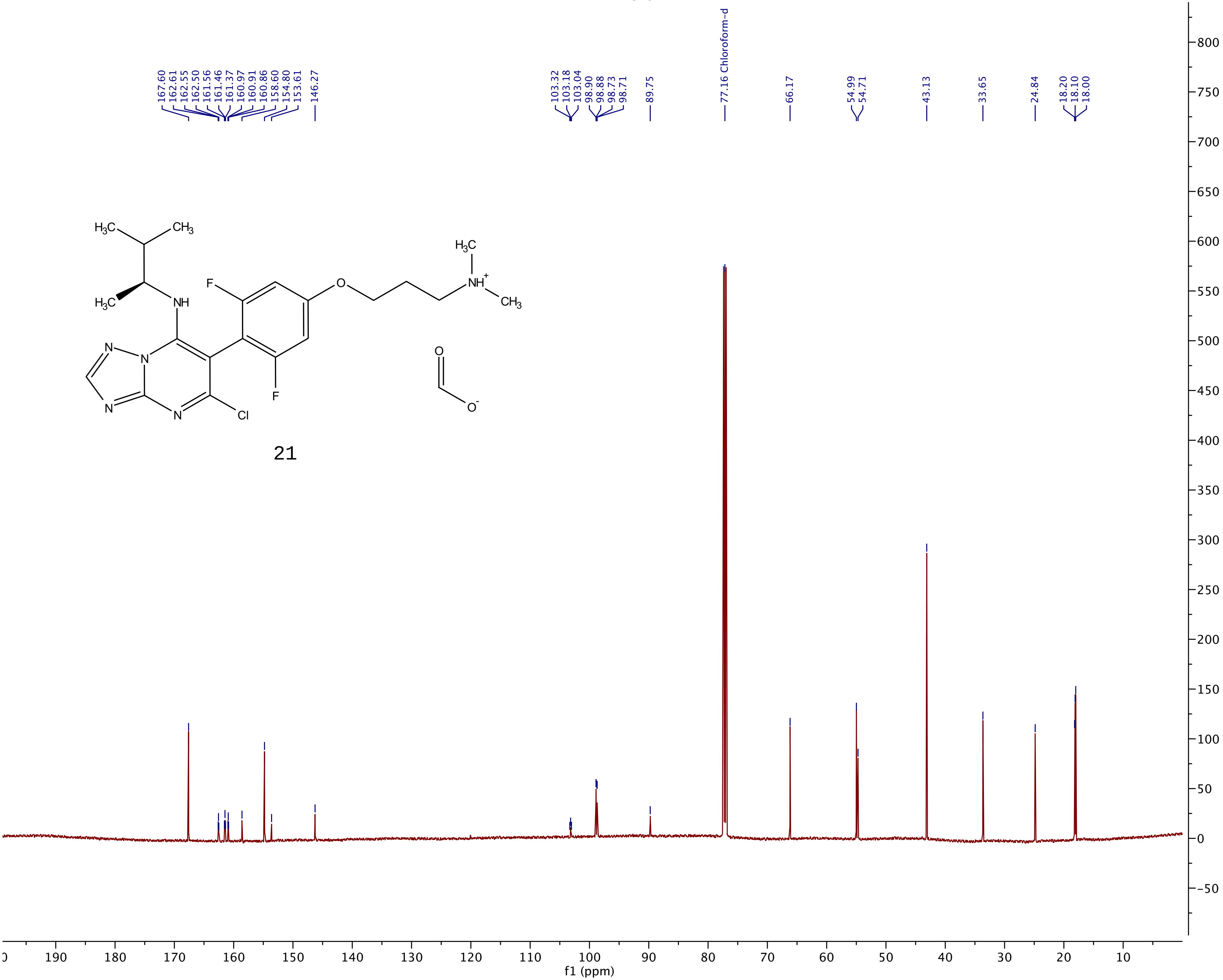
167.60
162.61
162.55
162.50
161.56
161.46
161.37
160.97
160.91
160.86
158.60
154.80
153.61
146.27
103.32
103.18
103.04
98.90
98.88
98.73
98.71
89.75
77.16 Chloroform-d
66.17
54.99
54.71
43.13
33.65
24.84
18.20
18.10
18.00
21
f1 (ppm)

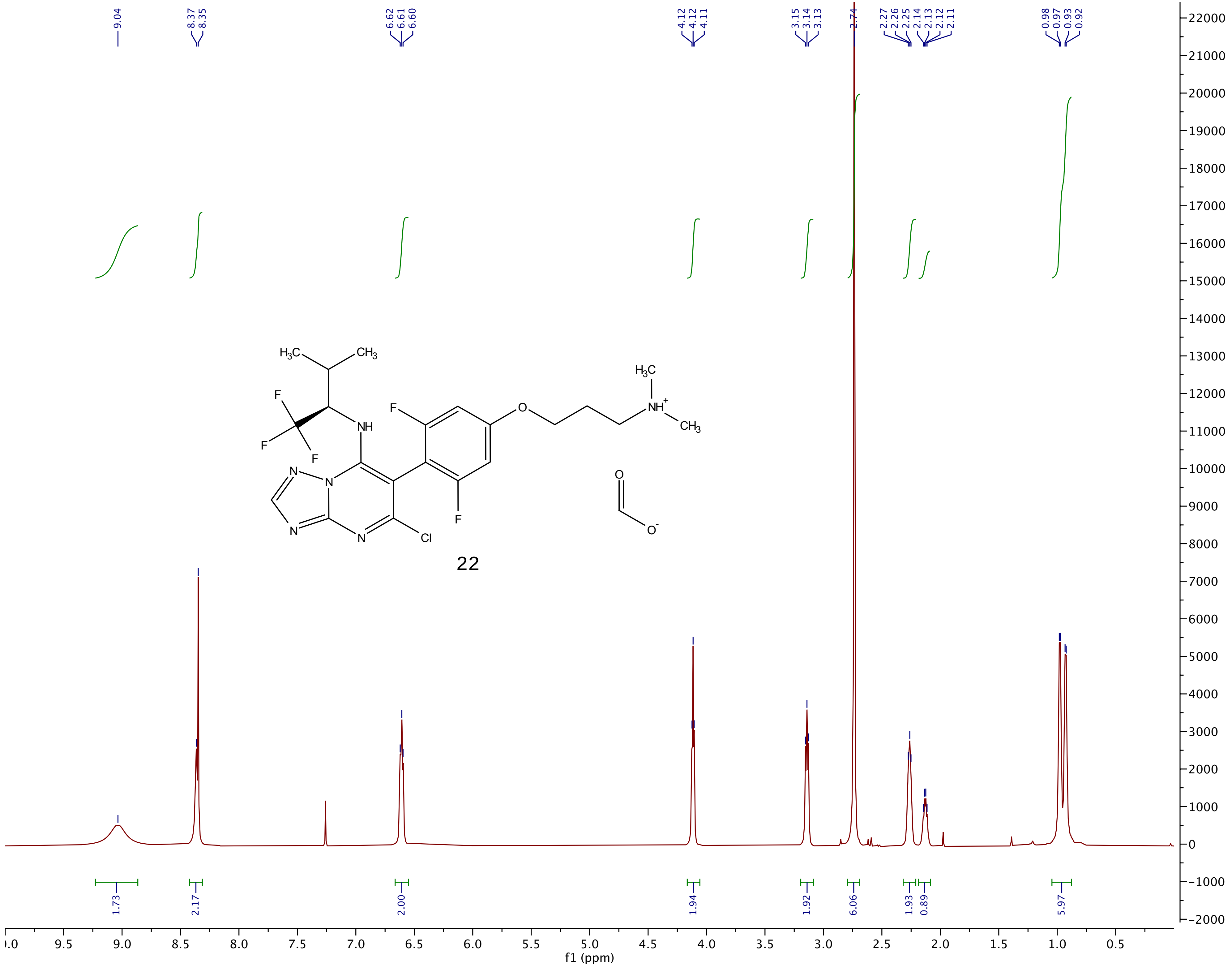
9.04
8.37
8.35
6.62
6.61
6.60
4.12
4.12
4.11
3.15
3.14
3.13
2.74
2.27
2.26
2.25
2.14
2.13
2.12
2.11
0.98
0.97
0.93
0.92
H3C
CH3
F
F
F
NH
F
O
NH+
CH3
H3C
N
N
N
N
Cl
F
O
O-
22
1.73
2.17
2.00
1.94
1.92
6.06
1.93
0.89
5.97
f1 (ppm)

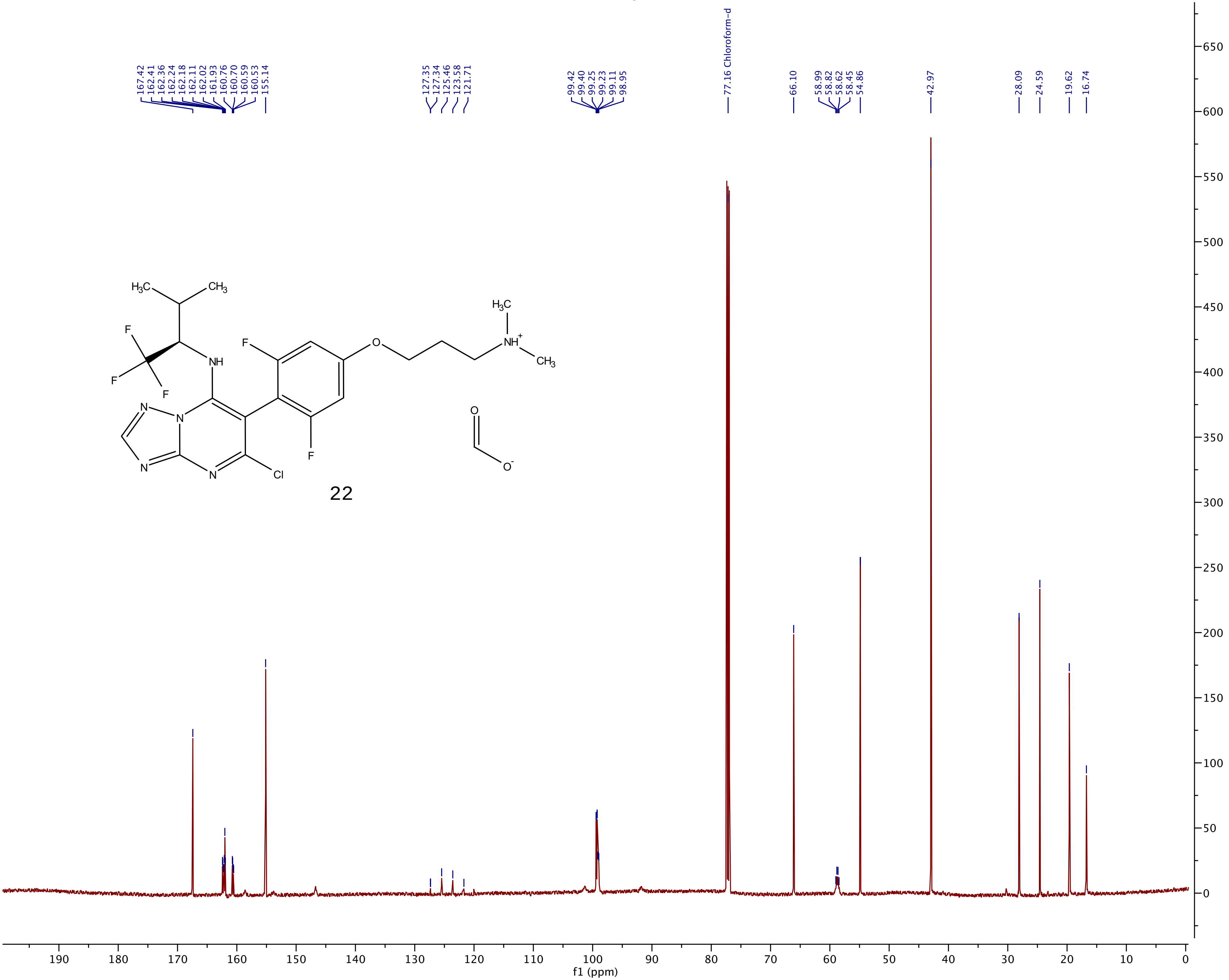
167.42
162.41
162.36
162.24
162.18
162.11
162.02
161.93
160.76
160.70
160.59
160.53
155.14
127.35
127.34
125.46
123.58
121.71
99.42
99.40
99.25
99.23
99.11
98.95
77.16 Chloroform-d
66.10
58.99
58.82
58.62
58.45
54.86
42.97
28.09
24.59
19.62
16.74
22
f1 (ppm)

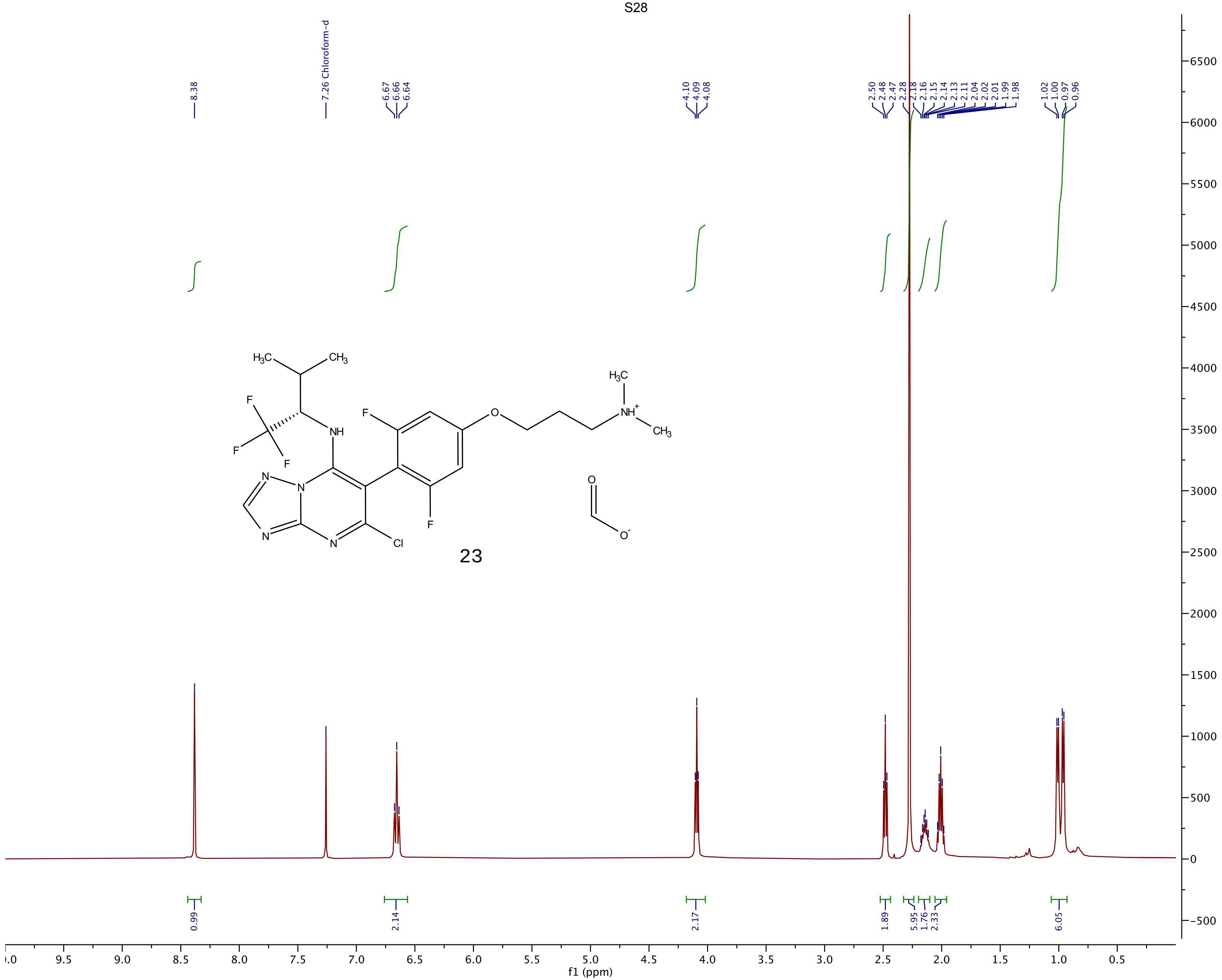
8.38
7.26 Chloroform-d
6.67
6.66
6.64
4.10
4.09
4.08
2.50
2.48
2.47
2.28
2.18
2.16
2.15
2.14
2.13
2.11
2.04
2.02
2.01
1.99
1.98
1.02
1.00
0.97
0.96
23
0.99
2.14
2.17
1.89
5.95
1.76
2.33
6.05
f1 (ppm)

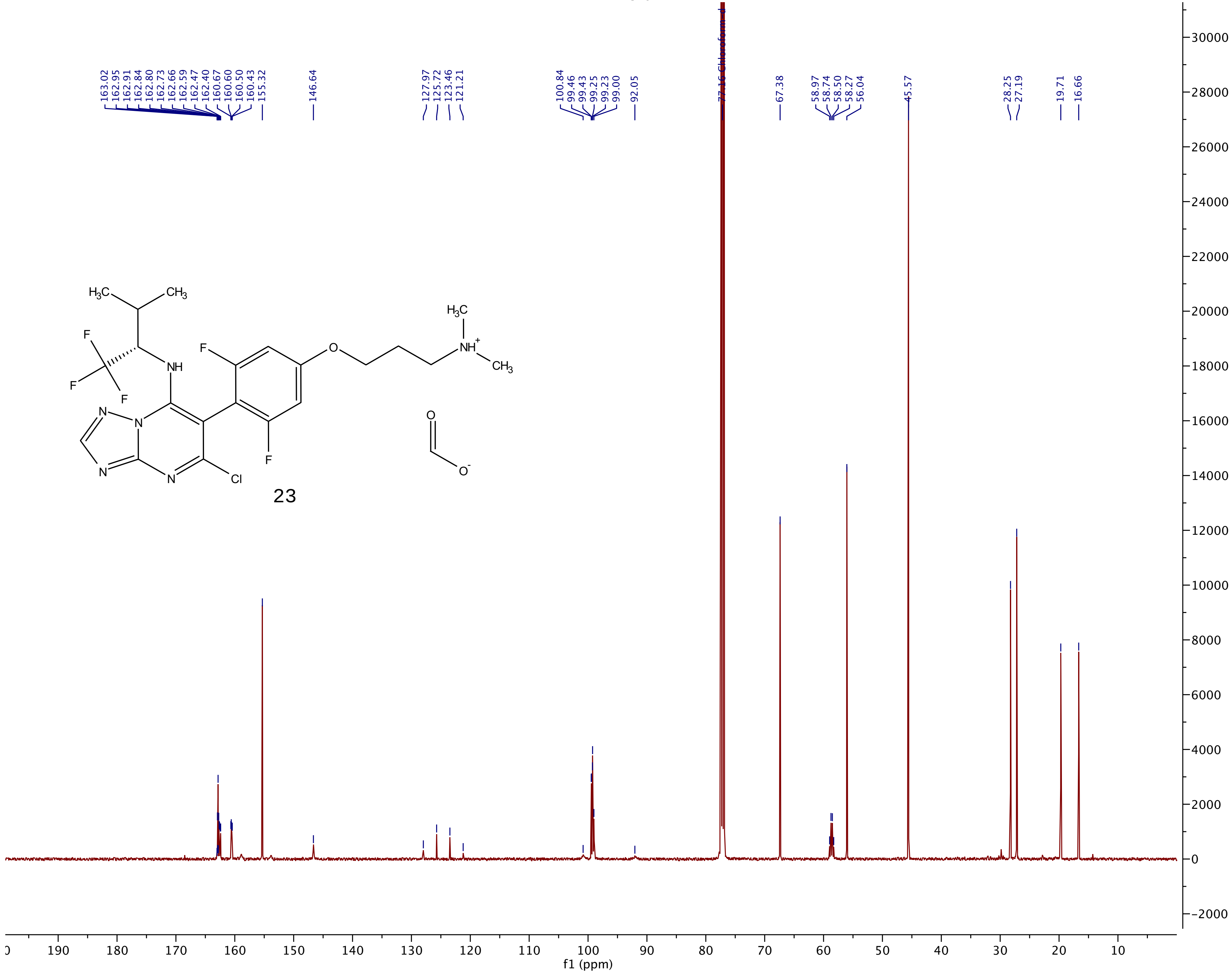

163.02
162.95
162.91
162.84
162.80
162.73
162.66
162.59
162.47
162.40
160.67
160.60
160.50
160.43
155.32
146.64
127.97
125.72
123.46
121.21
100.84
99.46
99.43
99.25
99.23
99.00
92.05
77.16 Chloroform-d
67.38
58.97
58.74
58.50
58.27
56.04
45.57
28.25
27.19
19.71
16.66
H3C
CH3
F
NH
O
NH+
CH3
N
Cl
O
O-
23
f1 (ppm)

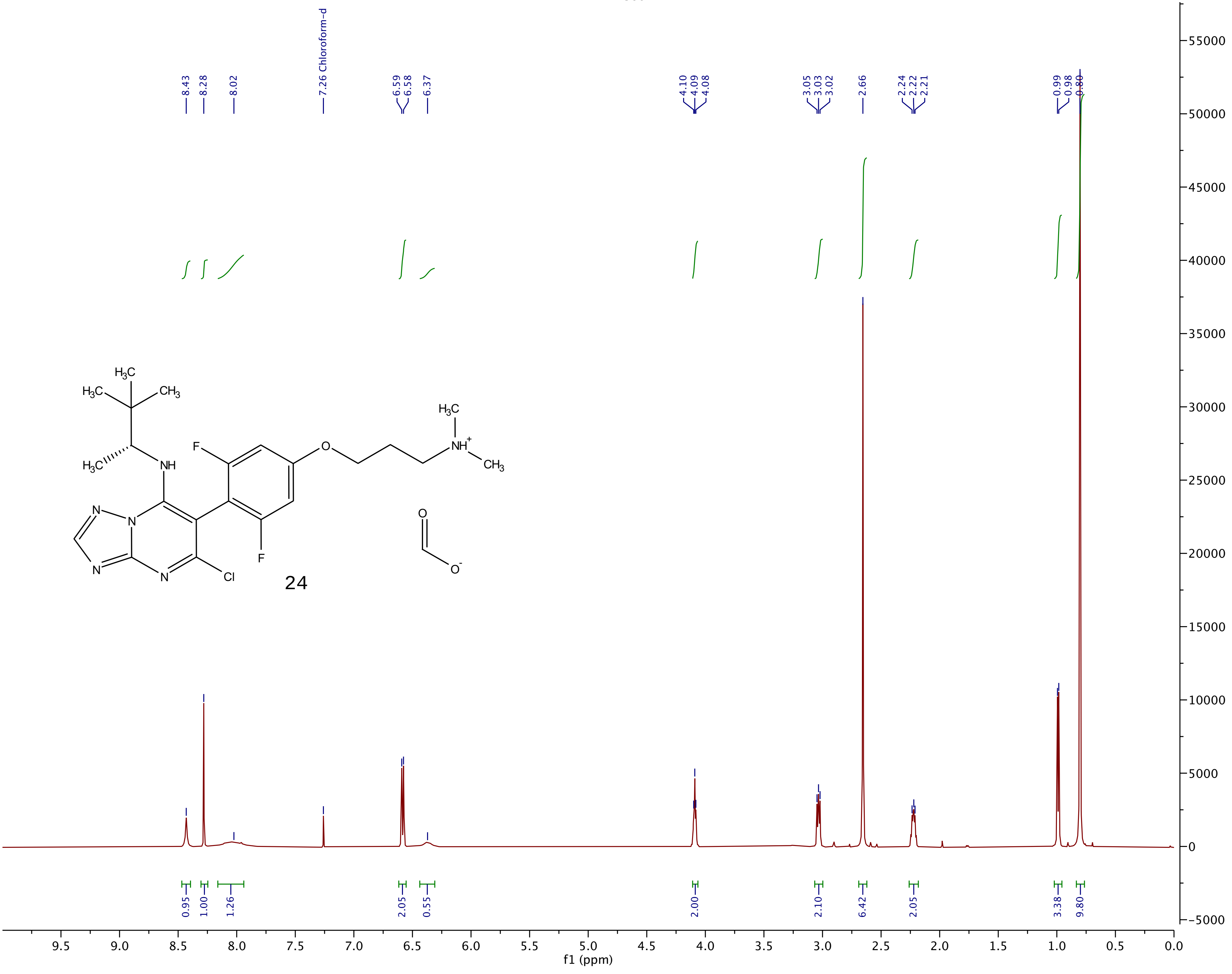

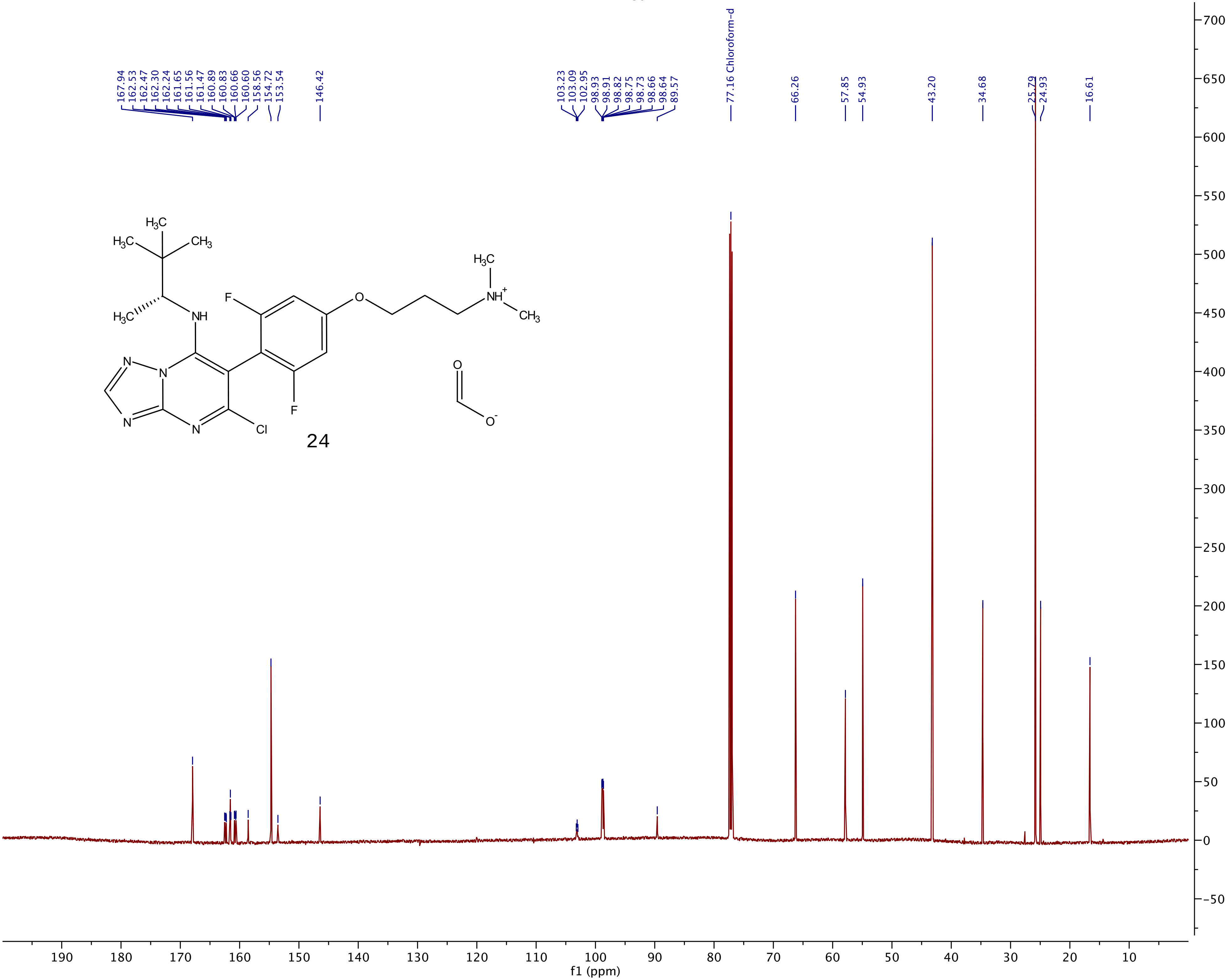
167.94
162.53
162.47
162.30
162.24
161.65
161.56
161.47
160.89
160.83
160.66
160.60
158.56
154.72
153.54
146.42
103.23
103.09
102.95
98.93
98.91
98.82
98.75
98.73
98.66
98.64
89.57
77.16 Chloroform-d
66.26
57.85
54.93
43.20
34.68
25.79
24.93
16.61
f1 (ppm)

24

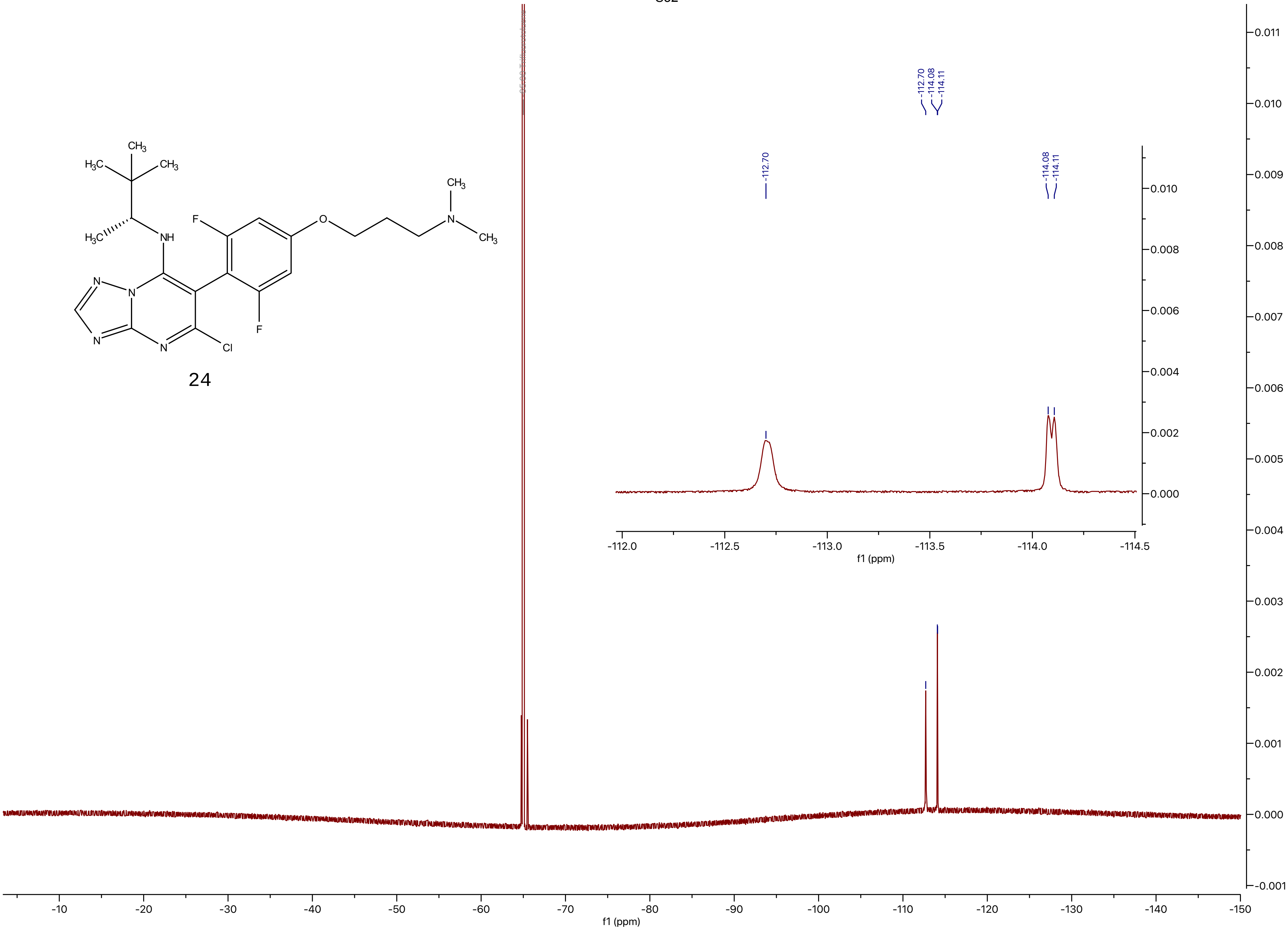

24
-112.70
-114.08
-114.11
f1 (ppm)

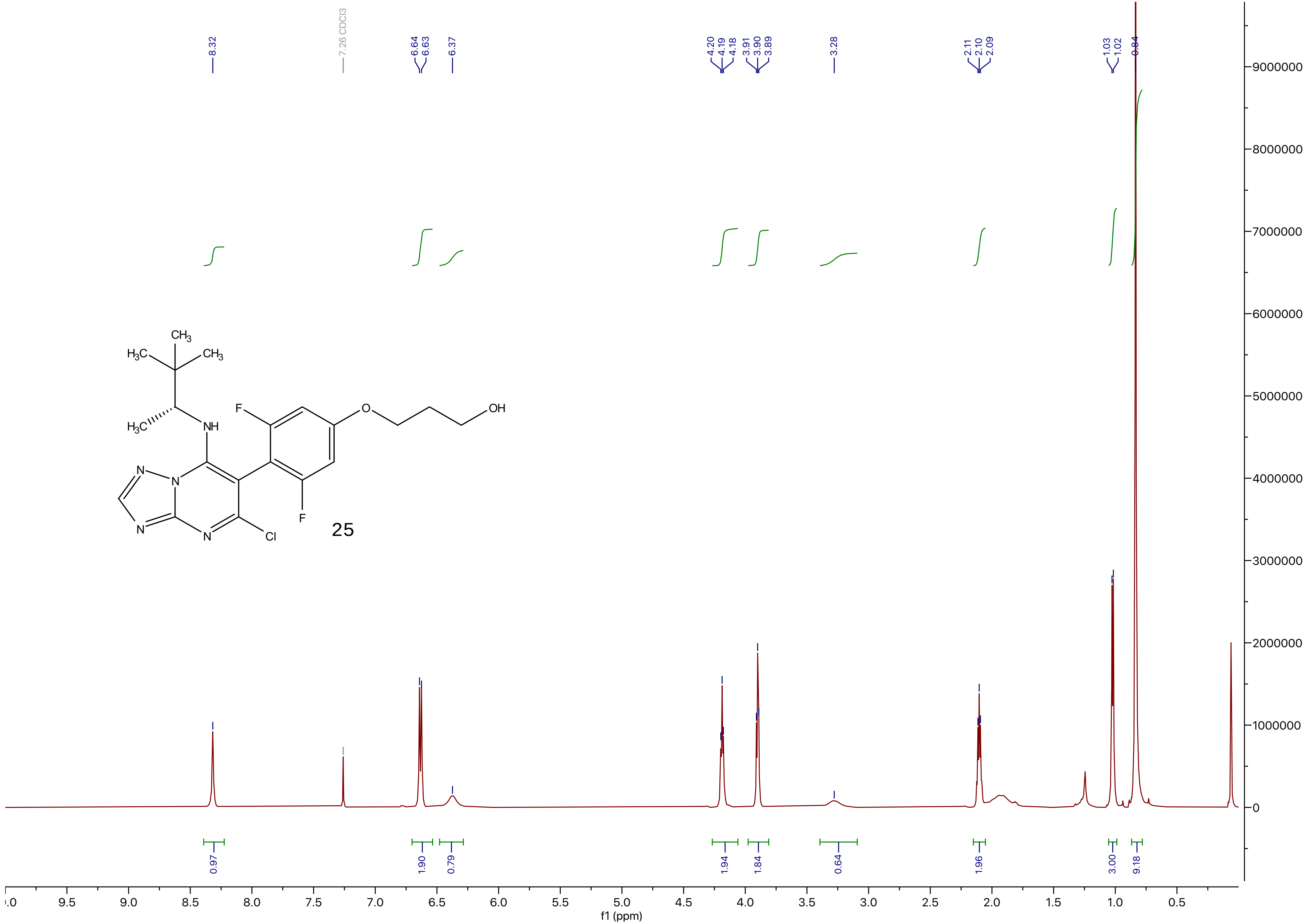
8.32
7.26 CDCl3
6.64
6.63
6.37
4.20
4.19
4.18
3.91
3.90
3.89
3.28
2.11
2.10
2.09
1.03
1.02
0.84
0.97
1.90
0.79
1.94
1.84
0.64
1.96
3.00
9.18
f1 (ppm)
CH3
H3C
CH3
F
O
OH
H3C
NH
N
N
F
N
N
Cl
25

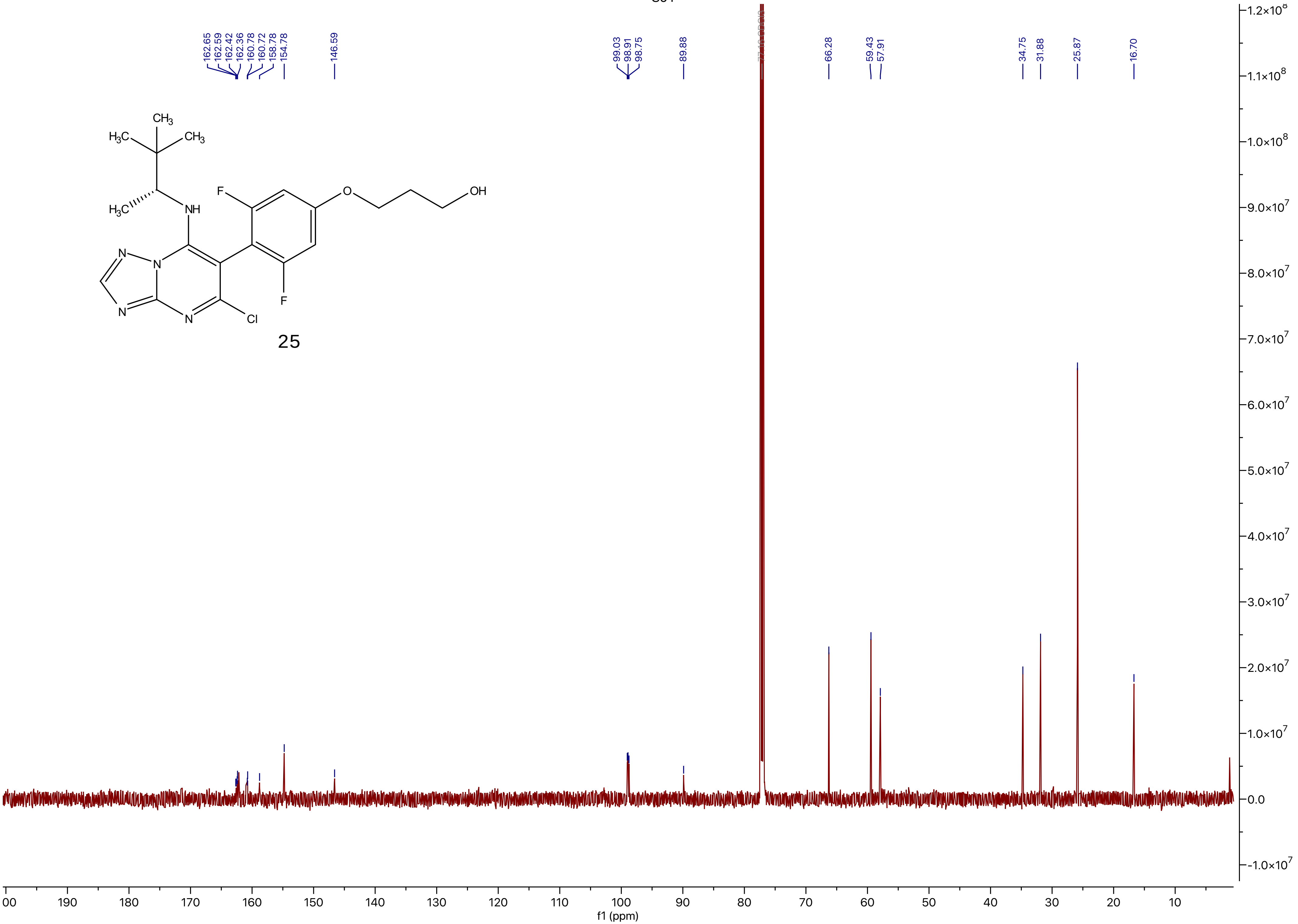

162.65
162.59
162.42
162.36
160.78
160.72
158.78
154.78
146.59
99.03
98.91
98.75
89.88
66.28
59.43
57.91
34.75
31.88
25.87
16.70
CH3
H3C
CH3
H3C
NH
F
O
OH
N
N
N
N
F
Cl
25
f1 (ppm)

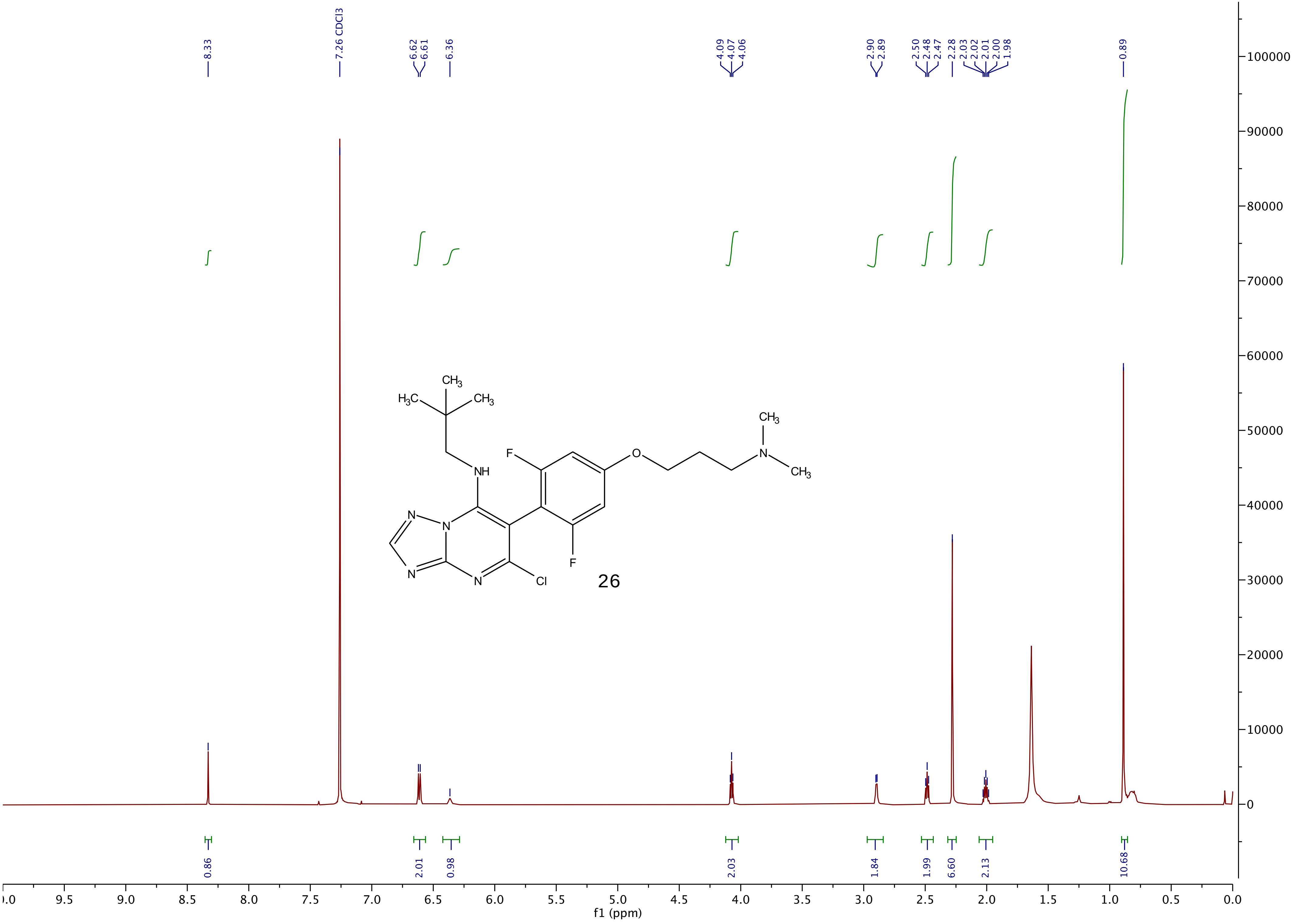

8.33
7.26 CDCl3
6.62
6.61
6.36
4.09
4.07
4.06
2.90
2.89
2.50
2.48
2.47
2.28
2.03
2.02
2.01
2.00
1.98
0.89
CH3
H3C
CH3
CH3
F
O
N
CH3
NH
N
N
N
F
N
Cl
26
0.86
2.01
0.98
2.03
1.84
1.99
6.60
2.13
10.68
100000
90000
80000
70000
60000
50000
40000
30000
20000
10000
0
9.5
9.0
8.5
8.0
7.5
7.0
6.5
6.0
5.5
5.0
4.5
4.0
3.5
3.0
2.5
2.0
1.5
1.0
0.5
0.0
f1 (ppm)

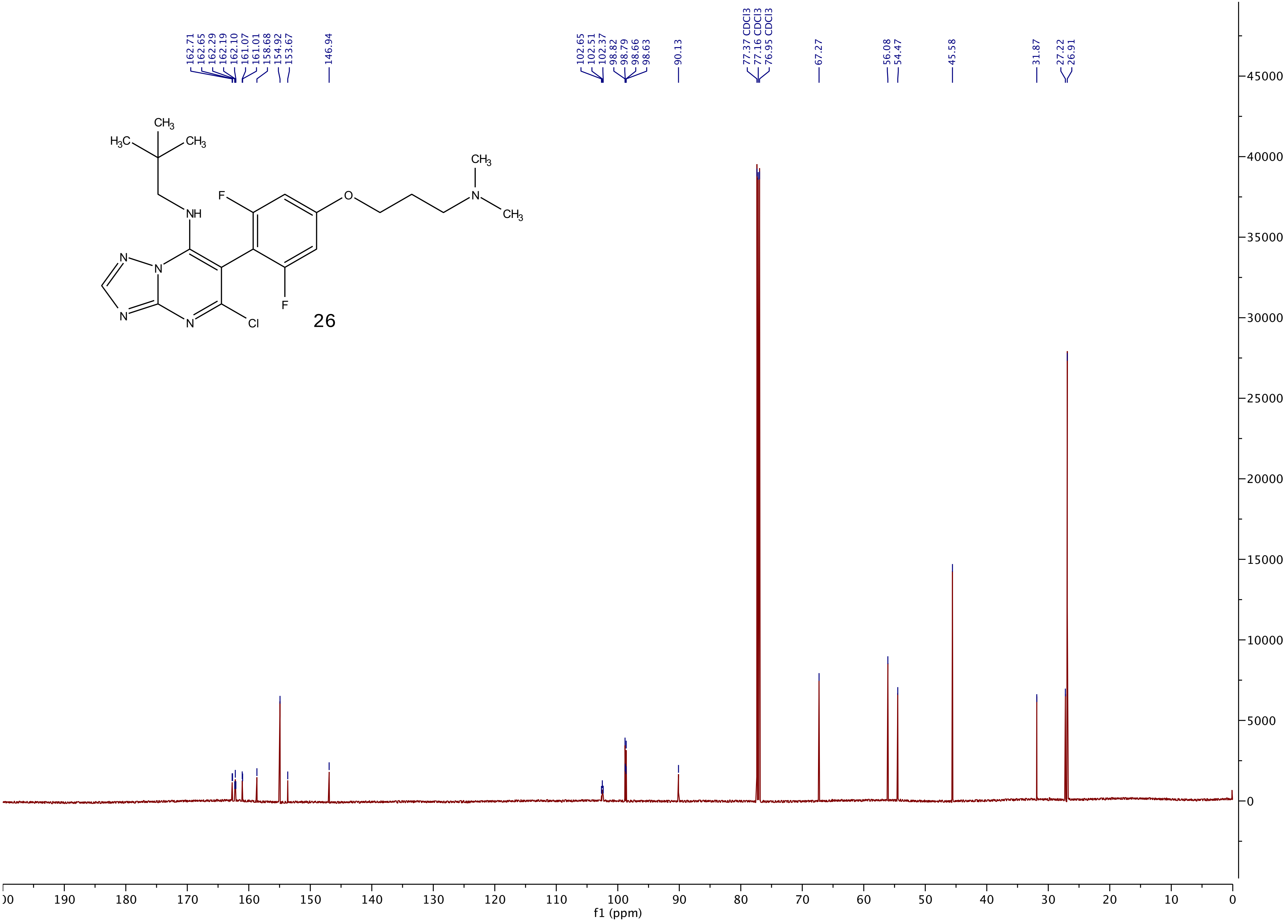
162.71
162.65
162.29
162.19
162.10
161.07
161.01
158.68
154.92
153.67
146.94
102.65
102.51
102.37
98.82
98.79
98.66
98.63
90.13
77.37 CDCl3
77.16 CDCl3
76.95 CDCl3
67.27
56.08
54.47
45.58
31.87
27.22
26.91
CH3
H3C
CH3
CH3
N
CH3
F
O
NH
N
N
N
F
N
Cl
26
45000
40000
35000
30000
25000
20000
15000
10000
5000
0
190
180
170
160
150
140
130
120
110
100
90
80
70
60
50
40
30
20
10
0
f1 (ppm)

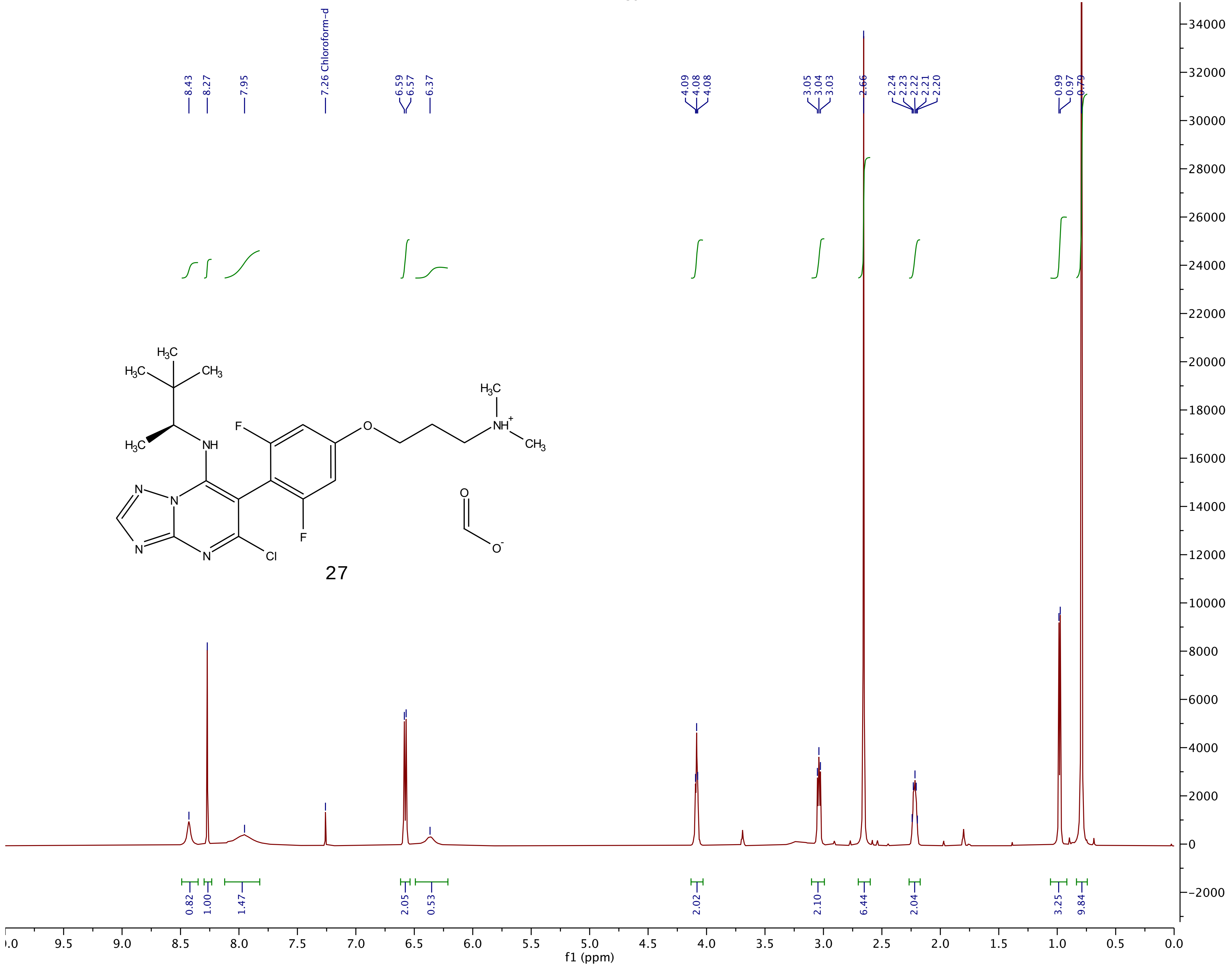
8.43
8.27
7.95
7.26 Chloroform-d
6.59
6.57
6.37
4.09
4.08
4.08
3.05
3.04
3.03
2.66
2.24
2.23
2.22
2.21
2.20
0.99
0.97
0.79
0.82
1.00
1.47
2.05
0.53
2.02
2.10
6.44
2.04
3.25
9.84
27
f1 (ppm)

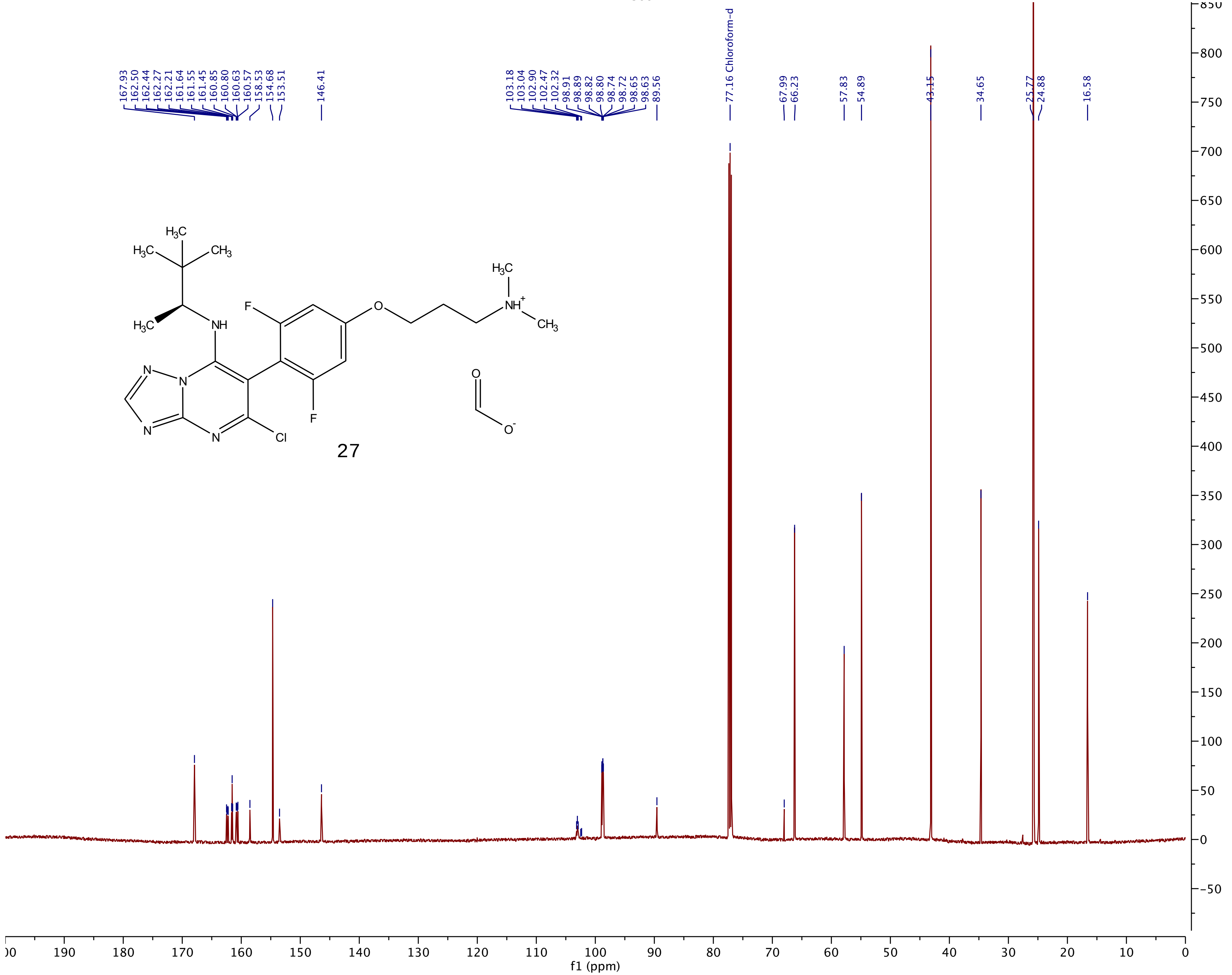
167.93
162.50
162.44
162.27
162.21
161.64
161.55
161.45
160.85
160.80
160.63
160.57
158.53
154.68
153.51
146.41
103.18
103.04
102.90
102.47
102.32
98.91
98.89
98.82
98.80
98.74
98.72
98.65
98.63
89.56
77.16 Chloroform-d
67.99
66.23
57.83
54.89
43.15
34.65
25.77
24.88
16.58
27
f1 (ppm)

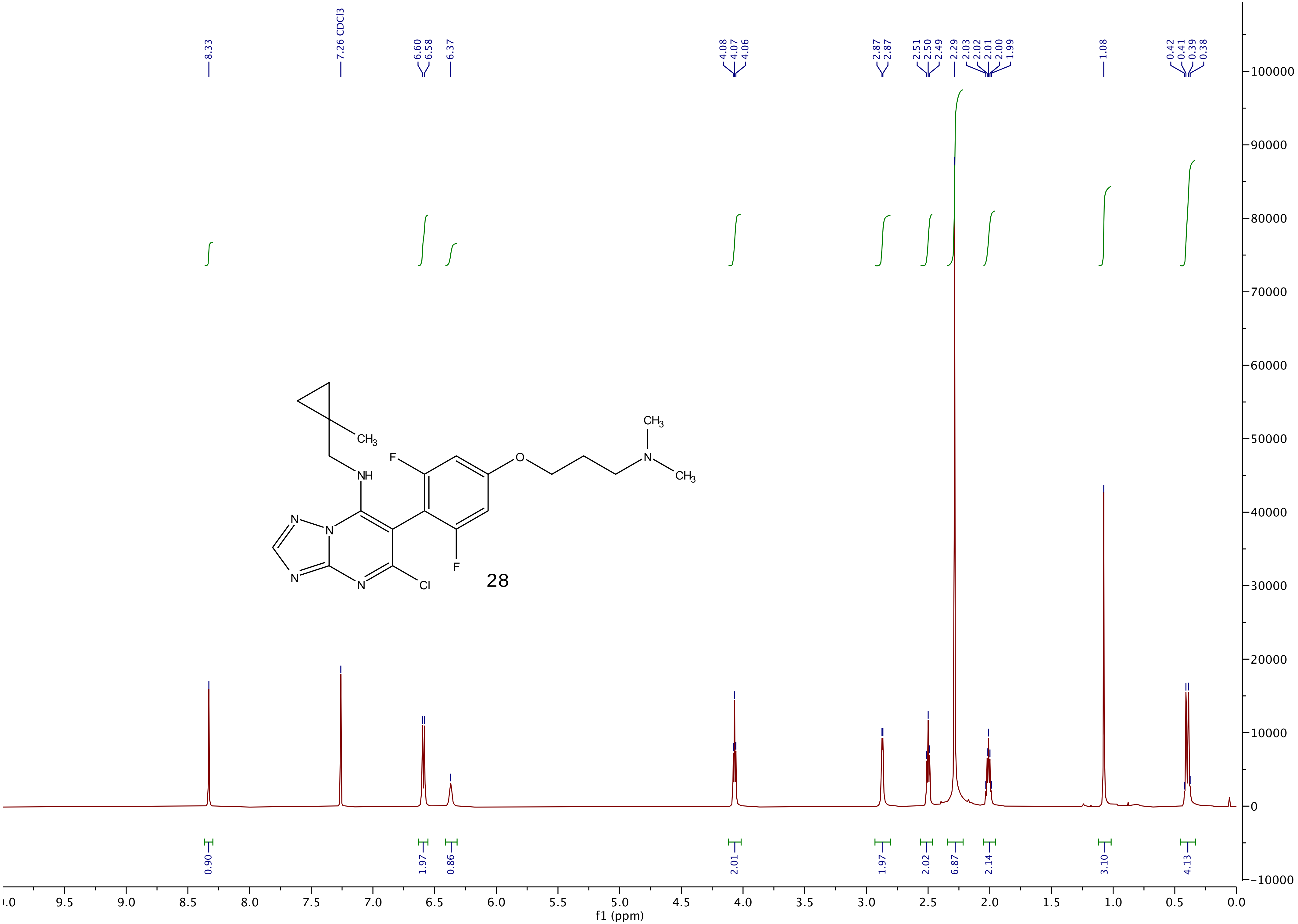
8.33
7.26 CDCl3
6.60
6.58
6.37
4.08
4.07
4.06
2.87
2.87
2.51
2.50
2.49
2.29
2.03
2.02
2.01
2.00
1.99
1.08
0.42
0.41
0.39
0.38
0.90
1.97
0.86
2.01
1.97
2.02
6.87
2.14
3.10
4.13
CH3
F
O
N
NH
N
F
Cl
28
f1 (ppm)

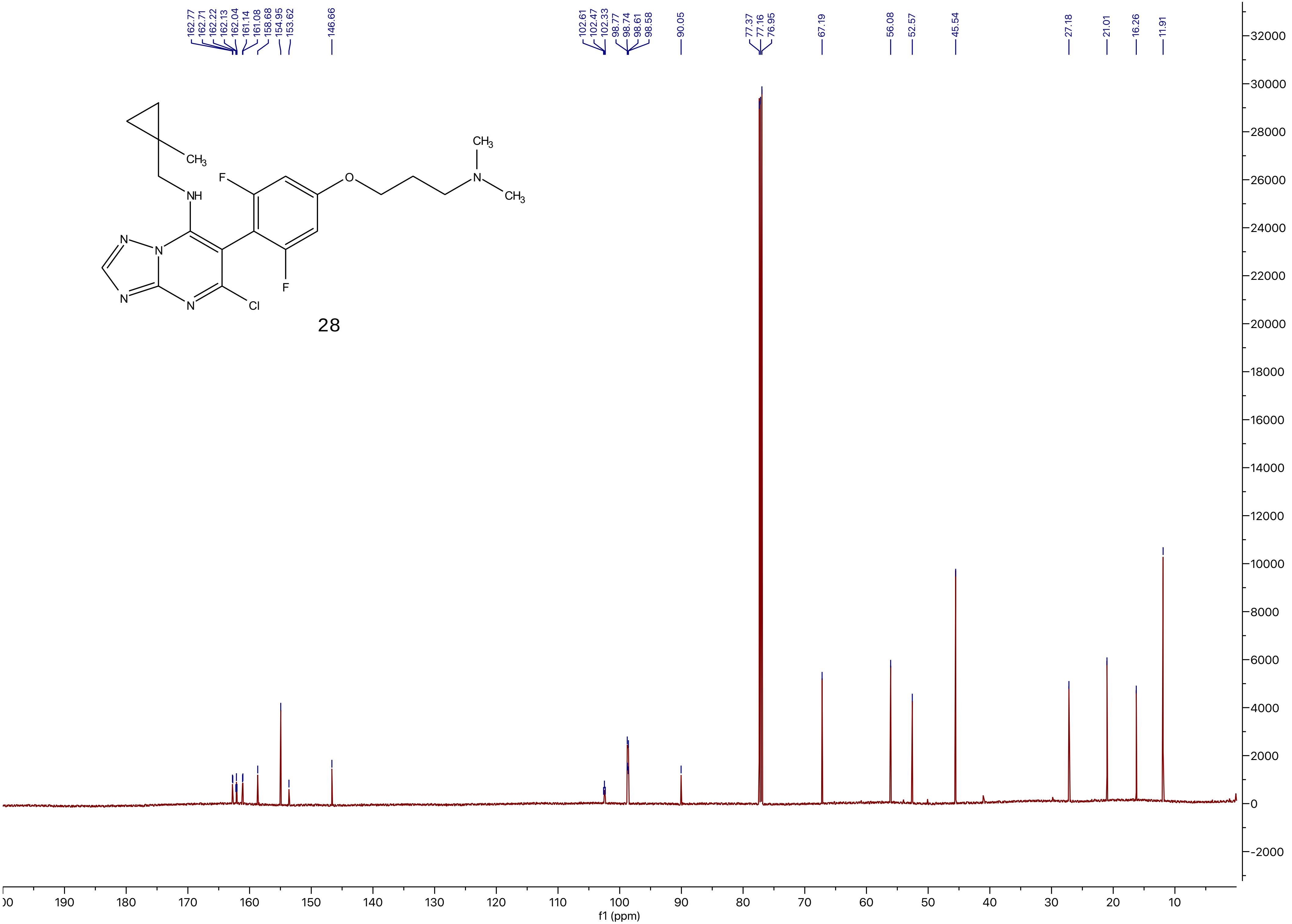
162.77
162.71
162.22
162.13
162.04
161.14
161.08
158.68
154.95
153.62
146.66
102.61
102.47
102.33
98.77
98.74
98.61
98.58
90.05
77.37
77.16
76.95
67.19
56.08
52.57
45.54
27.18
21.01
16.26
11.91
CH3
CH3
F
O
N
CH3
NH
N
N
F
N
N
Cl
28
f1 (ppm)

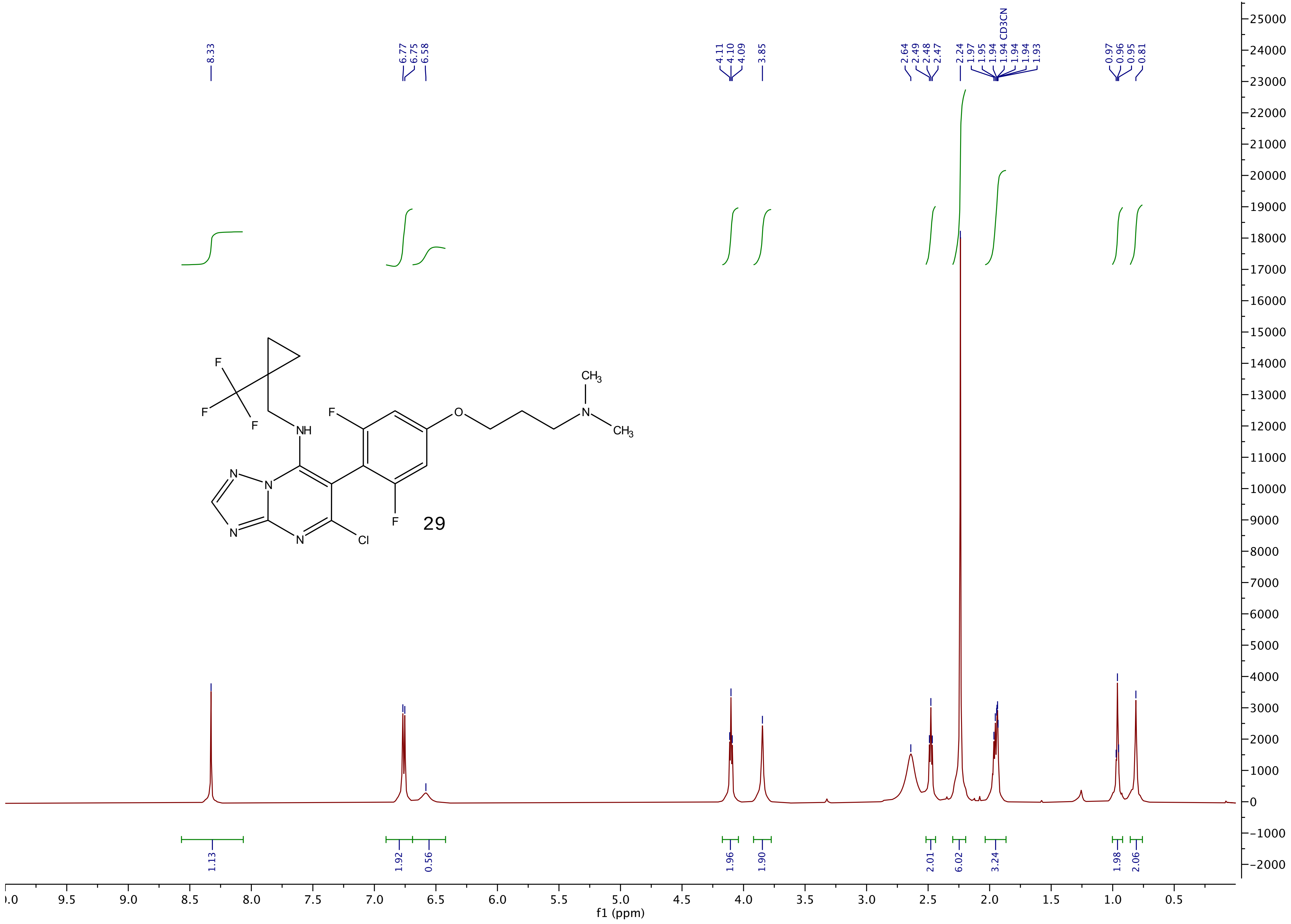
8.33
6.77
6.75
6.58
4.11
4.10
4.09
3.85
2.64
2.49
2.48
2.47
2.24
1.97
1.95
1.94
1.94 CD3CN
1.94
1.94
1.93
0.97
0.96
0.95
0.81
1.13
1.92
0.56
1.96
1.90
2.01
6.02
3.24
1.98
2.06
F
F
F
NH
N
N
N
N
Cl
F
F
O
N
CH3
CH3
29
f1 (ppm)

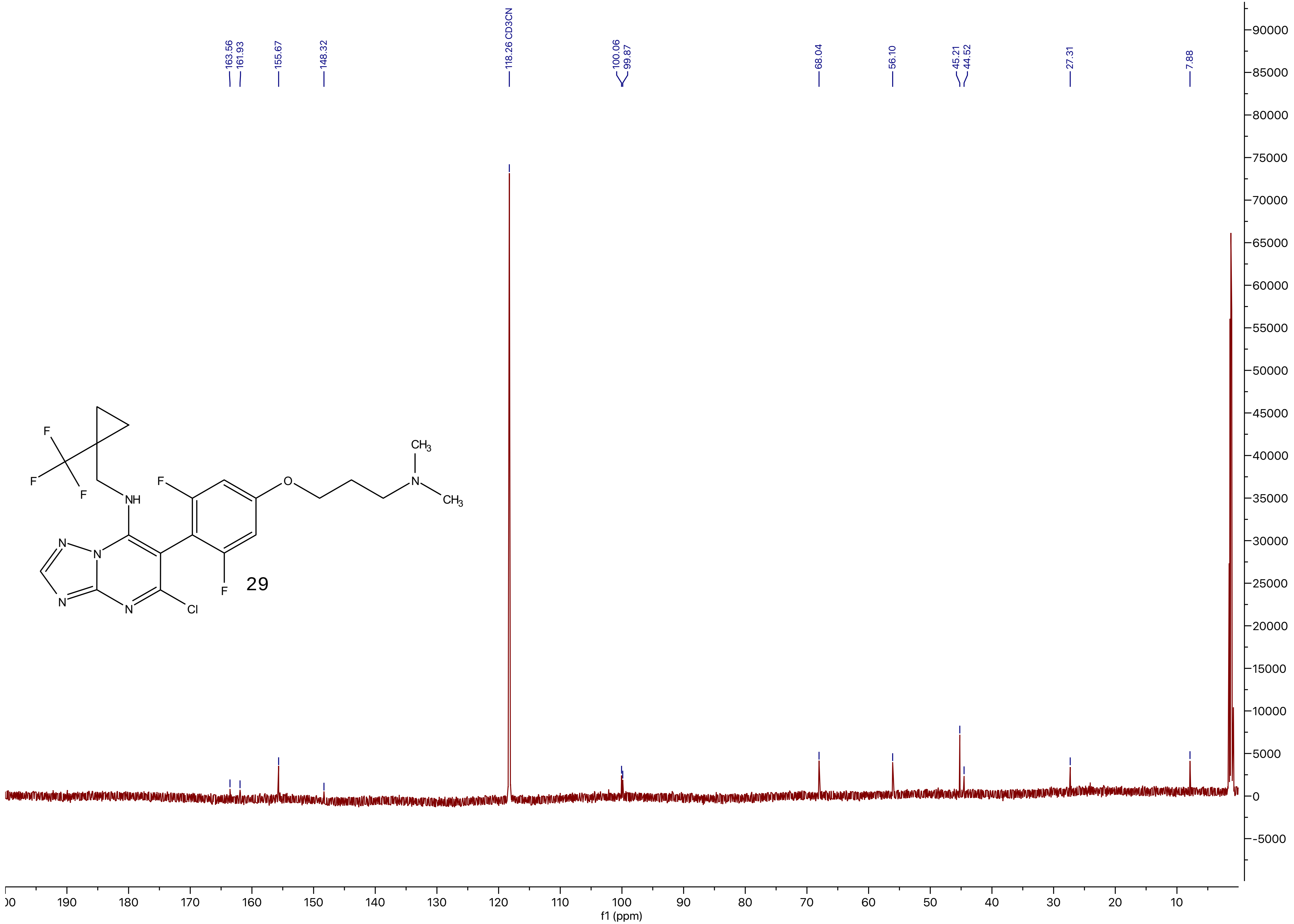
163.56
161.93
155.67
148.32
118.26 CD3CN
100.06
99.87
68.04
56.10
45.21
44.52
27.31
7.88
f1 (ppm)

29

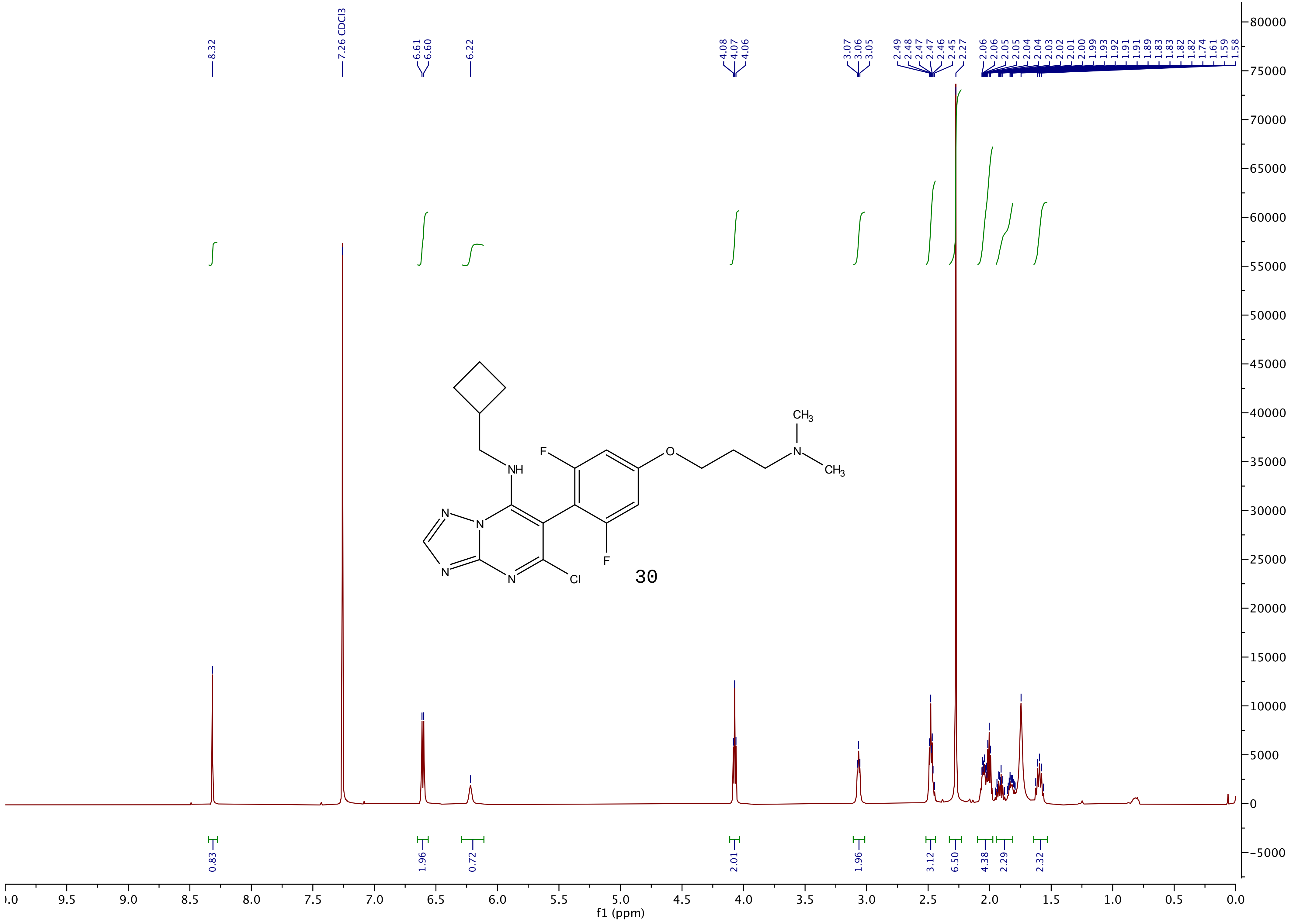
8.32
7.26 CDCl3
6.61
6.60
6.22
4.08
4.07
4.06
3.07
3.06
3.05
2.49
2.48
2.47
2.47
2.46
2.45
2.27
2.06
2.06
2.05
2.05
2.04
2.04
2.03
2.02
2.01
2.00
1.99
1.93
1.92
1.91
1.91
1.89
1.83
1.83
1.82
1.82
1.74
1.61
1.59
1.58
0.83
1.96
0.72
2.01
1.96
3.12
6.50
4.38
2.29
2.32
NH
F
F
O
N
CH3
CH3
N
N
N
N
Cl
30
f1 (ppm)

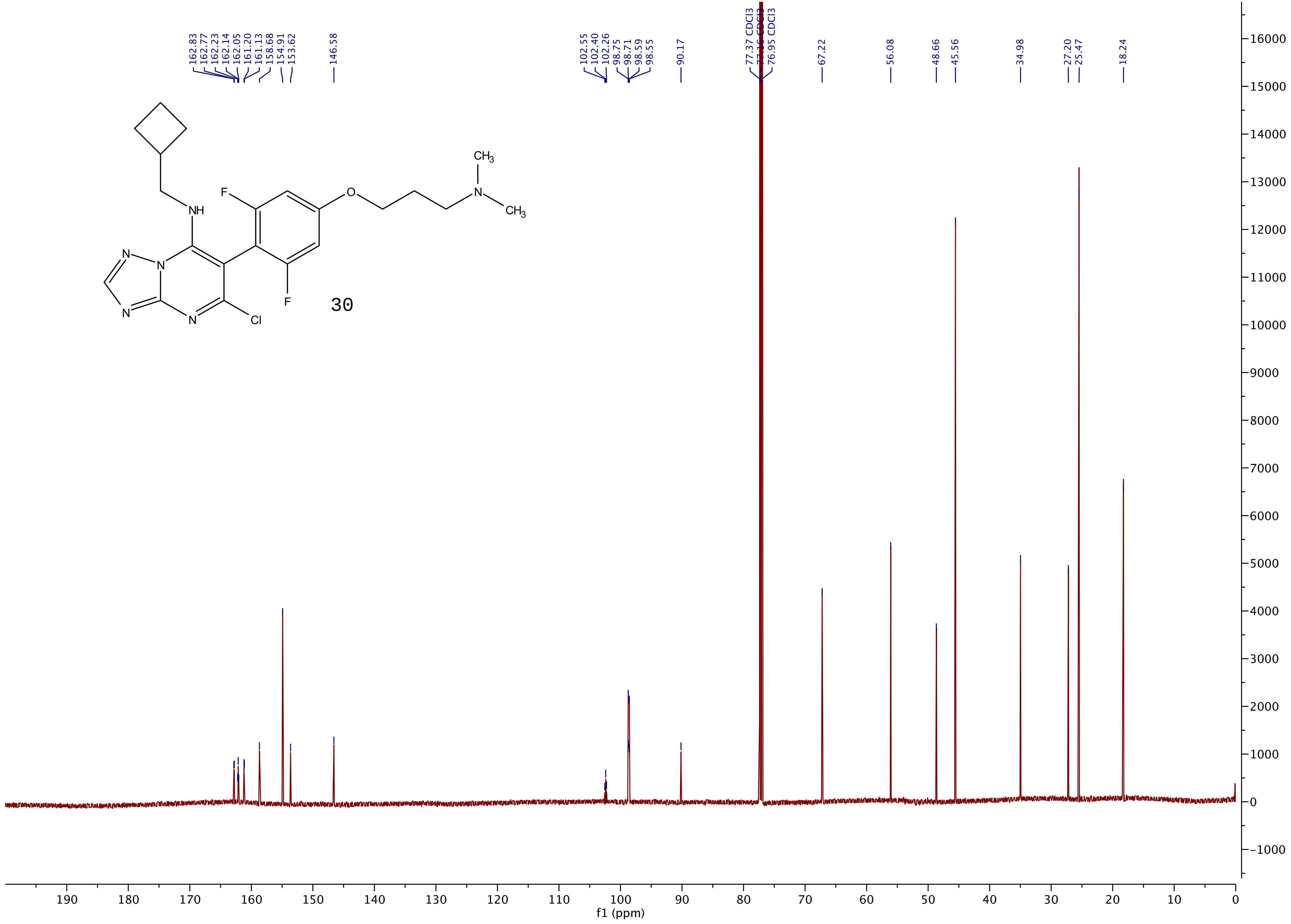
162.83
162.77
162.23
162.14
162.05
161.20
161.13
158.68
154.91
153.62
146.58
102.55
102.40
102.26
98.75
98.71
98.59
98.55
90.17
77.37 CDCl3
77.16 CDCl3
76.95 CDCl3
67.22
56.08
48.66
45.56
34.98
27.20
25.47
18.24
F
NH
O
N
CH3
CH3
N
N
N
N
Cl
F
30
16000
15000
14000
13000
12000
11000
10000
9000
8000
7000
6000
5000
4000
3000
2000
1000
0
−1000
190
180
170
160
150
140
130
120
110
100
90
80
70
60
50
40
30
20
10
0
f1 (ppm)

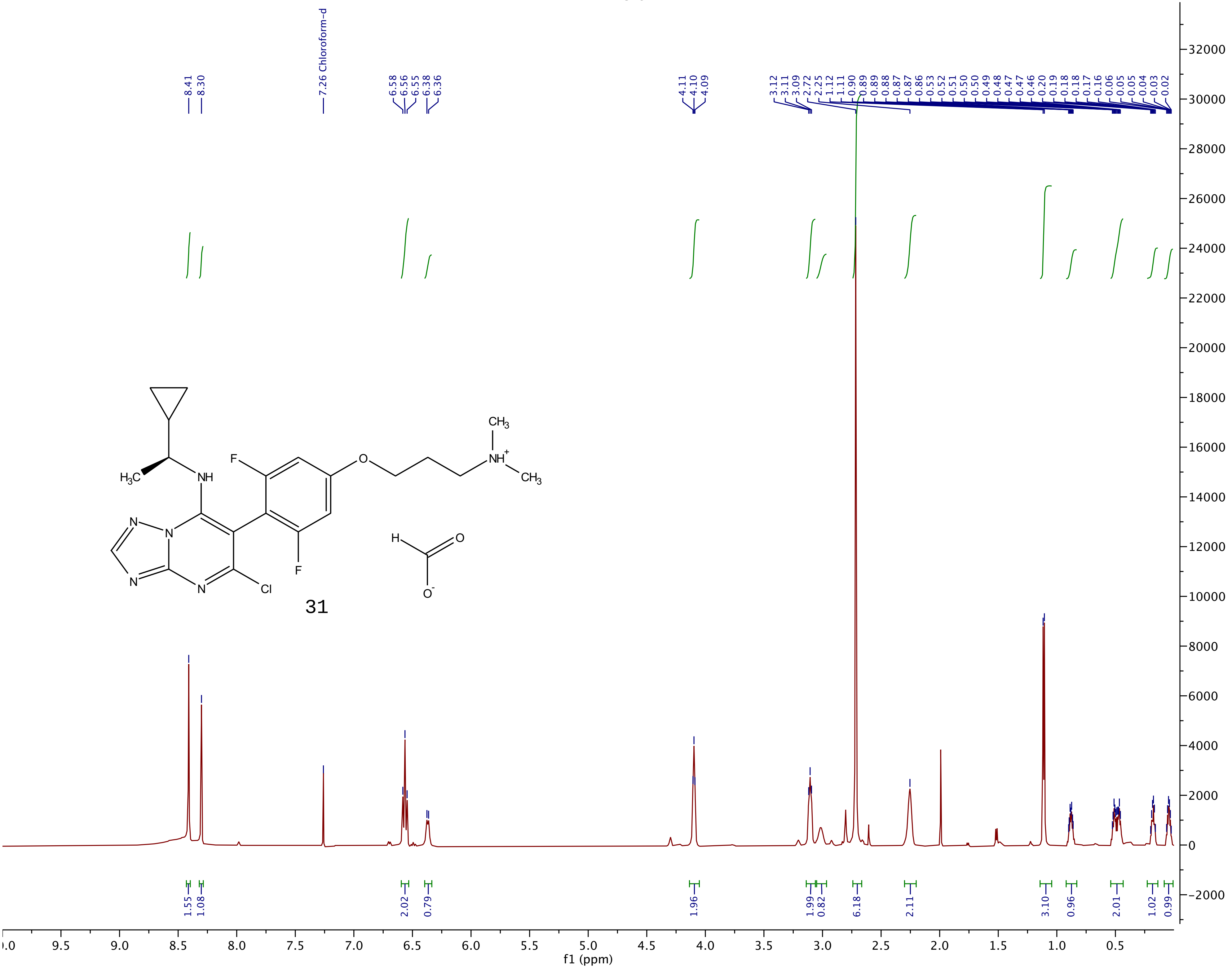
7.26 Chloroform-d
31
f1 (ppm)

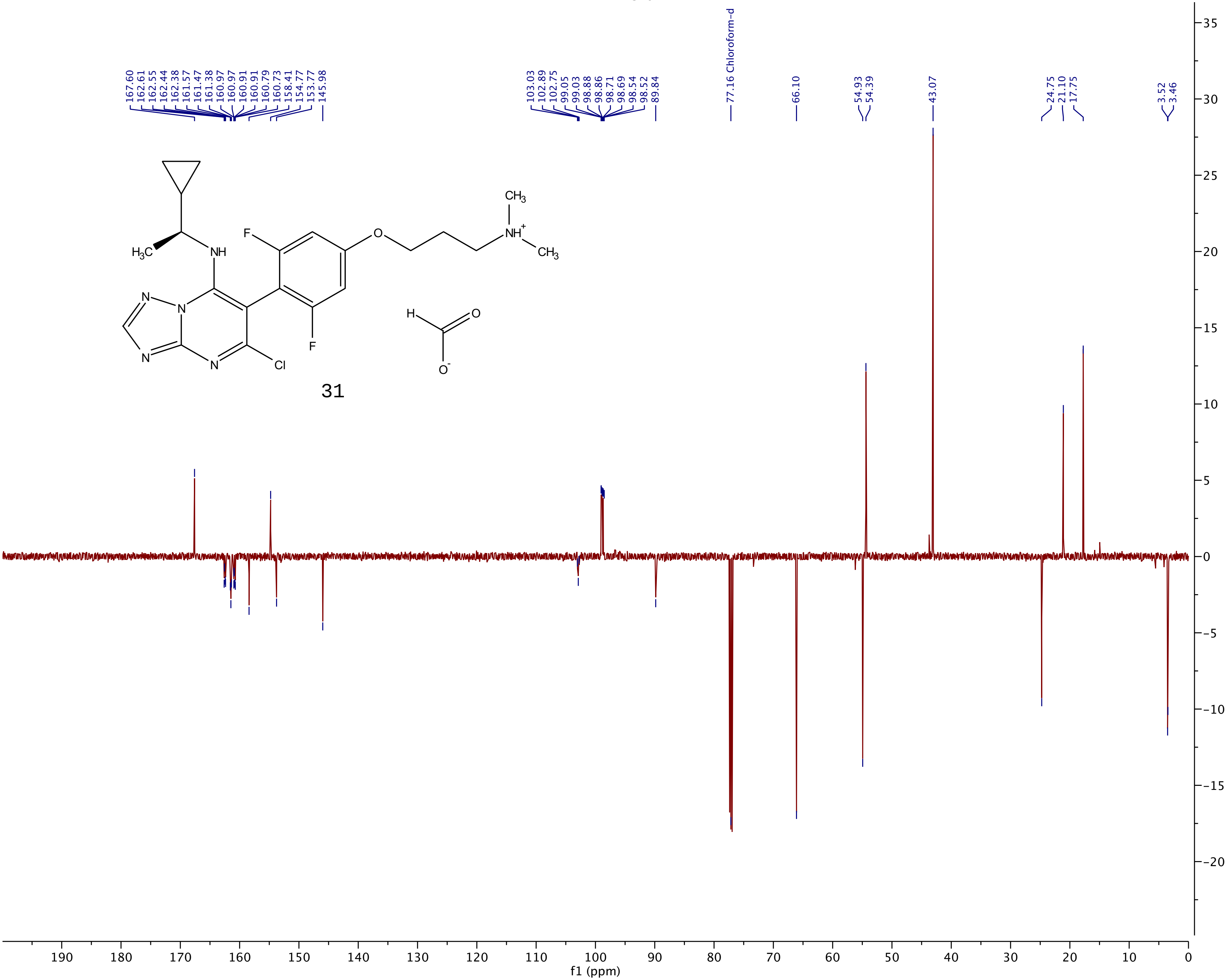
167.60
162.61
162.55
162.44
162.38
161.57
161.47
161.38
160.97
160.97
160.91
160.91
160.79
160.73
158.41
154.77
153.77
145.98
103.03
102.89
102.75
99.05
99.03
98.88
98.86
98.71
98.69
98.54
98.52
89.84
77.16 Chloroform-d
66.10
54.93
54.39
43.07
24.75
21.10
17.75
3.52
3.46
CH3
NH+
CH3
O
F
F
H3C
NH
N
N
N
N
Cl
H
O
O-
31
f1 (ppm)

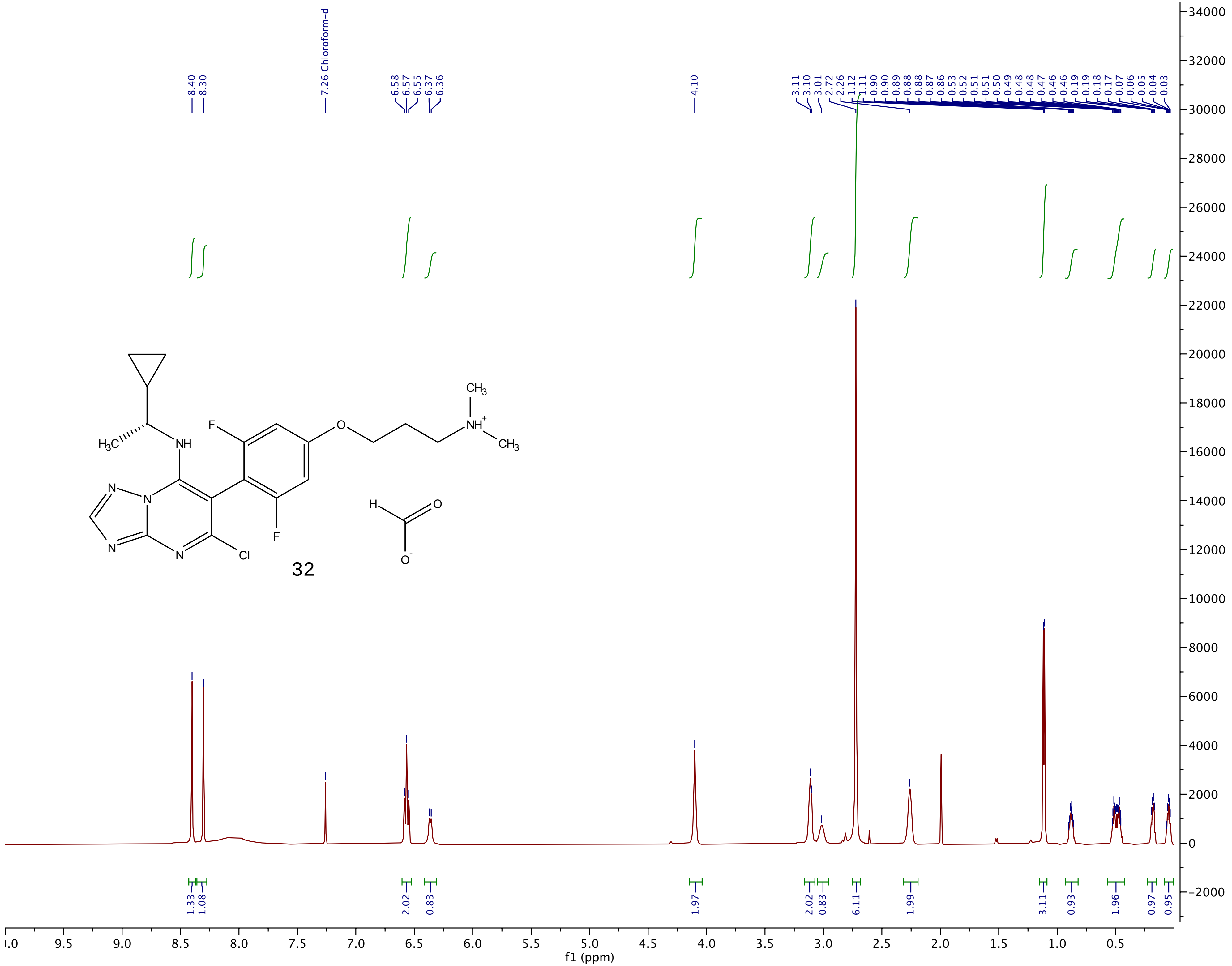
8.40
8.30
7.26 Chloroform-d
6.58
6.57
6.55
6.37
6.36
4.10
3.11
3.10
3.01
2.72
2.26
1.12
1.11
0.90
0.89
0.88
0.88
0.87
0.86
0.53
0.52
0.51
0.51
0.50
0.49
0.48
0.48
0.47
0.46
0.19
0.19
0.18
0.17
0.07
0.06
0.05
0.04
0.03
1.33
1.08
2.02
0.83
1.97
2.02
0.83
6.11
1.99
3.11
0.93
1.96
0.97
0.95
f1 (ppm)
CH3
NH+
CH3
O
F
F
H3C
NH
N
N
N
N
Cl
H
O
O-
32

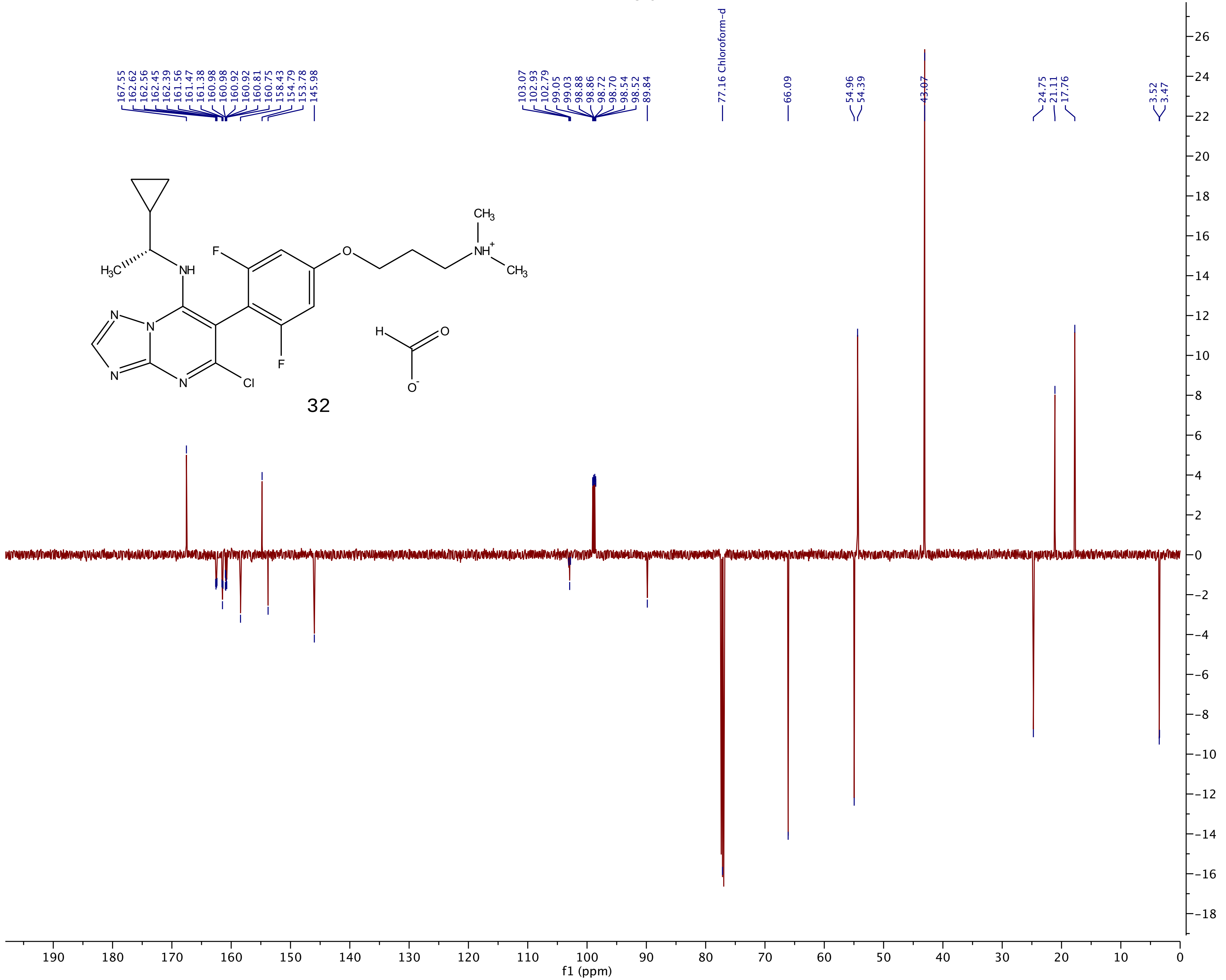
167.55
162.62
162.56
162.45
162.39
161.56
161.47
161.38
160.98
160.98
160.92
160.92
160.81
160.75
158.43
154.79
153.78
145.98
103.07
102.93
102.79
99.05
99.03
98.88
98.86
98.72
98.70
98.54
98.52
89.84
77.16 Chloroform-d
66.09
54.96
54.39
43.07
24.75
21.11
17.76
3.52
3.47
32
f1 (ppm)

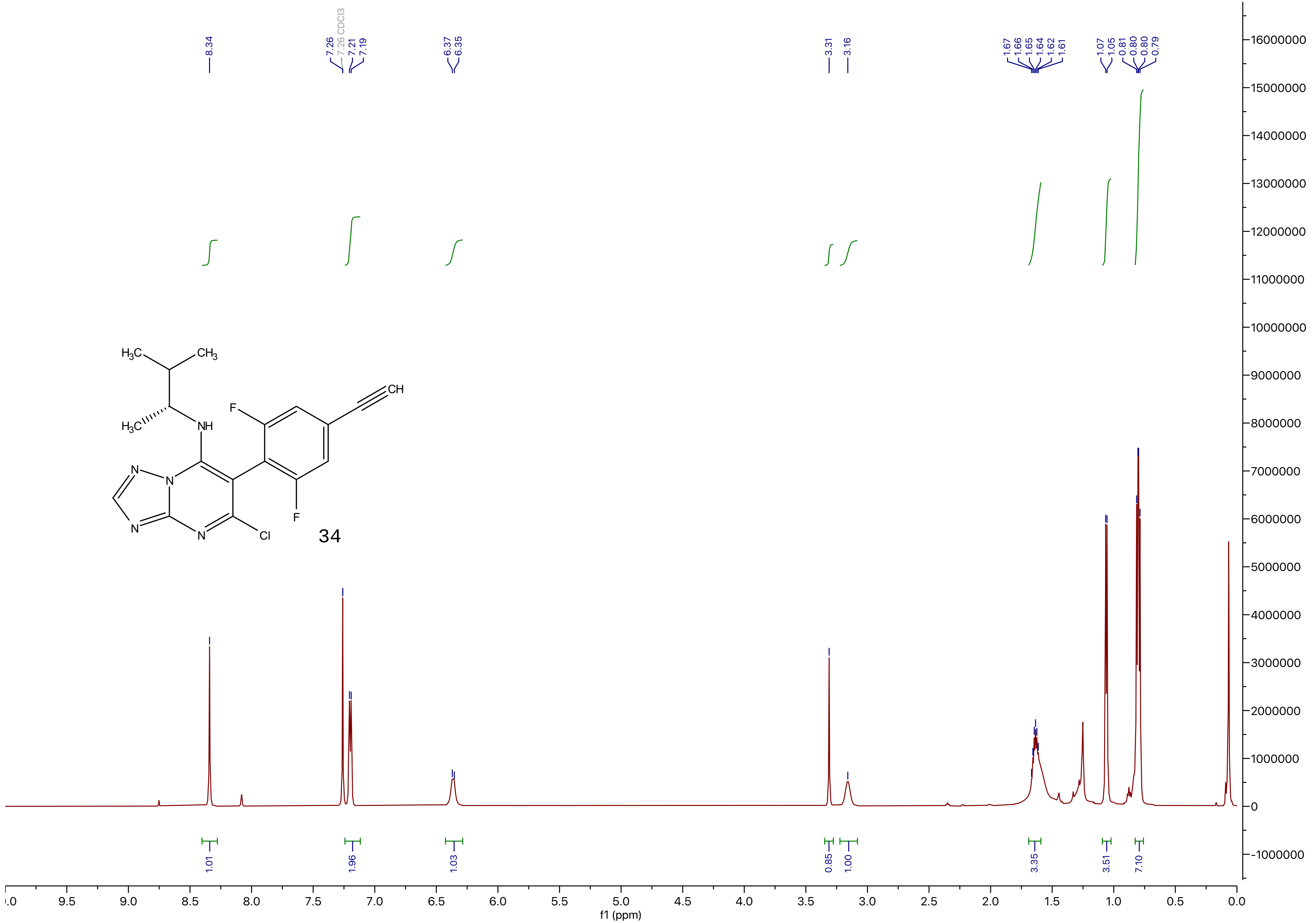
8.34
7.26
7.26 CDCl3
7.21
7.19
6.37
6.35
3.31
3.16
1.67
1.66
1.65
1.64
1.62
1.61
1.07
1.05
0.81
0.80
0.80
0.79
1.01
1.96
1.03
0.85
1.00
3.35
3.51
7.10
f1 (ppm)
H3C
CH3
NH
F
F
CH
N
N
N
N
Cl

34

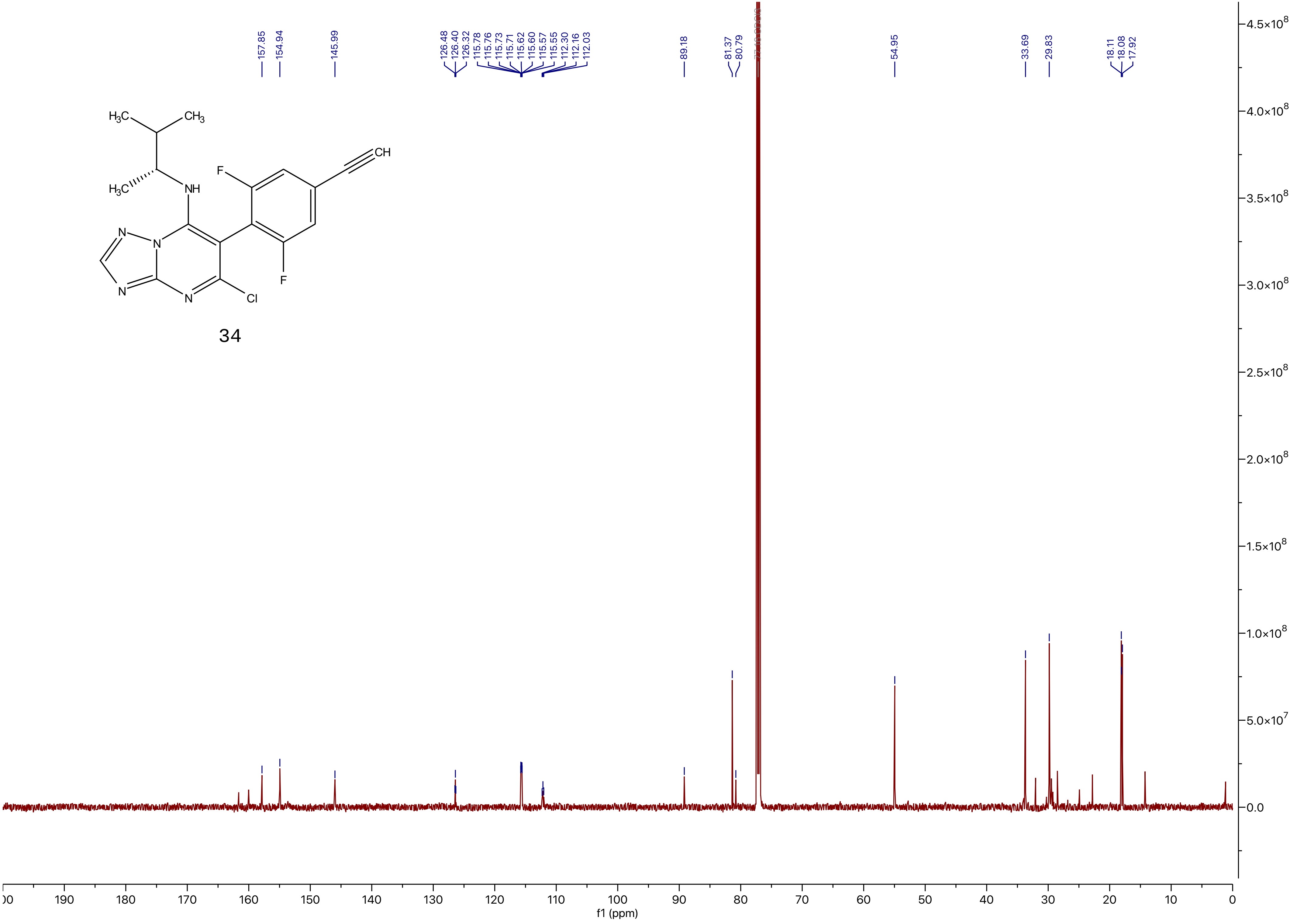
157.85
154.94
145.99
126.48
126.40
126.32
115.78
115.76
115.73
115.71
115.62
115.60
115.57
115.55
112.30
112.16
112.03
89.18
81.37
80.79
54.95
33.69
29.83
18.11
18.08
17.92
H3C
CH3
CH
F
H3C
NH
N
N
N
N
F
Cl
34
4.5×10^8
4.0×10^8
3.5×10^8
3.0×10^8
2.5×10^8
2.0×10^8
1.5×10^8
1.0×10^8
5.0×10^7
0.0
190
180
170
160
150
140
130
120
110
100
90
80
70
60
50
40
30
20
10
0
f1 (ppm)

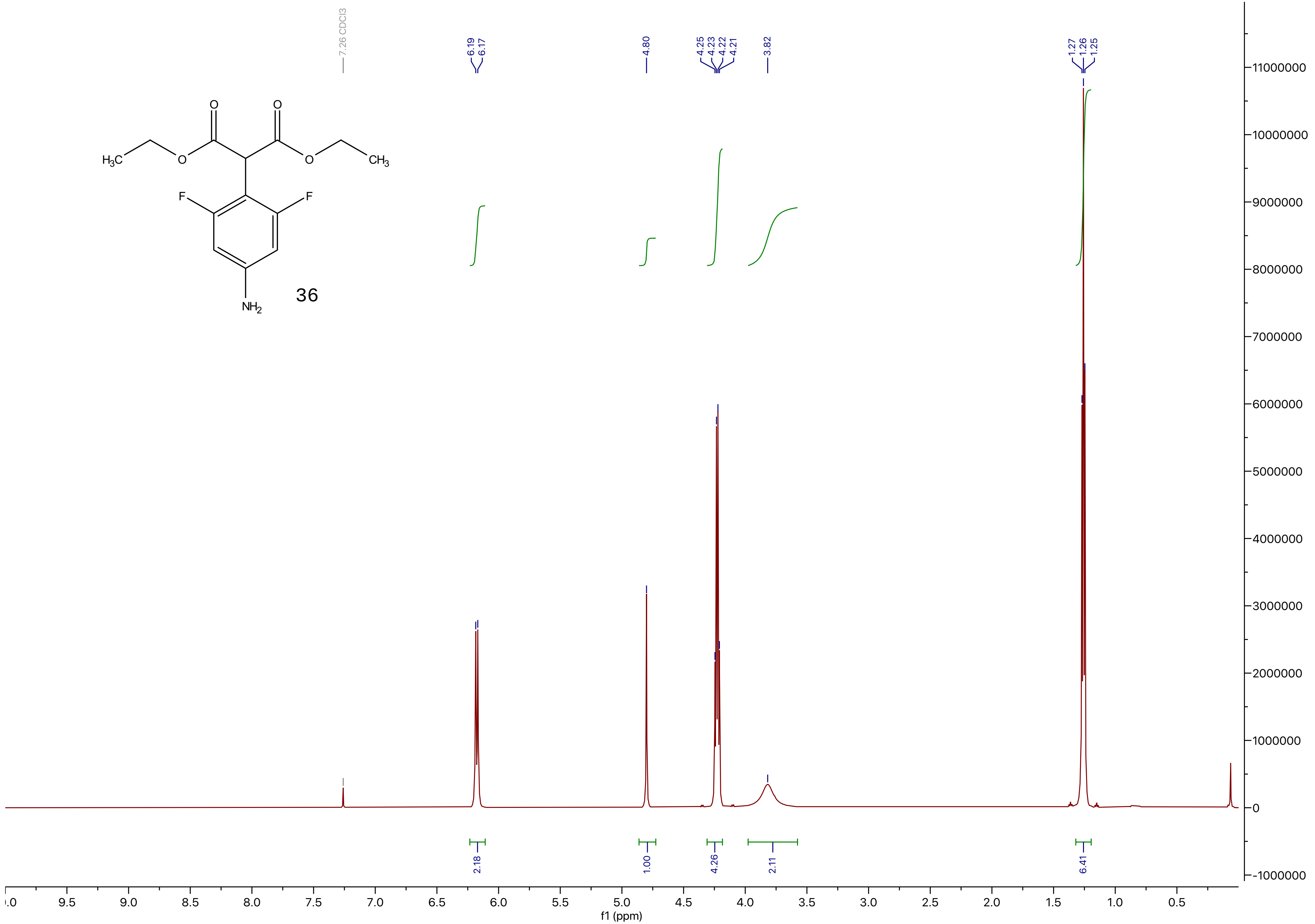
7.26 CDCl3
6.19
6.17
4.80
4.25
4.23
4.22
4.21
3.82
1.27
1.26
1.25
36
2.18
1.00
4.26
2.11
6.41
f1 (ppm)

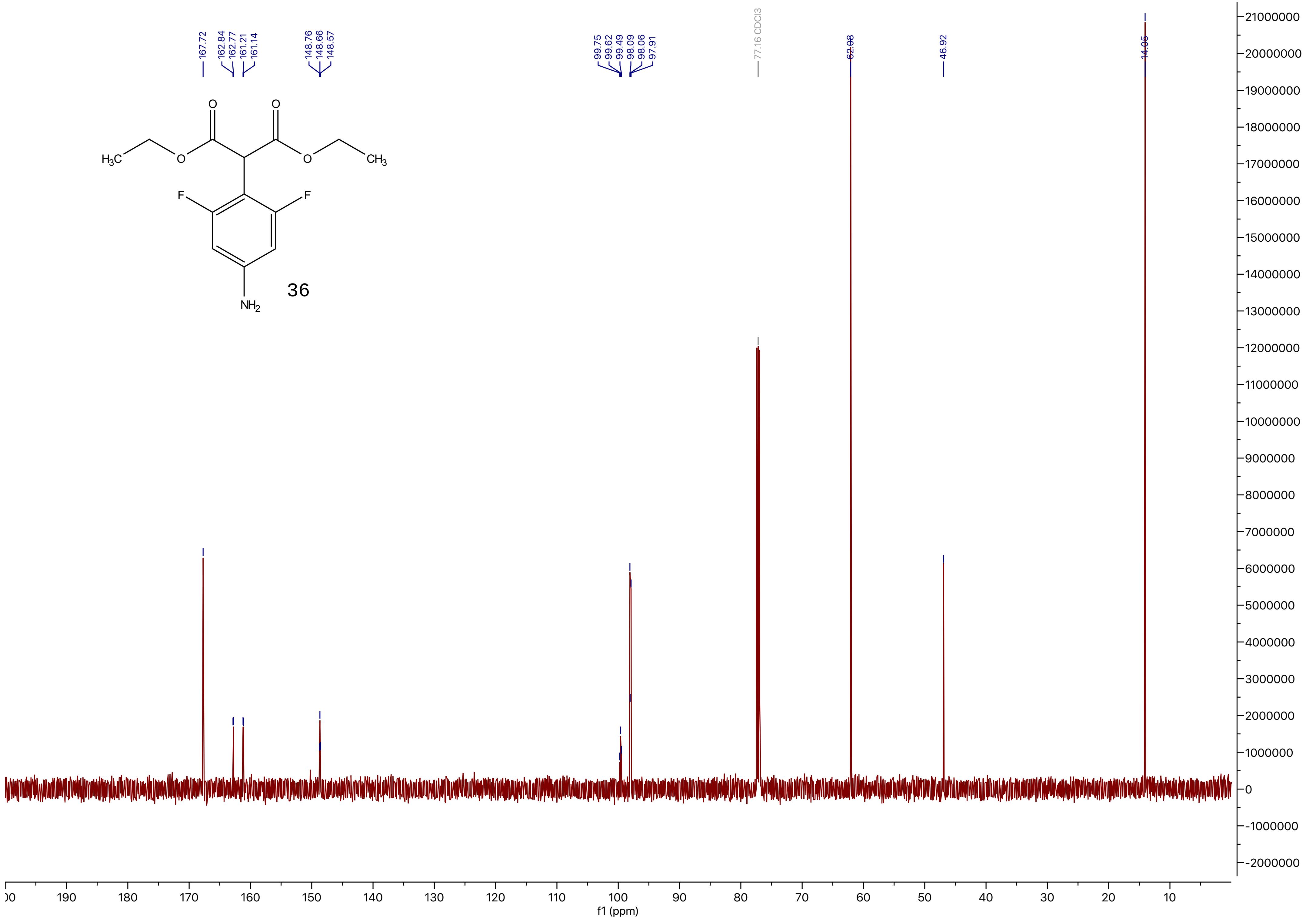
167.72
162.84
162.77
161.21
161.14
148.76
148.66
148.57
99.75
99.62
99.49
98.09
98.06
97.91
77.16 CDCl3
62.08
46.92
14.05
f1 (ppm)

36

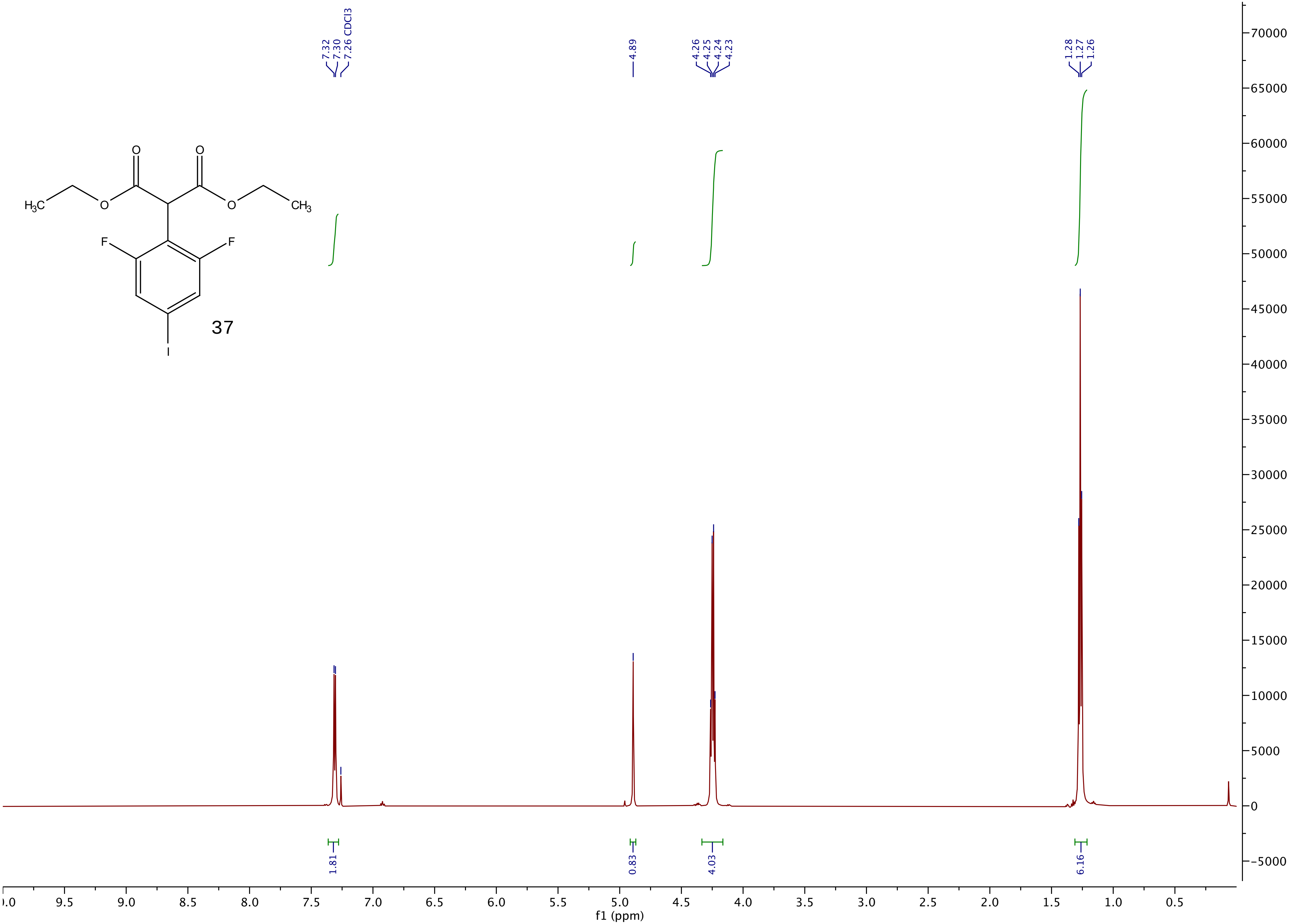
7.32
7.30
7.26 CDCl3
4.89
4.26
4.25
4.24
4.23
1.28
1.27
1.26
O
O
H3C
O
O
CH3
F
F
I
37
1.81
0.83
4.03
6.16
70000
65000
60000
55000
50000
45000
40000
35000
30000
25000
20000
15000
10000
5000
0
-5000
9.5
9.0
8.5
8.0
7.5
7.0
6.5
6.0
5.5
5.0
4.5
4.0
3.5
3.0
2.5
2.0
1.5
1.0
0.5
f1 (ppm)

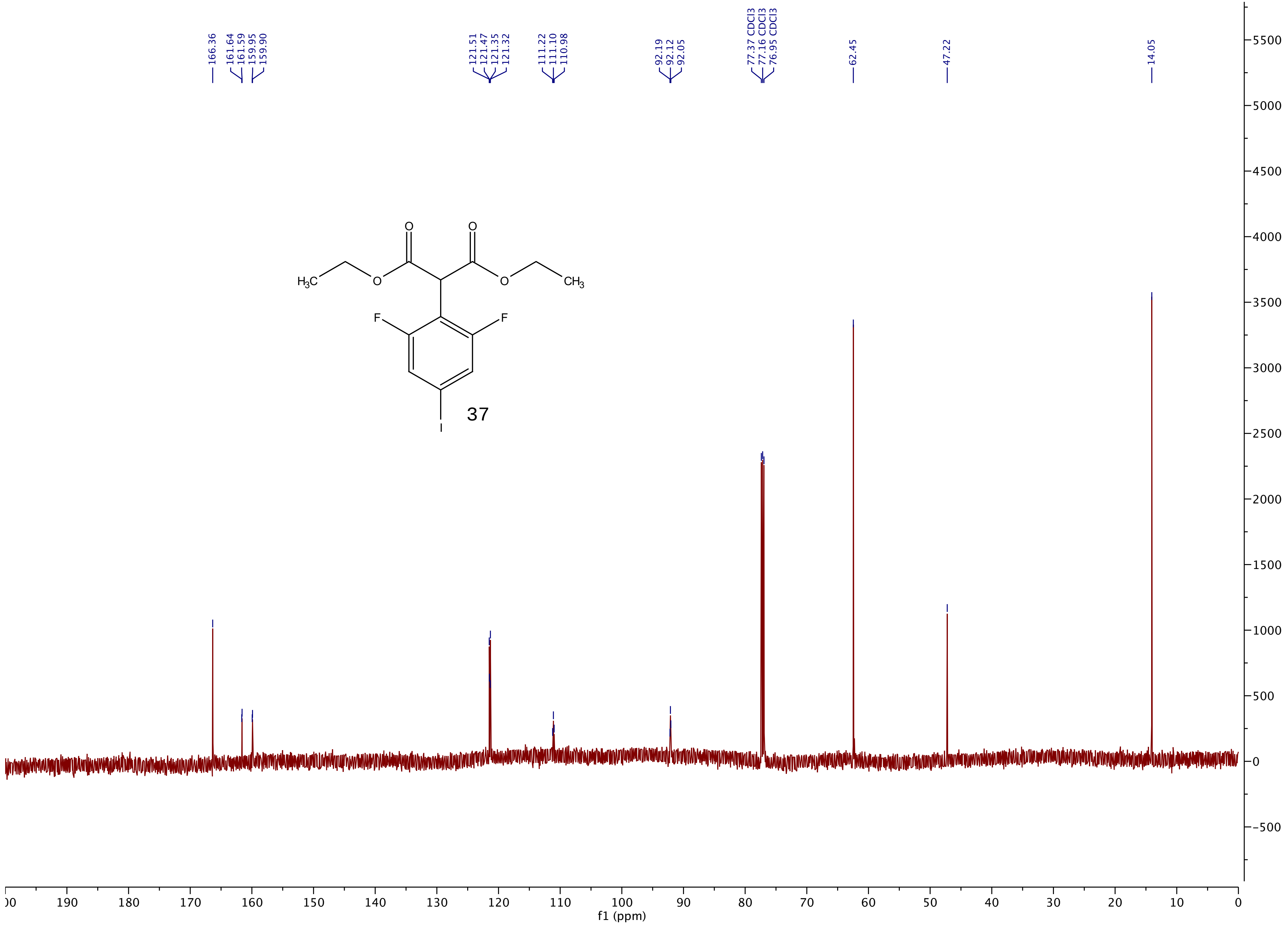
166.36
161.64
161.59
159.95
159.90
121.51
121.47
121.35
121.32
111.22
111.10
110.98
92.19
92.12
92.05
77.37 CDCl3
77.16 CDCl3
76.95 CDCl3
62.45
47.22
14.05
O
O
H3C
O
O
CH3
F
F
I
37
f1 (ppm)

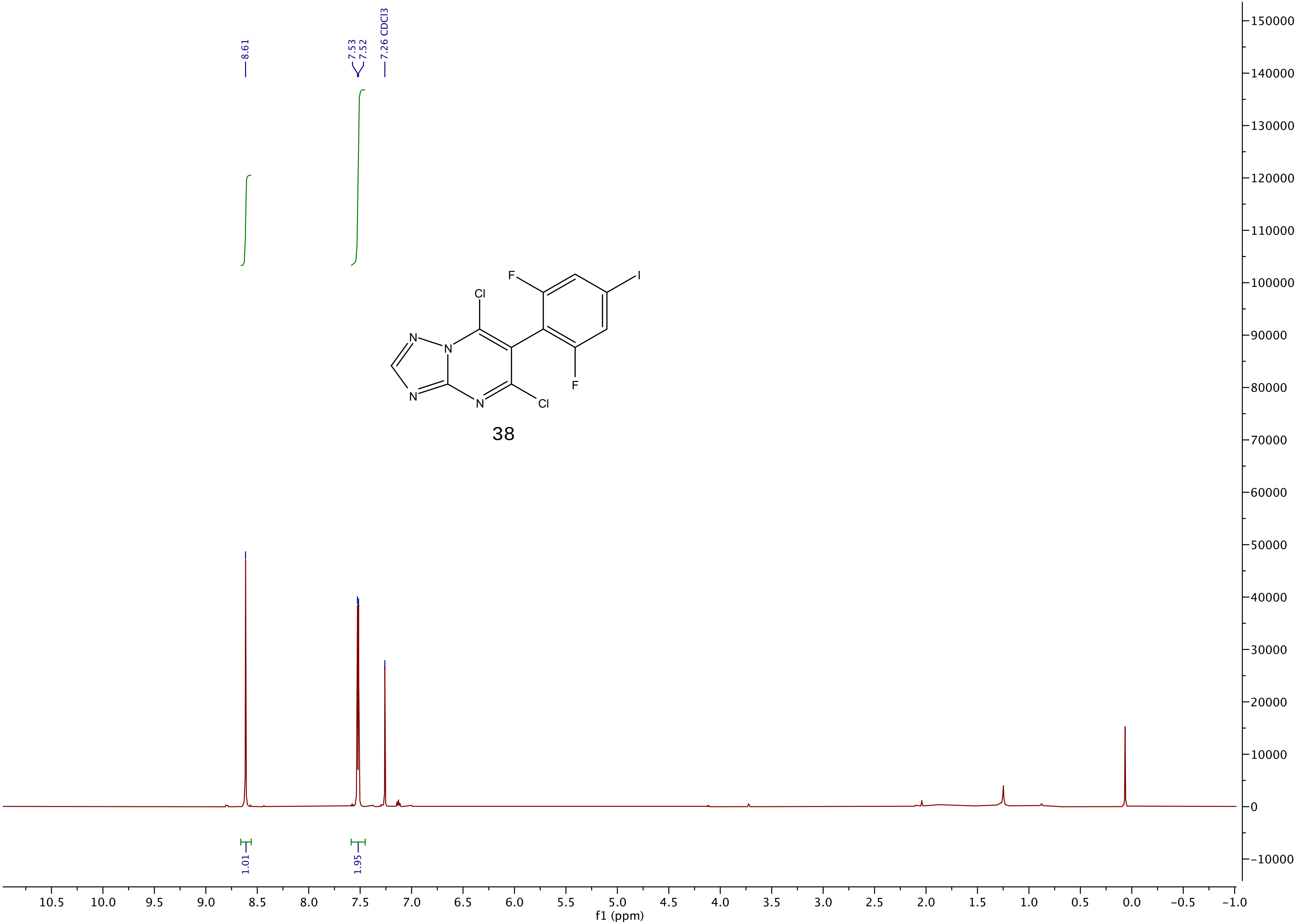
8.61
7.53
7.52
7.26 CDCl3
Cl
F
I
N
N
N
N
F
Cl
38
150000
140000
130000
120000
110000
100000
90000
80000
70000
60000
50000
40000
30000
20000
10000
0
−10000
1.01
1.95
10.5 10.0 9.5 9.0 8.5 8.0 7.5 7.0 6.5 6.0 5.5 5.0 4.5 4.0 3.5 3.0 2.5 2.0 1.5 1.0 0.5 0.0 −0.5 −1.0
f1 (ppm)

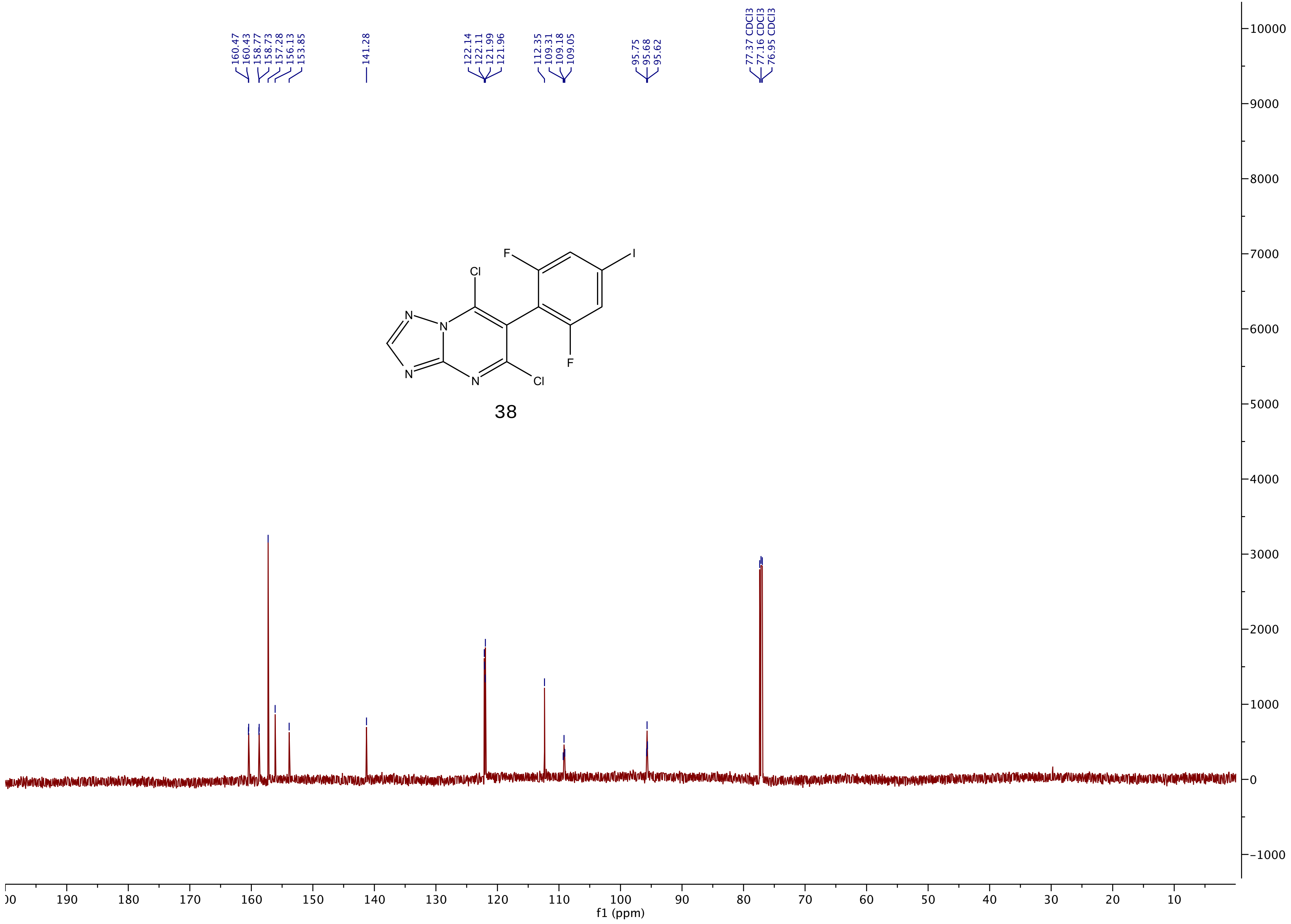
160.47
160.43
158.77
158.73
157.28
156.13
153.85
141.28
122.14
122.11
121.99
121.96
112.35
109.31
109.18
109.05
95.75
95.68
95.62
77.37 CDCl3
77.16 CDCl3
76.95 CDCl3
F
I
Cl
N
N
N
N
Cl
F
38
10000
9000
8000
7000
6000
5000
4000
3000
2000
1000
0
−1000
190
180
170
160
150
140
130
120
110
100
90
80
70
60
50
40
30
20
10
f1 (ppm)

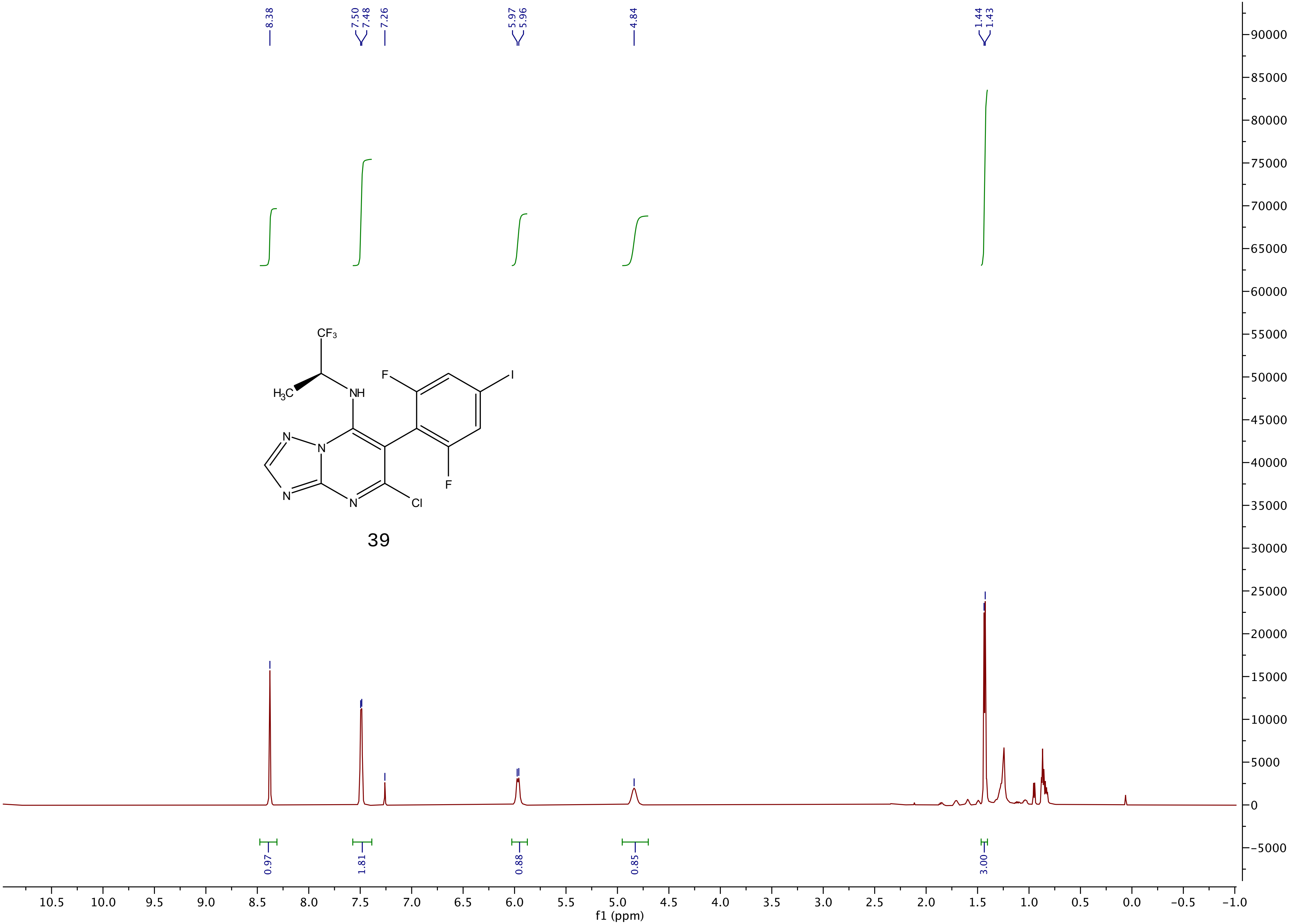

39

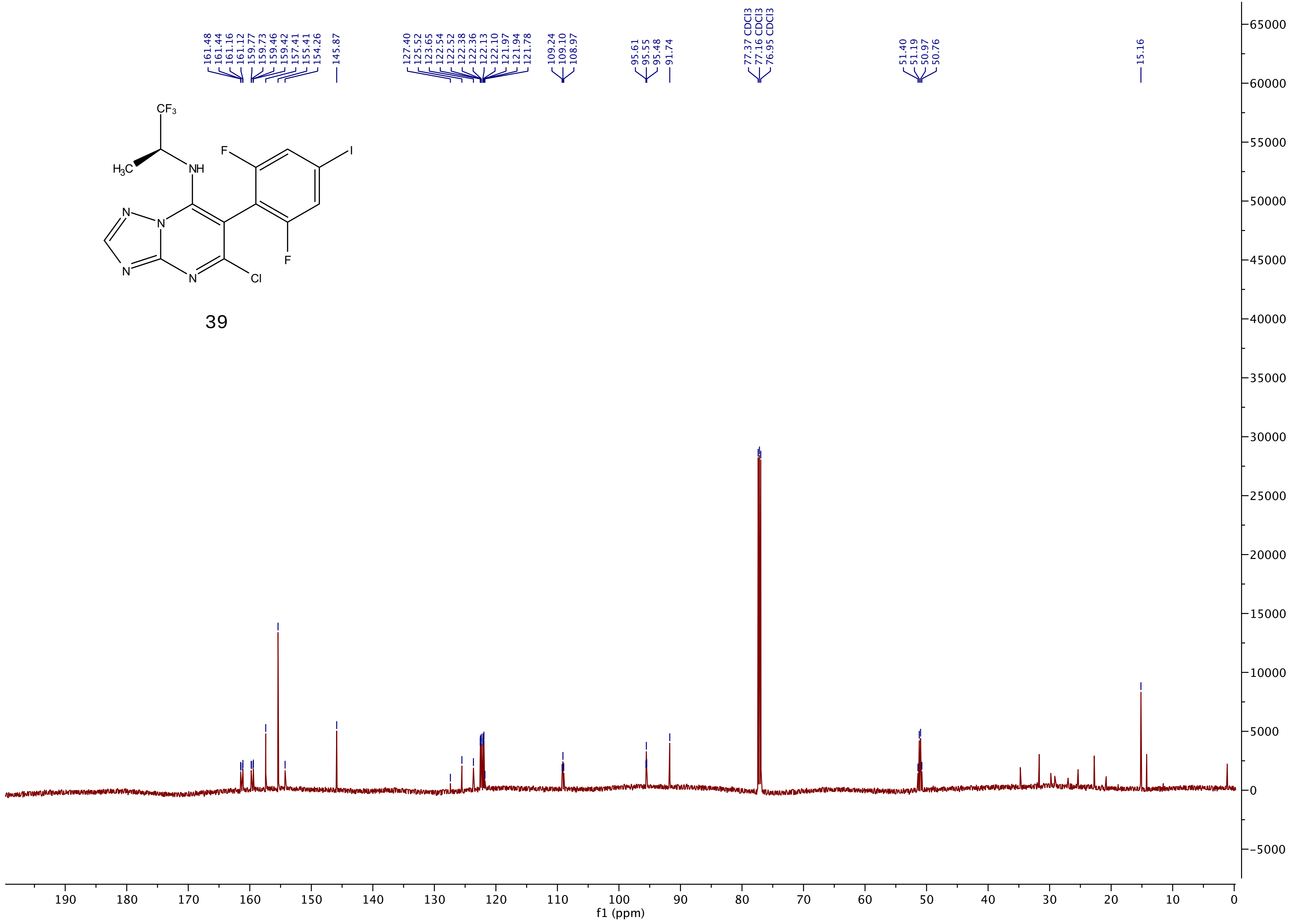
161.48
161.44
161.16
161.12
159.77
159.73
159.46
159.42
157.41
155.41
154.26
145.87
127.40
125.52
123.65
122.54
122.52
122.38
122.36
122.13
122.10
121.97
121.94
121.78
109.24
109.10
108.97
95.61
95.55
95.48
91.74
77.37 CDCl3
77.16 CDCl3
76.95 CDCl3
51.40
51.19
50.97
50.76
15.16
CF3
H3C
NH
F
I
N
N
N
N
F
Cl
f1 (ppm)

39

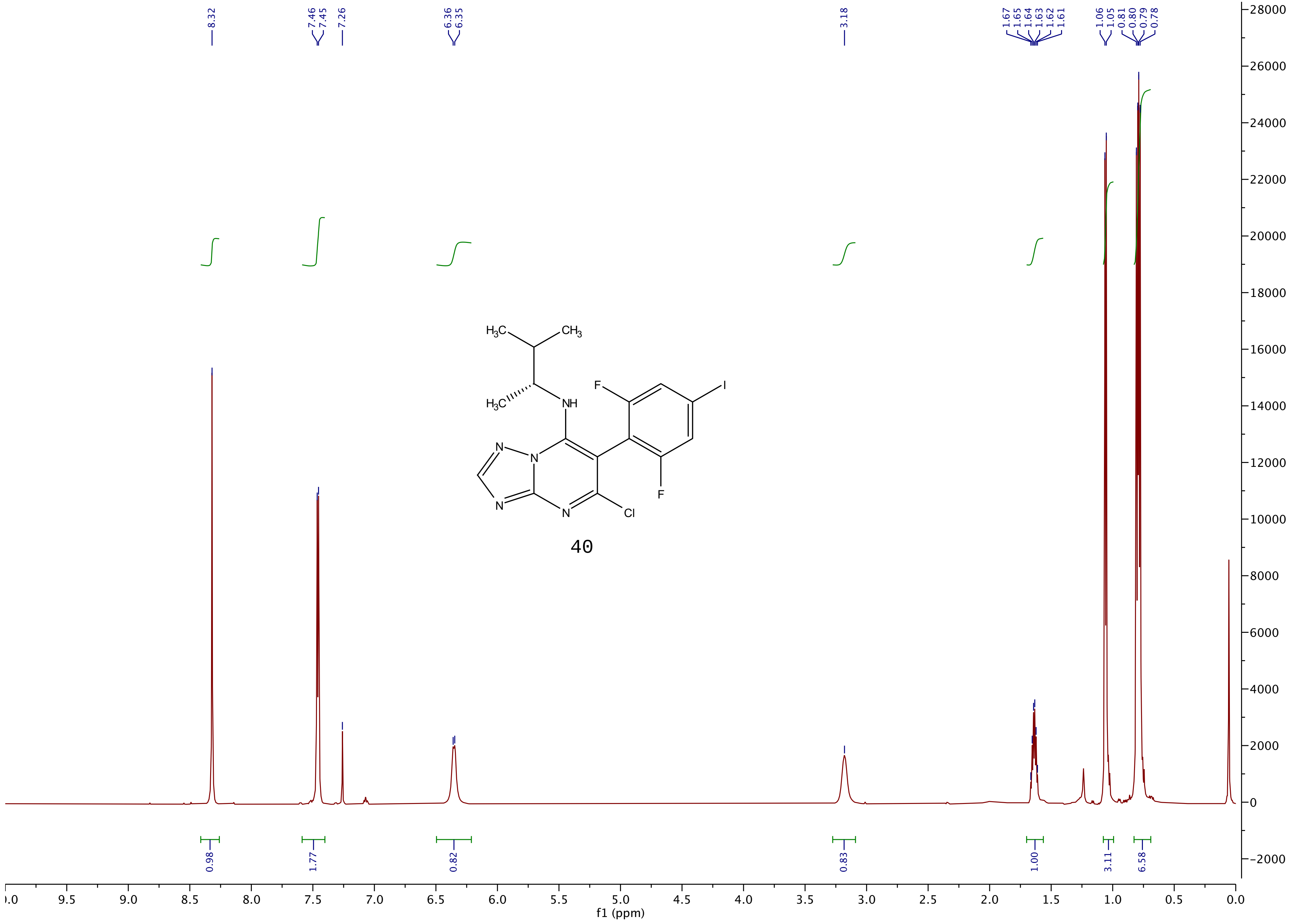
8.32
7.46
7.45
7.26
6.36
6.35
3.18
1.67
1.65
1.64
1.63
1.62
1.61
1.06
1.05
0.81
0.80
0.79
0.78
H3C
CH3
H3C
NH
F
I
F
N
N
N
N
Cl
40
0.98
1.77
0.82
0.83
1.00
3.11
6.58
f1 (ppm)
28000
26000
24000
22000
20000
18000
16000
14000
12000
10000
8000
6000
4000
2000
0
-2000

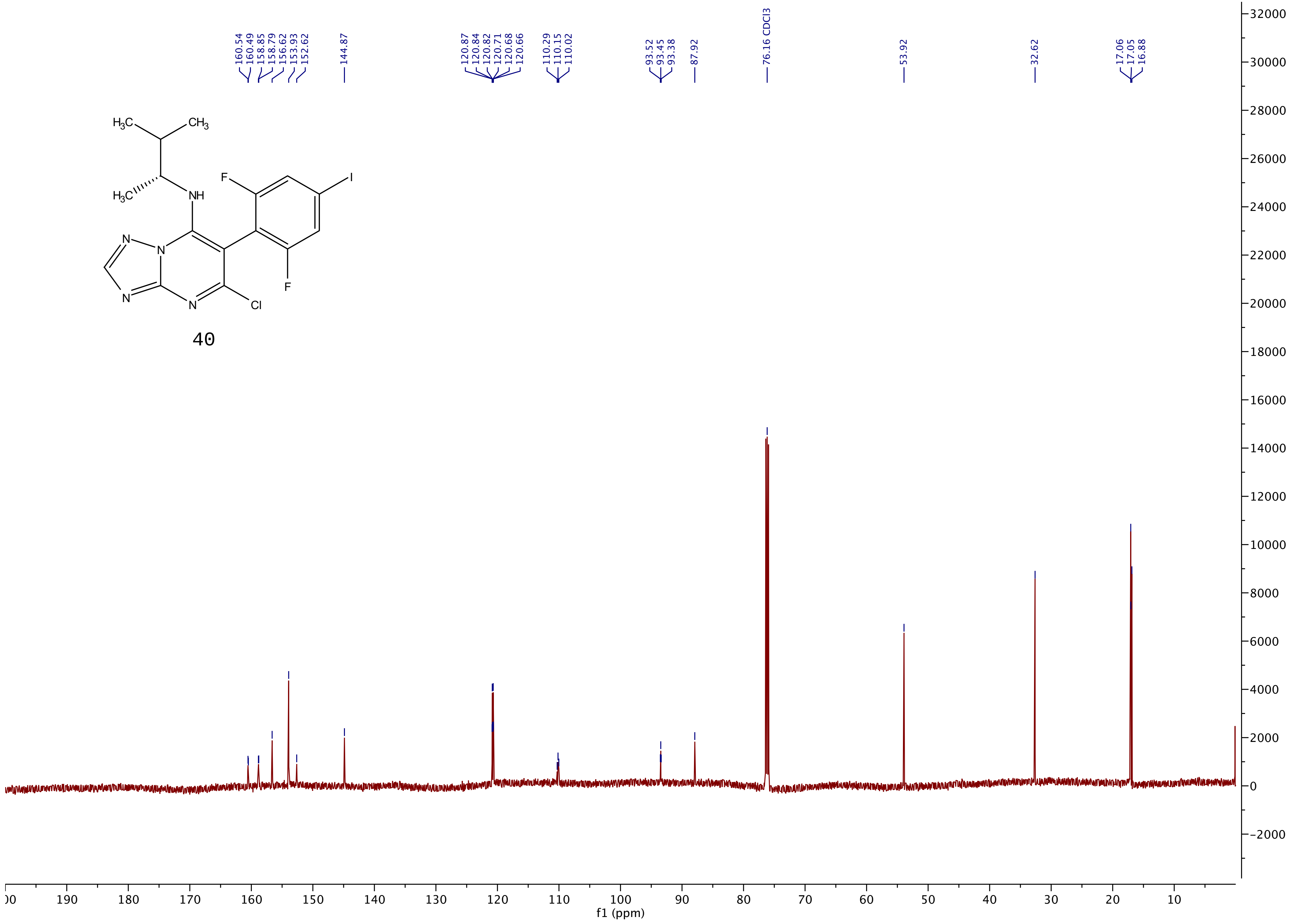
160.54
160.49
158.85
158.79
156.62
153.93
152.62
144.87
120.87
120.84
120.82
120.71
120.68
120.66
110.29
110.15
110.02
93.52
93.45
93.38
87.92
76.16 CDCl3
53.92
32.62
17.06
17.05
16.88
H3C
CH3
H3C
NH
F
I
N
N
N
N
F
Cl
40
f1 (ppm)

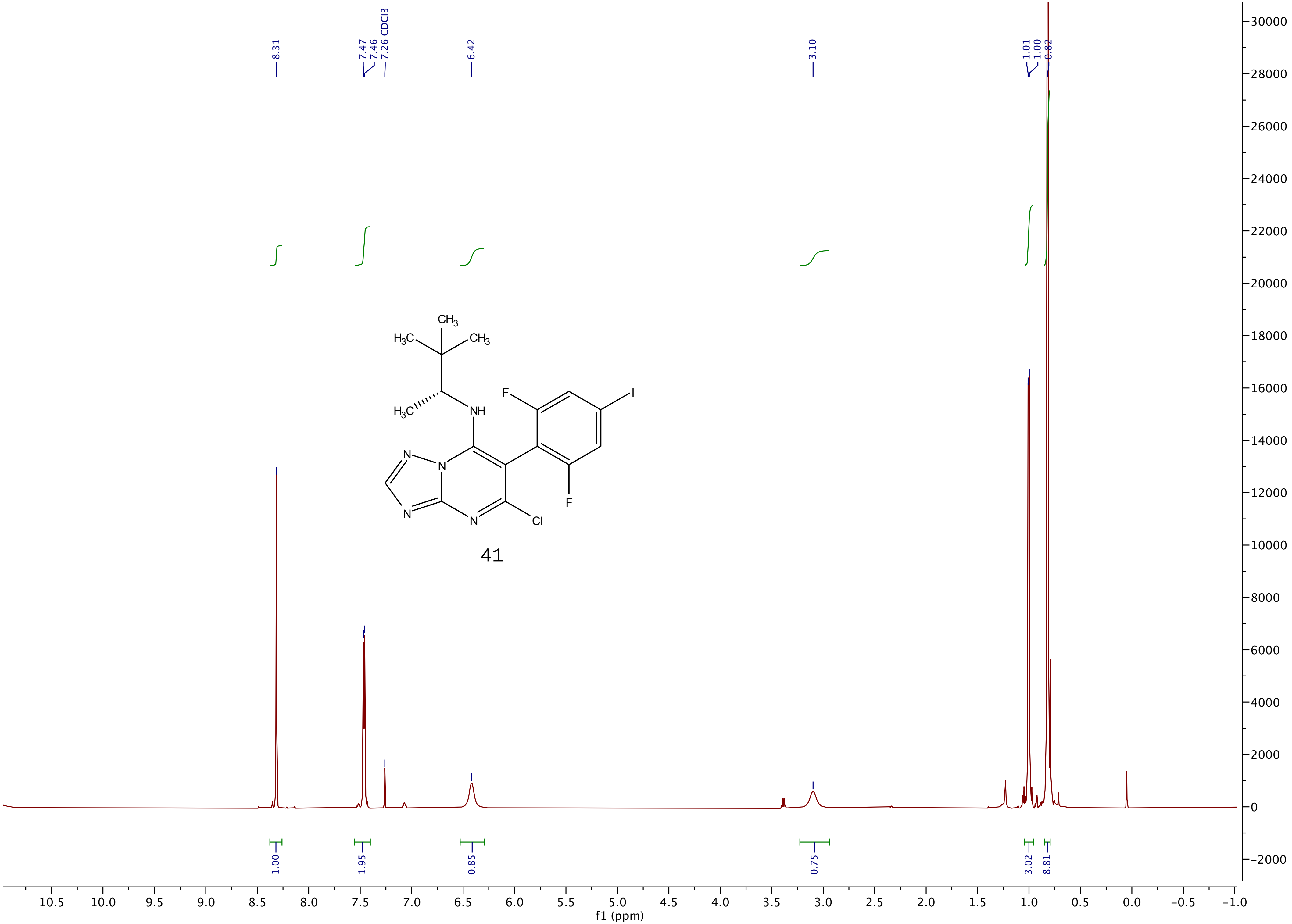
8.31
7.47
7.46
7.26 CDCl3
6.42
3.10
1.01
1.00
0.82
CH3
H3C
CH3
H3C
NH
F
I
F
N
N
N
N
Cl
41
1.00
1.95
0.85
0.75
3.02
8.81
30000
28000
26000
24000
22000
20000
18000
16000
14000
12000
10000
8000
6000
4000
2000
0
−2000
10.5 10.0 9.5 9.0 8.5 8.0 7.5 7.0 6.5 6.0 5.5 5.0 4.5 4.0 3.5 3.0 2.5 2.0 1.5 1.0 0.5 0.0 −0.5 −1.0
f1 (ppm)

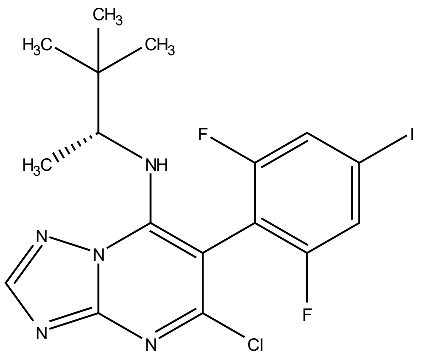

41

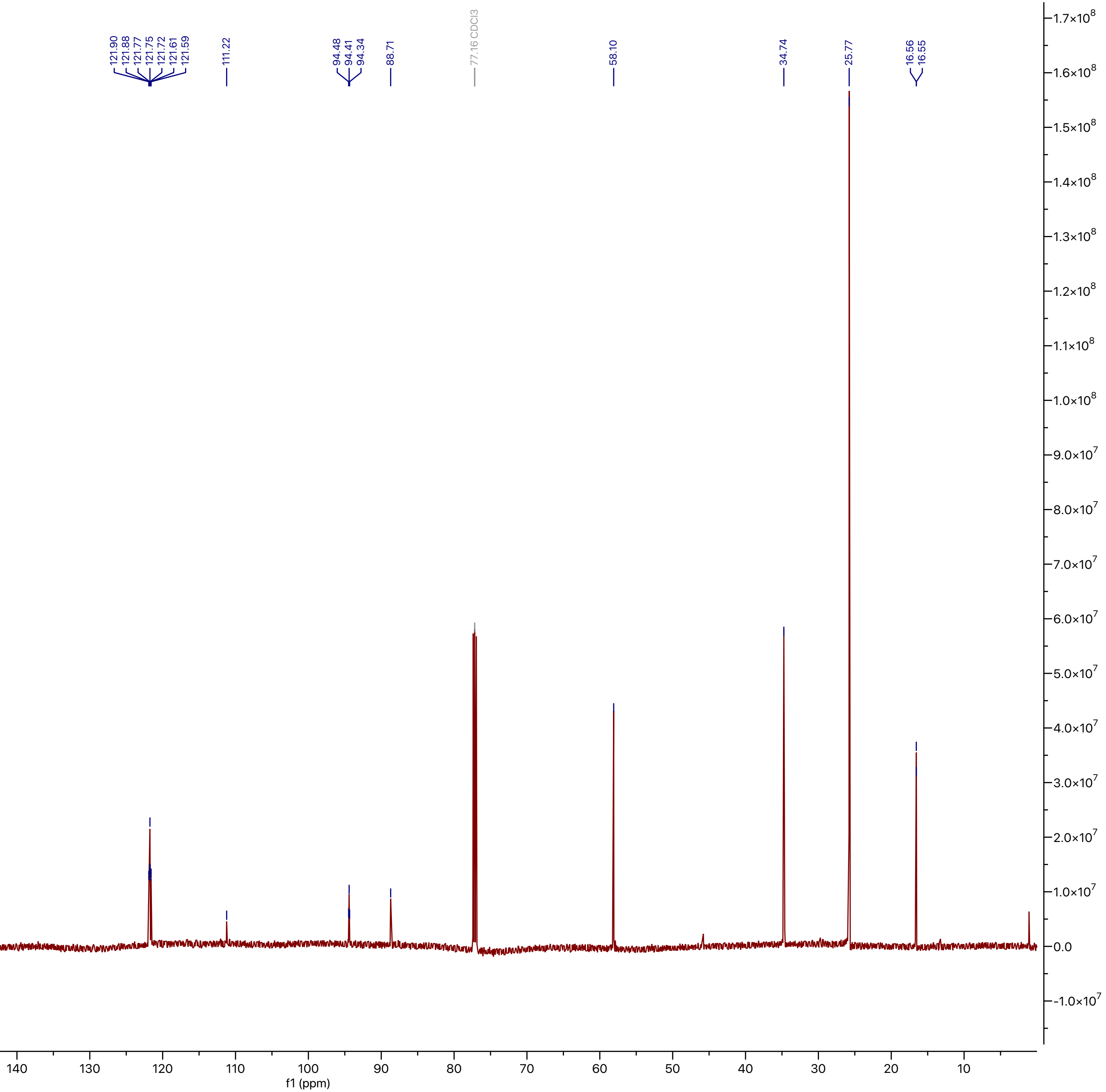
161.54
161.50
161.25
159.81
159.60
159.56
157.58
154.89
153.57
146.03
121.90
121.88
121.77
121.75
121.72
121.61
121.59
111.22
94.48
94.41
94.34
88.71
77.16 CDCl3
58.10
34.74
25.77
16.56
16.55
f1 (ppm)

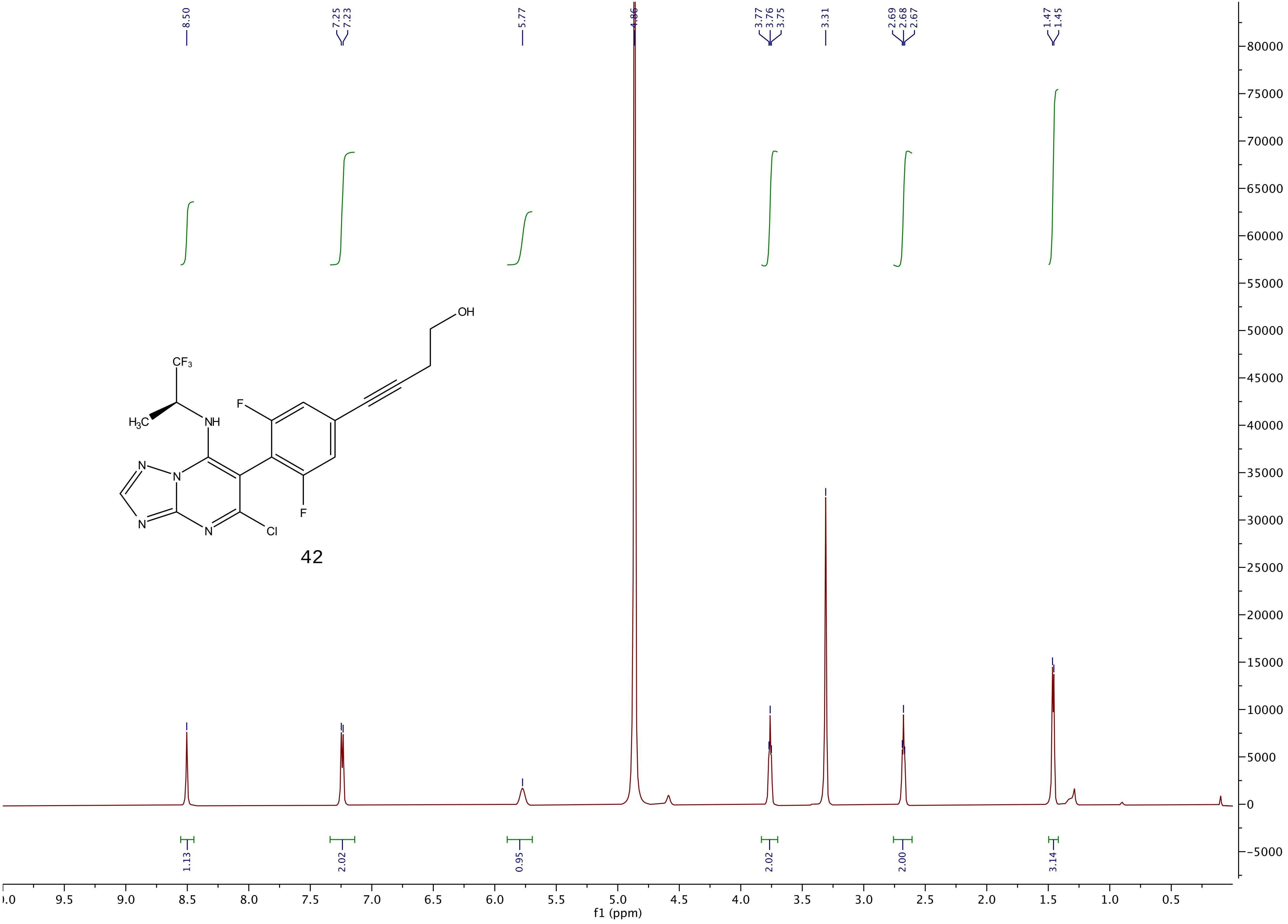
8.50
7.25
7.23
5.77
4.86
3.77
3.76
3.75
3.31
2.69
2.68
2.67
1.47
1.45
OH
CF3
F
H3C
NH
N
N
N
N
F
Cl
42
1.13
2.02
0.95
2.02
2.00
3.14
f1 (ppm)

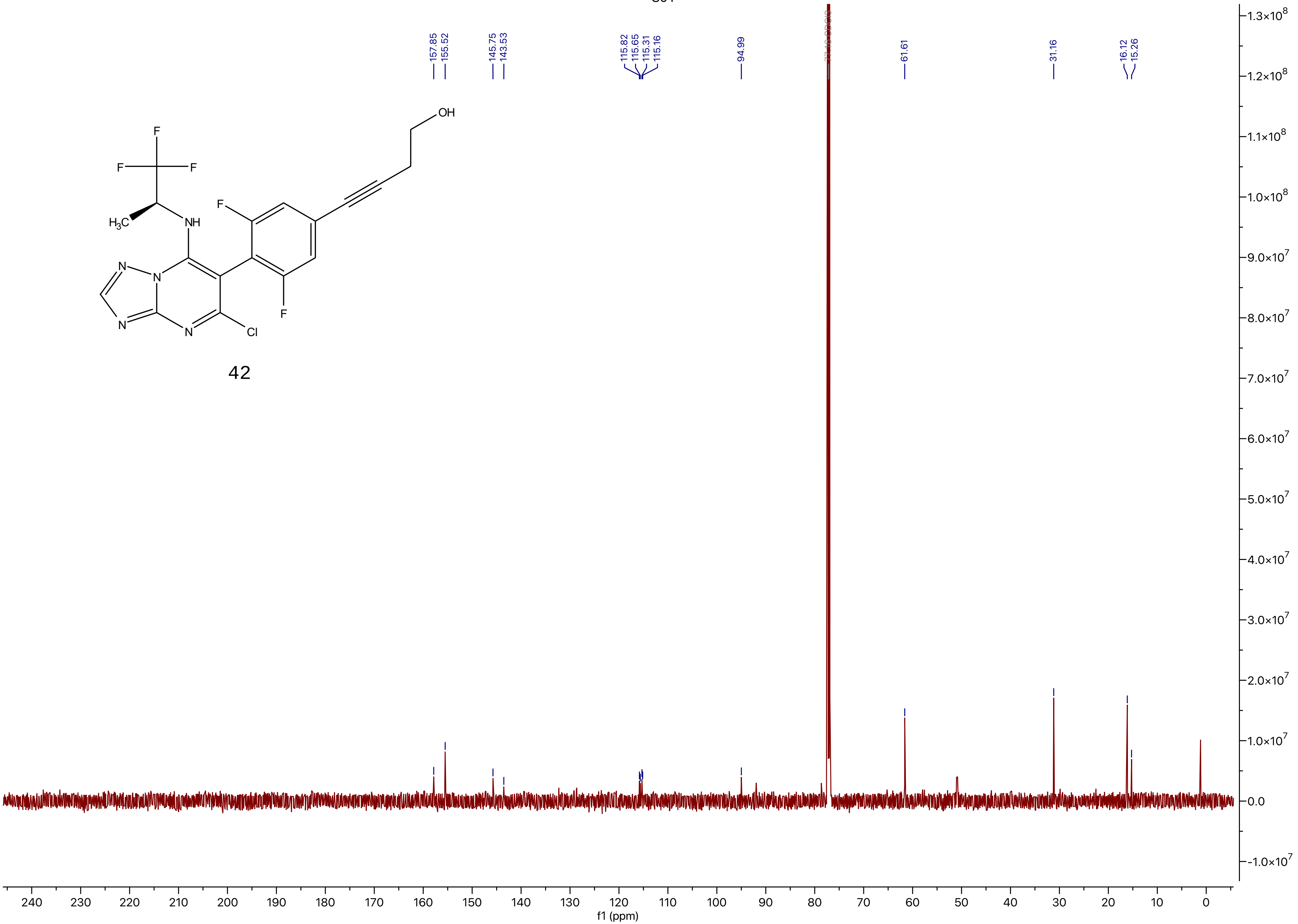

157.85
155.52
145.75
143.53
115.82
115.65
115.31
115.16
94.99
61.61
31.16
16.12
15.26
OH
F
F
F
H3C
NH
F
F
N
N
N
N
Cl
42
f1 (ppm)

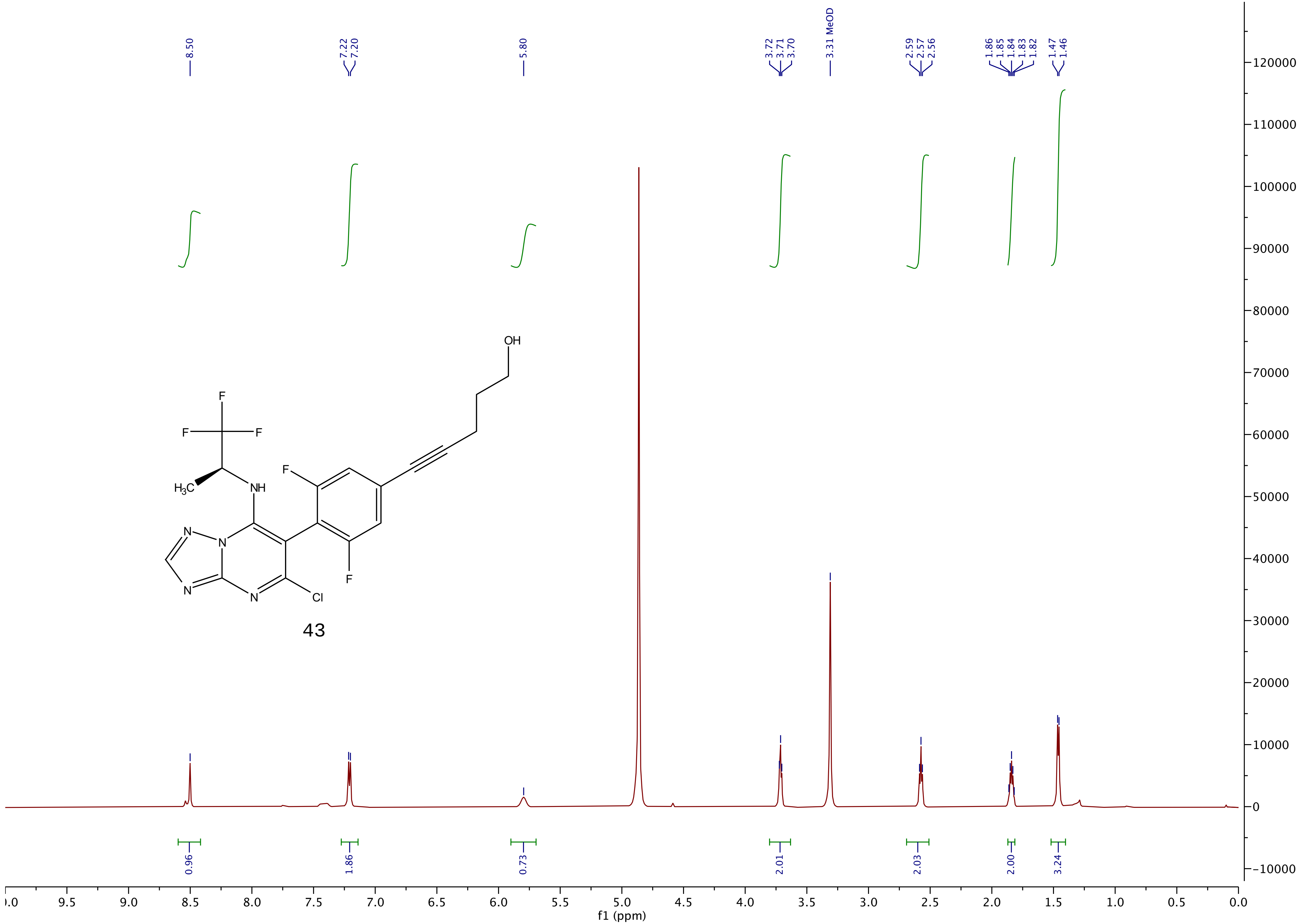
8.50
7.22
7.20
5.80
3.72
3.71
3.70
3.31 MeOD
2.59
2.57
2.56
1.86
1.85
1.84
1.83
1.82
1.47
1.46
OH
F
F
F
F
H3C
NH
N
N
N
N
Cl
F
0.96
1.86
0.73
2.01
2.03
2.00
3.24
f1 (ppm)
120000
110000
100000
90000
80000
70000
60000
50000
40000
30000
20000
10000
0
−10000

43

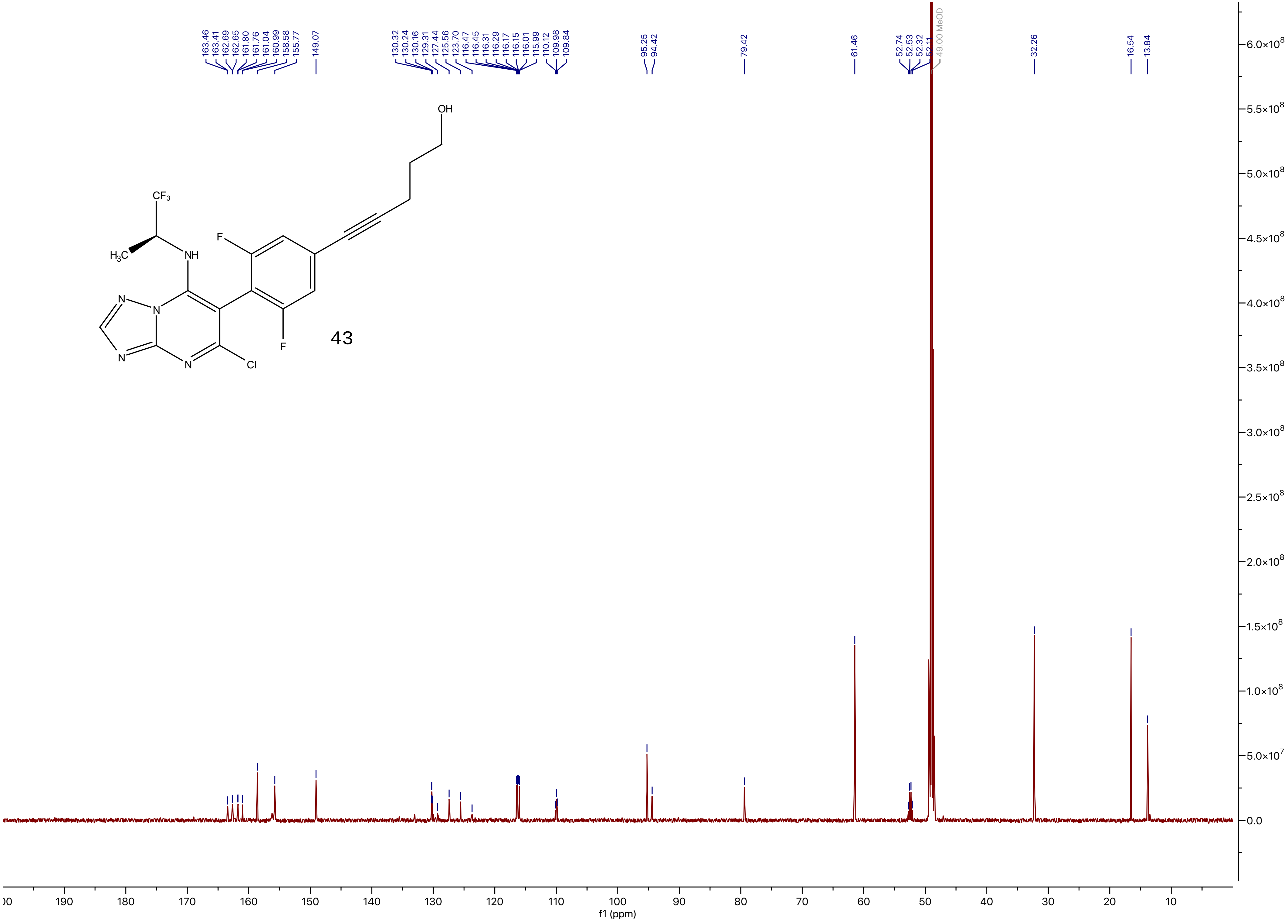
163.46
163.41
162.69
162.65
161.80
161.76
161.04
160.99
158.58
155.77
149.07
130.32
130.24
130.16
129.31
127.44
125.56
123.70
116.47
116.45
116.31
116.29
116.17
116.15
116.01
115.99
110.12
109.98
109.84
95.25
94.42
79.42
61.46
52.74
52.53
52.32
52.11
49.00 MeOD
32.26
16.54
13.84
OH
CF3
F
H3C
NH
N
N
N
N
F
Cl
f1 (ppm)

43

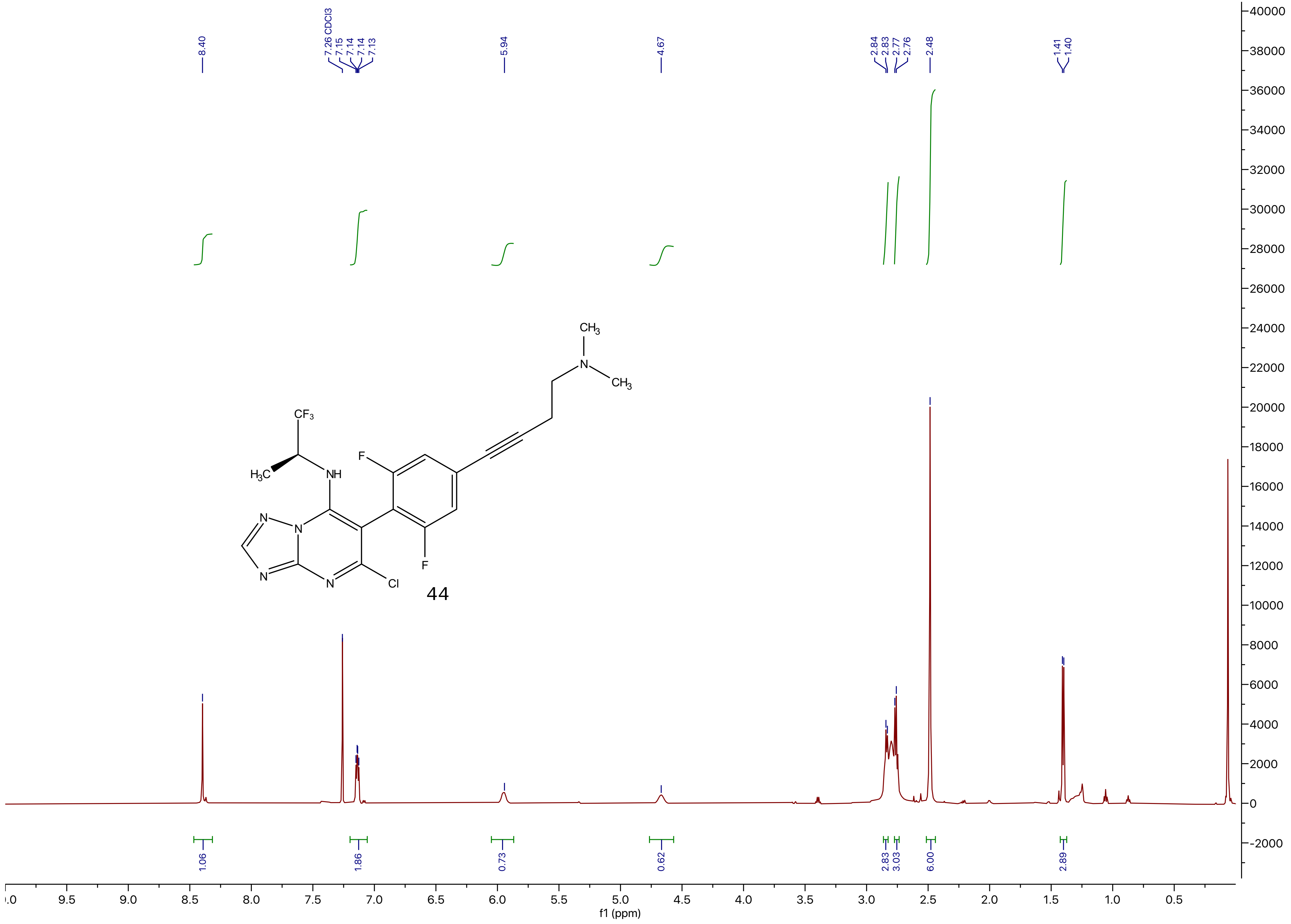
7.26 CDCl3
8.40
7.15
7.14
7.14
7.13
5.94
4.67
2.84
2.83
2.77
2.76
2.48
1.41
1.40
1.06
1.86
0.73
0.62
2.83
3.03
6.00
2.89
CF3
H3C
NH
F
F
N
N
N
N
Cl
CH3
CH3
44
f1 (ppm)

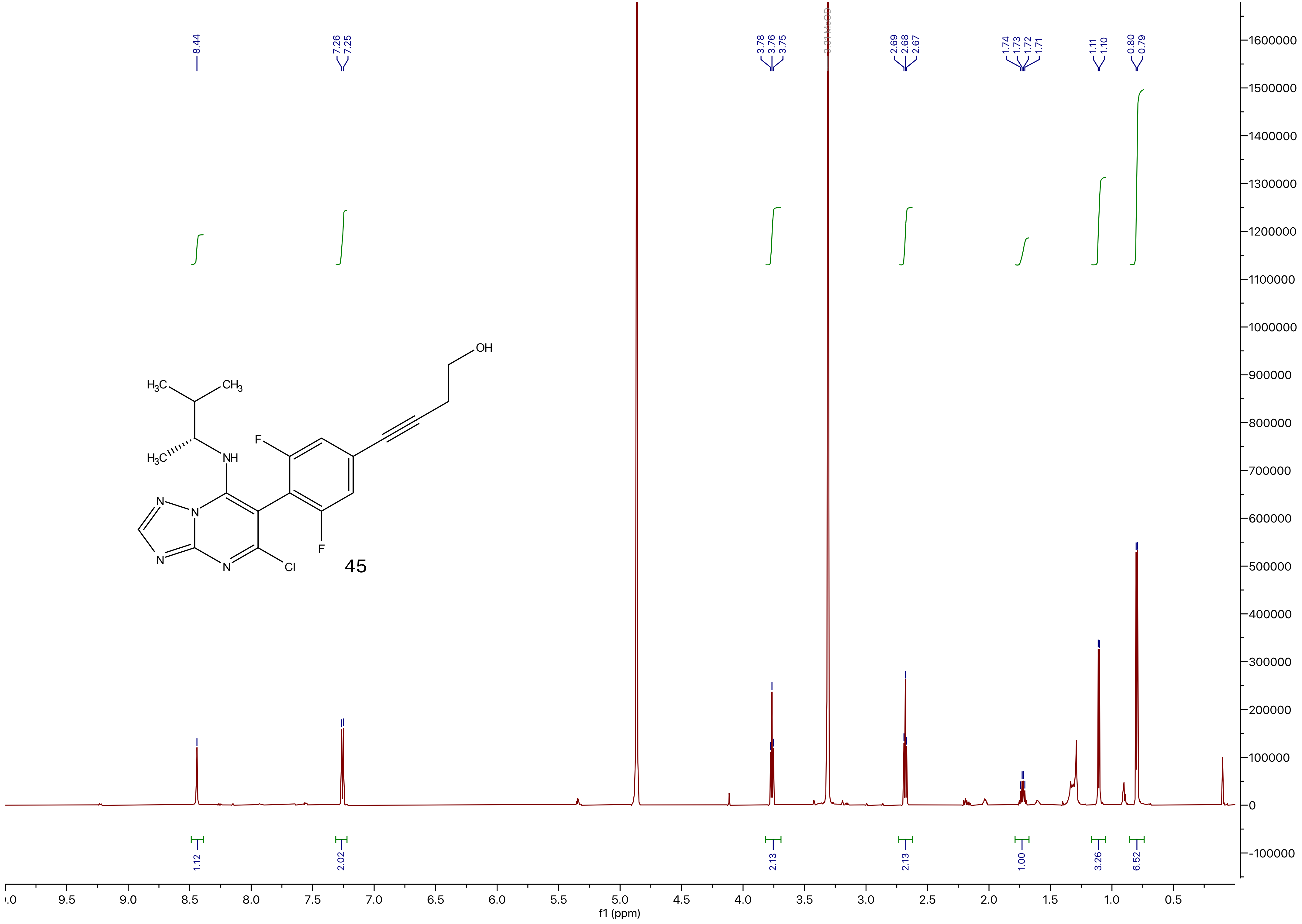

8.44
7.26
7.25
3.78
3.76
3.75
3.31 MeOD
2.69
2.68
2.67
1.74
1.73
1.72
1.71
1.11
1.10
0.80
0.79
OH
H3C
CH3
F
NH
N
Cl
45
1.12
2.02
2.13
2.13
1.00
3.26
6.52
f1 (ppm)

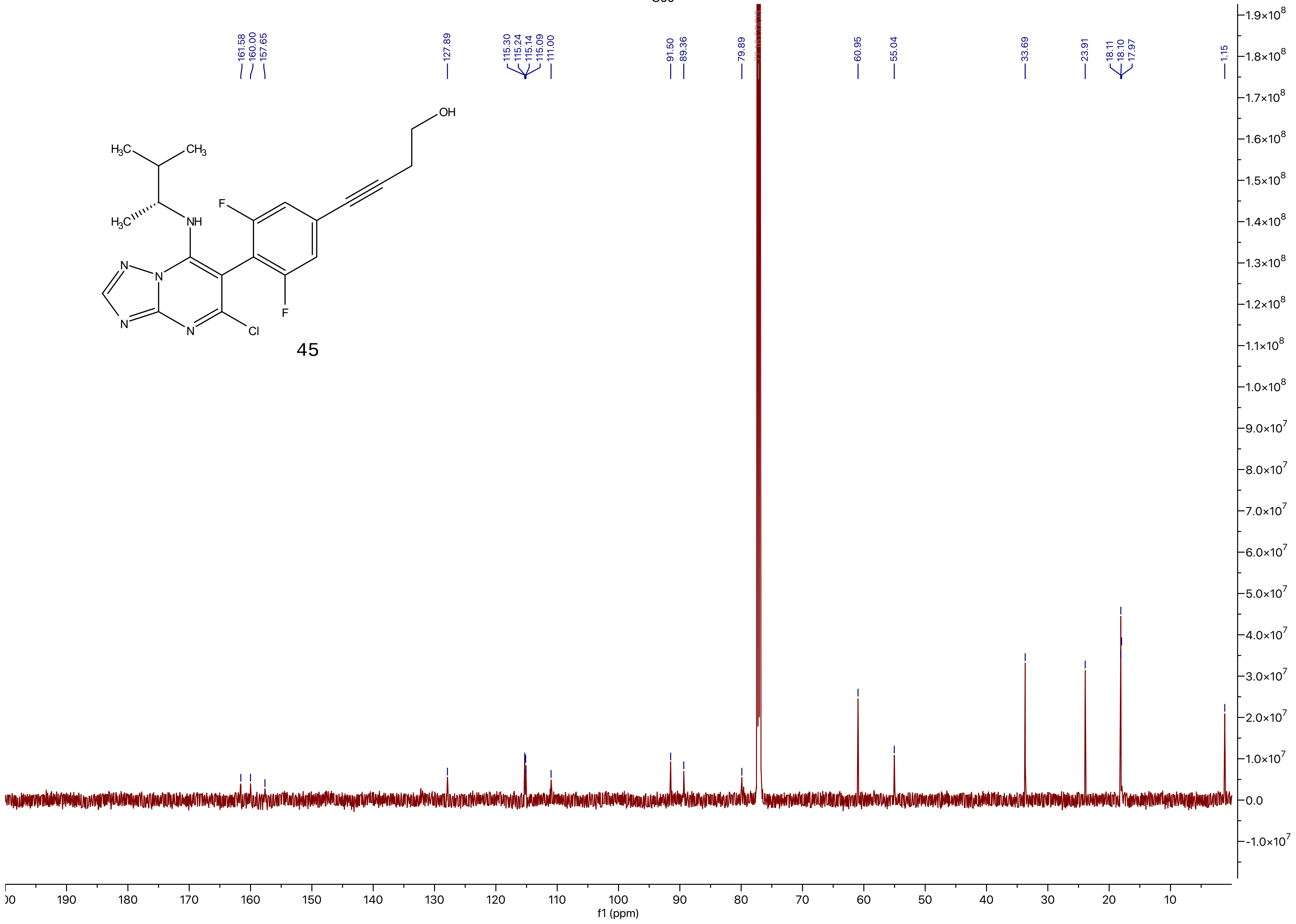
161.58
160.00
157.65
127.89
115.30
115.24
115.14
115.09
111.00
91.50
89.36
79.89
60.95
55.04
33.69
23.91
18.11
18.10
17.97
1.15
OH
H3C
CH3
H3C
NH
F
F
N
N
N
N
Cl
45
f1 (ppm)

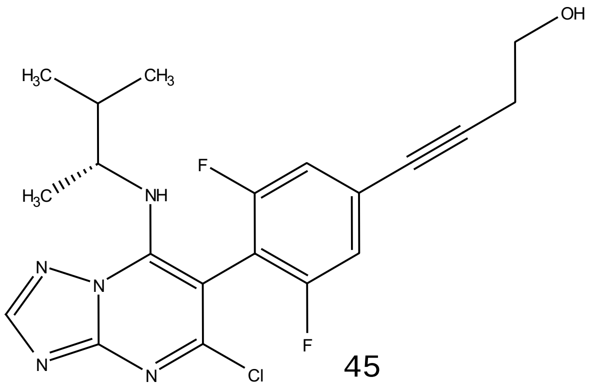
OH
H3C
CH3
F
H3C
NH
N
N
F
N
N
Cl

45

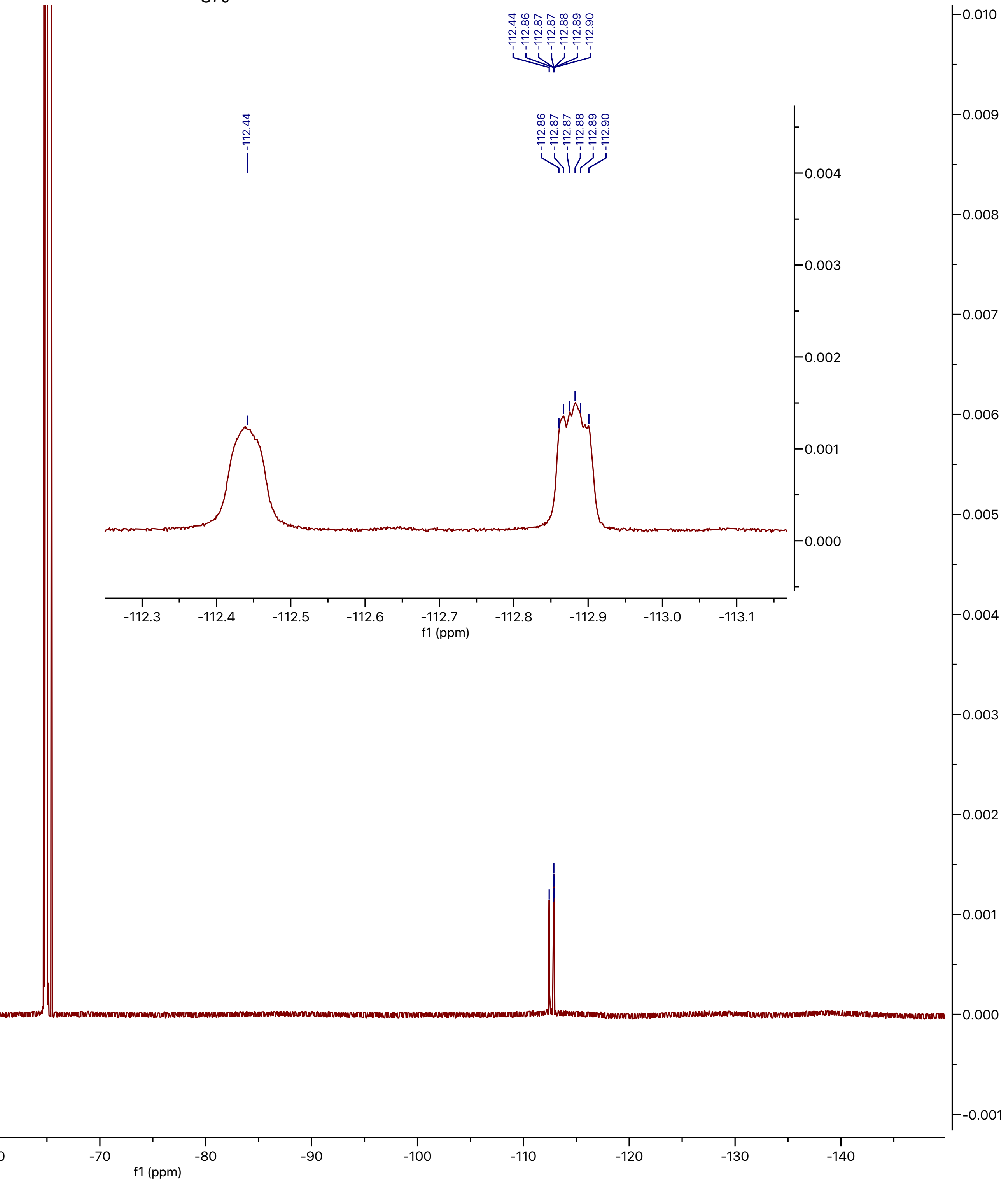
-112.44
-112.86
-112.87
-112.87
-112.88
-112.89
-112.90
f1 (ppm)

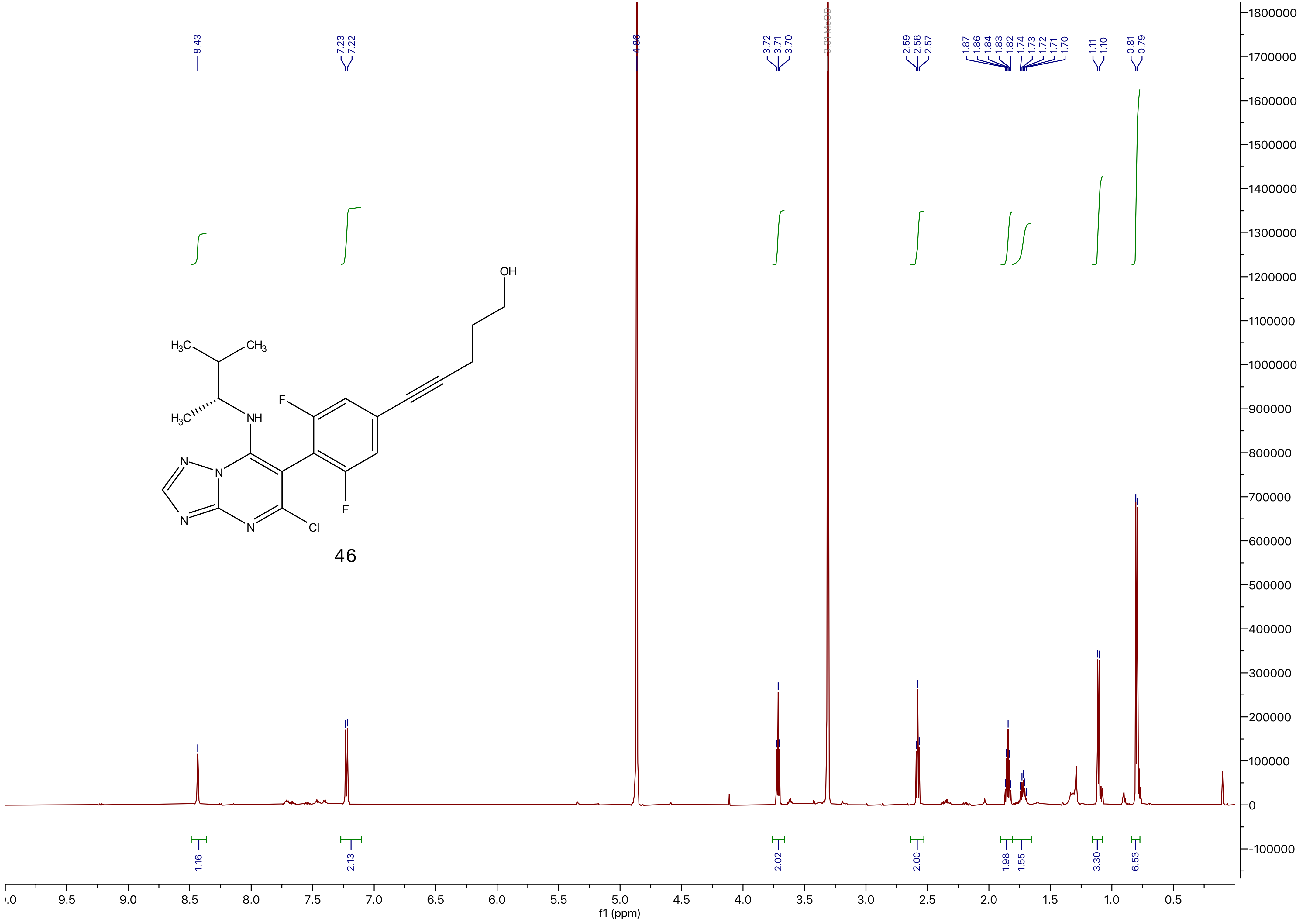

8.43
7.23
7.22
4.86
3.72
3.71
3.70
3.31 MeOD
2.59
2.58
2.57
1.87
1.86
1.84
1.83
1.82
1.74
1.73
1.72
1.71
1.70
1.11
1.10
0.81
0.79
1.16
2.13
2.02
2.00
1.98
1.55
3.30
6.53
OH
H3C
CH3
H3C
NH
F
F
N
N
N
N
Cl
46
f1 (ppm)

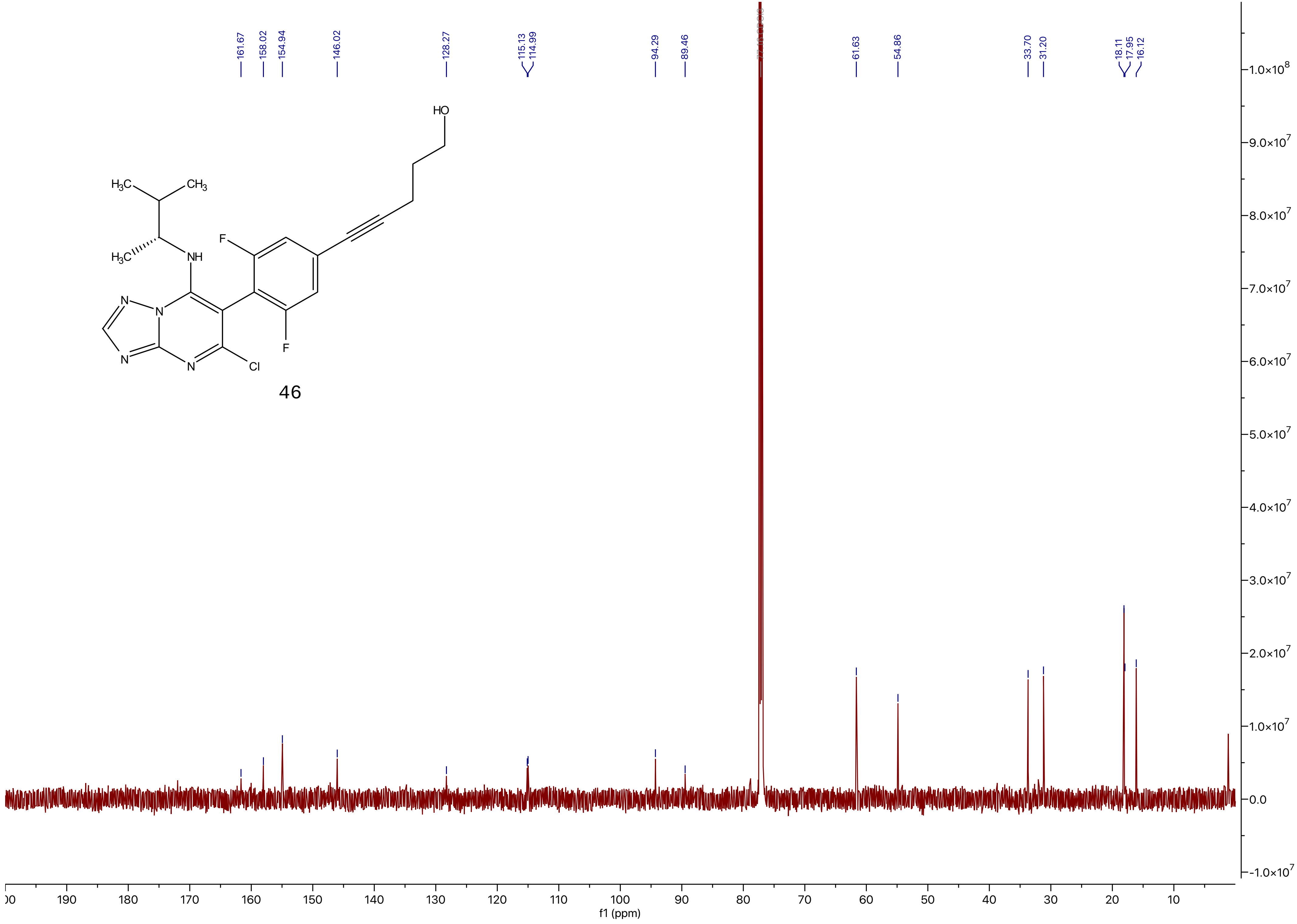
161.67
158.02
154.94
146.02
128.27
115.13
114.99
94.29
89.46
61.63
54.86
33.70
31.20
18.11
17.95
16.12
HO
H_3C
CH_3
H_3C
NH
F
F
N
N
N
N
Cl
1.0×10^8
9.0×10^7
8.0×10^7
7.0×10^7
6.0×10^7
5.0×10^7
4.0×10^7
3.0×10^7
2.0×10^7
1.0×10^7
0.0
−1.0×10^7
190
180
170
160
150
140
130
120
110
100
90
80
70
60
50
40
30
20
10
f1 (ppm)

46

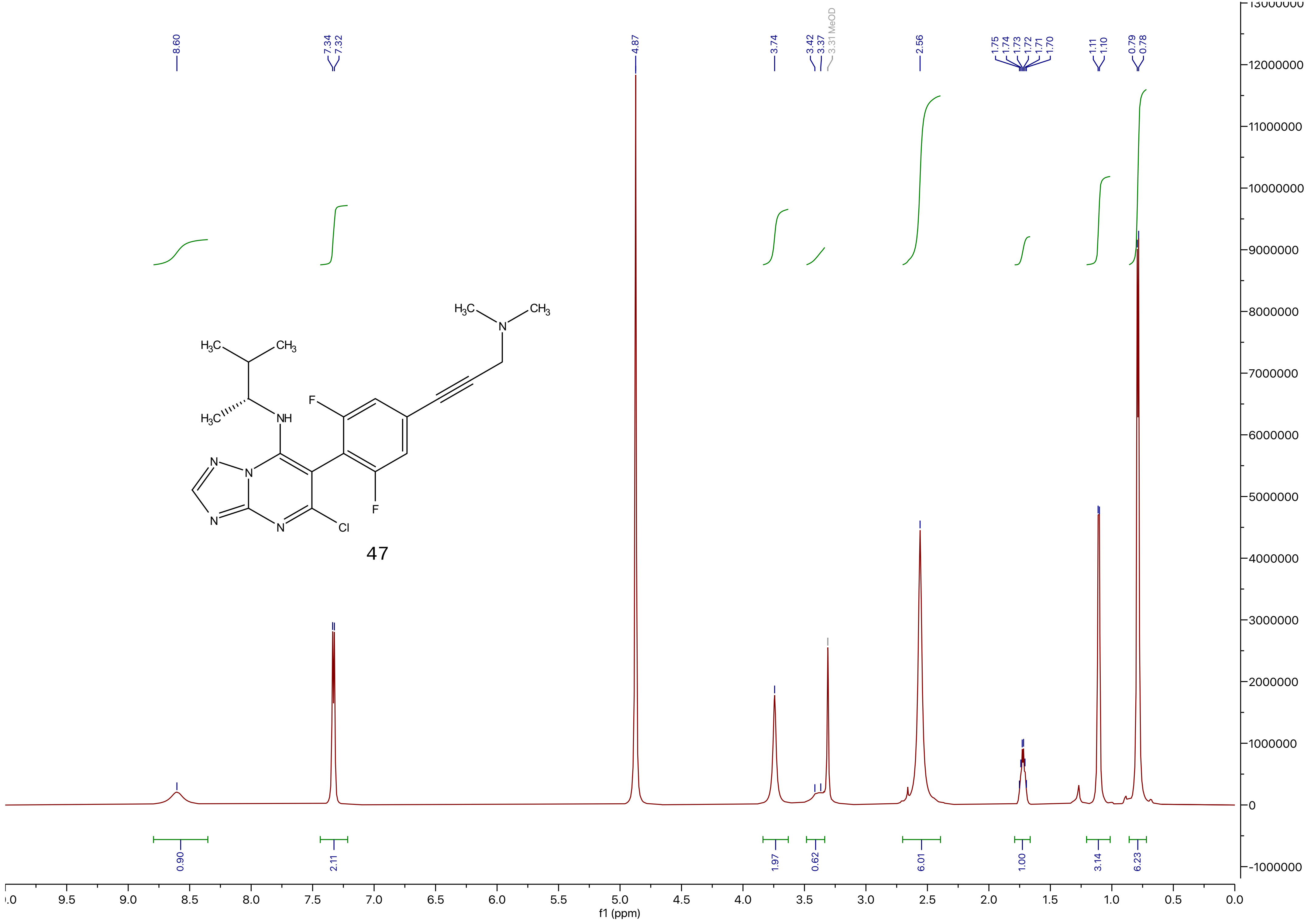
8.60
7.34
7.32
4.87
3.74
3.42
3.37
3.31 MeOD
2.56
1.75
1.74
1.73
1.72
1.71
1.70
1.11
1.10
0.79
0.78
H3C
CH3
N
NH
F
Cl
47
0.90
2.11
1.97
0.62
6.01
1.00
3.14
6.23
f1 (ppm)

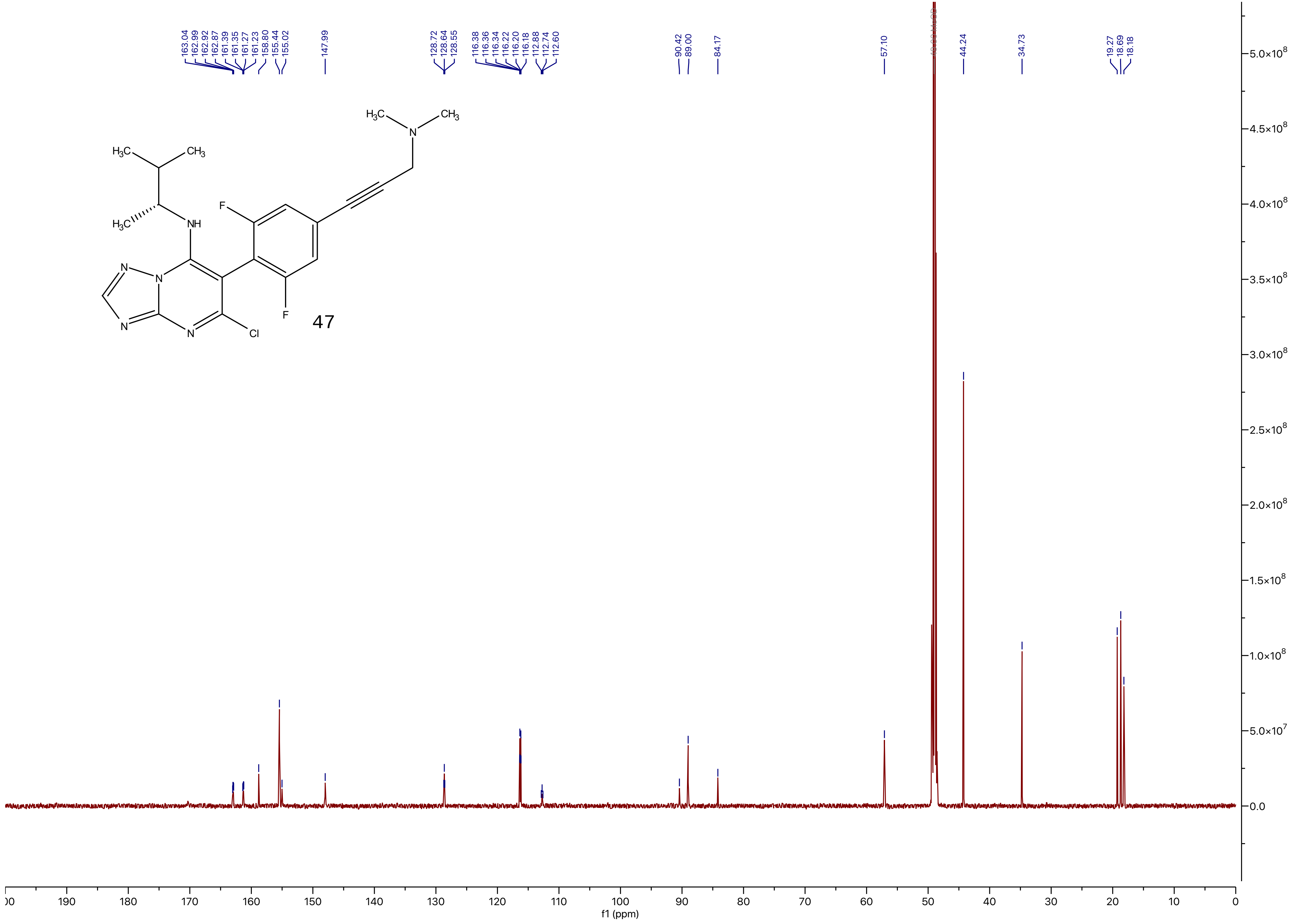
163.04
162.99
162.92
162.87
161.39
161.35
161.27
161.23
158.80
155.44
155.02
147.99
128.72
128.64
128.55
116.38
116.36
116.34
116.22
116.20
116.18
112.88
112.74
112.60
90.42
89.00
84.17
57.10
44.24
34.73
19.27
18.69
18.18
H3C
CH3
N
F
NH
Cl
f1 (ppm)

47

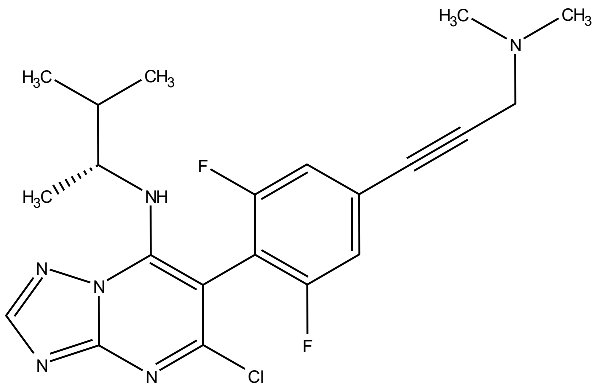

47

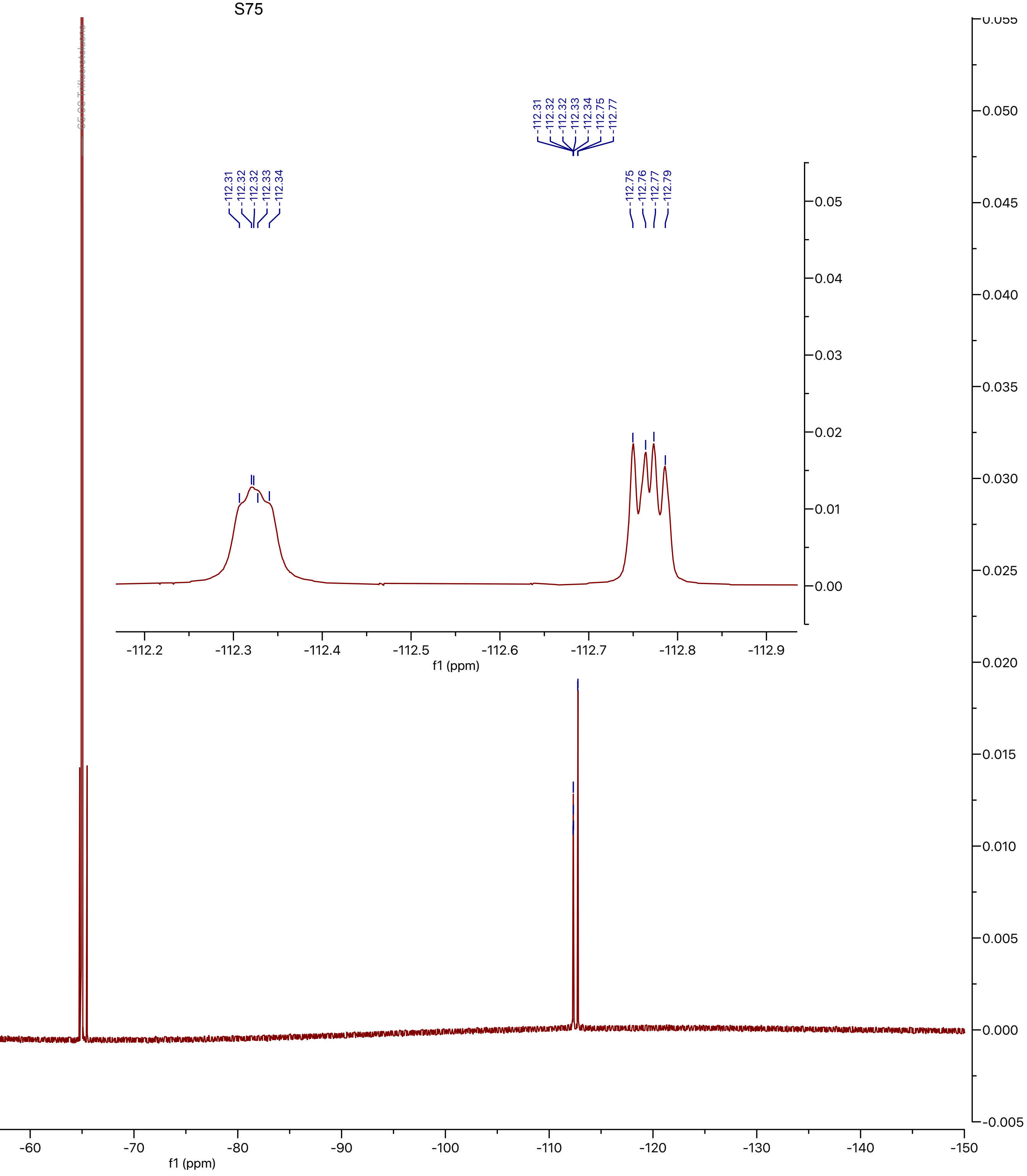
-112.31
-112.32
-112.32
-112.33
-112.34
-112.75
-112.77
-112.31
-112.32
-112.32
-112.33
-112.34
-112.75
-112.76
-112.77
-112.79
f1 (ppm)
f1 (ppm)

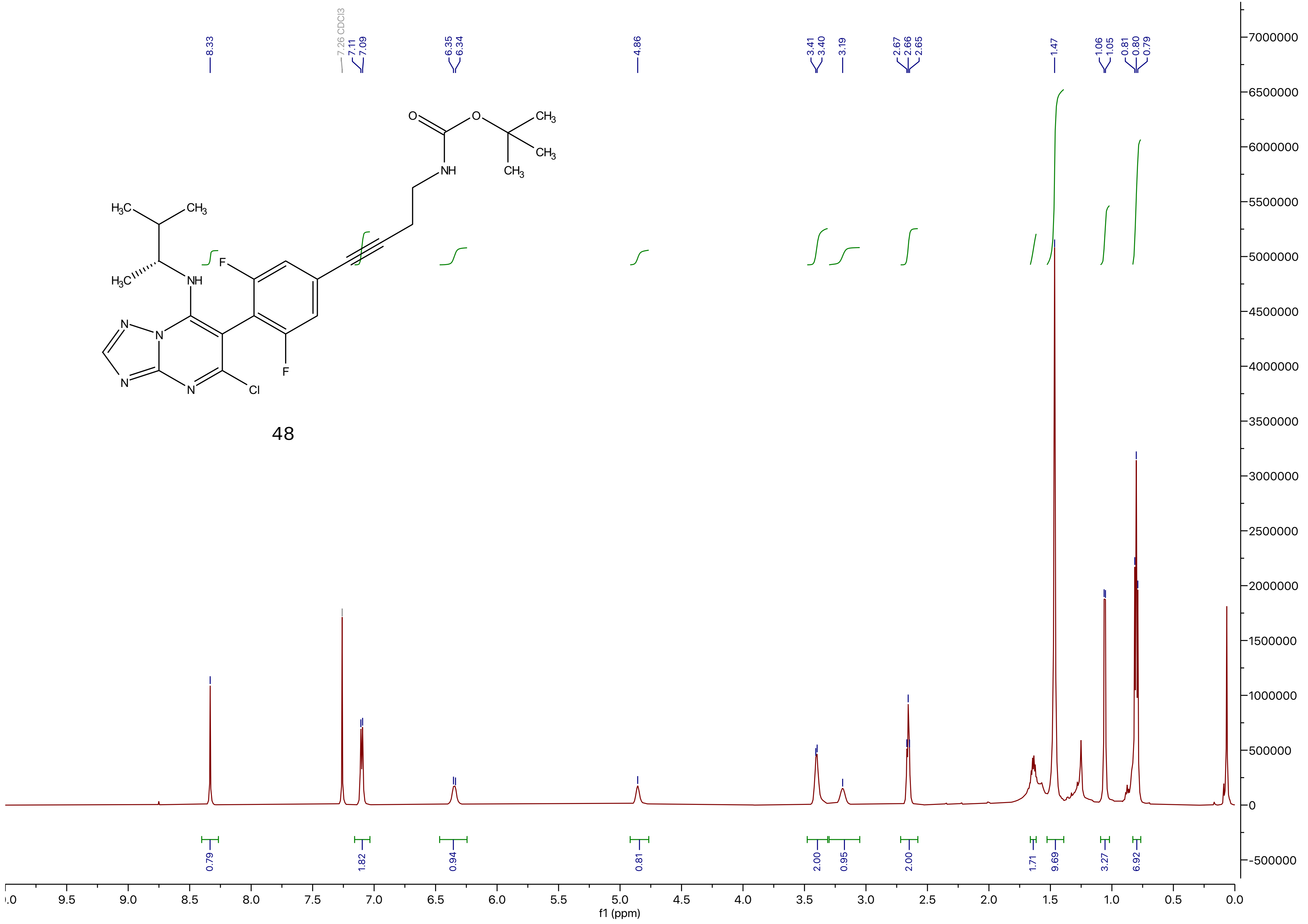

48
8.33
7.26 CDCl3
7.11
7.09
6.35
6.34
4.86
3.41
3.40
3.19
2.67
2.66
2.65
1.47
1.06
1.05
0.81
0.80
0.79
0.79
1.82
0.94
0.81
2.00
0.95
2.00
1.71
9.69
3.27
6.92
f1 (ppm)

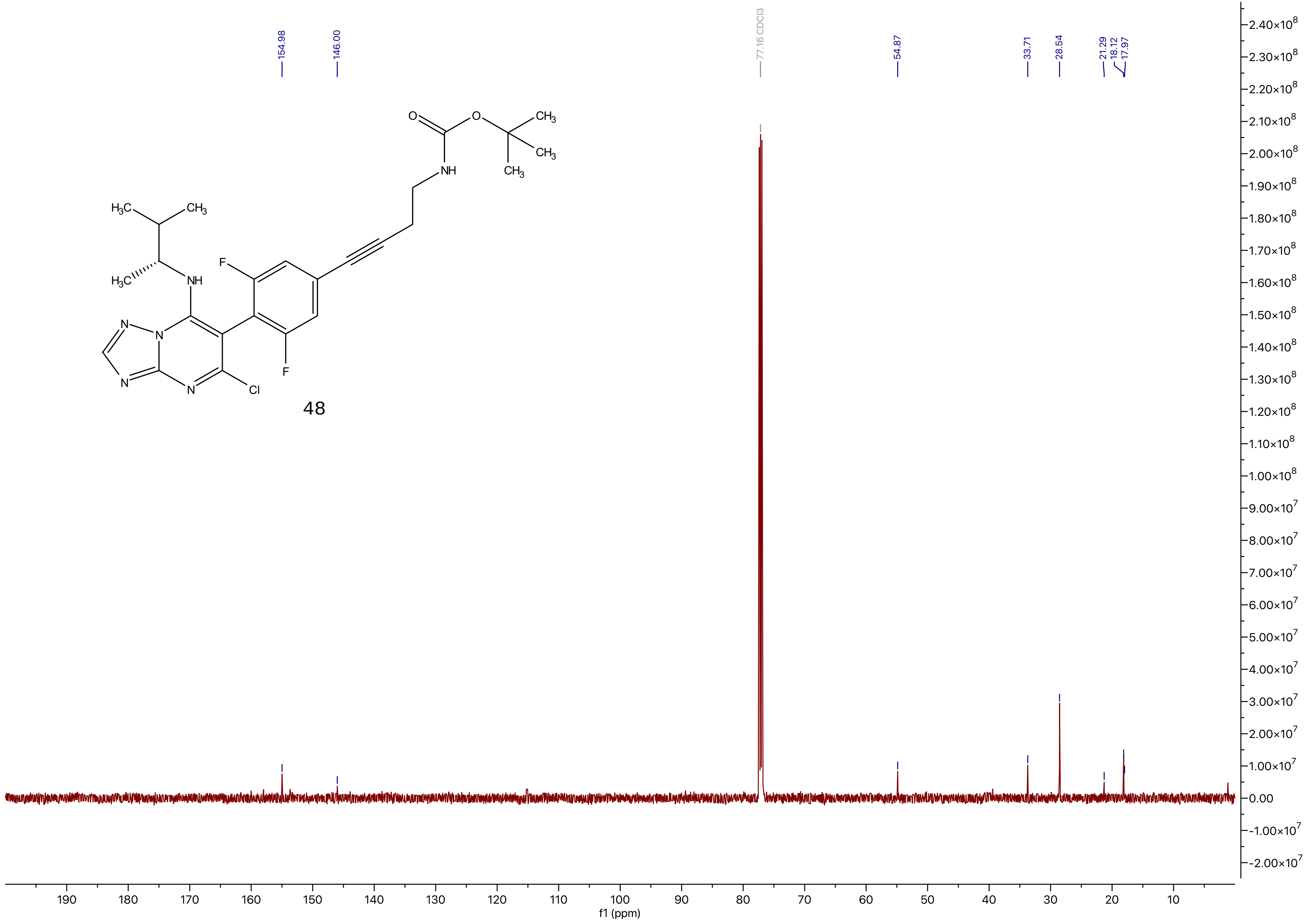
154.98
146.00
77.16 CDCl3
54.87
33.71
28.54
21.29
18.12
17.97
48
f1 (ppm)

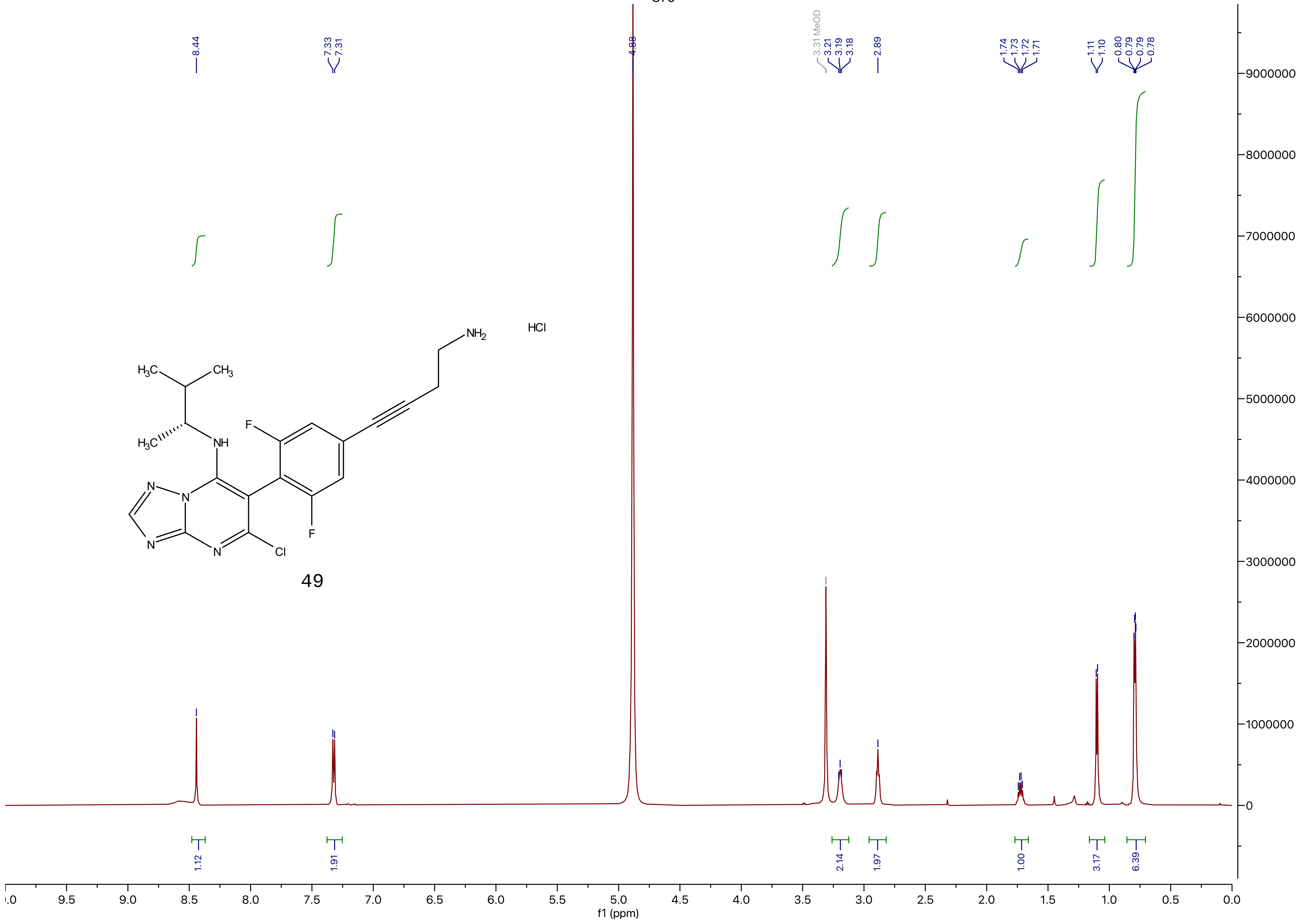
8.44
7.33
7.31
4.88
3.31 MeOD
3.21
3.19
3.18
2.89
1.74
1.73
1.72
1.71
1.11
1.10
0.80
0.79
0.79
0.78
HCl
49
1.12
1.91
2.14
1.97
1.00
3.17
6.39
f1 (ppm)
9000000
8000000
7000000
6000000
5000000
4000000
3000000
2000000
1000000
0

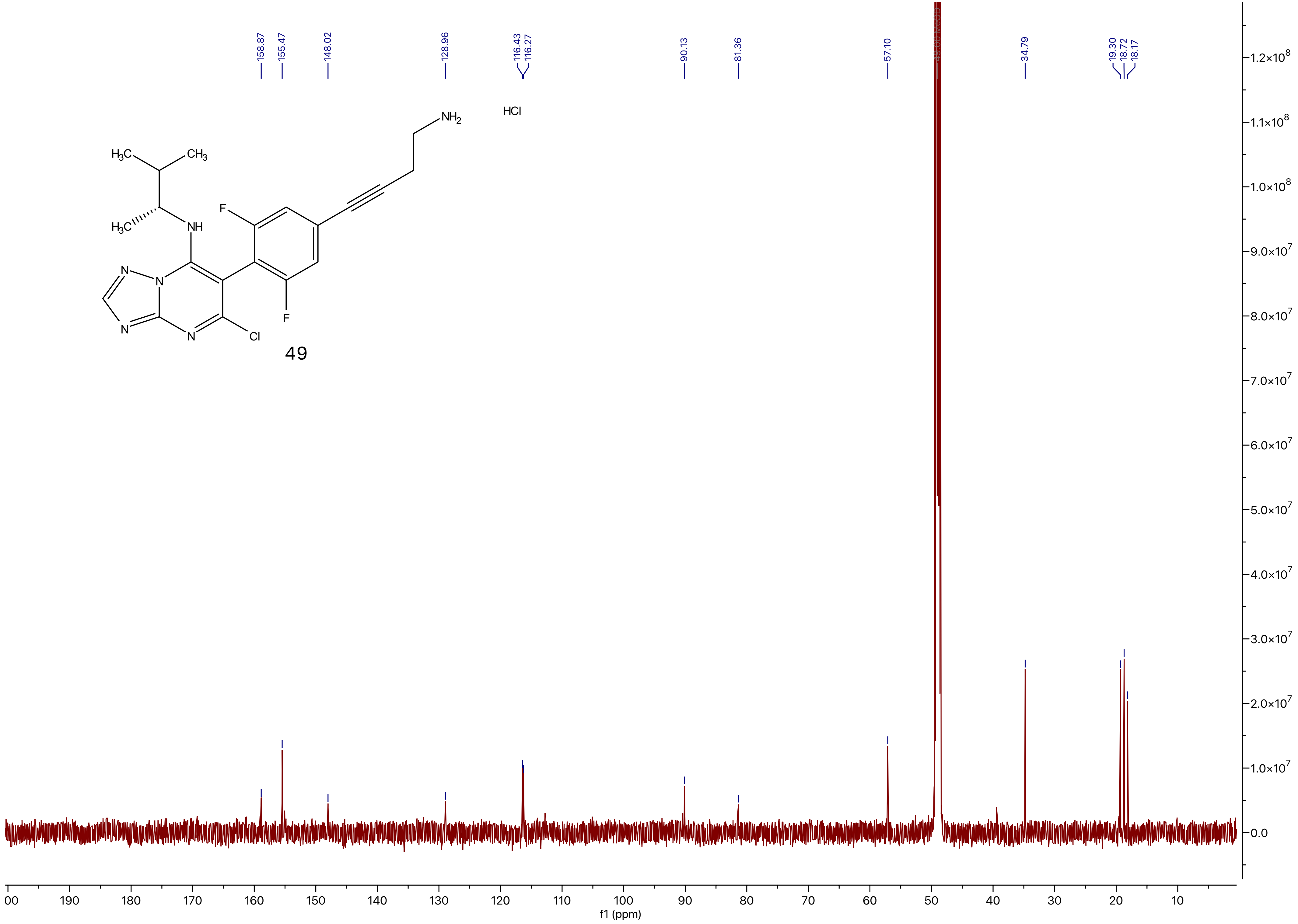
158.87
155.47
148.02
128.96
116.43
116.27
90.13
81.36
57.10
34.79
19.30
18.72
18.17
HCl
49
f1 (ppm)

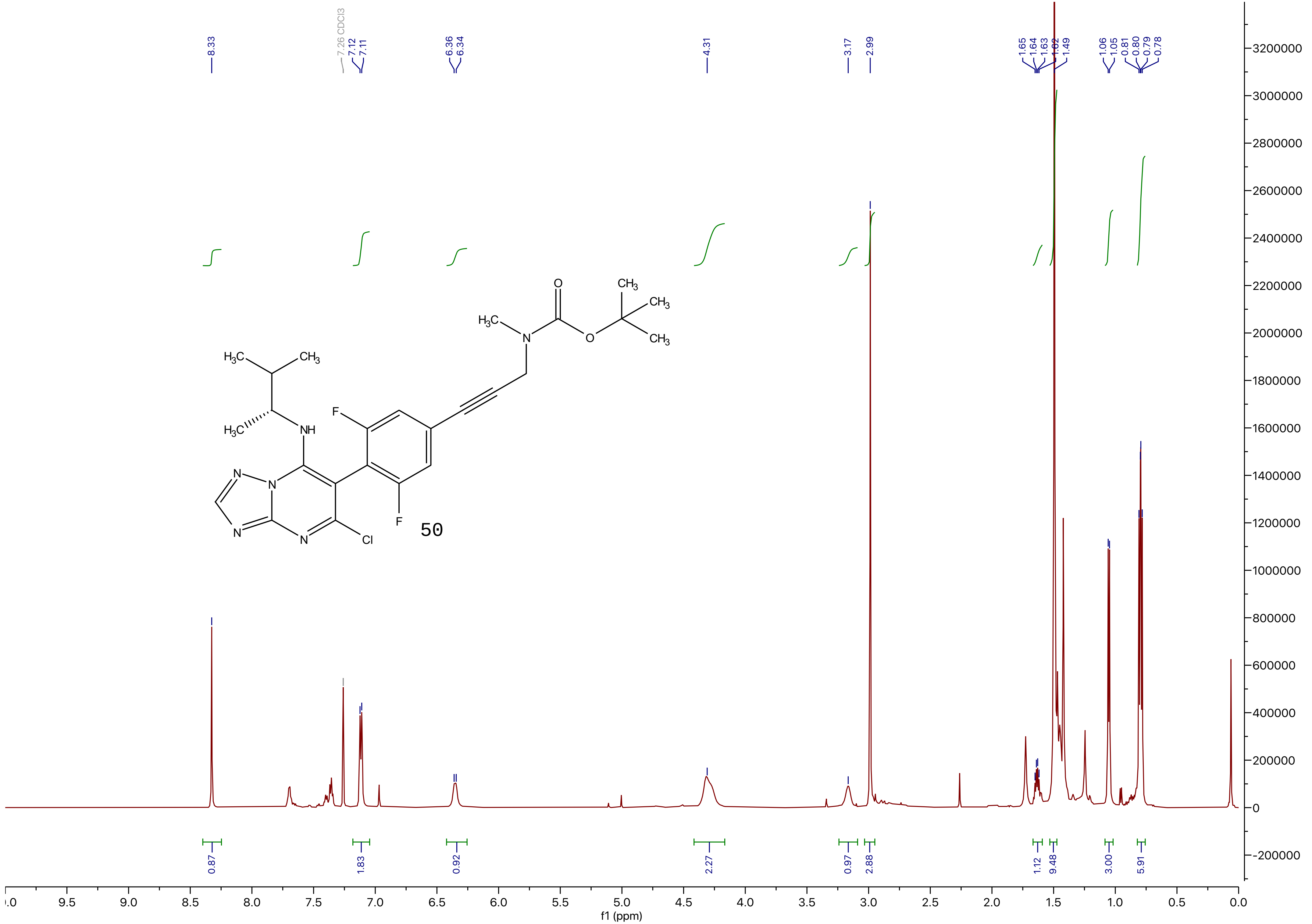
8.33
7.26 CDCl3
7.12
7.11
6.36
6.34
4.31
3.17
2.99
1.65
1.64
1.63
1.62
1.49
1.06
1.05
0.81
0.80
0.79
0.78
0.87
1.83
0.92
2.27
0.97
2.88
1.12
9.48
3.00
5.91
50
f1 (ppm)

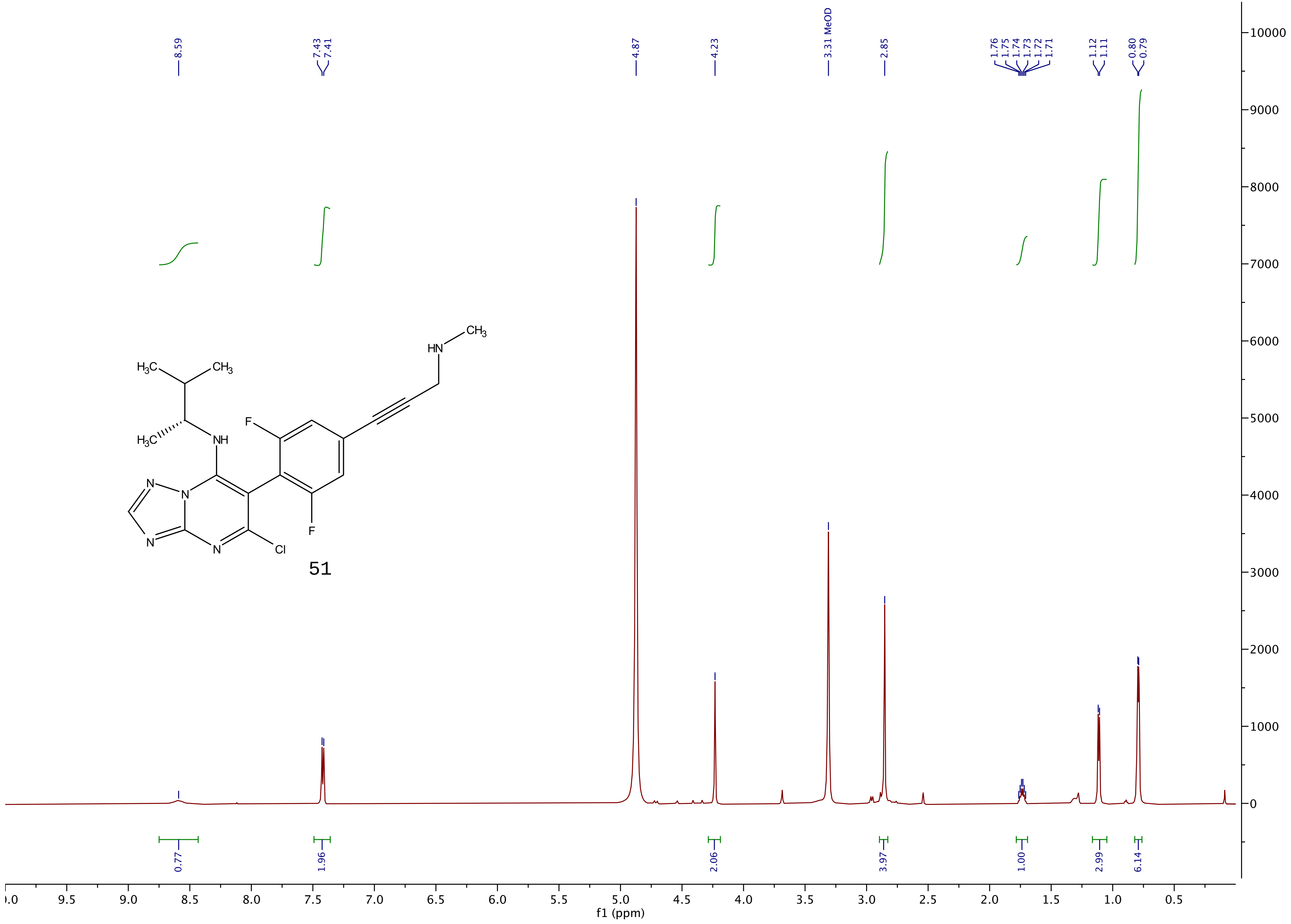

8.59
7.43
7.41
4.87
4.23
3.31 MeOD
2.85
1.76
1.75
1.74
1.73
1.72
1.71
1.12
1.11
0.80
0.79
H3C
CH3
H3C
NH
F
F
HN
CH3
N
N
N
N
Cl
51
0.77
1.96
2.06
3.97
1.00
2.99
6.14
f1 (ppm)

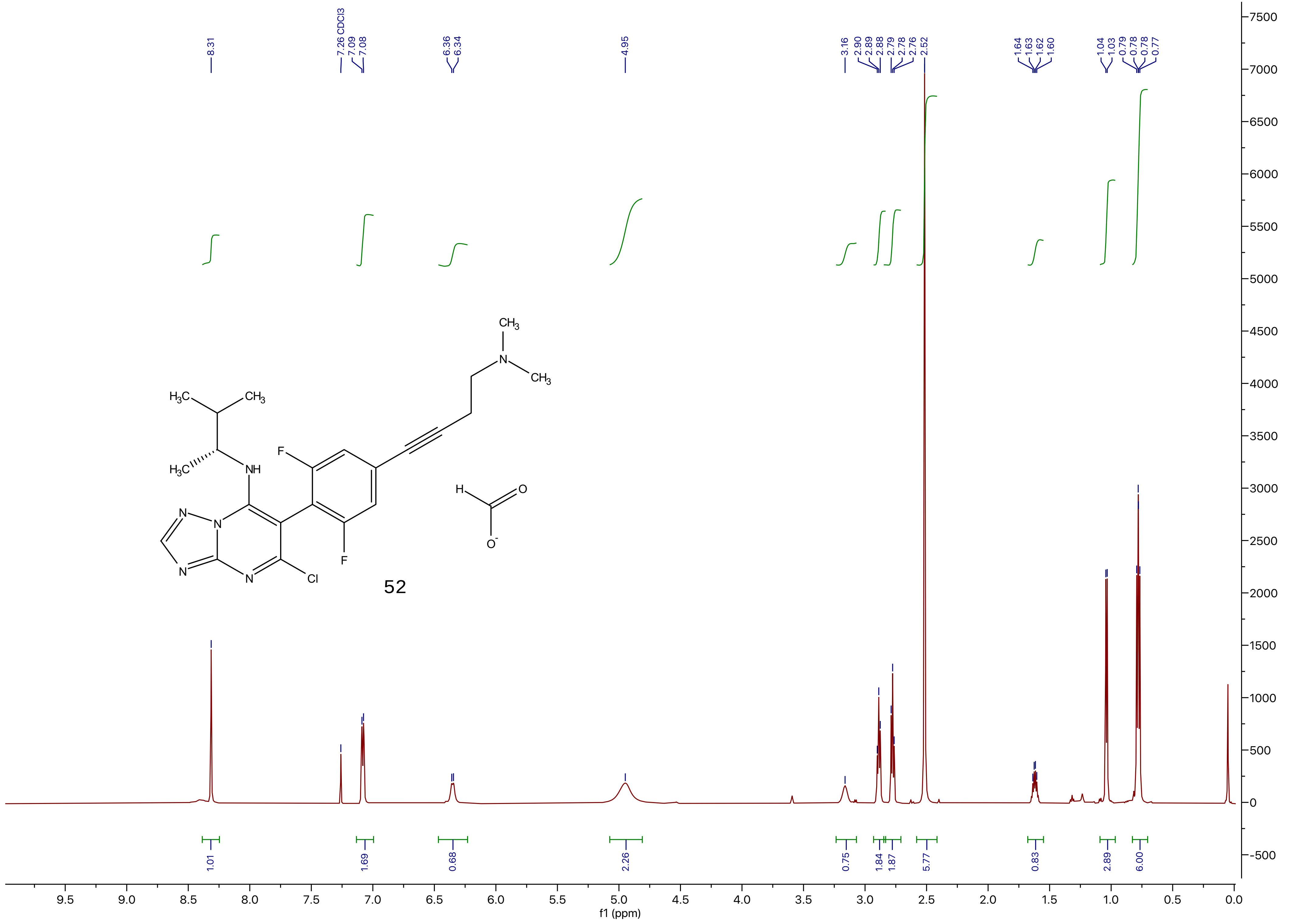

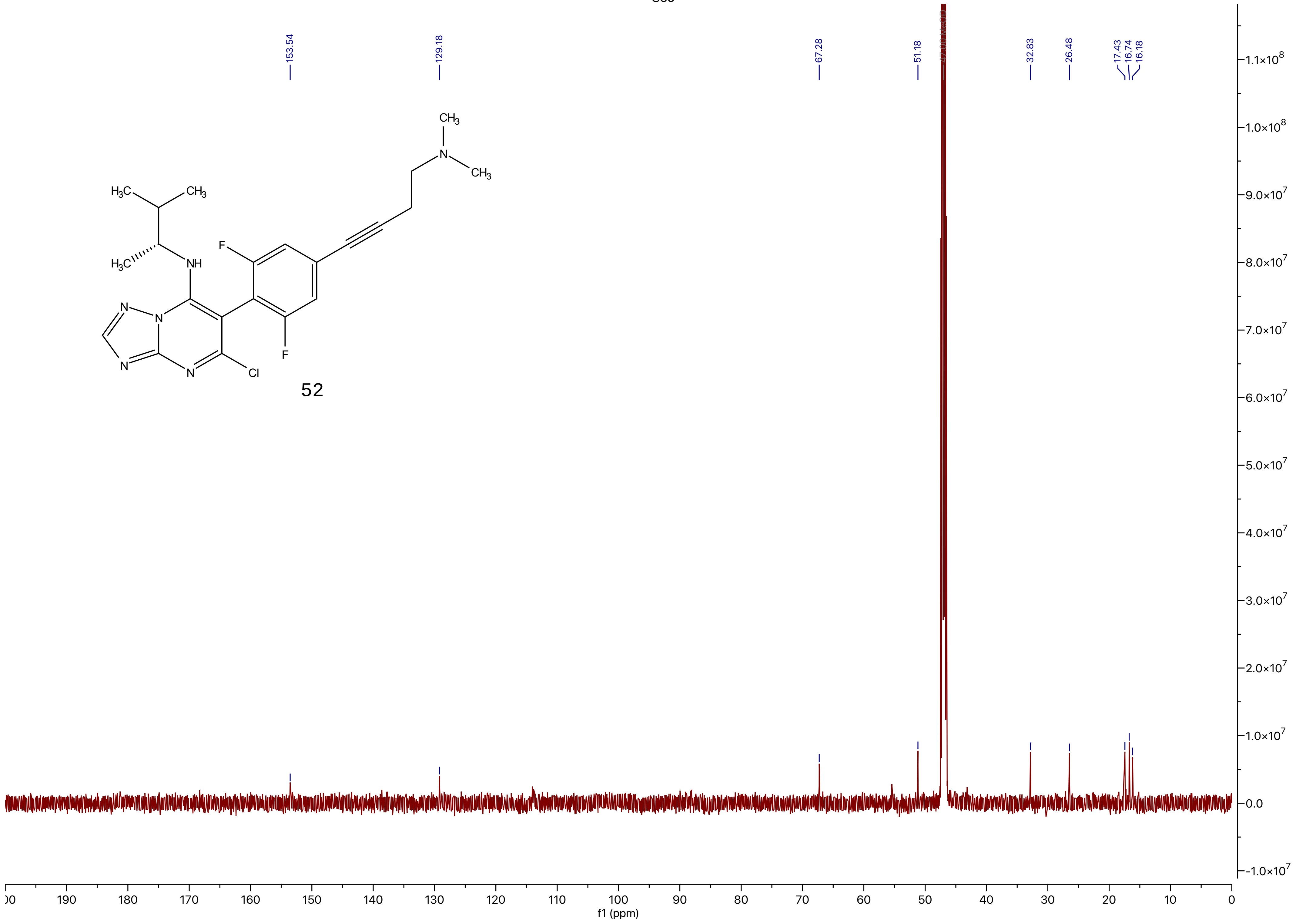
153.54
129.18
67.28
51.18
32.83
26.48
17.43
16.74
16.18
CH3
N
CH3
H3C
CH3
F
H3C
NH
N
N
N
N
F
Cl
52
1.1×10^8
1.0×10^8
9.0×10^7
8.0×10^7
7.0×10^7
6.0×10^7
5.0×10^7
4.0×10^7
3.0×10^7
2.0×10^7
1.0×10^7
0.0
-1.0×10^7
190
180
170
160
150
140
130
120
110
100
90
80
70
60
50
40
30
20
10
0
f1 (ppm)

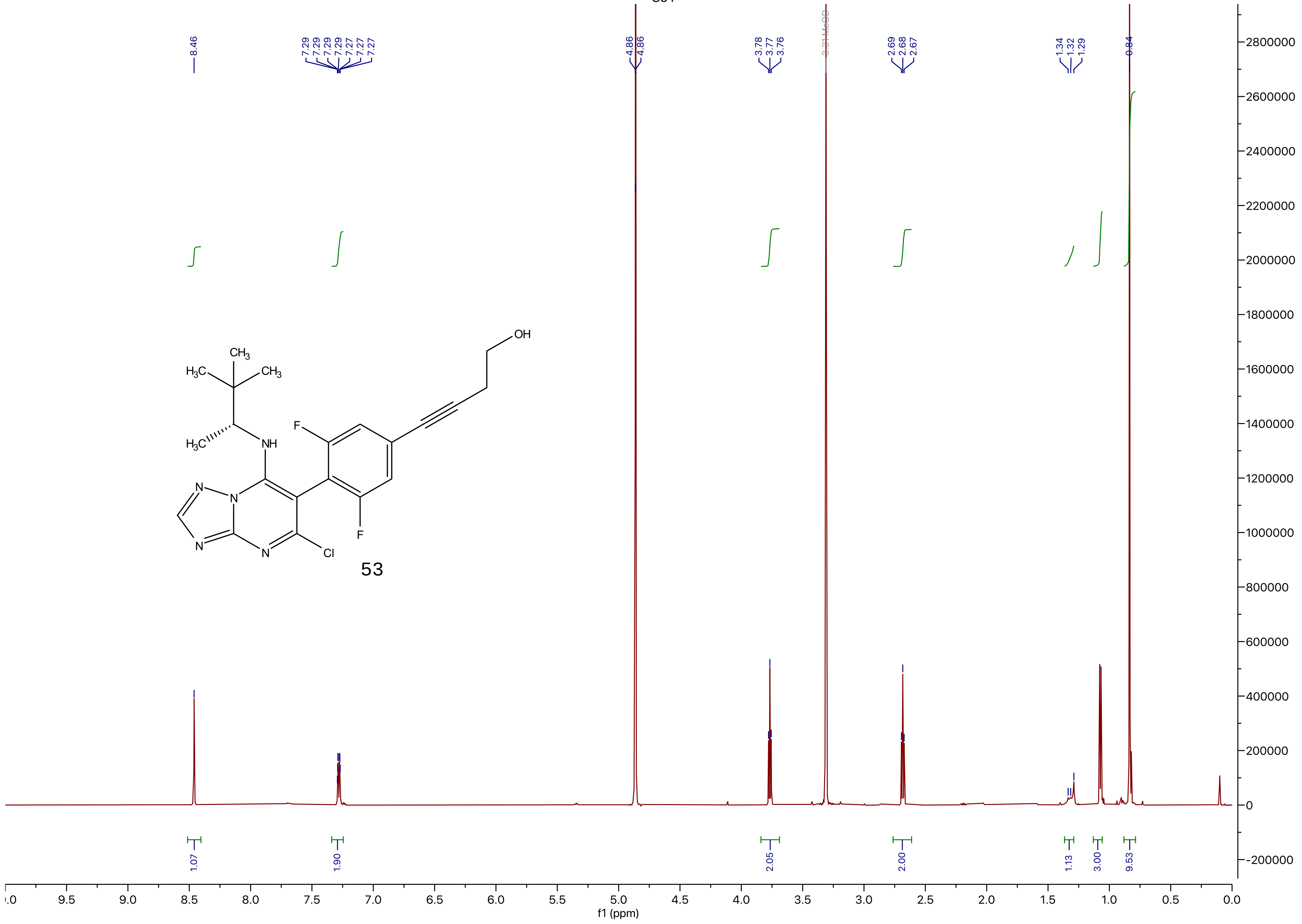

8.46
7.29
7.29
7.29
7.29
7.27
7.27
7.27
4.86
4.86
3.78
3.77
3.76
3.31 MeOD
2.69
2.68
2.67
1.34
1.32
1.29
0.84
1.07
1.90
2.05
2.00
1.13
3.00
9.53
OH
CH3
H3C
CH3
F
H3C
NH
N
N
N
N
F
Cl
53
2800000
2600000
2400000
2200000
2000000
1800000
1600000
1400000
1200000
1000000
800000
600000
400000
200000
0
-200000
9.5
9.0
8.5
8.0
7.5
7.0
6.5
6.0
5.5
5.0
4.5
4.0
3.5
3.0
2.5
2.0
1.5
1.0
0.5
0.0
f1 (ppm)

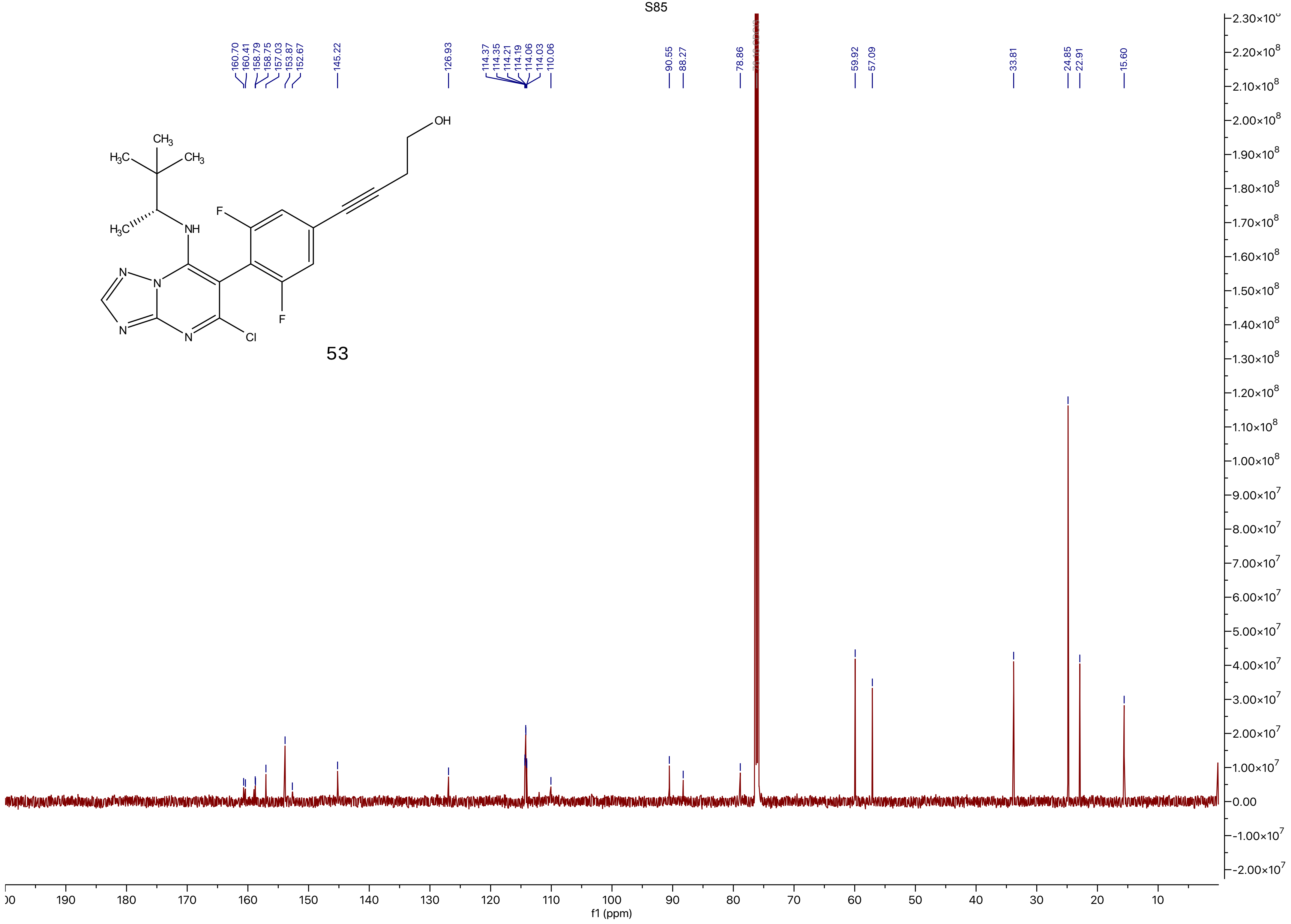
160.70
160.41
158.79
158.75
157.03
153.87
152.67
145.22
126.93
114.37
114.35
114.21
114.19
114.06
114.03
110.06
90.55
88.27
78.86
59.92
57.09
33.81
24.85
22.91
15.60
OH
CH3
H3C
CH3
F
H3C
NH
N
N
N
F
N
N
Cl
f1 (ppm)

53

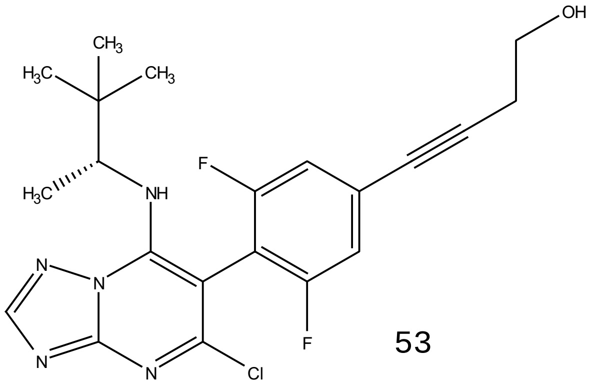

53

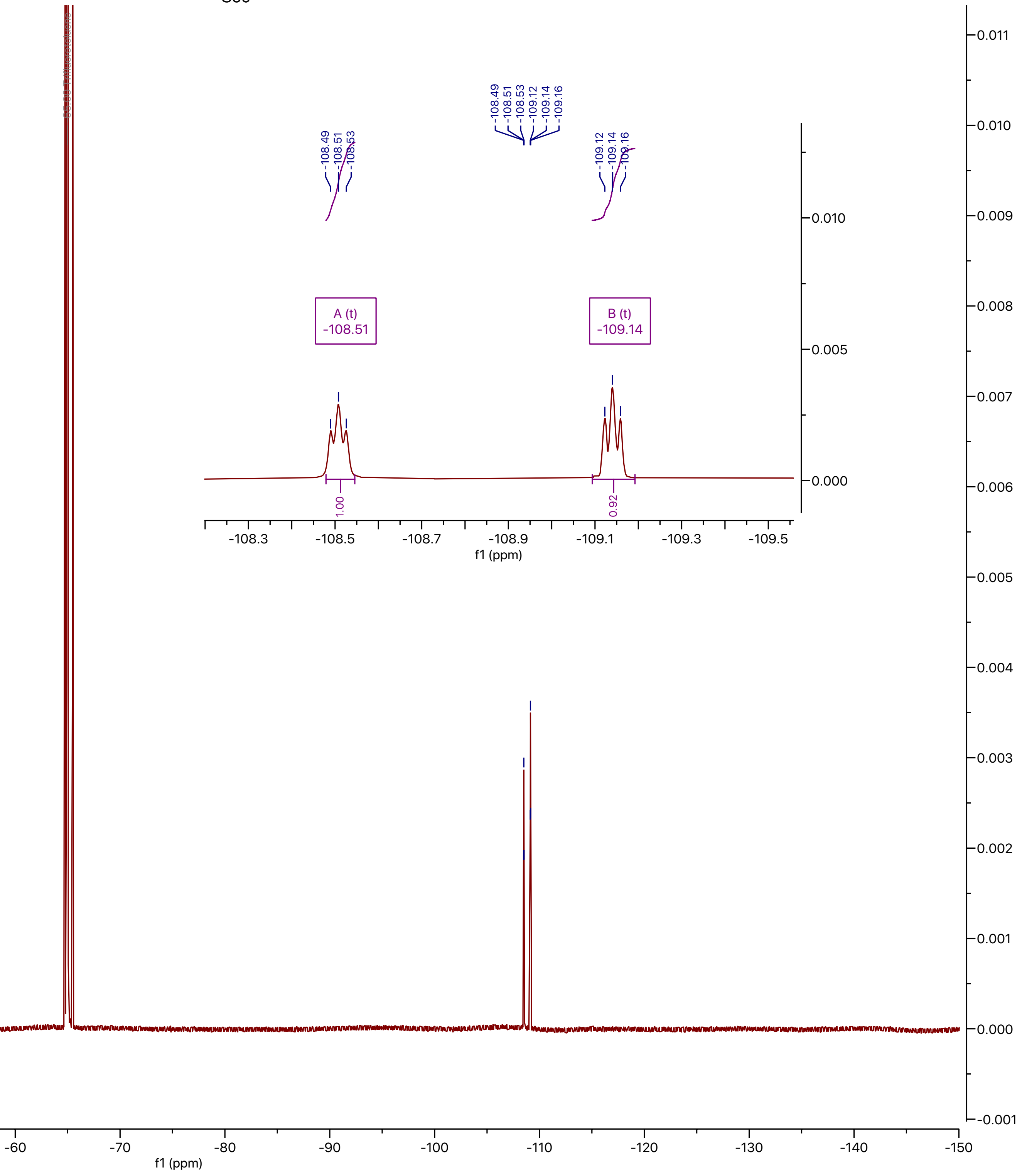
-108.49
-108.51
-108.53
-109.12
-109.14
-109.16
A (t)
-108.51
B (t)
-109.14
1.00
0.92
-108.3
-108.5
-108.7
-108.9
-109.1
-109.3
-109.5
f1 (ppm)
0.010
0.005
0.000
0.011
0.010
0.009
0.008
0.007
0.006
0.005
0.004
0.003
0.002
0.001
0.000
-0.001
-10
-20
-30
-40
-50
-60
-70
-80
-90
-100
-110
-120
-130
-140
-150
f1 (ppm)

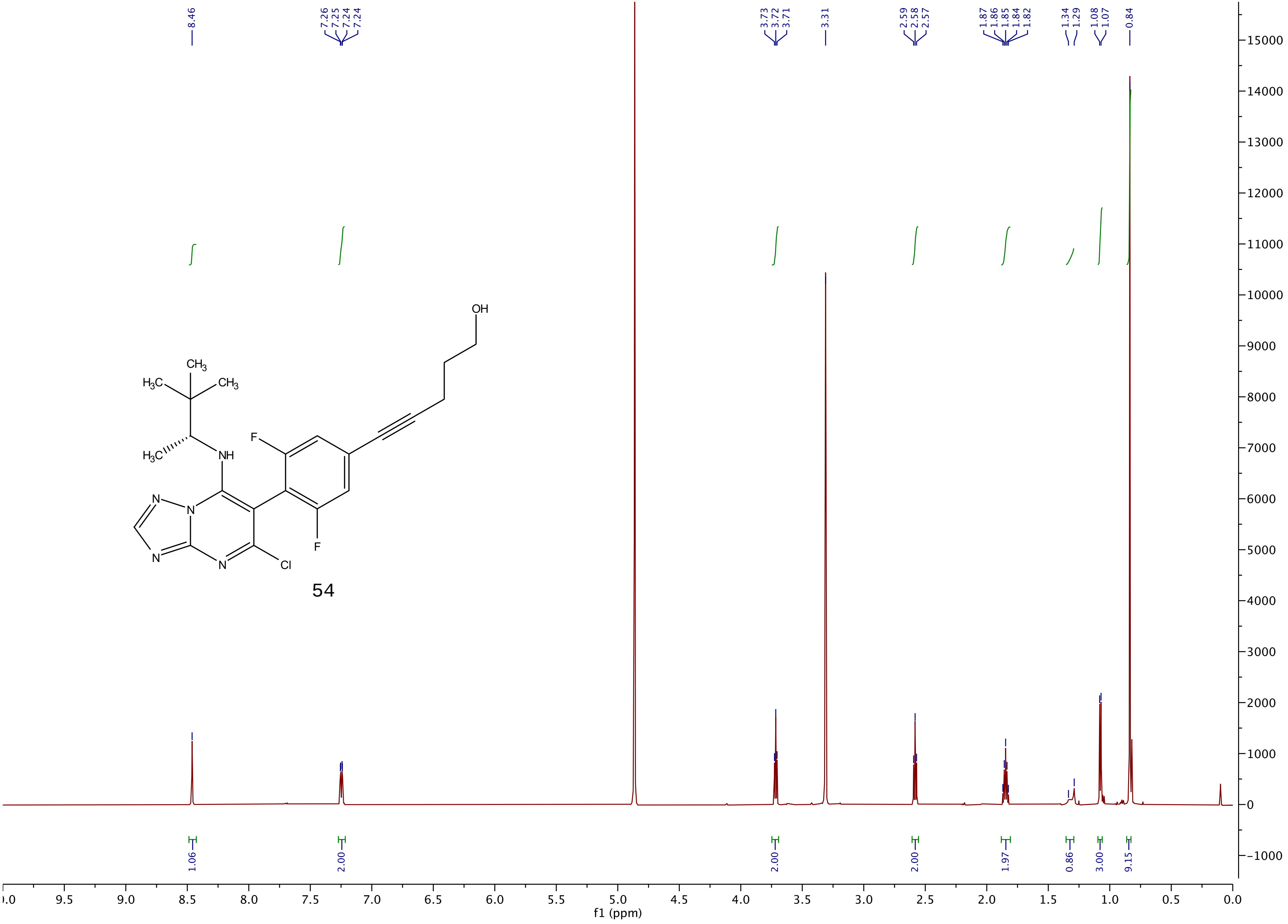
8.46
7.26
7.25
7.24
7.24
3.73
3.72
3.71
3.31
2.59
2.58
2.57
1.87
1.86
1.85
1.84
1.82
1.34
1.29
1.08
1.07
0.84
OH
CH3
H3C
CH3
F
H3C
NH
N
N
N
N
Cl
F
1.06
2.00
2.00
2.00
1.97
0.86
3.00
9.15
f1 (ppm)

54

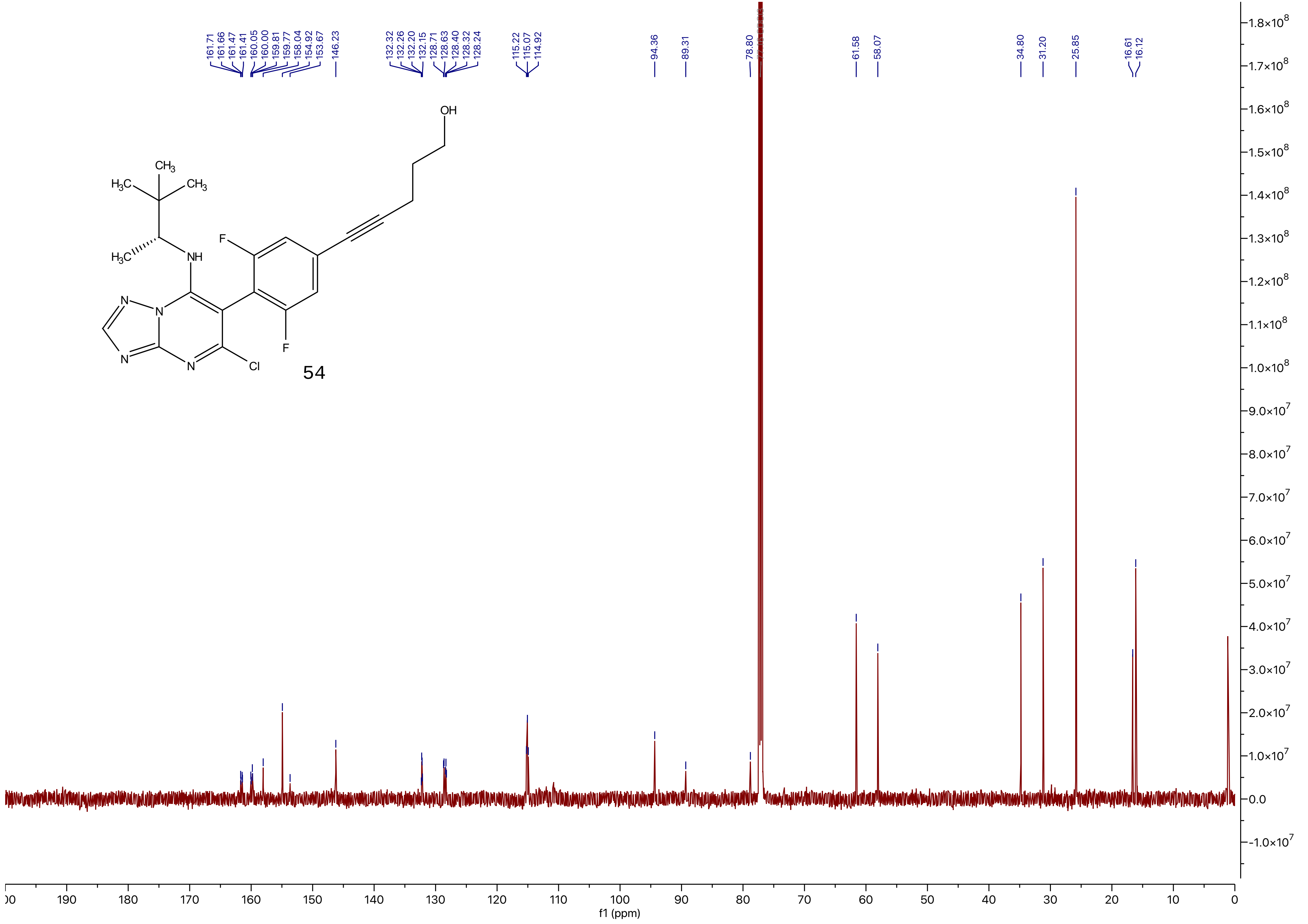
161.71
161.66
161.47
161.41
160.05
160.00
159.81
159.77
158.04
154.92
153.67
146.23
132.32
132.26
132.20
132.15
128.71
128.63
128.40
128.32
128.24
115.22
115.07
114.92
94.36
89.31
78.80
61.58
58.07
34.80
31.20
25.85
16.61
16.12
OH
CH_3
H_3C
CH_3
F
H_3C
NH
N
N
N
N
F
Cl
54
f1 (ppm)

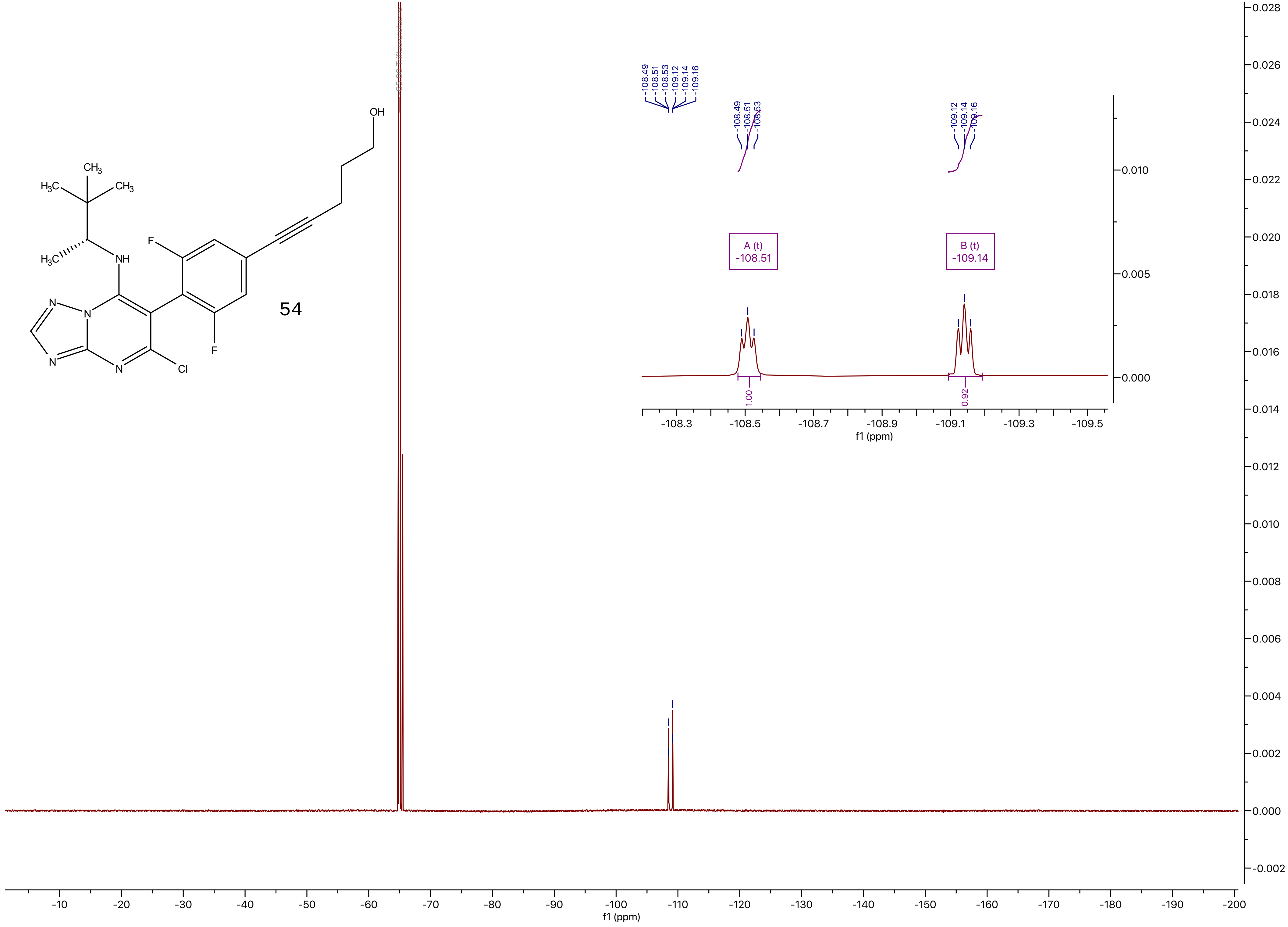

OH
CH3
H3C
CH3
F
H3C
NH
N
N
N
F
N
N
Cl
54
-108.49
-108.51
-108.53
-109.12
-109.14
-109.16
A (t)
-108.51
B (t)
-109.14
1.00
0.92
-108.3
-108.5
-108.7
-108.9
-109.1
-109.3
-109.5
f1 (ppm)
0.010
0.005
0.000
-10
-20
-30
-40
-50
-60
-70
-80
-90
-100
-110
-120
-130
-140
-150
-160
-170
-180
-190
-200
f1 (ppm)
0.028
0.026
0.024
0.022
0.020
0.018
0.016
0.014
0.012
0.010
0.008
0.006
0.004
0.002
0.000
-0.002

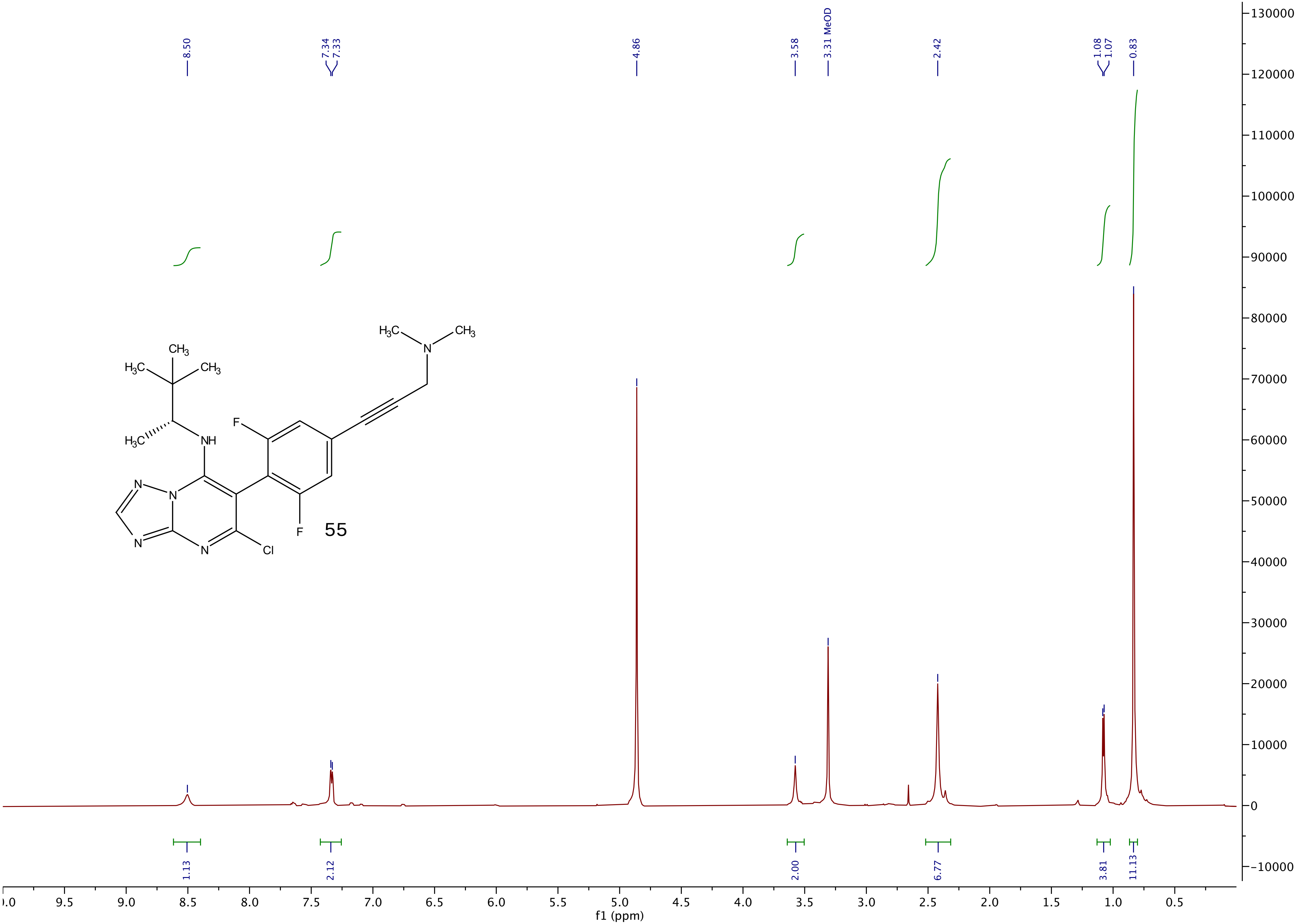
8.50
7.34
7.33
4.86
3.58
3.31 MeOD
2.42
1.08
1.07
0.83
1.13
2.12
2.00
6.77
3.81
11.13
55
f1 (ppm)

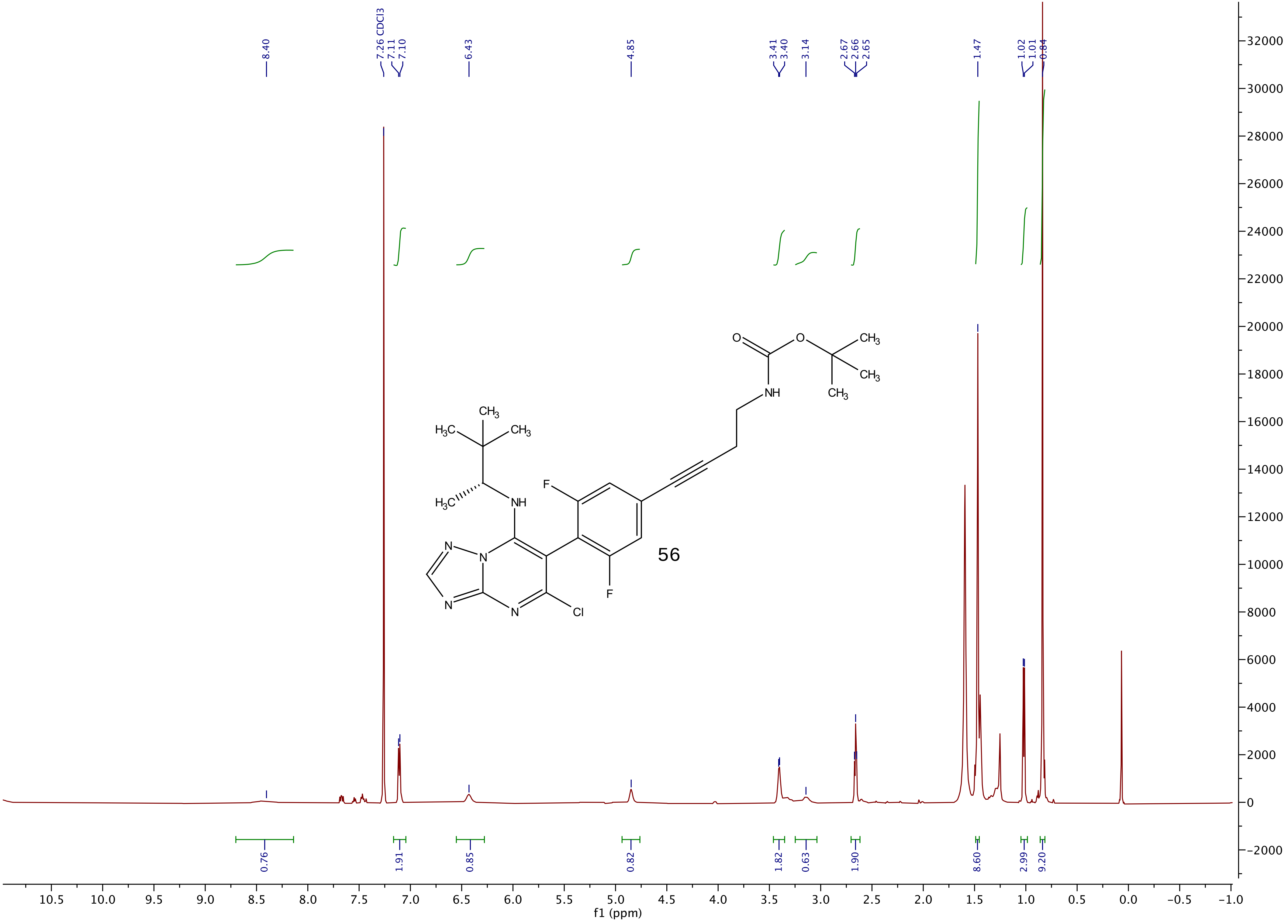
7.26 CDCl3
8.40
7.11
7.10
6.43
4.85
3.41
3.40
3.14
2.67
2.66
2.65
1.47
1.02
1.01
0.84
0.76
1.91
0.85
0.82
1.82
0.63
1.90
8.60
2.99
9.20
56
f1 (ppm)

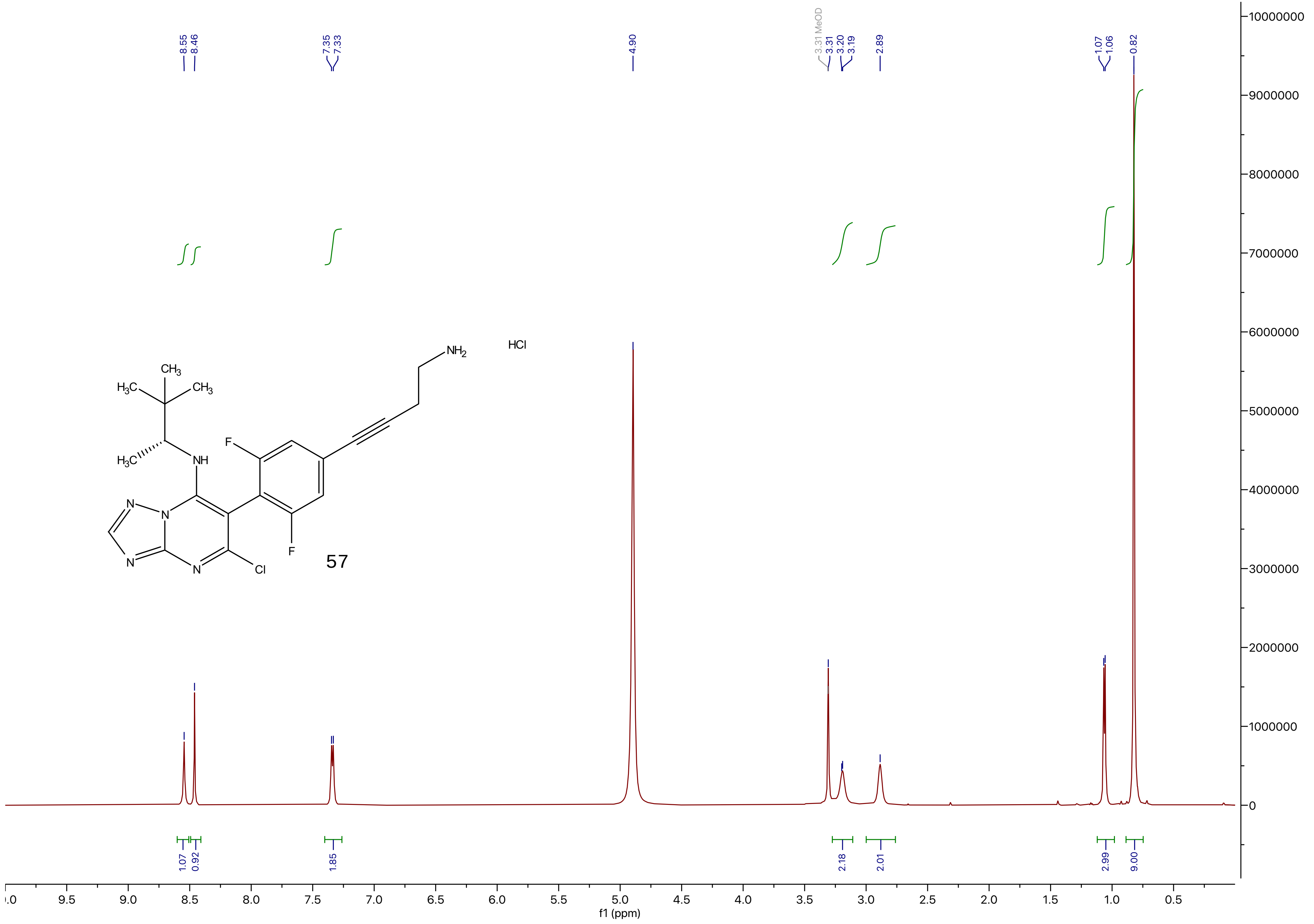

8.55
8.46
7.35
7.33
4.90
3.31 MeOD
3.31
3.20
3.19
2.89
1.07
1.06
0.82
NH2
HCl
CH3
H3C
CH3
H3C
NH
F
F
N
N
N
N
Cl
57
1.07
0.92
1.85
2.18
2.01
2.99
9.00
f1 (ppm)

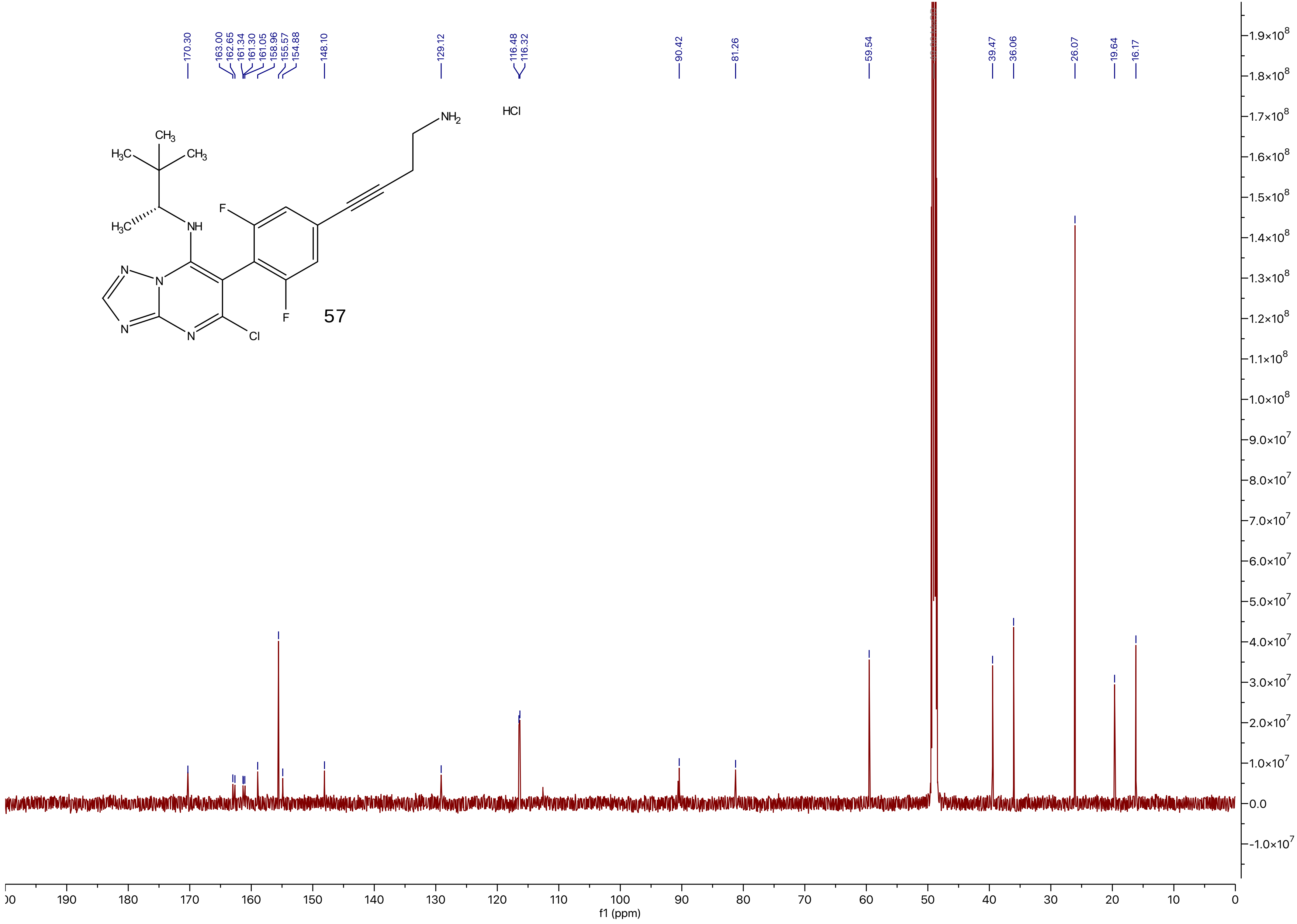
170.30
163.00
162.65
161.34
161.30
161.05
158.96
155.57
154.88
148.10
129.12
116.48
116.32
90.42
81.26
59.54
39.47
36.06
26.07
19.64
16.17
HCl
f1 (ppm)

57

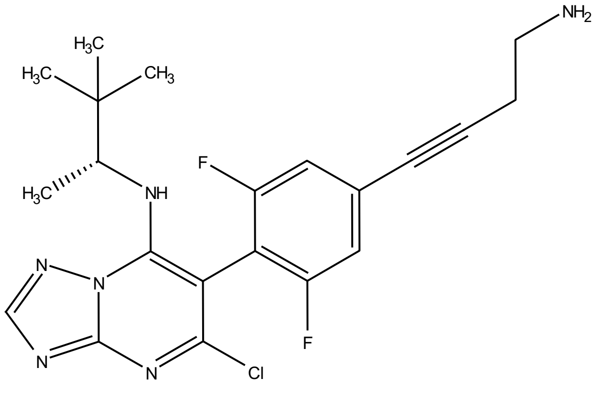

57

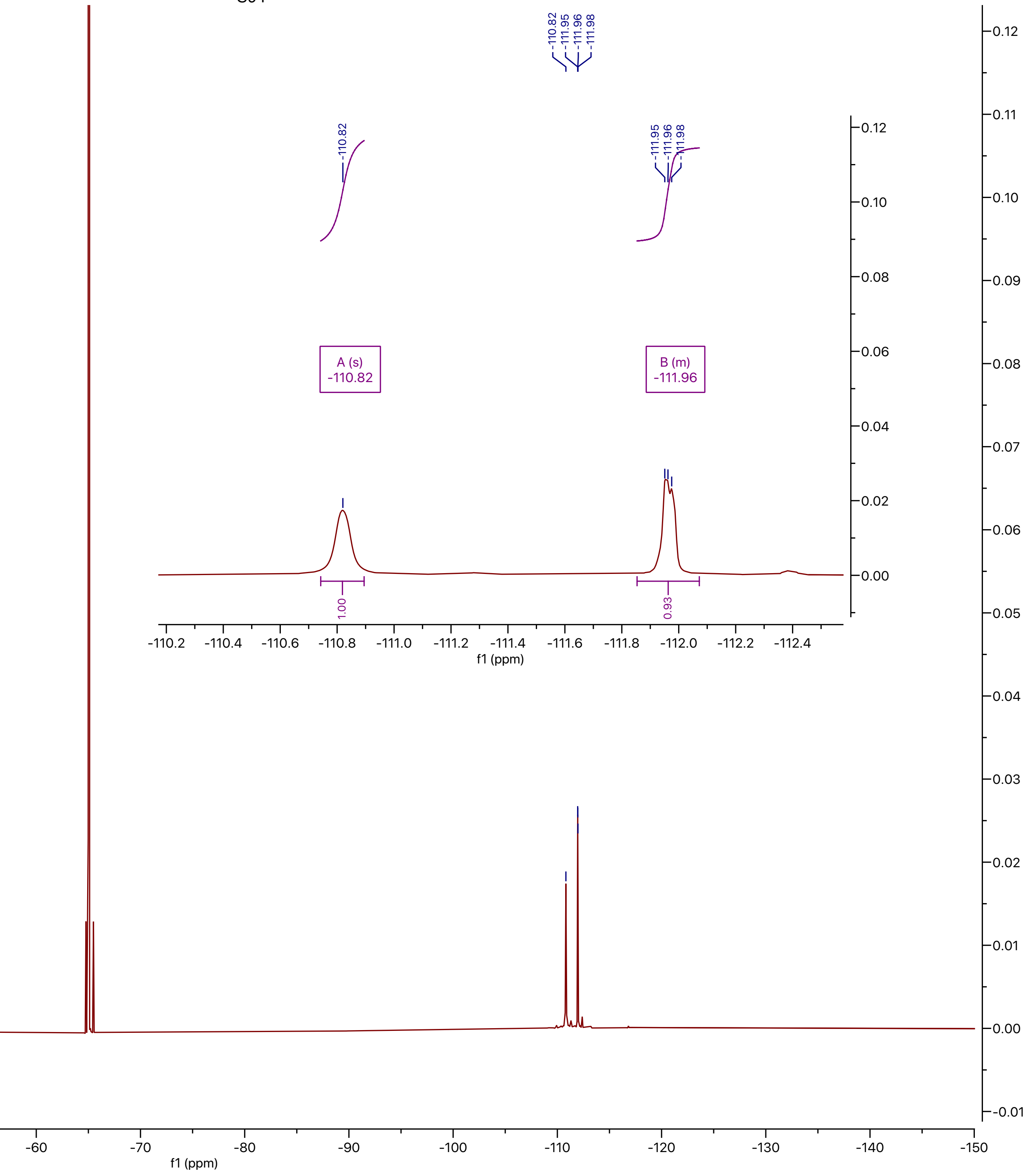
-110.82
-111.95
-111.96
-111.98
A (s)
-110.82
B (m)
-111.96
1.00
0.93
f1 (ppm)
f1 (ppm)

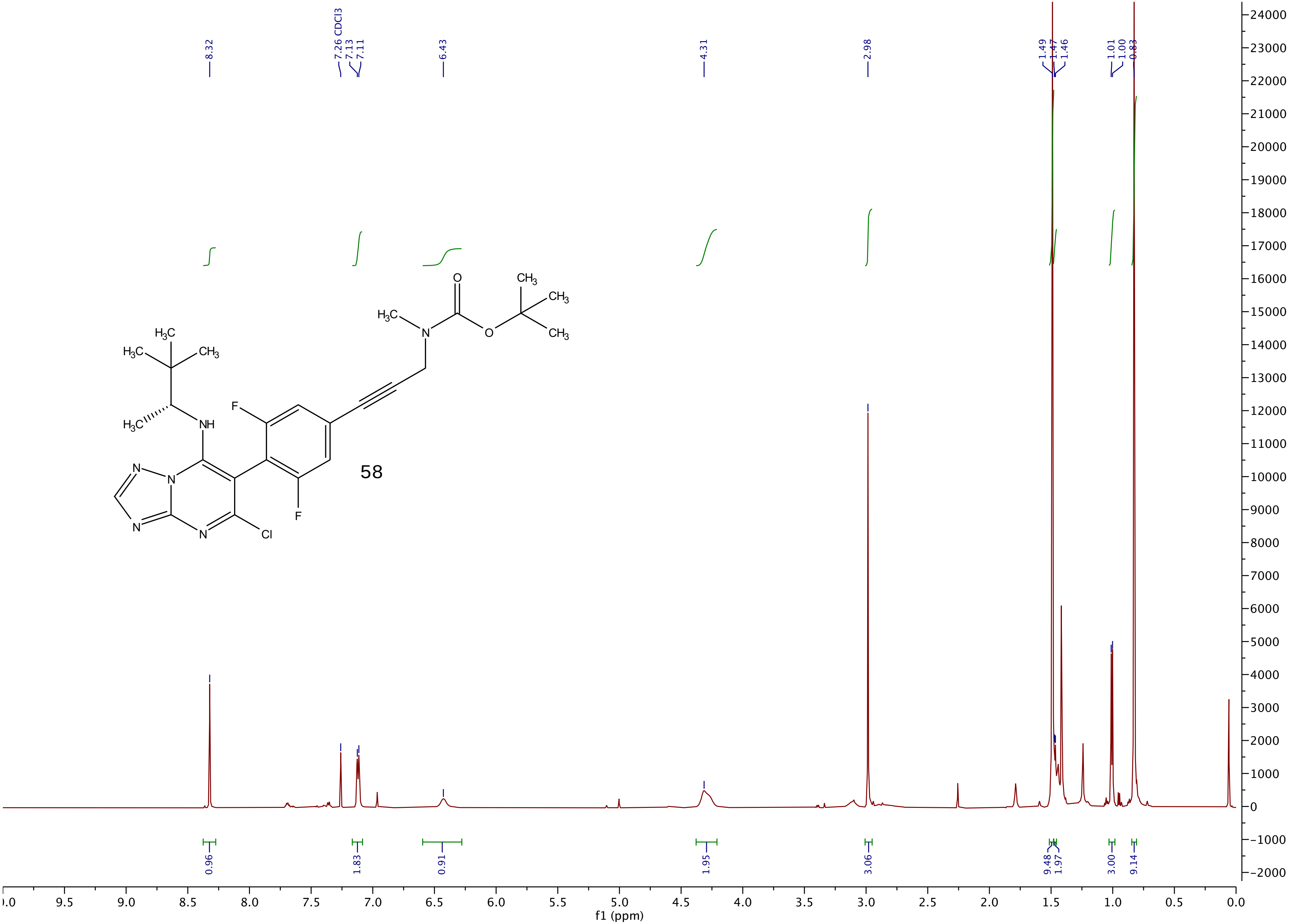
7.26 CDCl3
7.13
7.11
8.32
6.43
4.31
2.98
1.49
1.47
1.46
1.01
1.00
0.83
58
H3C
CH3
NH
F
N
Cl
O
0.96
1.83
0.91
1.95
3.06
9.48
1.97
3.00
9.14
f1 (ppm)

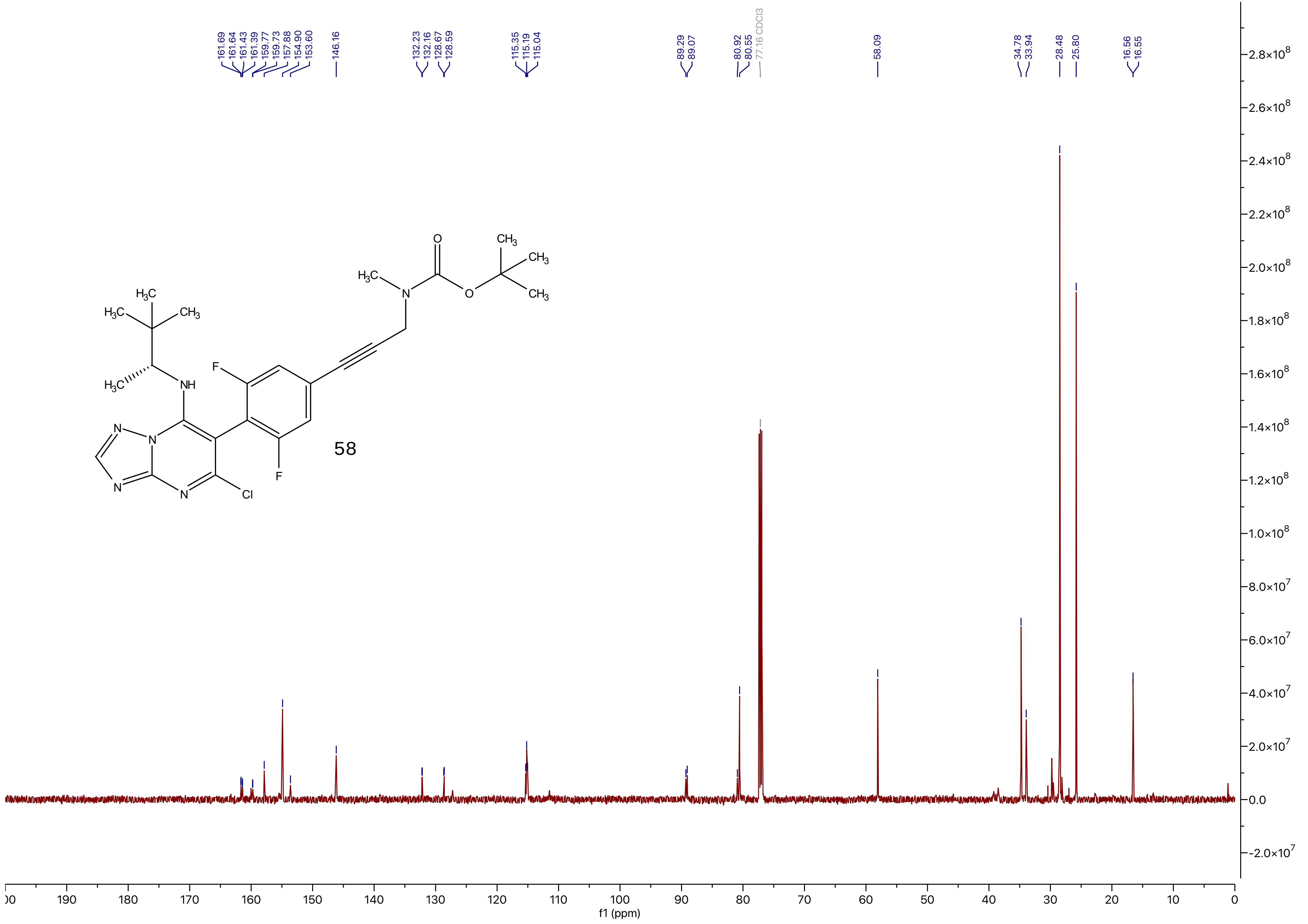
161.69
161.64
161.43
161.39
159.77
159.73
157.88
154.90
153.60
146.16
132.23
132.16
128.67
128.59
115.35
115.19
115.04
89.29
89.07
80.92
80.55
77.16 CDCl3
58.09
34.78
33.94
28.48
25.80
16.56
16.55
58
f1 (ppm)

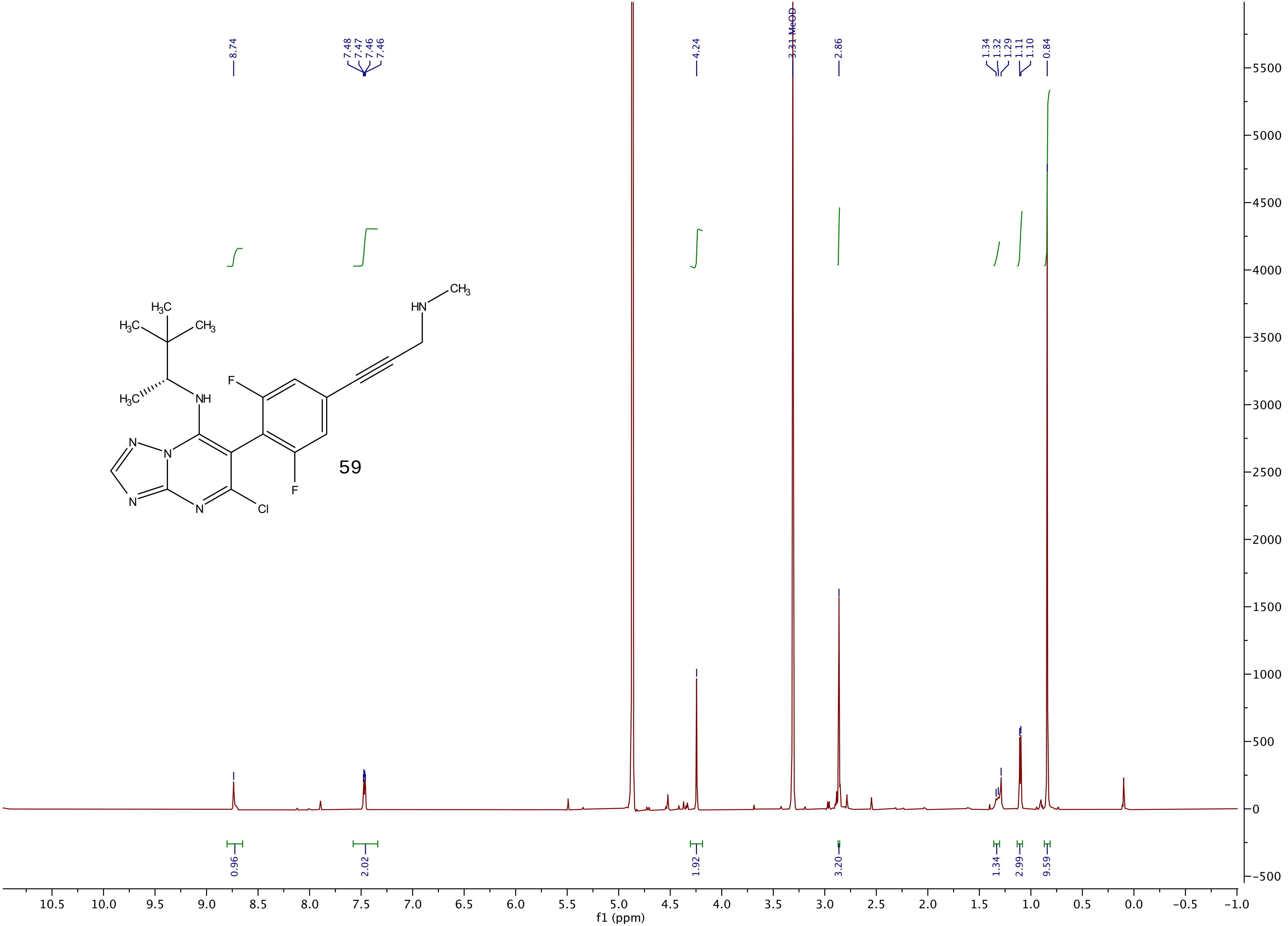
8.74
7.48
7.47
7.46
7.46
4.24
3.31 MeOD
2.86
1.34
1.32
1.29
1.11
1.10
0.84
59
0.96
2.02
1.92
3.20
1.34
2.99
9.59
f1 (ppm)

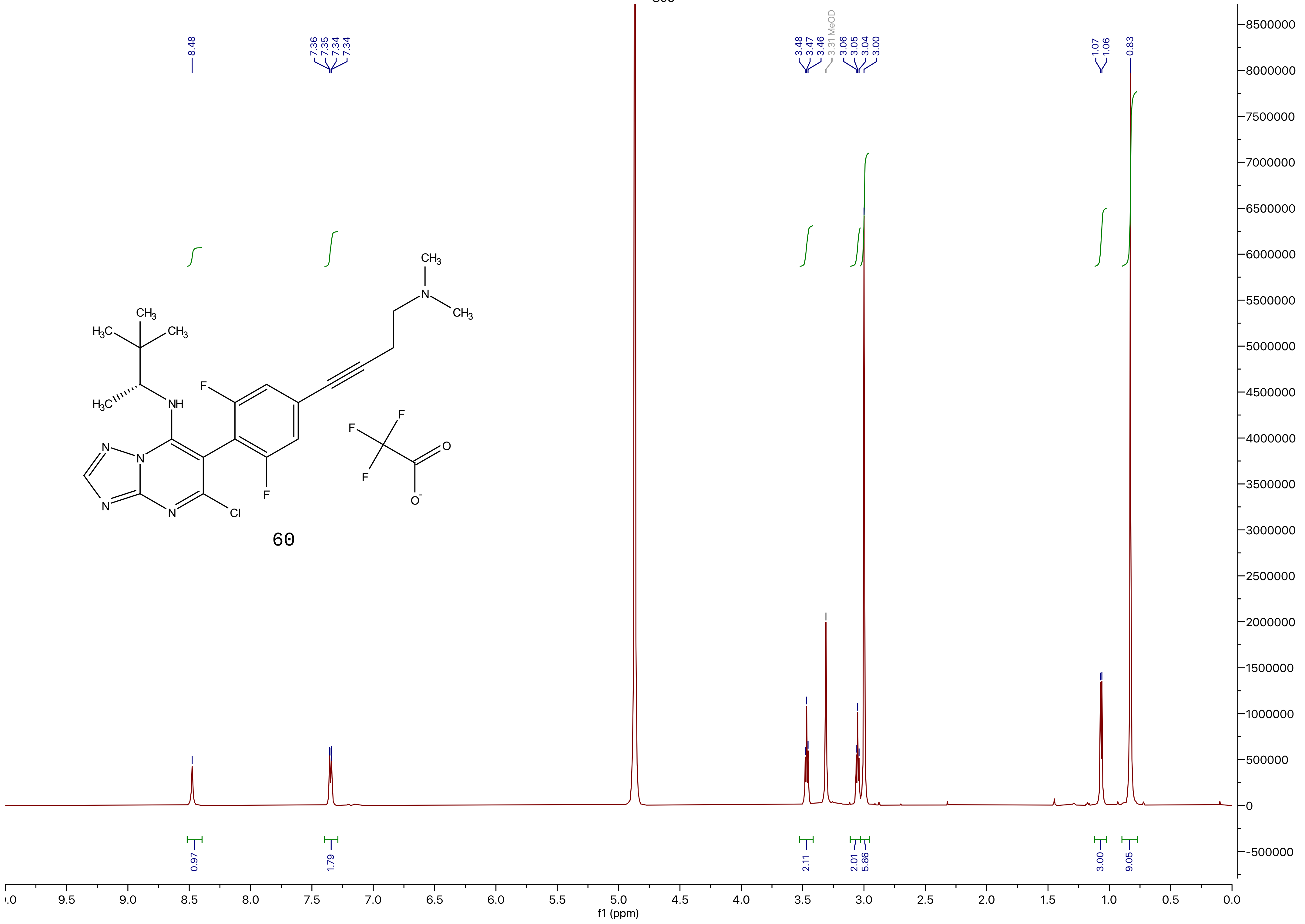
8.48
7.36
7.35
7.34
7.34
3.48
3.47
3.46
3.31 MeOD
3.06
3.05
3.04
3.00
1.07
1.06
0.83
CH3
N
CH3
CH3
H3C
CH3
F
H3C
NH
F
F
O
N
N
F
F
N
N
Cl
O-
60
0.97
1.79
2.11
2.01
5.86
3.00
9.05
f1 (ppm)

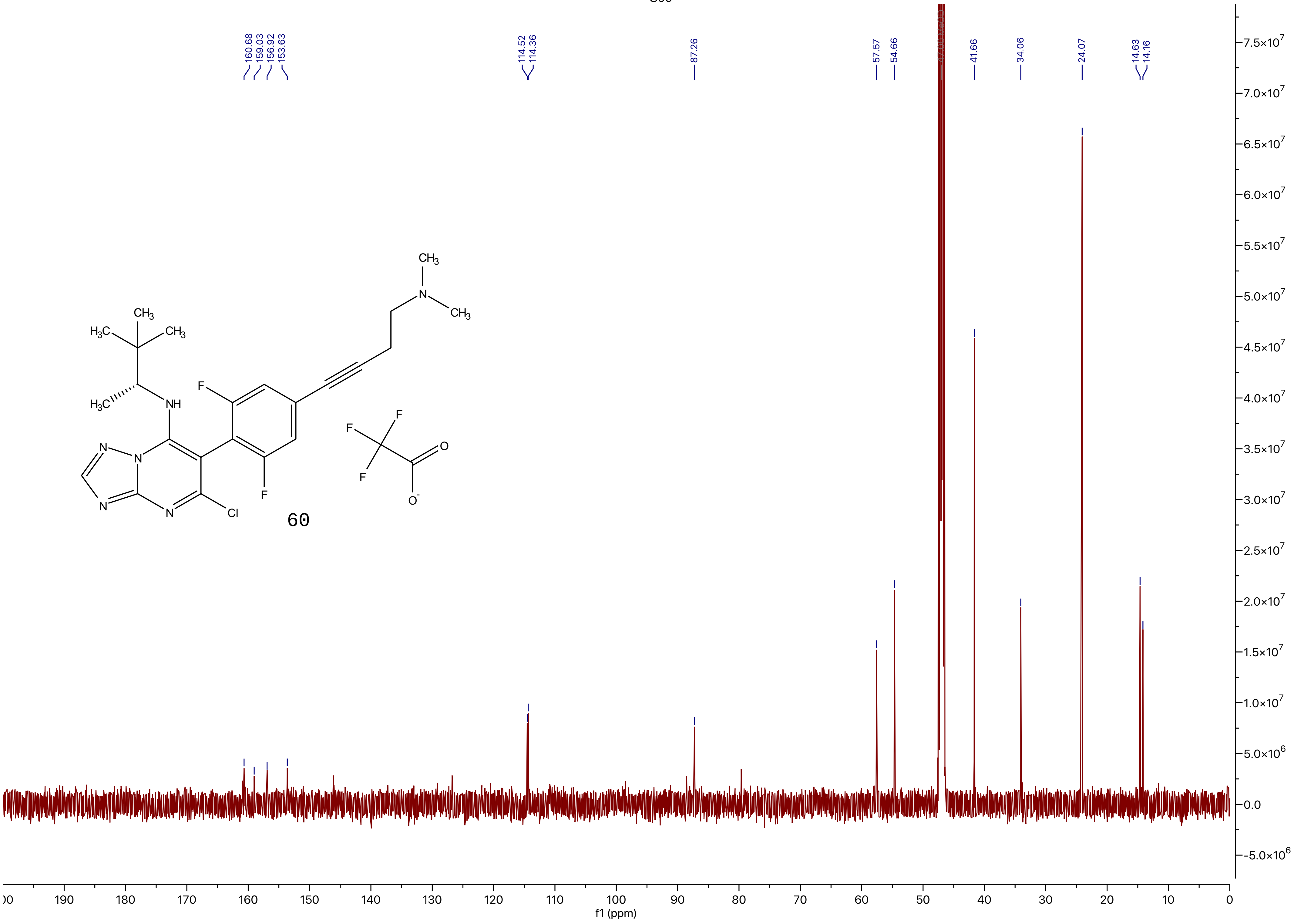
160.68
159.03
156.92
153.63
114.52
114.36
87.26
57.57
54.66
41.66
34.06
24.07
14.63
14.16
CH3
N
CH3
CH3
H3C
CH3
F
H3C
NH
F
F
O
F
N
N
N
N
Cl
F
O-
60
7.5×10^7
7.0×10^7
6.5×10^7
6.0×10^7
5.5×10^7
5.0×10^7
4.5×10^7
4.0×10^7
3.5×10^7
3.0×10^7
2.5×10^7
2.0×10^7
1.5×10^7
1.0×10^7
5.0×10^6
0.0
-5.0×10^6
190
180
170
160
150
140
130
120
110
100
90
80
70
60
50
40
30
20
10
0
f1 (ppm)

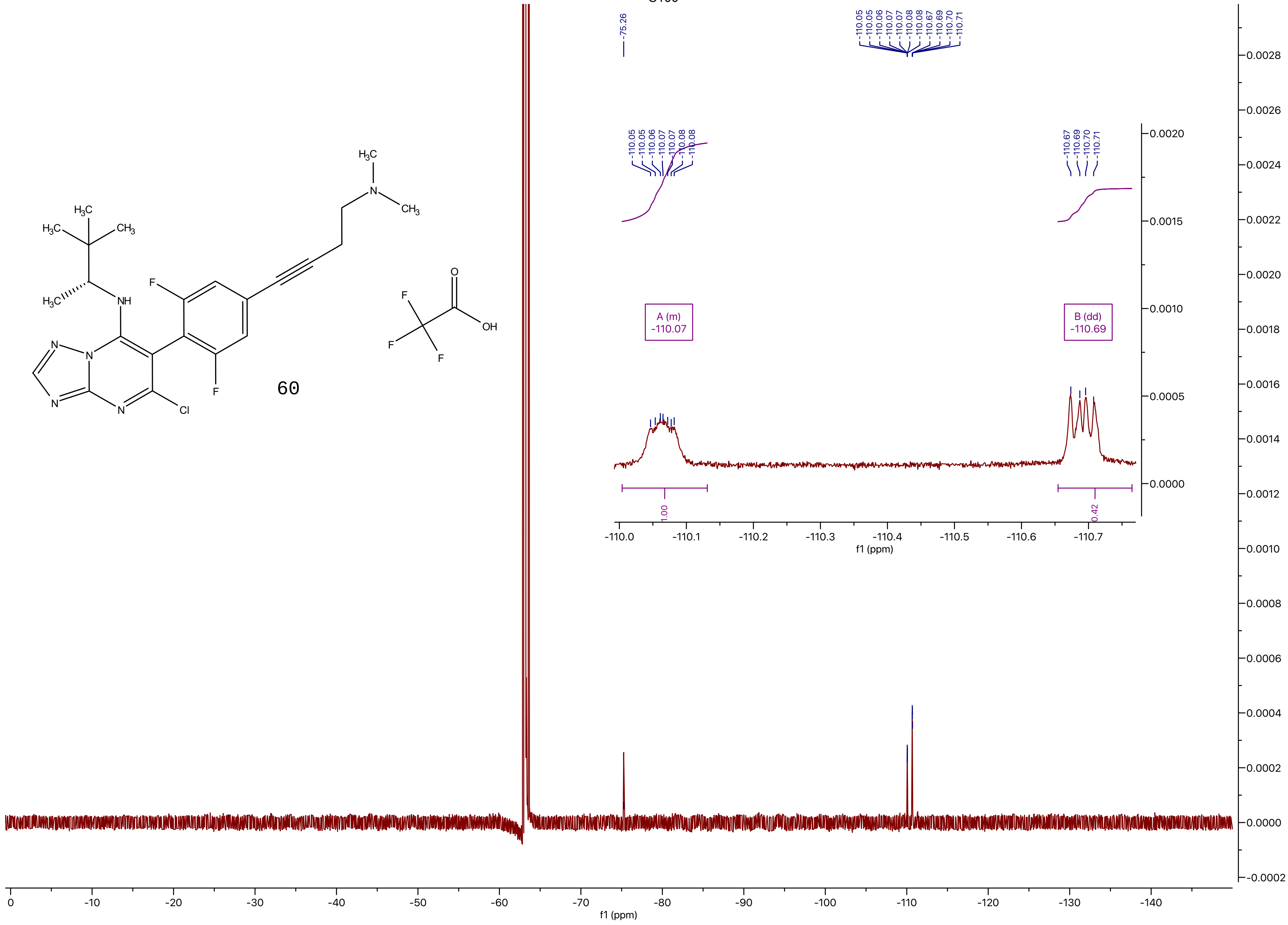

60
A (m)
-110.07
B (dd)
-110.69
1.00
0.42
f1 (ppm)

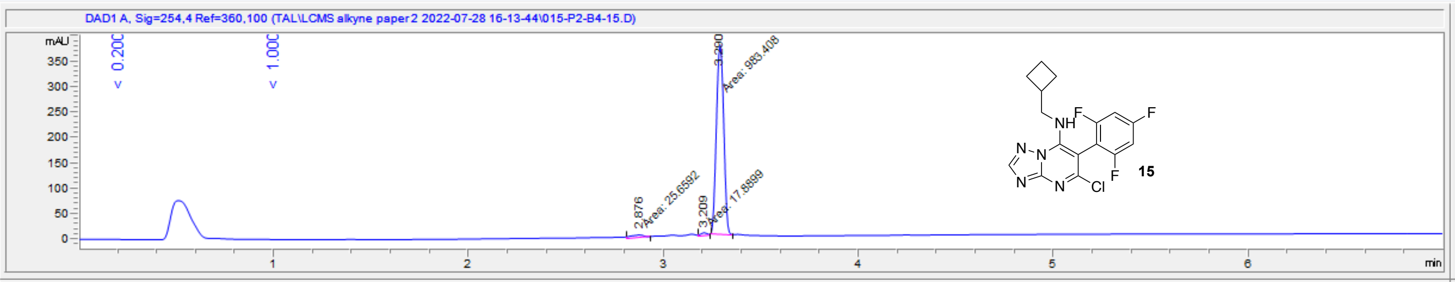

#	Time	Type	Area	Height	Width	Area%	Symmetry
1	2.876	MM	25.7	5.4	0.0788	2.499	2.209
2	3.209	MM	17.9	7	0.0429	1.742	0.884
3	3.29	MM	983.4	376.7	0.0435	95.759	0.969

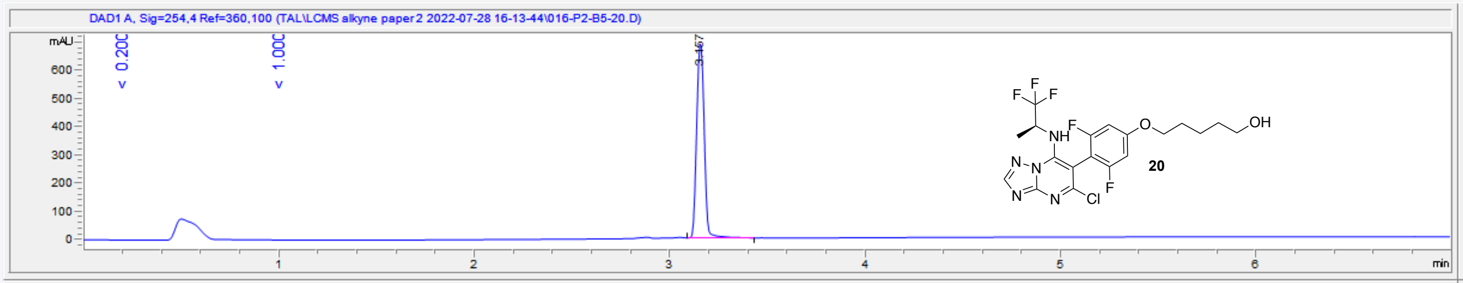

#	Time	Type	Area	Height	Width	Area%	Symmetry
1	3.157	VV	1886.3	694.7	0.0437	100.000	0.878

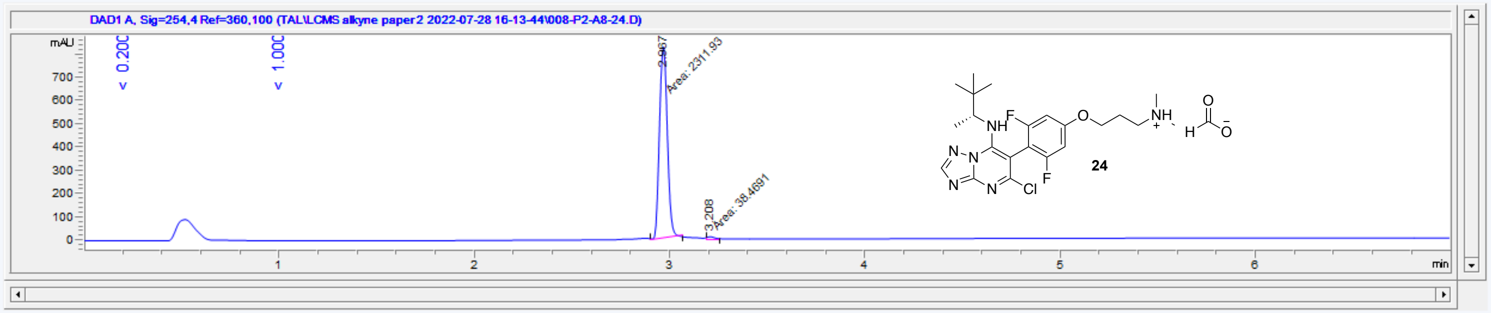

#	Time	Type	Area	Height	Width	Area%	Symmetry
1	2.967	MM	2311.9	832	0.0463	98.363	0.952
2	3.208	MM	38.5	13.2	0.0485	1.637	0.492

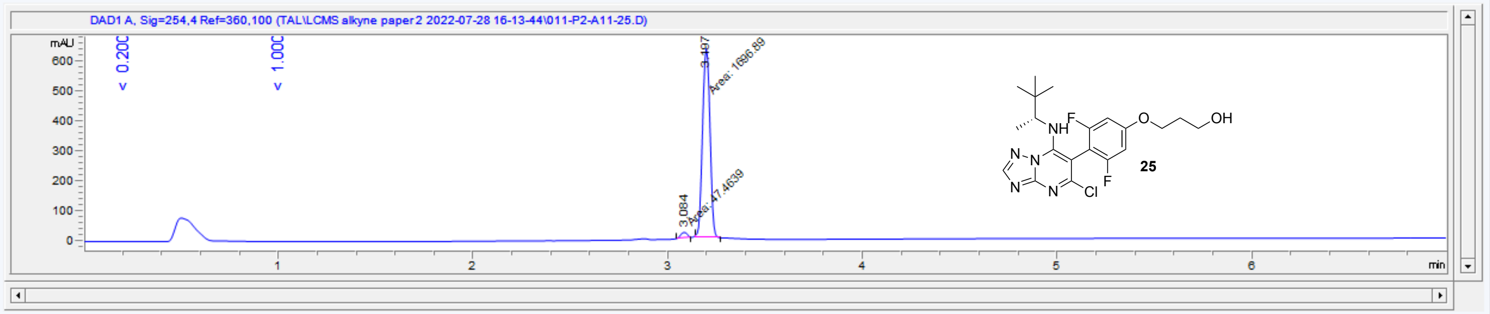

#	Time	Type	Area	Height	Width	Area%	Symmetry
1	3.084	MM	47.5	19.9	0.0397	2.721	1.101
2	3.197	MM T	1696.9	641.7	0.0441	97.279	0.997

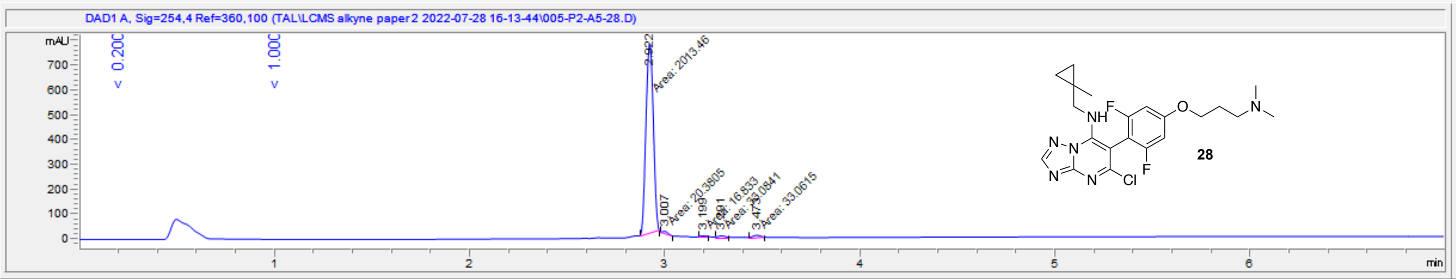

#	Time	Type	Area	Height	Width	Area%	Symmetry
1	2.922	MM	2013.5	770.1	0.0436	95.117	1.021
2	3.007	MM	20.4	10.1	0.0335	0.963	0.334
3	3.199	MM	16.8	7	0.0402	0.795	0.764
4	3.291	MM	33.1	10.4	0.0531	1.563	0.79
5	3.473	MM	33.1	11	0.0502	1.562	1.144

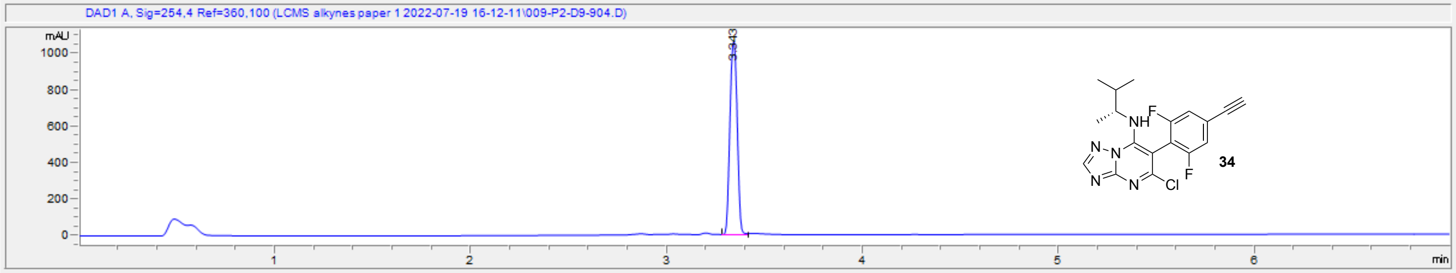

#	Time	Type	Area	Height	Width	Area%	Symmetry
1	3.343	VV	2774.7	1079.3	0.042	100.000	0.971

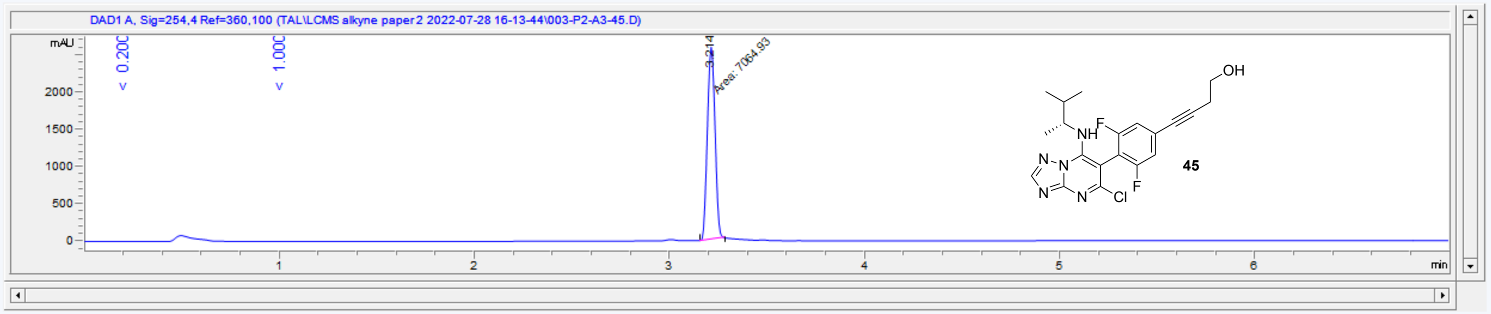

#	Time	Type	Area	Height	Width	Area%	Symmetry
1	3.214	MM	7064.9	2609.8	0.0451	100.000	0.98

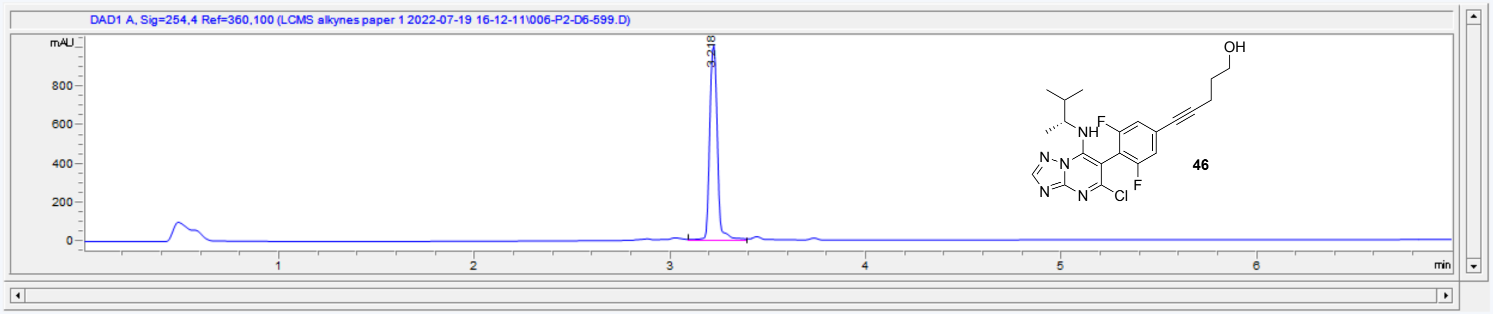

#	Time	Type	Area	Height	Width	Area%	Symmetry
1	3.218	VV	2771	1008	0.0441	100.000	0.882

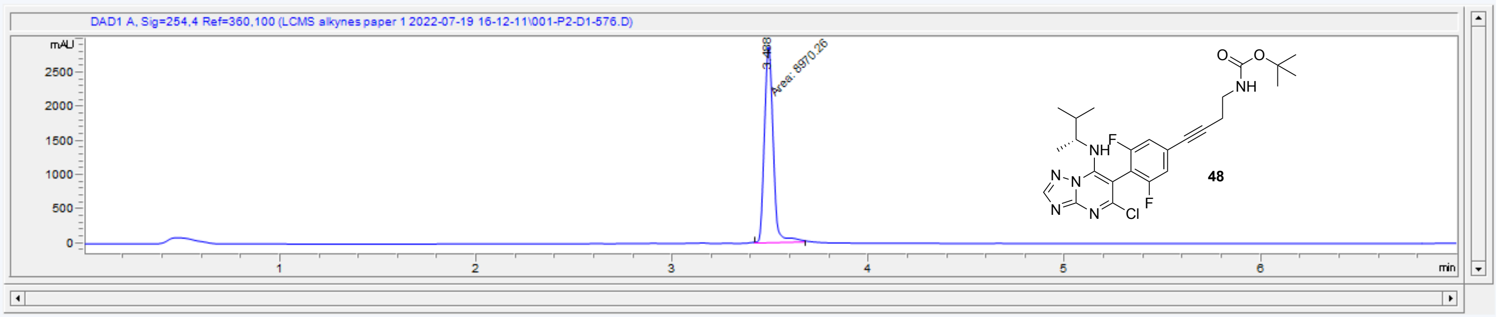

#	Time	Type	Area	Height	Width	Area%	Symmetry
1	3.488	MM	8970.3	2902.6	0.0515	100.000	0.854

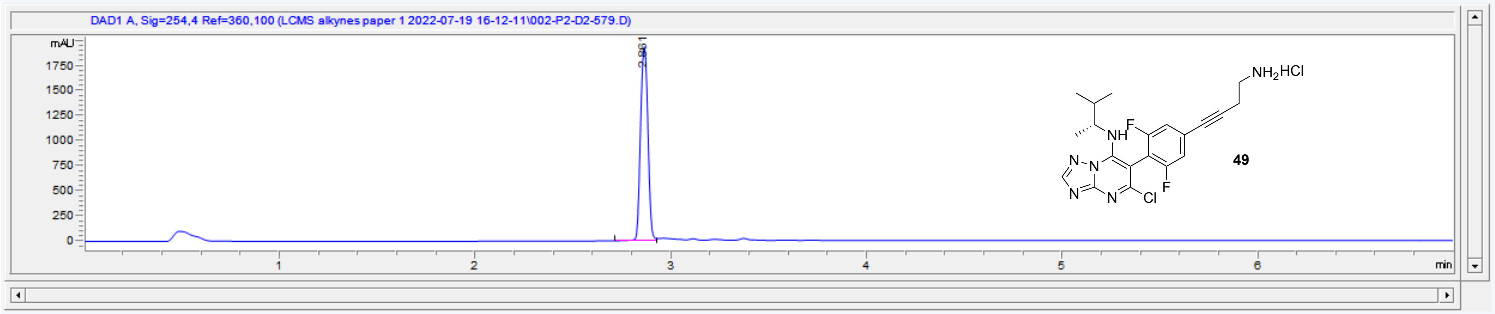

#	Time	Type	Area	Height	Width	Area%	Symmetry
1	2.861	VV	5106.4	1937.6	0.0427	100.000	1.002

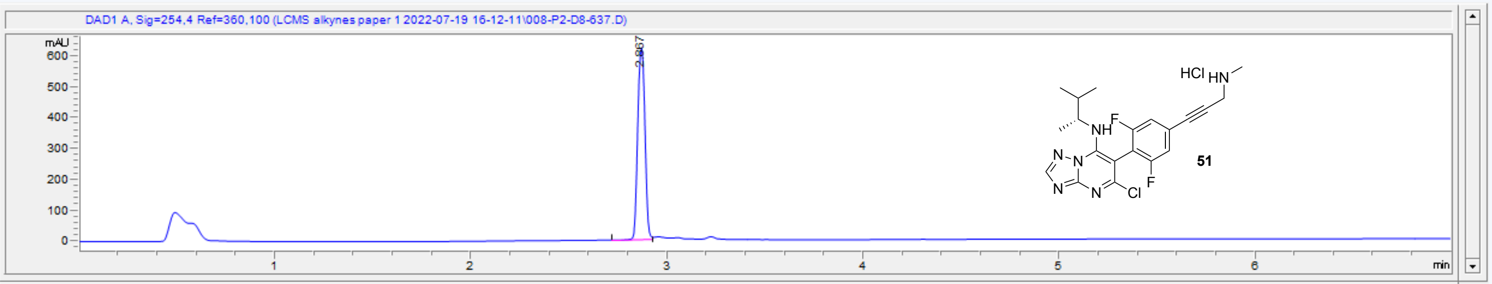

#	Time	Type	Area	Height	Width	Area%	Symmetry
1	2.867	VV	1633.7	629.4	0.0423	100.000	1.004

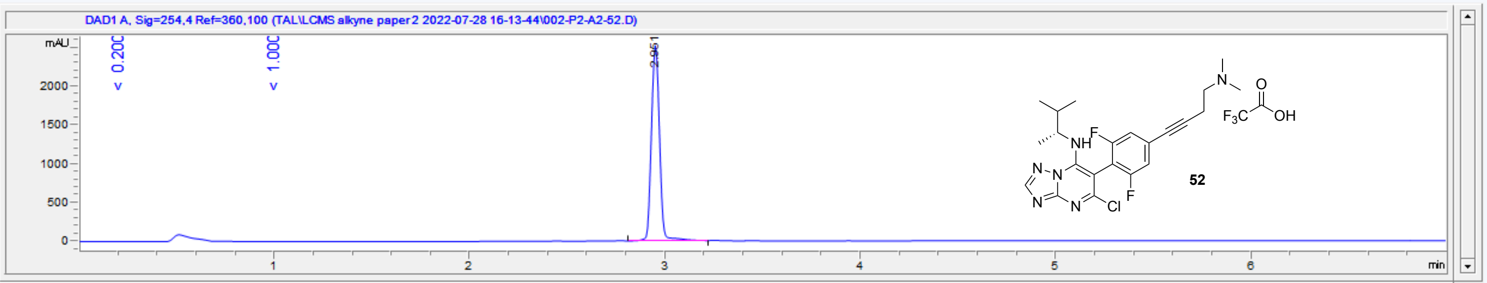

#	Time	Type	Area	Height	Width	Area%	Symmetry
1	2.951	VV	7377.5	2511.2	0.0463	100.000	0.901

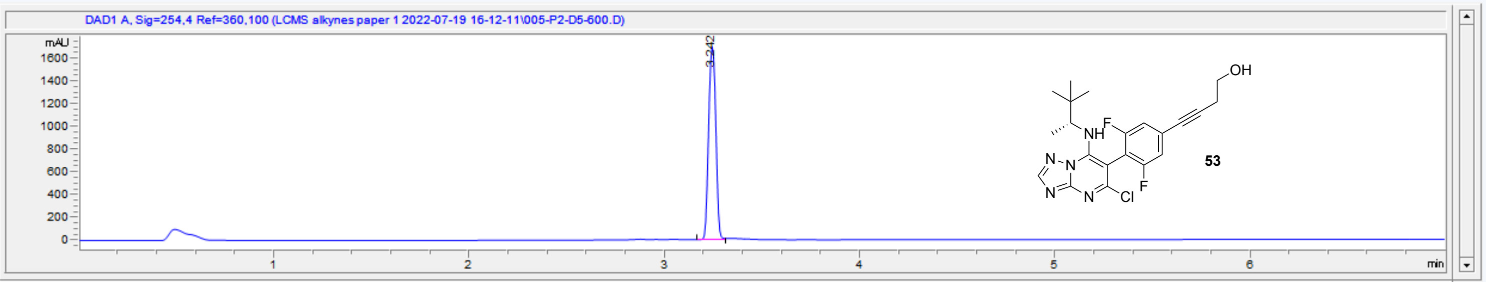

#	Time	Type	Area	Height	Width	Area%	Symmetry
1	3.242	VV	4385.6	1707.6	0.042	100.000	0.959

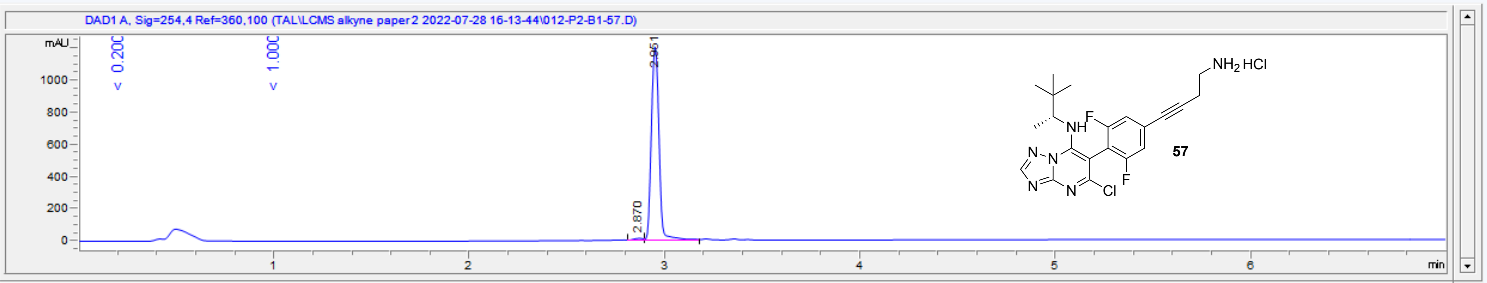

#	Time	Type	Area	Height	Width	Area%	Symmetry
1	2.87	VV	50.7	14.3	0.0591	1.501	1.608
2	2.951	VV	3329.2	1207.5	0.0442	98.499	0.877

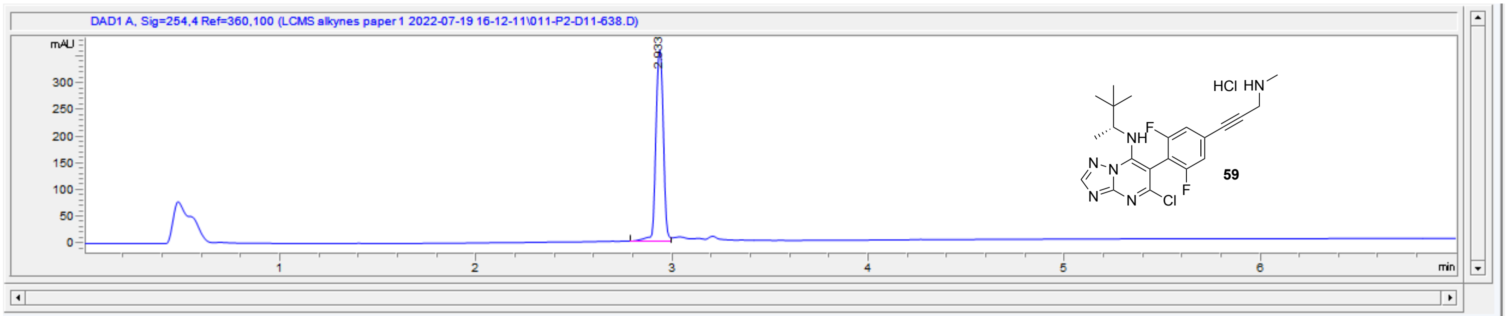

#	Time	Type	Area	Height	Width	Area%	Symmetry
1	2.933	VV	969.9	363.1	0.0432	100.000	1.059

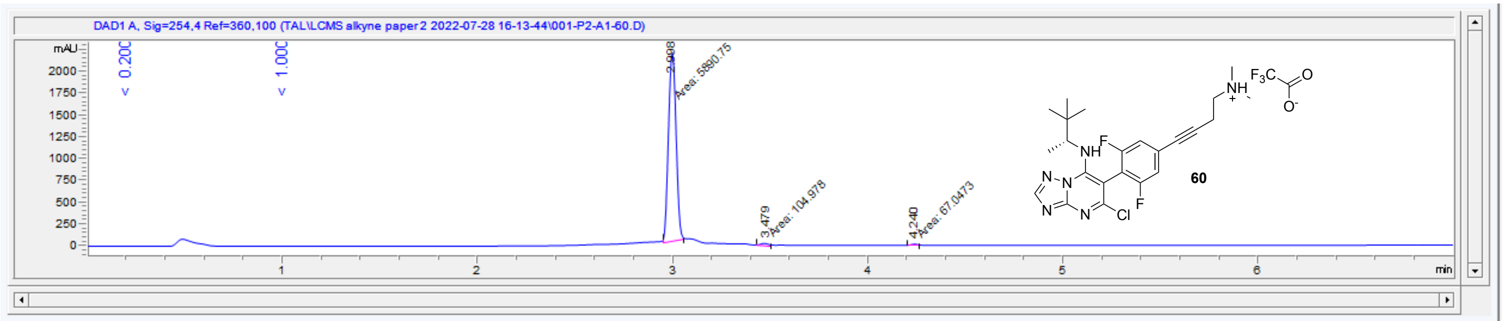

#	Time	Type	Area	Height	Width	Area%	Symmetry
1	2.998	MM	5890.8	2184	0.045	97.163	0.986
2	3.479	MM	105	33.3	0.0526	1.732	0.579
3	4.24	MM	67	22.9	0.0487	1.106	1.084

Crystal Structure Report

Jake Bailey
UCSD Crystallography Lab
5128 Urey Hall
9500 Gilman Dr
La Jolla, CA 92093-0358
Tel: (858) 822-3871
Fax: (858) 822-3872
jbbailey@ucsd.edu

Experimental Summary

The single crystal X-ray diffraction studies were carried out on a Bruker Microstar APEX II CCD diffractometer equipped with Cu K_{α} radiation (λ = 1.54178 Å).

Crystals of the subject compound were grown from DCM/Pentane. A 0.17 x 0.12 x 0.02 mm piece of a crystal was mounted on a Cryoloop with Paratone oil. Data were collected in a nitrogen gas stream at 100(2) K using ϕ and ω scans. Crystal-to-detector distance was 40 mm and exposure time was 2, 4, 6, 8, or 10 seconds depending on the 2θ range per frame using a scan width of 1.25°. Data collection was 98.3 % complete to 67.679° in θ. A total of 32312 reflections were collected covering the indices, −8<=h<=8, −13<=k<=13, −16<=l<=16. 7433 reflections were found to be symmetry independent, with a R_{int} of 0.0497. Indexing and unit cell refinement indicated a **Triclinic** lattice. The space group was found to be ***P*1**. The data were integrated using the Bruker SAINT Software program and scaled using the SADABS software program. Solution by direct methods (SHELXT) produced a complete phasing model consistent with the proposed structure.

All nonhydrogen atoms were refined anisotropically by full-matrix least-squares (SHELXL-2014). All carbon bonded hydrogen atoms were placed using a riding model. Their positions were constrained relative to their parent atom using the appropriate HFIX command in SHELXL-2014. Crystallographic data are summarized in Table 1.

Notes: Great data! Absolute stereochemistry was confirmed (Flack = 0.099(14)). There are two copies of the compound in the asymmetric unit. The crystals behaved poorly at 100K and had to be collected at RT. The terminal alcohols are positionally disordered. The alcohol hydrogens were fixed during refinement. The chemical formula for the compound is: $C_{21}H_{22}ClF_2N_5O$

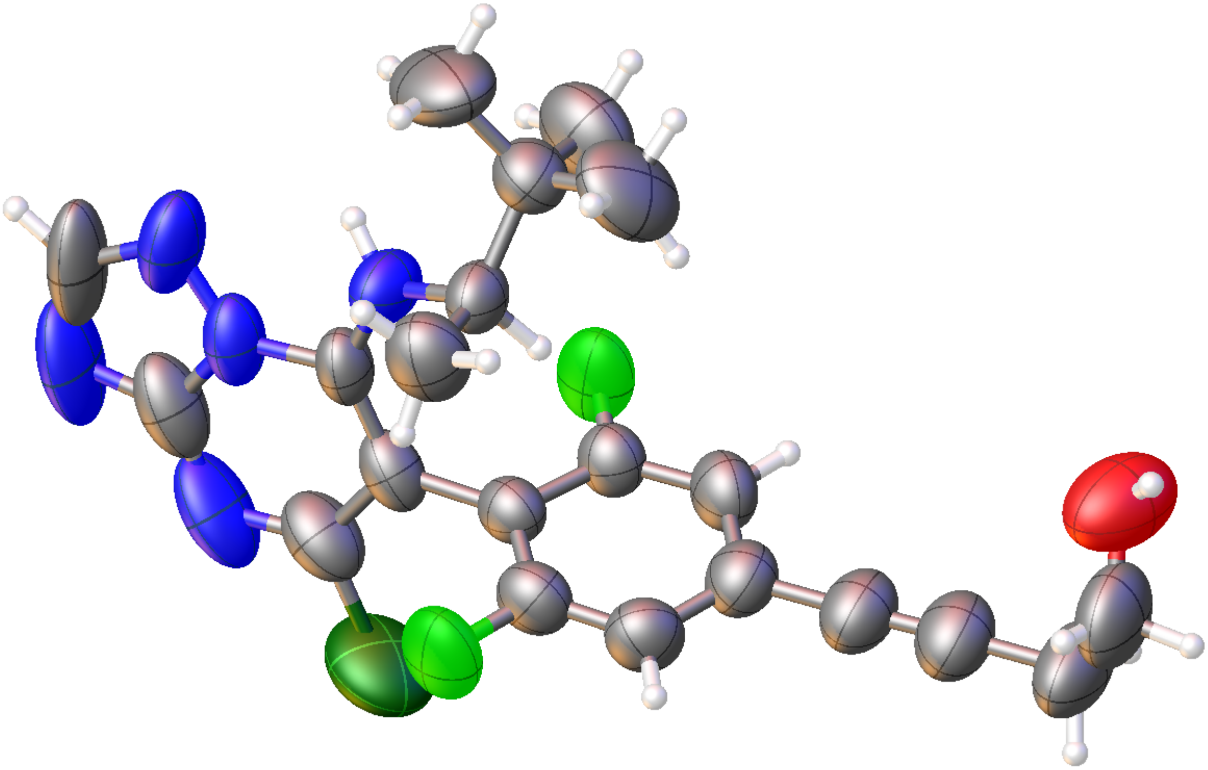

Table 1. Crystal data and structure refinement for Ballatore_SD16_E_RT.

Identification code	ballatore_sd16_e_rt_0m_b	
Empirical formula	C21 H22 Cl F2 N5 O	
Formula weight	433.88	
Temperature	297 K	
Wavelength	1.54178 Å	
Crystal system	Triclinic	
Space group	P1	
Unit cell dimensions	a = 7.3903(5) Å	α= 66.901(3)°.
	b = 11.4003(7) Å	β= 88.273(3)°.
	c = 13.7835(9) Å	γ = 85.305(3)°.
Volume	1064.59(12) $Å^3$	
Z	2	
Density (calculated)	1.354 Mg/m^3	
Absorption coefficient	1.936 mm^{-1}	
F(000)	452	
Crystal size	0.17 x 0.12 x 0.02 mm^3	
Theta range for data collection	3.486 to 68.974°.	
Index ranges	-8<=h<=8, -13<=k<=13, -16<=l<=16	
Reflections collected	32312	
Independent reflections	7433 [R(int) = 0.0497]	
Completeness to theta = 67.679°	98.3 %	
Absorption correction	Semi-empirical from equivalents	
Max. and min. transmission	0.5213 and 0.2929	
Refinement method	Full-matrix least-squares on F^2	
Data / restraints / parameters	7433 / 9 / 563	
Goodness-of-fit on F^2	1.097	
Final R indices [I>2sigma(I)]	R1 = 0.0584, wR2 = 0.1753	
R indices (all data)	R1 = 0.0680, wR2 = 0.1884	
Absolute structure parameter	0.099(14)	
Largest diff. peak and hole	0.216 and -0.375 e.$Å^{-3}$	

Table 2. Atomic coordinates (x 10^4) and equivalent isotropic displacement parameters ($Å^2$x 10^3) for Ballatore_SD16_E_RT. U(eq) is defined as one third of the trace of the orthogonalized U^{ij} tensor.

	x	y	z	U(eq)
Cl(1)	7842(4)	6993(2)	7508(3)	157(1)
F(1)	10594(4)	4311(5)	8754(3)	115(1)
F(2)	4389(4)	4056(4)	8508(3)	107(1)
N(1)	7805(9)	6620(7)	5777(7)	129(3)
N(2)	7717(11)	6206(11)	4215(6)	148(4)
N(3)	7437(8)	4086(7)	5014(4)	112(2)
N(4)	7531(6)	4623(5)	5731(3)	85(1)
N(5)	7413(8)	2730(5)	7117(4)	89(1)
C(1)	7414(16)	128(10)	14454(5)	134(3)
C(2)	6980(17)	1434(9)	14013(6)	135(3)
C(3)	7047(12)	2058(8)	12851(6)	108(2)
C(4)	7130(11)	2564(7)	11918(5)	98(2)
C(5)	7245(8)	3141(6)	10784(5)	83(1)
C(6)	8896(8)	3502(6)	10290(5)	85(1)
C(7)	8976(7)	3990(6)	9211(5)	79(1)
C(8)	7511(6)	4190(5)	8564(4)	67(1)
C(9)	5872(6)	3870(5)	9095(4)	71(1)
C(10)	5719(7)	3344(6)	10169(5)	82(1)
C(11)	7609(6)	4717(5)	7397(4)	74(1)
C(12)	7747(9)	6034(6)	6813(6)	98(2)
C(13)	7690(9)	5857(8)	5220(6)	108(2)
C(14)	7542(11)	5100(15)	4145(6)	142(5)
C(15)	7521(6)	3961(5)	6821(4)	70(1)
C(16)	7895(7)	1678(5)	8130(3)	72(1)
C(17)	9920(9)	1382(9)	8194(6)	111(2)
C(18)	6743(8)	575(5)	8261(5)	86(1)
C(19)	7047(17)	72(12)	7408(10)	156(4)
C(20)	4745(10)	982(10)	8290(9)	130(3)
C(21)	7285(17)	-502(9)	9343(8)	150(4)
O(1)	6524(19)	-649(10)	14203(9)	164(5)
O(1')	7530(40)	-540(20)	15355(10)	176(12)

Cl(1A)	1969(4)	3000(2)	2815(2)	153(1)
F(1A)	-588(4)	5939(5)	1662(3)	108(1)
F(2A)	5720(4)	5597(5)	1715(3)	111(1)
N(1A)	2137(9)	3351(6)	4549(6)	118(2)
N(2A)	2280(9)	3820(10)	6119(5)	131(3)
N(3A)	2827(8)	5887(7)	5302(4)	103(2)
N(4A)	2669(6)	5328(5)	4582(3)	82(1)
N(5A)	3071(8)	7184(5)	3222(3)	87(1)
C(1A)	2425(19)	9894(13)	-4229(13)	172(5)
C(2A)	2556(19)	8487(10)	-3695(7)	148(4)
C(3A)	2604(13)	7887(8)	-2541(6)	114(2)
C(4A)	2640(10)	7393(7)	-1611(5)	98(2)
C(5A)	2627(8)	6800(5)	-480(4)	79(1)
C(6A)	977(8)	6617(6)	66(5)	83(1)
C(7A)	1005(6)	6099(5)	1139(4)	74(1)
C(8A)	2572(6)	5748(4)	1756(4)	66(1)
C(9A)	4147(6)	5918(6)	1176(5)	76(1)
C(10A)	4222(8)	6427(6)	95(5)	84(1)
C(11A)	2526(6)	5237(5)	2928(4)	69(1)
C(12A)	2236(9)	3937(6)	3501(6)	99(2)
C(13A)	2346(8)	4072(7)	5073(5)	100(2)
C(14A)	2584(11)	4916(11)	6193(6)	121(3)
C(15A)	2755(6)	5971(5)	3509(4)	66(1)
C(16A)	3265(7)	8217(5)	2192(3)	72(1)
C(17A)	5219(9)	8591(9)	2078(7)	113(2)
C(18A)	1822(7)	9326(5)	2064(4)	82(1)
C(19A)	2107(15)	10371(8)	973(8)	143(3)
C(20A)	2026(14)	9853(11)	2908(9)	144(3)
C(21A)	-49(9)	8856(11)	2132(8)	130(3)
O(1A)	1360(20)	10591(12)	-3944(10)	184(5)
O(1A')	3020(50)	10610(30)	-3870(20)	184(5)

Table 3. Bond lengths [Å] and angles [°] for Ballatore_SD16_E_RT.

Cl(1)-C(12)	1.719(9)	C(10)-H(10)	0.9300
F(1)-C(7)	1.344(7)	C(11)-C(12)	1.409(8)
F(2)-C(9)	1.335(6)	C(11)-C(15)	1.389(8)
N(1)-C(12)	1.320(11)	C(14)-H(14)	0.9300
N(1)-C(13)	1.375(12)	C(16)-H(16)	0.9800
N(2)-C(13)	1.283(11)	C(16)-C(17)	1.504(9)
N(2)-C(14)	1.318(16)	C(16)-C(18)	1.525(8)
N(3)-N(4)	1.358(9)	C(17)-H(17A)	0.9600
N(3)-C(14)	1.303(12)	C(17)-H(17B)	0.9600
N(4)-C(13)	1.317(10)	C(17)-H(17C)	0.9600
N(4)-C(15)	1.391(6)	C(18)-C(19)	1.501(10)
N(5)-H(5)	0.8600	C(18)-C(20)	1.516(10)
N(5)-C(15)	1.307(7)	C(18)-C(21)	1.553(11)
N(5)-C(16)	1.470(6)	C(19)-H(19A)	0.9600
C(1)-H(1AA)	0.9700	C(19)-H(19B)	0.9600
C(1)-H(1AB)	0.9700	C(19)-H(19C)	0.9600
C(1)-H(1BC)	0.9700	C(20)-H(20A)	0.9600
C(1)-H(1BD)	0.9700	C(20)-H(20B)	0.9600
C(1)-C(2)	1.384(13)	C(20)-H(20C)	0.9600
C(1)-O(1)	1.298(13)	C(21)-H(21A)	0.9600
C(1)-O(1')	1.175(14)	C(21)-H(21B)	0.9600
C(2)-H(2A)	0.9700	C(21)-H(21C)	0.9600
C(2)-H(2B)	0.9700	O(1)-H(1)	0.825(11)
C(2)-C(3)	1.477(10)	O(1')-H(1')	0.81(3)
C(3)-C(4)	1.187(10)	Cl(1A)-C(12A)	1.706(8)
C(4)-C(5)	1.442(9)	F(1A)-C(7A)	1.350(6)
C(5)-C(6)	1.390(9)	F(2A)-C(9A)	1.343(6)
C(5)-C(10)	1.380(8)	N(1A)-C(12A)	1.336(10)
C(6)-H(6)	0.9300	N(1A)-C(13A)	1.309(11)
C(6)-C(7)	1.369(8)	N(2A)-C(13A)	1.356(10)
C(7)-C(8)	1.371(7)	N(2A)-C(14A)	1.330(13)
C(8)-C(9)	1.395(7)	N(3A)-N(4A)	1.385(8)
C(8)-C(11)	1.481(7)	N(3A)-C(14A)	1.312(10)
C(9)-C(10)	1.367(7)	N(4A)-C(13A)	1.361(9)

N(4A)-C(15A)	1.371(6)	C(18A)-C(21A)	1.511(10)
N(5A)-H(5A)	0.8600	C(19A)-H(19D)	0.9600
N(5A)-C(15A)	1.319(7)	C(19A)-H(19E)	0.9600
N(5A)-C(16A)	1.458(6)	C(19A)-H(19F)	0.9600
C(1A)-H(1AC)	0.9700	C(20A)-H(20D)	0.9600
C(1A)-H(1AD)	0.9700	C(20A)-H(20E)	0.9600
C(1A)-H(1AE)	0.9700	C(20A)-H(20F)	0.9600
C(1A)-H(1AF)	0.9700	C(21A)-H(21D)	0.9600
C(1A)-C(2A)	1.475(17)	C(21A)-H(21E)	0.9600
C(1A)-O(1A)	1.235(14)	C(21A)-H(21F)	0.9600
C(1A)-O(1A')	1.22(2)	O(1A)-H(1A)	0.830(13)
C(2A)-H(2AA)	0.9700	O(1A')-H(1A')	0.85(3)
C(2A)-H(2AB)	0.9700		
C(2A)-C(3A)	1.465(11)	C(12)-N(1)-C(13)	115.9(6)
C(3A)-C(4A)	1.181(10)	C(13)-N(2)-C(14)	100.2(7)
C(4A)-C(5A)	1.435(8)	C(14)-N(3)-N(4)	99.7(9)
C(5A)-C(6A)	1.399(8)	N(3)-N(4)-C(15)	125.2(6)
C(5A)-C(10A)	1.381(9)	C(13)-N(4)-N(3)	108.5(6)
C(6A)-H(6A)	0.9300	C(13)-N(4)-C(15)	126.3(7)
C(6A)-C(7A)	1.360(7)	C(15)-N(5)-H(5)	115.1
C(7A)-C(8A)	1.393(7)	C(15)-N(5)-C(16)	129.9(5)
C(8A)-C(9A)	1.375(7)	C(16)-N(5)-H(5)	115.1
C(8A)-C(11A)	1.487(7)	H(1AA)-C(1)-H(1AB)	106.8
C(9A)-C(10A)	1.372(8)	H(1BC)-C(1)-H(1BD)	106.0
C(10A)-H(10A)	0.9300	C(2)-C(1)-H(1AA)	107.0
C(11A)-C(12A)	1.410(8)	C(2)-C(1)-H(1AB)	107.0
C(11A)-C(15A)	1.389(8)	C(2)-C(1)-H(1BC)	105.5
C(14A)-H(14A)	0.9300	C(2)-C(1)-H(1BD)	105.5
C(16A)-H(16A)	0.9800	O(1)-C(1)-H(1AA)	107.0
C(16A)-C(17A)	1.525(8)	O(1)-C(1)-H(1AB)	107.0
C(16A)-C(18A)	1.544(7)	O(1)-C(1)-C(2)	121.2(10)
C(17A)-H(17D)	0.9600	O(1')-C(1)-H(1BC)	105.5
C(17A)-H(17E)	0.9600	O(1')-C(1)-H(1BD)	105.5
C(17A)-H(17F)	0.9600	O(1')-C(1)-C(2)	127.4(15)
C(18A)-C(19A)	1.530(10)	C(1)-C(2)-H(2A)	108.2
C(18A)-C(20A)	1.521(10)	C(1)-C(2)-H(2B)	108.2

C(1)-C(2)-C(3)	116.5(8)	N(5)-C(15)-N(4)	113.6(5)
H(2A)-C(2)-H(2B)	107.3	N(5)-C(15)-C(11)	131.5(5)
C(3)-C(2)-H(2A)	108.2	C(11)-C(15)-N(4)	114.9(5)
C(3)-C(2)-H(2B)	108.2	N(5)-C(16)-H(16)	107.4
C(4)-C(3)-C(2)	178.9(10)	N(5)-C(16)-C(17)	109.6(5)
C(3)-C(4)-C(5)	178.2(7)	N(5)-C(16)-C(18)	108.0(4)
C(6)-C(5)-C(4)	120.6(6)	C(17)-C(16)-H(16)	107.4
C(10)-C(5)-C(4)	120.6(6)	C(17)-C(16)-C(18)	116.5(5)
C(10)-C(5)-C(6)	118.8(5)	C(18)-C(16)-H(16)	107.4
C(5)-C(6)-H(6)	120.4	C(16)-C(17)-H(17A)	109.5
C(7)-C(6)-C(5)	119.1(5)	C(16)-C(17)-H(17B)	109.5
C(7)-C(6)-H(6)	120.4	C(16)-C(17)-H(17C)	109.5
F(1)-C(7)-C(6)	117.9(5)	H(17A)-C(17)-H(17B)	109.5
F(1)-C(7)-C(8)	117.6(5)	H(17A)-C(17)-H(17C)	109.5
C(6)-C(7)-C(8)	124.4(5)	H(17B)-C(17)-H(17C)	109.5
C(7)-C(8)-C(9)	114.3(5)	C(16)-C(18)-C(21)	106.1(5)
C(7)-C(8)-C(11)	124.1(4)	C(19)-C(18)-C(16)	113.4(6)
C(9)-C(8)-C(11)	121.6(4)	C(19)-C(18)-C(20)	108.9(8)
F(2)-C(9)-C(8)	117.3(4)	C(19)-C(18)-C(21)	109.4(8)
F(2)-C(9)-C(10)	118.9(4)	C(20)-C(18)-C(16)	110.4(6)
C(10)-C(9)-C(8)	123.8(5)	C(20)-C(18)-C(21)	108.6(7)
C(5)-C(10)-H(10)	120.3	C(18)-C(19)-H(19A)	109.5
C(9)-C(10)-C(5)	119.4(5)	C(18)-C(19)-H(19B)	109.5
C(9)-C(10)-H(10)	120.3	C(18)-C(19)-H(19C)	109.5
C(12)-C(11)-C(8)	121.0(6)	H(19A)-C(19)-H(19B)	109.5
C(15)-C(11)-C(8)	122.5(4)	H(19A)-C(19)-H(19C)	109.5
C(15)-C(11)-C(12)	116.5(6)	H(19B)-C(19)-H(19C)	109.5
N(1)-C(12)-Cl(1)	115.9(6)	C(18)-C(20)-H(20A)	109.5
N(1)-C(12)-C(11)	126.7(8)	C(18)-C(20)-H(20B)	109.5
C(11)-C(12)-Cl(1)	117.5(6)	C(18)-C(20)-H(20C)	109.5
N(2)-C(13)-N(1)	127.2(9)	H(20A)-C(20)-H(20B)	109.5
N(2)-C(13)-N(4)	113.1(10)	H(20A)-C(20)-H(20C)	109.5
N(4)-C(13)-N(1)	119.7(7)	H(20B)-C(20)-H(20C)	109.5
N(2)-C(14)-H(14)	120.7	C(18)-C(21)-H(21A)	109.5
N(3)-C(14)-N(2)	118.5(9)	C(18)-C(21)-H(21B)	109.5
N(3)-C(14)-H(14)	120.7	C(18)-C(21)-H(21C)	109.5

H(21A)-C(21)-H(21B)	109.5	C(10A)-C(5A)-C(6A)	118.5(5)
H(21A)-C(21)-H(21C)	109.5	C(5A)-C(6A)-H(6A)	120.6
H(21B)-C(21)-H(21C)	109.5	C(7A)-C(6A)-C(5A)	118.9(5)
C(1)-O(1)-H(1)	109.1(11)	C(7A)-C(6A)-H(6A)	120.6
C(1)-O(1')-H(1')	109(2)	F(1A)-C(7A)-C(6A)	118.7(4)
C(13A)-N(1A)-C(12A)	115.4(6)	F(1A)-C(7A)-C(8A)	116.4(5)
C(14A)-N(2A)-C(13A)	105.2(7)	C(6A)-C(7A)-C(8A)	124.9(5)
C(14A)-N(3A)-N(4A)	101.0(8)	C(7A)-C(8A)-C(11A)	122.8(4)
C(13A)-N(4A)-N(3A)	111.5(5)	C(9A)-C(8A)-C(7A)	113.5(5)
C(13A)-N(4A)-C(15A)	124.2(6)	C(9A)-C(8A)-C(11A)	123.7(4)
C(15A)-N(4A)-N(3A)	124.3(5)	F(2A)-C(9A)-C(8A)	117.1(5)
C(15A)-N(5A)-H(5A)	113.8	F(2A)-C(9A)-C(10A)	118.1(5)
C(15A)-N(5A)-C(16A)	132.4(4)	C(10A)-C(9A)-C(8A)	124.7(5)
C(16A)-N(5A)-H(5A)	113.8	C(5A)-C(10A)-H(10A)	120.3
H(1AC)-C(1A)-H(1AD)	106.6	C(9A)-C(10A)-C(5A)	119.4(5)
H(1AE)-C(1A)-H(1AF)	106.4	C(9A)-C(10A)-H(10A)	120.3
C(2A)-C(1A)-H(1AC)	106.6	C(12A)-C(11A)-C(8A)	119.5(6)
C(2A)-C(1A)-H(1AD)	106.6	C(15A)-C(11A)-C(8A)	123.7(4)
C(2A)-C(1A)-H(1AE)	106.2	C(15A)-C(11A)-C(12A)	116.9(5)
C(2A)-C(1A)-H(1AF)	106.2	N(1A)-C(12A)-Cl(1A)	115.6(5)
O(1A)-C(1A)-H(1AC)	106.6	N(1A)-C(12A)-C(11A)	126.1(7)
O(1A)-C(1A)-H(1AD)	106.6	C(11A)-C(12A)-Cl(1A)	118.3(6)
O(1A)-C(1A)-C(2A)	122.8(14)	N(1A)-C(13A)-N(2A)	131.6(7)
O(1A')-C(1A)-H(1AE)	106.2	N(1A)-C(13A)-N(4A)	122.2(6)
O(1A')-C(1A)-H(1AF)	106.2	N(2A)-C(13A)-N(4A)	106.1(8)
O(1A')-C(1A)-C(2A)	125(2)	N(2A)-C(14A)-H(14A)	121.9
C(1A)-C(2A)-H(2AA)	107.4	N(3A)-C(14A)-N(2A)	116.3(8)
C(1A)-C(2A)-H(2AB)	107.4	N(3A)-C(14A)-H(14A)	121.9
H(2AA)-C(2A)-H(2AB)	107.0	N(4A)-C(15A)-C(11A)	115.1(4)
C(3A)-C(2A)-C(1A)	119.6(9)	N(5A)-C(15A)-N(4A)	113.0(5)
C(3A)-C(2A)-H(2AA)	107.4	N(5A)-C(15A)-C(11A)	131.9(4)
C(3A)-C(2A)-H(2AB)	107.4	N(5A)-C(16A)-H(16A)	108.0
C(4A)-C(3A)-C(2A)	179.4(8)	N(5A)-C(16A)-C(17A)	108.5(5)
C(3A)-C(4A)-C(5A)	178.3(8)	N(5A)-C(16A)-C(18A)	109.7(4)
C(6A)-C(5A)-C(4A)	120.1(6)	C(17A)-C(16A)-H(16A)	108.0
C(10A)-C(5A)-C(4A)	121.3(6)	C(17A)-C(16A)-C(18A)	114.3(5)

C(18A)-C(16A)-H(16A)	108.0
C(16A)-C(17A)-H(17D)	109.5
C(16A)-C(17A)-H(17E)	109.5
C(16A)-C(17A)-H(17F)	109.5
H(17D)-C(17A)-H(17E)	109.5
H(17D)-C(17A)-H(17F)	109.5
H(17E)-C(17A)-H(17F)	109.5
C(19A)-C(18A)-C(16A)	106.9(5)
C(20A)-C(18A)-C(16A)	111.3(5)
C(20A)-C(18A)-C(19A)	109.8(7)
C(21A)-C(18A)-C(16A)	109.3(6)
C(21A)-C(18A)-C(19A)	110.1(7)
C(21A)-C(18A)-C(20A)	109.5(6)
C(18A)-C(19A)-H(19D)	109.5
C(18A)-C(19A)-H(19E)	109.5
C(18A)-C(19A)-H(19F)	109.5
H(19D)-C(19A)-H(19E)	109.5
H(19D)-C(19A)-H(19F)	109.5
H(19E)-C(19A)-H(19F)	109.5
C(18A)-C(20A)-H(20D)	109.5
C(18A)-C(20A)-H(20E)	109.5
C(18A)-C(20A)-H(20F)	109.5
H(20D)-C(20A)-H(20E)	109.5
H(20D)-C(20A)-H(20F)	109.5
H(20E)-C(20A)-H(20F)	109.5
C(18A)-C(21A)-H(21D)	109.5
C(18A)-C(21A)-H(21E)	109.5
C(18A)-C(21A)-H(21F)	109.5
H(21D)-C(21A)-H(21E)	109.5
H(21D)-C(21A)-H(21F)	109.5
H(21E)-C(21A)-H(21F)	109.5
C(1A)-O(1A)-H(1A)	108.6(14)
C(1A)-O(1A')-H(1A')	107(3)

Symmetry transformations used to generate equivalent atoms:

Table 4. Anisotropic displacement parameters ($Å^2 x 10^3$) for Ballatore_SD16_E_RT. The anisotropic displacement factor exponent takes the form: $-2\pi^2[h^2 a^{*2}U^{11} + ... + 2 h k a^* b^* U^{12}]$

	U^{11}	U^{22}	U^{33}	U^{23}	U^{13}	U^{12}
Cl(1)	197(2)	74(1)	193(3)	-45(1)	21(2)	-24(1)
F(1)	66(2)	161(4)	108(3)	-38(2)	10(2)	-31(2)
F(2)	57(2)	162(4)	90(2)	-37(2)	-1(1)	-10(2)
N(1)	120(4)	76(4)	129(6)	27(4)	19(4)	-8(3)
N(2)	122(5)	169(8)	70(4)	44(5)	1(3)	-13(5)
N(3)	103(3)	161(6)	51(3)	-19(3)	-4(2)	-3(3)
N(4)	71(2)	102(4)	54(2)	-1(2)	-1(2)	-2(2)
N(5)	128(4)	77(3)	56(2)	-18(2)	-8(2)	-16(2)
C(1)	186(9)	129(8)	65(4)	-16(4)	4(4)	-13(6)
C(2)	218(10)	111(6)	71(4)	-30(4)	5(5)	-24(6)
C(3)	141(6)	100(5)	86(5)	-39(4)	9(4)	-7(4)
C(4)	127(5)	90(4)	76(4)	-33(3)	0(3)	-4(3)
C(5)	94(4)	84(4)	74(3)	-34(3)	5(3)	-6(3)
C(6)	80(3)	90(4)	83(4)	-30(3)	-10(2)	-9(3)
C(7)	65(3)	83(3)	88(4)	-32(3)	2(2)	-10(2)
C(8)	60(2)	69(3)	74(3)	-30(2)	7(2)	-10(2)
C(9)	62(2)	77(3)	70(3)	-24(2)	3(2)	-5(2)
C(10)	78(3)	89(3)	80(3)	-33(3)	19(2)	-12(2)
C(11)	61(2)	63(3)	76(3)	-5(2)	8(2)	-5(2)
C(12)	91(4)	66(3)	118(5)	-17(4)	14(3)	-8(3)
C(13)	83(4)	94(5)	98(5)	14(4)	7(3)	-5(3)
C(14)	98(5)	221(13)	47(3)	12(5)	1(3)	-7(6)
C(15)	66(2)	78(3)	49(2)	-6(2)	3(2)	-11(2)
C(16)	92(3)	68(3)	52(2)	-17(2)	2(2)	-10(2)
C(17)	91(4)	124(5)	104(5)	-28(4)	-7(3)	-16(3)
C(18)	96(3)	77(3)	85(3)	-29(3)	7(3)	-16(3)
C(19)	181(9)	160(9)	185(9)	-125(8)	39(7)	-56(7)
C(20)	91(4)	137(7)	160(8)	-54(6)	24(4)	-17(4)
C(21)	182(9)	85(5)	140(8)	7(5)	-15(6)	-37(5)
O(1)	244(13)	117(7)	132(7)	-48(6)	-38(7)	-8(7)
O(1')	310(30)	133(16)	45(8)	12(8)	17(11)	-27(17)

Cl(1A)	202(2)	74(1)	180(2)	-40(1)	-38(2)	-34(1)
F(1A)	56(1)	163(4)	89(2)	-31(2)	5(1)	-18(2)
F(2A)	61(2)	154(4)	108(3)	-43(2)	3(2)	1(2)
N(1A)	117(4)	78(4)	112(5)	19(4)	-14(3)	-30(3)
N(2A)	108(4)	159(7)	66(3)	24(4)	1(3)	-22(4)
N(3A)	110(4)	134(5)	53(3)	-23(3)	2(2)	-8(3)
N(4A)	77(2)	93(3)	52(2)	-3(2)	2(2)	-12(2)
N(5A)	138(4)	72(3)	47(2)	-17(2)	3(2)	-16(2)
C(1A)	168(9)	161(11)	234(14)	-131(11)	11(9)	-4(8)
C(2A)	243(12)	121(7)	85(5)	-44(5)	24(6)	-32(7)
C(3A)	164(7)	107(5)	80(4)	-43(4)	21(4)	-34(4)
C(4A)	129(5)	95(4)	74(4)	-37(3)	19(3)	-29(4)
C(5A)	101(4)	70(3)	71(3)	-33(2)	7(2)	-17(2)
C(6A)	79(3)	94(4)	75(3)	-30(3)	-5(2)	-13(2)
C(7A)	62(2)	85(3)	72(3)	-27(2)	7(2)	-19(2)
C(8A)	64(2)	56(2)	73(3)	-22(2)	2(2)	-7(2)
C(9A)	61(3)	81(3)	93(4)	-42(3)	3(2)	-5(2)
C(10A)	80(3)	90(4)	89(4)	-43(3)	22(3)	-19(3)
C(11A)	59(2)	64(3)	73(3)	-14(2)	0(2)	-10(2)
C(12A)	92(4)	64(3)	116(5)	-6(3)	-11(3)	-17(3)
C(13A)	81(3)	95(5)	76(4)	23(4)	-7(3)	-21(3)
C(14A)	108(5)	157(8)	60(4)	-1(4)	0(3)	-13(5)
C(15A)	64(2)	65(3)	55(2)	-7(2)	-1(2)	-6(2)
C(16A)	90(3)	66(3)	54(2)	-17(2)	3(2)	-10(2)
C(17A)	76(3)	136(6)	116(5)	-37(4)	10(3)	-21(3)
C(18A)	82(3)	75(3)	82(3)	-25(2)	0(2)	-5(2)
C(19A)	167(8)	79(4)	132(7)	8(4)	4(5)	15(4)
C(20A)	156(7)	144(8)	183(9)	-120(8)	13(6)	-3(6)
C(21A)	78(4)	156(7)	155(8)	-58(6)	8(4)	-20(4)
O(1A)	241(14)	145(8)	146(7)	-35(6)	72(10)	-52(10)
O(1A')	241(14)	145(8)	146(7)	-35(6)	72(10)	-52(10)

Table 5. Hydrogen coordinates (x 10^4) and isotropic displacement parameters ($Å^2$x 10^3) for Ballatore_SD16_E_RT.

	x	y	z	U(eq)
H(5)	6996	2500	6648	107
H(1AA)	8683	-13	14298	161
H(1AB)	7323	-139	15213	161
H(1BC)	6539	-239	14166	161
H(1BD)	8578	-5	14153	161
H(2A)	7802	1838	14300	162
H(2B)	5764	1601	14238	162
H(6)	9931	3414	10687	102
H(10)	4597	3126	10482	98
H(14)	7493	5045	3491	171
H(16)	7546	1979	8690	87
H(17A)	10229	682	8849	166
H(17B)	10503	2121	8155	166
H(17C)	10319	1153	7617	166
H(19A)	6546	691	6757	234
H(19B)	6463	-710	7598	234
H(19C)	8326	-89	7325	234
H(20A)	4551	1359	8801	196
H(20B)	4050	249	8481	196
H(20C)	4367	1597	7607	196
H(21A)	8483	-870	9297	224
H(21B)	6438	-1151	9528	224
H(21C)	7267	-150	9872	224
H(1)	6885	-1392	14572	246
H(1')	6648	-364	15660	264
H(5A)	3184	7408	3743	104
H(1AC)	3631	10163	-4211	206
H(1AD)	2154	10096	-4964	206
H(1AE)	2991	10089	-4913	206
H(1AF)	1144	10156	-4362	206

H(2AA)	1532	8182	-3928	178
H(2AB)	3644	8167	-3952	178
H(6A)	-120	6845	-299	100
H(10A)	5337	6520	-248	101
H(14A)	2622	4989	6841	145
H(16A)	3043	7889	1651	86
H(17D)	5400	9214	1379	169
H(17E)	6035	7847	2201	169
H(17F)	5452	8949	2583	169
H(19D)	2931	10950	1029	214
H(19E)	964	10831	701	214
H(19F)	2604	9988	506	214
H(20D)	1776	9210	3588	217
H(20E)	1188	10589	2771	217
H(20F)	3244	10092	2900	217
H(21D)	-122	8420	1665	195
H(21E)	-941	9571	1932	195
H(21F)	-271	8278	2842	195
H(1A)	985	11213	-4476	275
H(1A')	4062	10789	-4140	275
